# Impact of Green Cosolvents on the Catalytic Dehydrogenation of Formic Acid: The Case of Iridium Catalysts Bearing NHC-phosphane Ligands

**DOI:** 10.1021/acs.inorgchem.1c02132

**Published:** 2021-09-24

**Authors:** Ana Luque-Gómez, Susana García-Abellán, Julen Munarriz, Victor Polo, Vincenzo Passarelli, Manuel Iglesias

**Affiliations:** † Departamento Química Inorgánica-Instituto Síntesis Química y Catálisis Homogénea (ISQCH), Universidad de Zaragoza−CSIC, C/Pedro Cerbuna 12, 50009 Zaragoza, Spain; ‡ Departamento Química Física y Analítica, Universidad de Oviedo, Avda. Julian Clavería 8, 33006 Oviedo, Spain; § Departamento Química Física-Instituto de Biocomputación y Física de Sistemas Complejos (BIFI), Universidad de Zaragoza, Pedro Cerbuna 12, 50009 Zaragoza, Spain

## Abstract

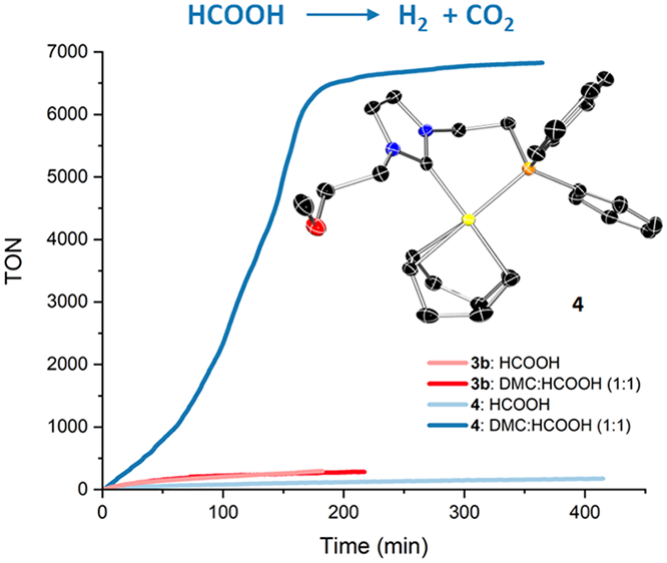

The catalysts [Ir(COD)(κ^3^-*P*,*C*,*P′*-PC^NHC^P)]BF_4_ and [Ir(COD)(κ^2^-*P,C*-PC^NHC^O)]BF_4_ proved to be active in the solventless dehydrogenation of formic acid. The impact of various cosolvents on the activity was evaluated, showing an outstanding improvement of the catalytic performance of [Ir(COD)(κ^2^-*P,C*-PC^NHC^O)]BF_4_] in “green” organic carbonates: namely, dimethyl carbonate (DMC) and propylene carbonate (PC). The TOF_1h_ value for [Ir(COD)(κ^2^-*P,C*-PC^NHC^O)]BF_4_ increases from 61 to 988 h^–1^ upon changing from solventless conditions to a 1/1 (v/v) DMC/HCOOH mixture. However, in the case of [Ir(COD)(PC^NHC^P)]BF_4_, only a marginal improvement from 156 to 172 h^–1^ was observed under analogous conditions. Stoichiometric experiments allowed the identification of various key reaction intermediates, providing valuable information on their reactivity. Experimental data and DFT calculations point to the formation of dinuclear species as the catalyst deactivation pathway, which is prevented in the presence of DMC and PC.

## Abstract 2

Green cosolvents trigger outstanding performance in catalyzed HCOOH dehydrogenation.

## 1 Introduction

One of the main challenges that our society needs to face in the coming decades entails the mitigation of climate change while meeting the ever-increasing global energy demand.^1^ The use of hydrogen as an energy vector has been proposed as an alternative to reduce anthropogenic CO_2_ emissions.^2^ However, the implementation of a hydrogen-based economy presents several issues that need to be addressed in order to enable the viability of this endeavor. One of them involves the storage and transportation of hydrogen, which raises economic and safety concerns. Hydrogen is the fuel with the highest gravimetric energy density; conversely, its low volumetric energy density at ambient temperature and atmospheric pressure is a major drawback. Therefore, molecular hydrogen has to be stored in pressurized tanks (350–700 bar) or as a liquid at cryogenic temperatures (below −253 °C). The use of LOHCs (liquid organic hydrogen carriers) instead of molecular hydrogen brings about some key advantages and circumvents the need for gas compression or cryogenic technologies.^3^ Formic acid in particular has a higher volumetric energy density in comparison to compressed hydrogen, while it presents low toxicity and a high hydrogen content.^4^

The homogeneously catalyzed dehydrogenation of HCOOH allows the generation of an equimolar H_2_/CO_2_ mixture virtually free of CO,^5^ which is crucial for the optimal performance of hydrogen fuel cells, since concentrations of CO over 10 ppm may damage the Pt electrode.^6^ Finally, the hydrogenation of CO_2_ would regenerate HCOOH, providing a C-neutral cycle.

Since the seminal work by Beller^7^ and Laurenczy^8^ in 2008, a great variety of new systems for the dehydrogenation of HCOOH have been developed.^5,9^ This increasing interest has led to a number of theoretical studies performed by us^10^ as well as different groups,^11^ which aim to unravel the reaction mechanism by which these systems operate and ultimately facilitate the development of new catalysts with improved properties. The examination of the catalytic cycles by which these systems operate shows a rich diversity of mechanisms—including inner- and outer-sphere reactions—with different rate-determining steps.^12^

The nature of the catalyst, i.e., the metal center and the ligand system, and the solvent and additives determine the reaction mechanism. The development of catalysts that operate under solventless conditions is desirable, due to the fact that the volumetric energy density of the reaction mixture decreases upon addition of a solvent.^10a,13^ However, the use of a solvent has been often described to enhance the activity of a system.^14^ Consequently, the use of small amounts of a cosolvent may be a suitable strategy to optimize the activity of the catalyst. The volumetric capacity of formic acid is 53 g H_2_/L, with an energy density of 1.77 kWh/L, while the volumetric capacity of compressed hydrogen (35 MPa, 27 °C) is 23 g of H_2_/L, with an energy density of 0.767 kWh/L. Therefore, diluting HCOOH with the same volume of a given solvent would still render a mixture with a volumetric capacity and energy density higher than those of compressed hydrogen (26.5 g H_2_/L and 0.885 kWh/L, respectively). On these grounds, solvent effects on the activity of HCOOH dehydrogenation catalysts have been somewhat overlooked from a mechanistic viewpoint. It is worth noting that, in certain cases, the solvent has been described to play an active role in the reaction mechanism: namely, in systems where outer-sphere interactions between the ligands and water—acting as a solvent—direct the protonation of a hydride species, thus generating H_2_.^15^

In this work, we describe the synthesis of two cationic Ir(I)-COD (COD = 1,5-cyclooctadiene) catalysts that feature a PC^NHC^P and a PC^NHC^O ligand based on an N-heterocyclic carbene (NHC) scaffold. Both catalysts proved to be active in the solventless dehydrogenation of HCOOH. The effect of a variety of cosolvents on the activity of both complexes was explored, showing that, in sharp contrast with the PC^NHC^P-Ir(I)-COD complex, its PC^NHC^O analogue experiences a remarkable performance enhancement in organic carbonates. This atypical behavior was studied from a mechanistic perspective, which includes kinetic isotope effect (KIE) experiments as well as DFT studies. Both approaches led to a feasible reaction mechanism in which the rate-determining step is the hydride abstraction of the formate ligand, while they explain the aforementioned different behavior exhibited by the PC^NHC^P-Ir(I)-COD and PC^NHC^O-Ir(I)-COD complexes.

## 2 Results and Discussion

### 2.1 Synthesis and Characterization of [Ir(COD)(κ^3^-*P*,*C*,*P′*-PC^NHC^P)]Cl (**3a**), [Ir(COD)(PC^NHC^P)]BF_4_ (**3b**), and [Ir(COD)(κ^2^-*P*,*C*-PC^NHC^O)]BF_4_ (**4**)

**Scheme 1:**
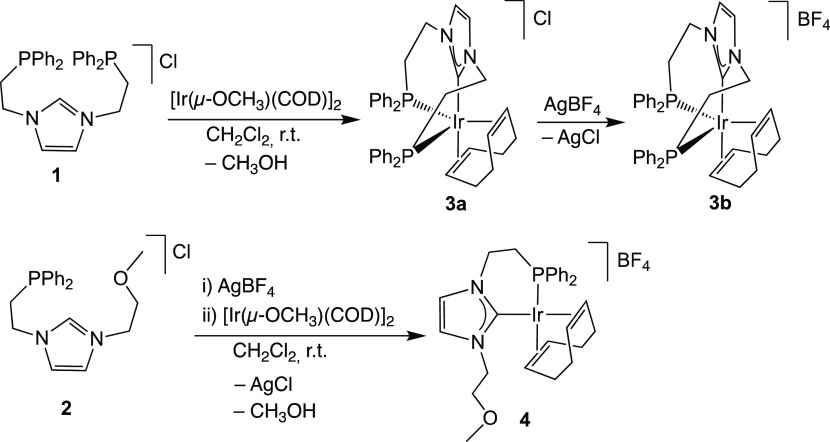
Synthesis of Complexes **3** and **4** from Imidazolium Salts **1** and **2**

The ligand precursors, imidazolium salts **1** and **2**,^30^ were reacted with 0.5 equiv of [Ir(μ-OCH_3_)(COD)]_2_ in dichloromethane at room temperature—with the methoxide ligand acting as an internal base for the deprotonation of the corresponding imidazolium salt—to afford complexes **3a** and **4**, [Ir(COD)(κ^3^-*P*,*C*,*P′*-PC^NHC^P)]Cl (PC^NHC^P = 1,3-bis(2-(diphenylphosphanyl)ethyl)imidazol-2-ylidene) and [Ir(COD)(κ^2^-*P*,*C*-PC^NHC^O)]BF_4_ (PC^NHC^O = 1-(2-(diphenylphosphanyl)ethyl)-3-(2-methoxyethyl)imidazol-2-ylidene), respectively. Complexes **3a** and **4** were obtained in good yields as off-white and deep red solids, respectively. Complex **3a** was readily transformed into **3b** by a reaction with AgBF_4_ at room temperature in CH_2_Cl_2_ (Scheme 1).

Note that complex **4** was synthesized from the tetrafluoroborate salt **2** instead of its related chloride salt. This divergent synthetic strategy was employed due to the fact that an intractable mixture of complexes was obtained upon starting from the chloride salt.

The ^31^P{^1^H} spectrum of complex **3a** shows a single peak at δ −21.0 ppm, suggesting the presence of a symmetry plane that is perpendicular to the imidazolium ring and renders both phosphorus nuclei equivalent. This is supported by the ^1^H NMR spectrum in CD_2_Cl_2_, which shows the CHs of the imidazolium moiety as a singlet at δ 7.43 ppm. Furthermore, the ^13^C{^1^H} NMR spectrum shows a triplet at δ 143.0 ppm (^2^*J*_C–P_ = 14.0 Hz) that corresponds to the carbenic carbon bound to the Ir center and coupled with the two equivalent phosphorus nuclei. The NMR spectra of **3b** show no substantial changes in comparison with those of **3a**.

In the case of complex **4**, the ^31^P{^1^H} NMR spectrum presents a singlet at δ 17.9 ppm, which confirms the coordination of the phosphane. The ^1^H NMR spectrum of **4** in CD_2_Cl_2_ shows the CHs of the imidazole ring as two apparent triplets at δ 7.12 and 7.09 ppm with a coupling constant of 2.0 Hz, which discards the possibility of C4 or C5 coordination. Representative peaks in the ^13^C{^1^H} NMR spectrum of **4** are two singlets at δ 121.7 and 122.4 ppm, which correspond to the C4 and C5 atoms at the imidazole ring, and the carbene carbon, which appears as a doublet at δ 171.6 ppm (^2^*J*_C–P_ = 13.7 Hz).

**Figure 1:**
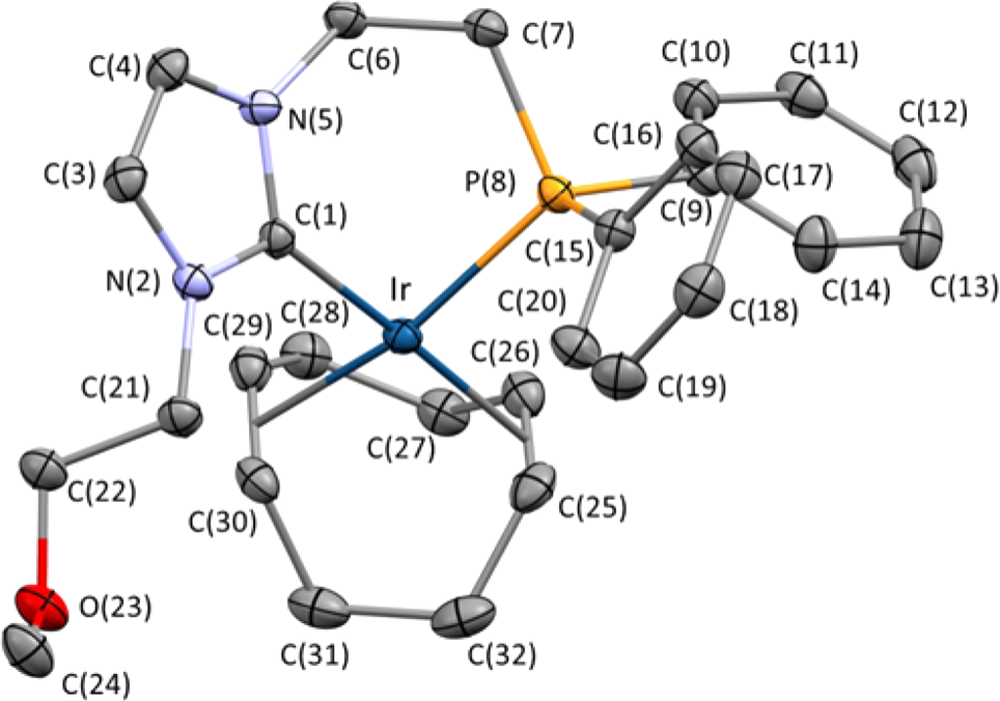
ORTEP view of **4** (ellipsoids are drawn at the 50% probability level). Hydrogen atoms, the lattice THF molecule, and the BF_4_^–^ counterion are omitted for clarity. Selected bond lengths (Å) and angles (deg): C(1)–Ir 2.032(3), P(8)–Ir 2.2969(7), ct1–Ir 2.08103(12), ct2–Ir 2.07369(13), C(25)–C(26) 1.391(4), C(29)–C(30) 1.390(4), N(5)–C(1)–N(2) 104.0(2), C(1)–Ir–P(8) 84.97(7), ct1–Ir–ct2 85.614(5). ct1 and ct2 are the centroids of the C(25) and C(26) atoms and of the C(29) and C(30) atoms, respectively.

The connectivity of **4** was confirmed by a single-crystal X-ray diffraction analysis (Figure 1). The PC^NHC^O ligand behaves as a chelate, with a P(8)–Ir–C(1) bite angle of 84.97(7)°, which results in a slightly distorted square-planar environment for the iridium center.

**Figure 2:**
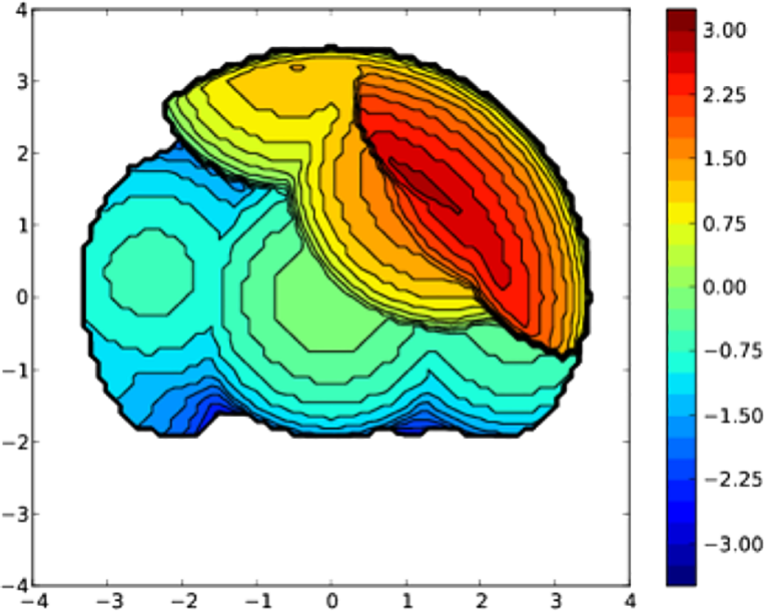
Topographic steric map for the PC^NHC^O ligand in **4** calculated with a sphere radius of 3.50 Å, a bond radius of 1.17 Å,and a mesh spacing of 0.10 (excluding H atoms).

Notably, similar Ir–ct(1) (2.08103(12) Å) and Ir–ct(2) (2.07369(13) Å) lengths are observed, ct1 and ct2 being the centroids of the C(25) and C(26) atoms and of the C(29) and C(30) atoms, respectively, which clearly suggest that the NHC moiety and the phosphano group in **4** exert comparable *trans* influences. Accordingly, almost identical C(25)–C(26) (1.391(4) Å) and C(29)–C(30) (1.390(4) Å) bond lengths are observed. The imidazolium ring C(1)–N(2)–C(3)–C(4)–N(5) forms a 59.9° angle with respect to the coordination plane Ir–ct(1)–ct(2)–C(1)–P(8), thus deviating from the least sterically hindered perpendicular arrangement. This behavior can be ascribed to the constraint originating from the coordination of the phosphane-containing wingtip group, which prevents free rotation about the Ir–C(1) bond. Nonetheless, it is worth mentioning that the pitch and yaw angles of the NHC moiety (θ = 0.3°, ψ = 3.3°)^16^ point to an almost ideal arrangement of the NHC moiety with respect to the Ir–C(1) bond. Finally, a visual inspection of the six-membered ring Ir–C(1)–N(5)–C(6)–C(7)–P(8) and the corresponding Cremer–Pople^17^ parameters (*q* = 0.9518 Å, θ = 81.70°, ψ = 359.40°) clearly indicate a boat conformation of this ring, reasonably brought about by the presence of the NHC fused ring along with the sp^2^ hybridization of its atoms. The percent buried volume (%*V*_bur_) steric parameter of the NHC in **4** was calculated using SambVca 2.0,^18^ giving a value of 46.6, which is similar to those reported by Dorta et al. for Ir-COD complexes featuring saturated imidazol-2-ylidene ligands with substituted naphthyl wingtip groups.^19^ The steric map for the PC^NHC^O ligand is shown in Figure 2.

### 2.2 Reactivity of **3b** and **4** with Carbon Monoxide and Molecular Hydrogen

**Scheme 2:**
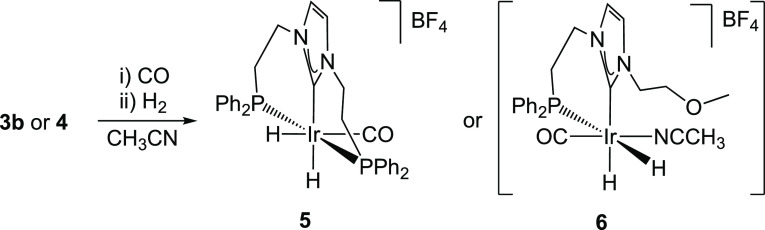
Synthesis of Complexes **5** and **6** by Sequential Carbonylation–Hydrogenation of **3b** and **4**

The reactivity of **3b** and **4** with carbon monoxide and hydrogen was explored in order to achieve a better understanding of the structural features of both complexes after COD release, since these new complexes are likely reminiscent of the active species formed under catalytic conditions. The reaction of complex **3b** with carbon monoxide (2 bar) affords an unstable complex and free COD. Attempts to isolate the carbonylated complex were unsuccessful, likely due to the initial formation of a bis-carbonyl species that decomposes by loss of a CO ligand upon removing the carbon monoxide atmosphere.^20^ Alternatively, **3b** was placed under a CO atmosphere that was subsequently replaced by H_2_ to afford the dihydride complex [Ir(H)_2_(CO)(κ^2^-*P*,*C*-PC^NHC^O)]BF_4_ (**5**) in good yield as a yellow solid. Following the same synthetic procedure, **4** was converted into a new complex, tentatively formulated as [Ir(H)_2_(CH_3_CN)(CO)(κ^2^-*P*,*C*-PC^NHC^O)]BF_4_ (**6**). The low stability of **6** prevented its isolation and was, therefore, only characterized in solution (Scheme 2).

In the ^1^H NMR spectrum of **5** in CD_2_Cl_2_, two doublets of triplets are observed at high field, δ −10.44 and −11.52 ppm, which correspond to two different hydride ligands, each coupled with the neighboring hydride (*J*_HH_ = 3.7 Hz) and the two equivalent P nuclei (*J*_HP_ = 15.6 and 14.2 Hz, respectively). In addition, the ^1^H NMR spectrum of **5** shows a singlet at δ 7.13 ppm in CD_2_Cl_2_ that corresponds to the two protons of the imidazole ring, which suggests the presence of a symmetry plane that contains the Ir center, the two hydrides, and the carbonyl ligand. The ^31^P{^1^H} spectrum of complex **5** shows a singlet at δ −3.9 ppm, which confirms the *trans* coordination of both phosphanes. The structure of **5** is, therefore, similar to that previously proposed by us for a related complex that features an N-heterocyclic olefin instead of the NHC moiety.^20^

At high field, the ^1^H NMR spectrum of **6** in CD_2_Cl_2_ shows two doublets of doublets that correspond to the two hydride ligands at δ −8.73 (*J*_HP_ = 135.6 Hz, *J*_HH_ = 4.7 Hz) and −18.97 (*J*_HP_ = 9.8 Hz, *J*_HH_ = 4.7 Hz) ppm. The two distinct H–P coupling constants suggest that the former is *trans* to the phosphane while the latter is *cis*. Note that the ^1^H{^31^P} NMR spectrum of **5** shows two doublets at δ −8.73 and −18.97 ppm with coupling constants of 4.7 Hz, and the phosphane signal in the ^31^P{^1^H} NMR spectrum appears as a singlet at −3.9 ppm. On these grounds, we proposed the structure depicted in Scheme 2. The possibility that the hydride *trans* to the NHC is in fact *cis* to the NHC and phosphane moieties cannot be discarded, which would place the CO ligand *trans* to the NHC.

### 2.3 Catalytic Activity of Complexes **3b** and **4** in the Dehydrogenation of Formic Acid

**Figure 3:**
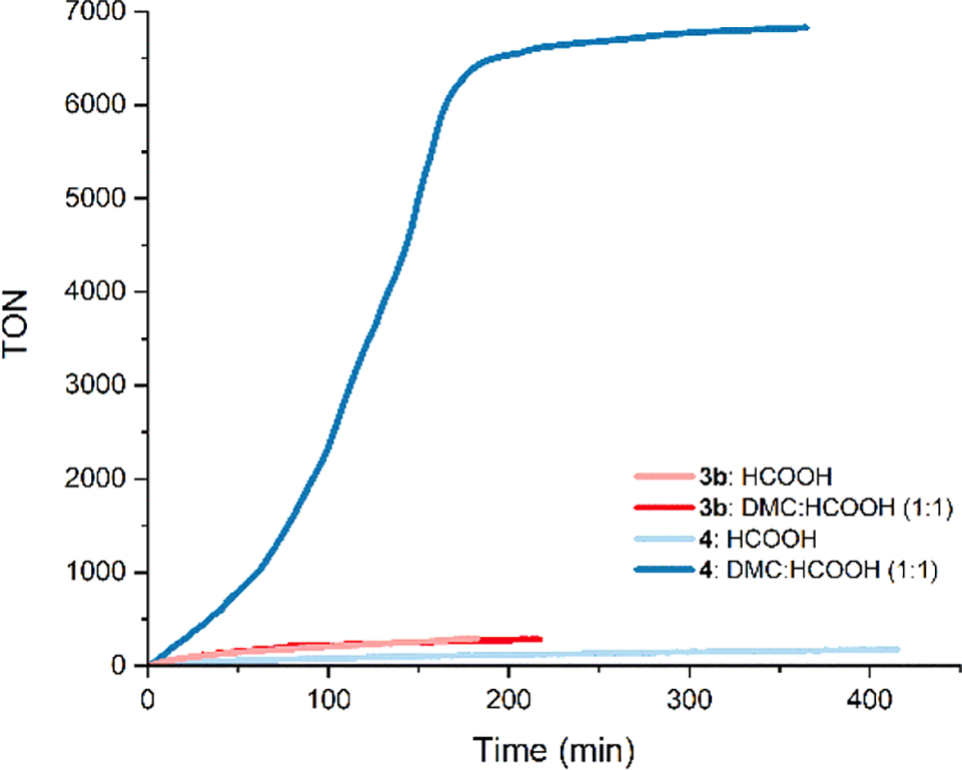
Reaction profiles for the dehydrogenation of HCOOH (0.016 mol % of **3b** or **4**, 30 mol % of HCOONa at 80 °C) with and without DMC as solvent.

Initially, we explored the catalytic activity of **3b** in the dehydrogenation of neat formic acid, employing the same reaction conditions that we previously applied to related systems.^10a^**3b** proved active for the dehydrogenation of HCOOH under solventless conditions in the presence of 0.016 mol % of the catalyst and 30 mol % of HCOONa at 80 °C. In order to evaluate the effect of removing one of the strongly coordinating phosphane side arms, we prepared **4**, which features an ether function at the wingtip group that could behave as a hemilabile ligand.^21^ Under reaction conditions analogous to those described above, **4** shows lower catalytic activity in comparison to **3b**. A plausible explanation is that the preactivation of **3b** or **4** likely involves the loss of the COD ligand, which leads to the formation of unsaturated species. The presence of the PC^NHC^P ligand in **3b** would bring about a more stable unsaturated complex in comparison to that resulting from **4**, which agrees with the latter being less active. A related behavior was also observed in the case of **5** and **6** because, upon loss of the COD ligand, the PC^NHC^O-Ir complex is less stable than its related PC^NHC^P complex. Therefore, we decided to explore the use of a cosolvent able to reversibly occupy the vacant coordination sites generated along the catalytic cycle. Organic carbonates, such as dimethyl carbonate (DMC) and propylene carbonate (PC), are considered green solvents,^22^ which we believed that could act as labile ligands able to prevent decomposition pathways. The catalytic dehydrogenation of HCOOH was attempted in a 1/1 (v/v) mixture of HCOOH and DMC with 0.016 mol % of **3b** or **4** and 30 mol % of HCOONa at 80 °C. In the case of **3b**, the TOF_1h_ value changes from 156 h^–1^ under solventless conditions to 172 h^–1^ in a 1/1 (v/v) DMC/HCOOH solution; on the other hand, **4** undergoes a dramatic increase in the TOF_1h_ value from 61 to 988 h^–1^ (Figure 3).

Remarkably, under solventless conditions, a loss of activity was observed for **4** during the course of the reaction. Namely, the TOF value decreases from 126 h^–1^ (after 10 min) to 61 h^–1^ (after 1 h), which deviates from the expected linear behavior at high HCOOH concentrations (Figure S4). Moreover, an activity boost is observed upon increasing the amount of DMC: namely, in a 4/1 (v/v) DMC/HCOOH mixture, a TOF_1h_ value of 1137 h^–1^ is obtained. Note that higher DMC to HCOOH ratios lead to an activity drop that can be attributed to solubility problems, because **3b** and **4** are barely soluble in neat DMC. In summary, in the presence of DMC, the activity of **4** undergoes an outstanding increase, while the performance of **3b** is only marginally affected. This suggests that the unsaturated species resulting from **4** is more active than that derived from **3b** but that the former requires the assistance of a coordinating solvent which precludes its deactivation.

**Figure 4:**
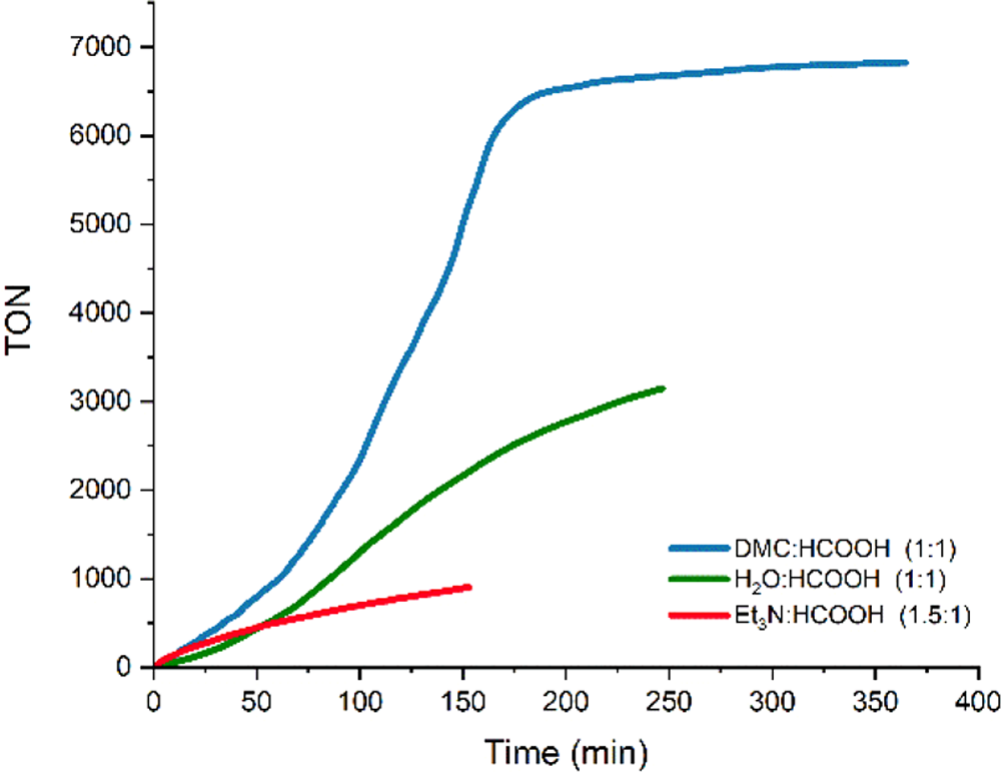
Reaction profiles for the dehydrogenation of HCOOH (0.016 mol % of **4**, 30 mol % of HCOONa at 80 °C) in various solvents.

In order to explore whether the stabilization effect could extend to other solvents (Figure 4), the activity of both catalysts was evaluated in a 1/1 (v/v) H_2_O/HCOOH mixture and the Et_3_N/HCOOH azeotrope (which is a 2/5 Et_3_N/HCOOH mixture or 1.5/1 v/v). The use of 0.016 mol % of **4** and 30 mol % of HCOONa at 80 °C resulted in TOF_1h_ values of 564 and 506 h^–1^ in H_2_O and HCOOH/Et_3_N, respectively. These TOF values undoubtedly cause an improvement in the results obtained without solvent but are markedly lower than those observed in DMC (988 h^–1^).

**Figure 5:**
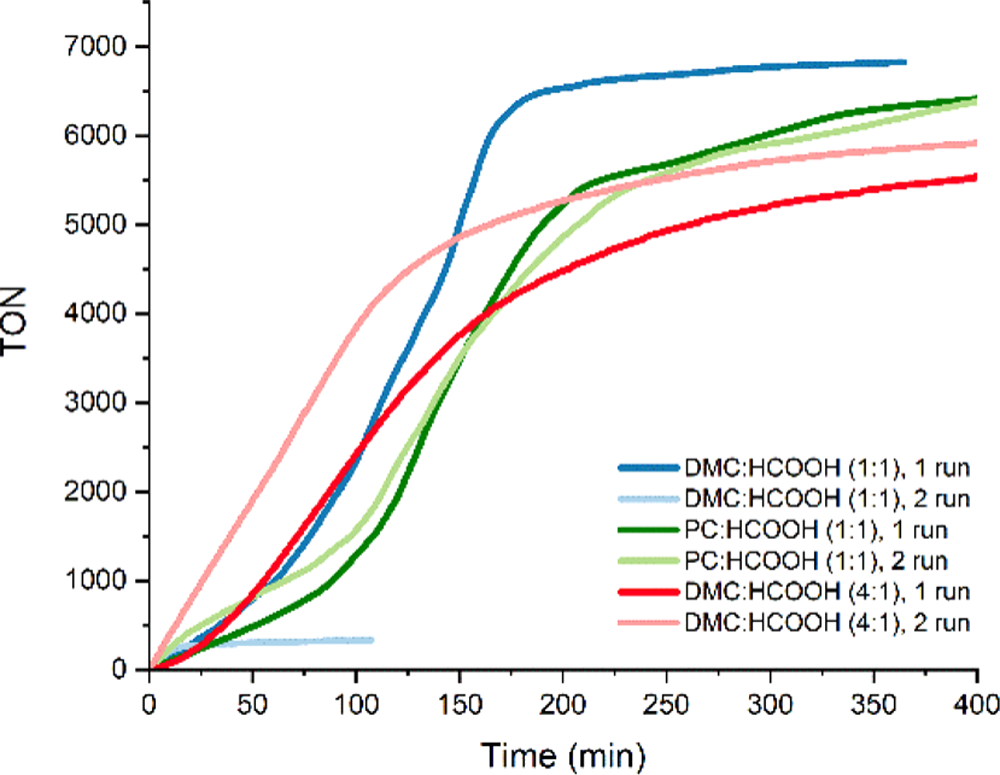
Reaction profiles for the recycling experiments in various HCOOH/solvent mixtures (0.016 mol % of **4**, 30 mol % of HCOONa at 80 °C).

The kinetic profiles of the reactions catalyzed by **4** in the presence of a solvent show a sigmoidal shape that suggests the presence of a catalyst preactivation pathway. This is supported by the fact that the induction period disappears upon catalyst recycling, thus resulting in a higher TOF_1h_ value in the second run in a 1/1 (v/v) DMC/HCOOH mixture. In a 4/1 (v/v) DMC/HCOOH mixture, the TOF_1h_ value increases from 1137 to 2271 h^–1^ from the first to the second run. The use of PC as a solvent leads to slightly lower catalytic activities in comparison to DMC in the first run (TOF_1h_ = 595 h^–1^), but remarkably, the catalyst shows better stability. Namely, in a 1/1 (v/v) PC/HCOOH mixture, the catalyst can be reused without an apparent loss of activity; in fact, an increase in the TOF_1h_ value is observed in the second run, which reaches a value of 934 h^–1^ (Figure 5). Overall, a TON value of over 13000 was achieved in the PC/HCOOH mixture without any sign of catalyst deactivation. In contrast with the good results obtained with **4** in DMC, under analogous conditions in a 1/1 (v:v) DMC/HCOOH mixture, the use of [Ir(dppp)(COD)]BF_4_ (dppp = 3-bis(diphenylphosphino)propane) as the catalyst, which essentially involves the substitution of the NHC moiety in **4** by a phosphane, brings about a dramatic drop in the catalytic activity. The value of TOF_1h_ for [Ir(dppp)(COD)]BF_4_ is 109 h^–1^, and the catalyst achieves a maximum TON value of 267 before deactivation, which highlights the key role of the NHC moiety.

The gas mixture obtained under optimized conditions (1/1 (v/v) DMC/HCOOH mixture, 30 mol % of HCOONa and 0.016 mol % of **4**) was analyzed by IR spectroscopy and GC-MS, showing no traces of CO (see the Supporting Information).

For the sake of comparison, the best-performing catalysts so far reported for the solventless dehydrogenation of formic acid are the Ir(III)-PC(sp^3^)P complex described by Gelman (TOF = 11760 h^–1^),^14b^ the [Ir(COD)(P-N)]OTf complex reported by Williams^23^ (TOF = 13320 h^–1^), the iridium(III) Cp*(dipyridylamine) complex described by Fischmeister (TOF = 5122 h^–1^),^14a^ the [Ir(COD)(PC^NHO^P)]BF_4_ reported by us^10b^ (TOF = 11590 h^–1^), and more recently the Ru(PNP) catalyst described by Milstein (TOF = 3067 h^–1^), which reaches TON values of over 1.7 million.^13b^

### 2.4 Mechanistic Studies on the Dehydrogenation of Formic Acid Catalyzed by Ir-PC^NHC^P and Ir-PC^NHC^O Complexes

#### 2.4.1 Reactivity of **3b** and **4** with HCOOH

With the intention of achieving a better understanding of the dehydrogenation mechanism, **3b** and **4** were reacted with an excess of HCOOH in the presence of 20 equiv of pyridine in an NMR tube, using CD_2_Cl_2_ as the solvent. In the case of **3b**, the reaction is sluggish, but small amounts (ca. 33% conversion) of a dihydride species can be observed after 48 h at 50 °C. The ^1^H NMR spectrum shows two doublets of triplets at δ −10.65 and −21.90 ppm (*J*_HH_ = 5.0 Hz and *J*_HP_ = 17.2 and 14.5 Hz, respectively), which are reminiscent of those for **5**. Note that the ^1^H{^31^P} NMR spectrum of **3b** shows two doublets at the same chemical shifts with the same H–H coupling constant. The resonance corresponding to the phosphorus nuclei of this dihydride complex appears as a singlet at δ 6.0 ppm in the ^31^P{^1^H} NMR. These data suggest the formation of a new complex with the formula [Ir(H)_2_(κ^2^-*P*,*C*,*P*′-PC^NHC^P)(py)]BF_4_, closely related to a complex recently reported by us, prepared by a similar procedure.^10a^ In addition, traces of a monohydride species are observed in the ^1^H NMR as a triplet at δ −20.03 ppm (*J*_HP_ = 12.8) and in the ^31^P{^1^H} NMR spectrum as a singlet at −5.6 ppm.

**Figure 6:**
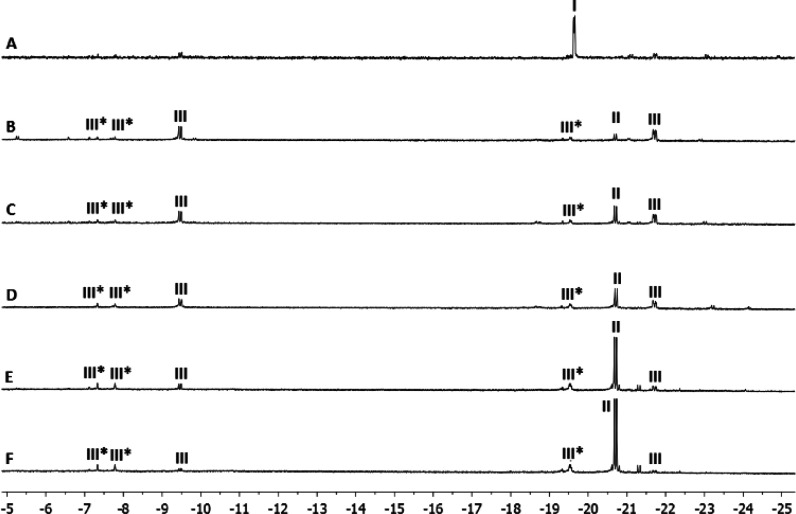
Evolution of the reaction of **4** with HCOOH and 20 equiv of pyridine.

The reaction of **4** with 4 equiv of HCOOH in the presence of 20 equiv of pyridine is remarkably faster than that of **3b**; in fact, gas evolution is immediately observed at room temperature. After 3 min, the ^31^P{^1^H} NMR spectrum shows no trace of **4** and a main peak appears at −12.4 ppm. This peak can be ascribed to the formation of a monohydride species (**I**) that displays a doublet at δ −19.64 ppm (*J*_HP_ = 7.5 Hz) in the ^1^H MNR spectrum (Figure 6A). After 4 h (see Figure 6B), a new monohydride (**II**) emerges as a doublet at δ −20.70 ppm (*J*_PH_ = 17.3 Hz) in the ^1^H NMR spectrum. At the same time, a dihydride species (**III**) appears as two doublets of doublets at δ −9.47 and −21.72 ppm (*J*_HH_ = 5.3 Hz and *J*_PH_ = 19.1 and 19.5 Hz, respectively), which suggests that both hydrides are *cis* to the phosphane moiety. It is noteworthy that traces of **III** are also observed in Figure 6A. Another 4 equiv of HCOOH was added to the reaction mixture, and the ^1^H NMR spectrum was measured after 3 min, which leads to an increase in the monohydride/dihydride ratio (Figure 6C). Four hours later, the monohydride becomes the major species in solution (Figure 6D). Subsequently, another 4 equiv of HCOOH was added and the temperature was increased to 45 °C, which further increases the monohydride/dihydride ratio (Figure 6E). In order to evaluate the stability of this species, an additional 4 equiv of HCOOH was added and the reaction mixture was left at 45 °C for 18 h (Figure 6F).

**Scheme 3:**
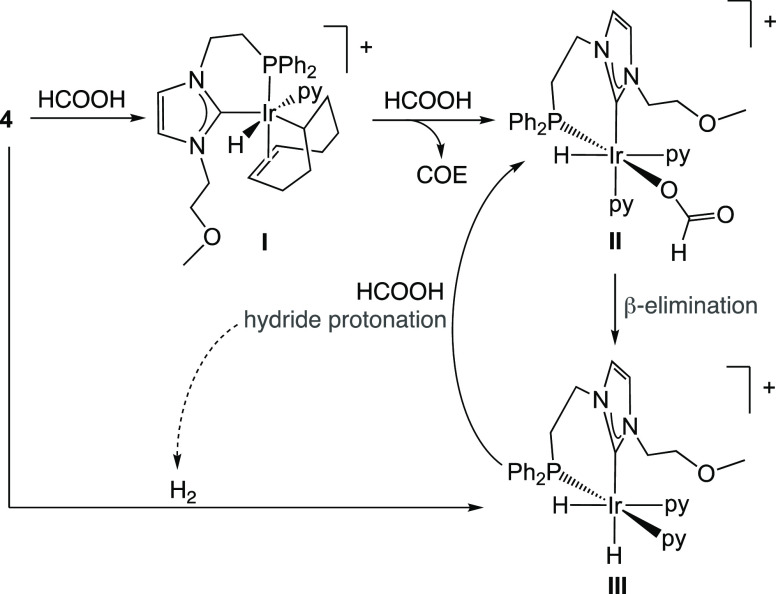
Postulated Species and Reactivity on the Basis of NMR Data

The first monohydride species, observed after 3 min at room temperature, is likely an intermediate in the hydrogenation of the COD ligand to COE (cyclooctene)—the formation of COE was confirmed by ^1^H NMR spectroscopy at this point. Possibly, as depicted in Scheme 3, **I** corresponds to **G-py**_**PCP-FA**_ (See section 8 of the Supporting Information and Figure S5), which is structurally related to intermediate species previously postulated in the literature, obtained upon reaction of [Ir(COD)(^t^Bu_2_PCH_2_(2-py))]CF_3_SO_3_ with HCOOH.^21^ Once COE is formed by reductive elimination of the alkyl and hydride ligands from **I**, the resulting Ir(I) complex is easily converted into monohydride **II** by oxidative addition of HCOOH. Subsequently, β-hydride abstraction affords the dihydride species, and protonation of one of the hydrides in **III** regenerates **II**.

The fact that **II** is the major species after long reaction times agrees with β-hydride elimination being the rate-determining step. **III** being the main species in solution at the initial reaction times of the reaction is due to the fact that **III** can also be formed from **4** (or **I**) in the presence of H_2_, which is generated by the dehydrogenation of HCOOH after the first turnover of the catalyst. Note that gas evolution is visible from the start of the reaction. In this regard, when **4** is placed under a H_2_ atmosphere with 20 equiv of pyridine in a Young NMR tube using CD_2_Cl_2_ as the solvent, in the absence of HCOOH, only species **I** and **III** are observed at the early stages.

After prolonged reaction times, **III** becomes the only species in solution, with no traces of **II** being detected throughout the reaction. Therefore, when no complex **4** (or **I**) remains in solution, the only source of **III** is the sluggish β-hydride elimination from **II**, whereas, **III** converts rapidly into **II** by protonation with HCOOH. Small amounts of an unidentified species (III*) are also observed in Figure 6.

At this point, it is worth mentioning that the same stoichiometric experiments were also performed in the presence of formate (30 mol %), providing results identical with those discussed above. This behavior suggests that the presence of formate is not required for the activation of the precatalyst. However, formate improves the catalytic performance of **4**, which probably has to do with the optimization of the acidity of the reaction media. In fact, we have observed that the use of formate concentrations higher than 30 mol % leads to a decrease in the catalytic activity.

**Figure 7:**
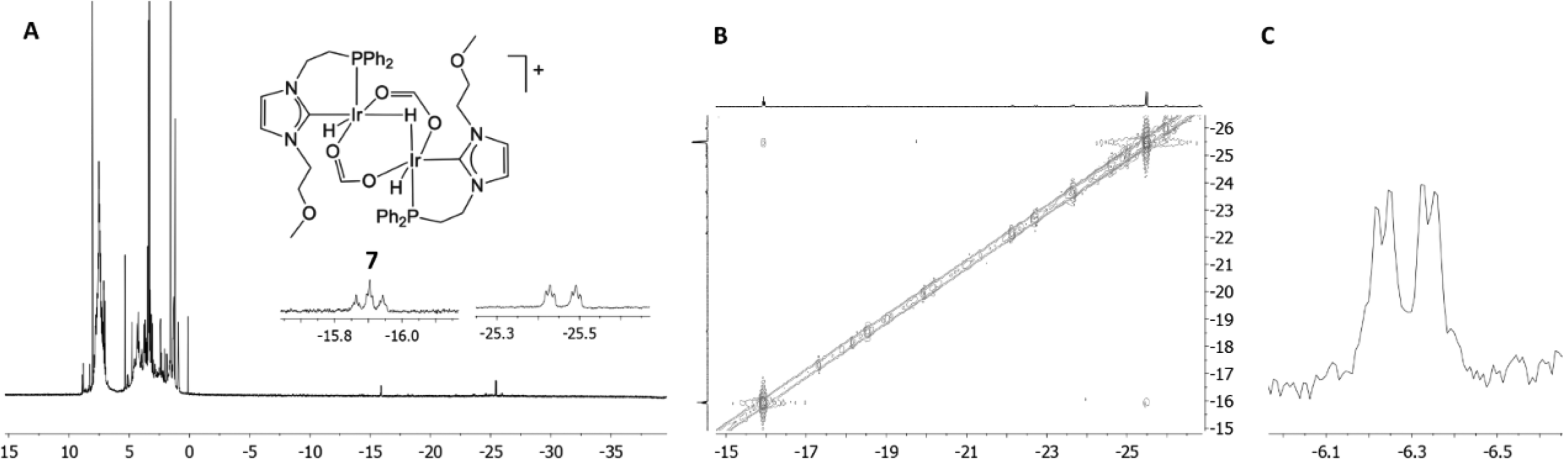
Depiction of the postulated dinuclear species **7** and ^1^H NMR (A), ^1^H–^1^H COSY NMR (B), and ^31^P NMR (C) spectra.

A new experiment was carried out in the absence of pyridine. Without the stabilizing effect of py, the reaction of **4** with excess HCOOH in CD_2_Cl_2_ shows the formation of a new hydride-containing species in solution. A plausible molecular structure would entail a cationic dinuclear complex with one bridging hydride and two terminal hydrides, with the coordination sphere of the Ir centers being completed with two bridging formates. A tentative structure supported by the NMR data discussed below is depicted in Figure 7.

In the ^1^H NMR spectrum (Figure 7A), two main resonances emerge in the high-field region, an apparent triplet of triplets at δ −15.91 ppm and an apparent doublet of triplets at δ −25.47 ppm with a 1:2 integration ratio. The multiplicity of the former is due to the coupling of the bridging hydride with the two terminal hydrides, which are likely chemically inequivalent, but the doublet of doublets expected for this system collapses to an apparent triplet due to the similar chemical shifts of both hydrides. Coupling with the two P nuclei eventually gives the apparent triplet of triplets observed in the ^1^H NMR spectrum. In the case of the latter, each terminal hydride couples with the bridging hydride to give two doublets, which appear as a triplet because of the similar chemical shifts of both hydrides. Coupling with each vicinal P nucleus gives an apparent doublet of triplets. This postulation is supported by the ^1^H{^31^P} NMR spectrum, which presents two apparent triplets at δ −15.91 and −25.47 ppm, and by the ^1^H–^1^H COSY NMR spectrum (Figure 7B), which features a correlation between both hydride resonances. The ^31^P NMR spectrum shows two very close doublets at δ −6.23 and −6.34 ppm (*J*_PP_ = 4.7 Hz), which confirms that the P nuclei have slightly different chemical environments (Figure 7C).

**Figure 8:**
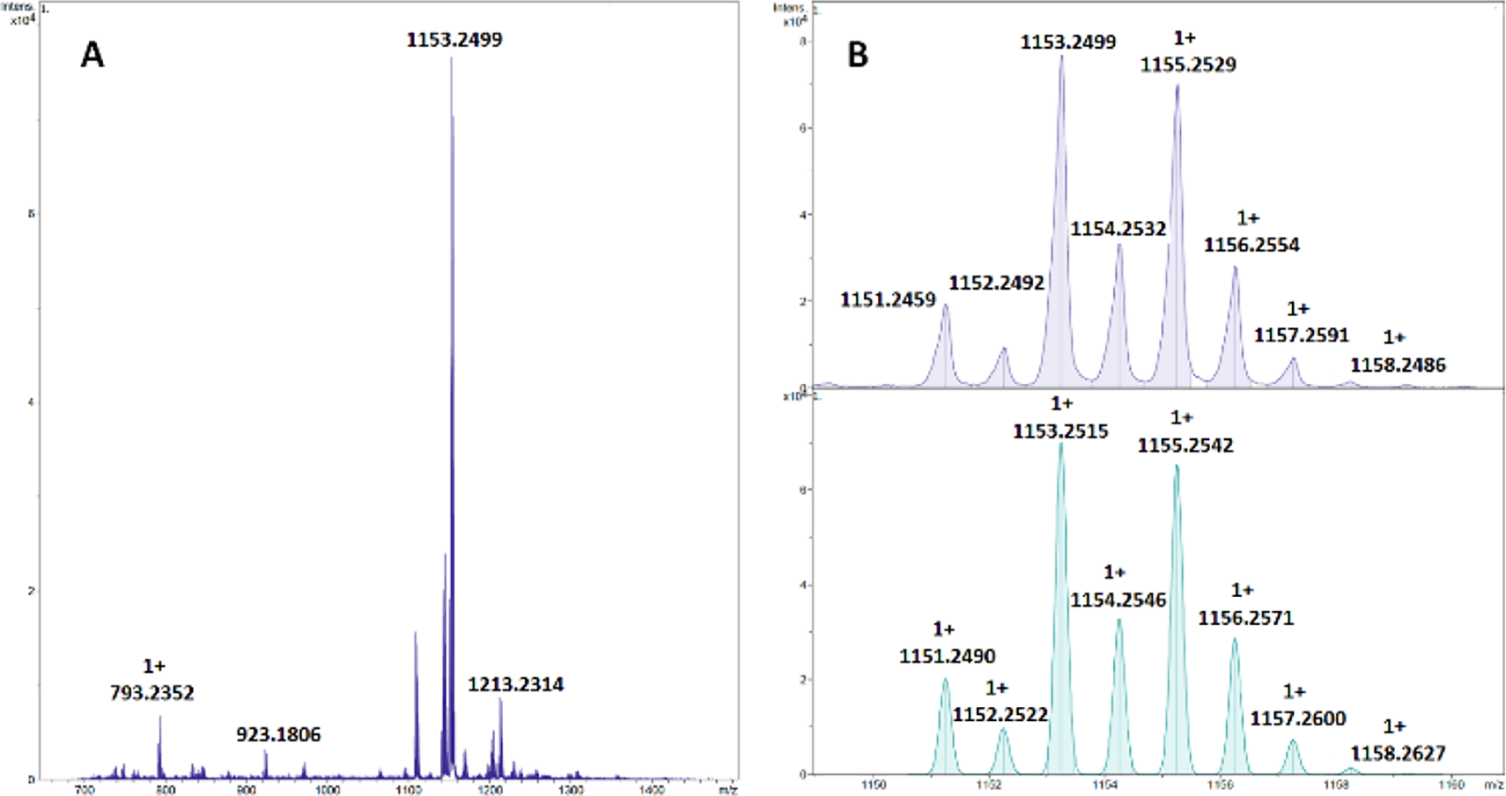
HR-MS (A) and isotopic distribution of the observed (B, top) and simulated (B, bottom) molecular ion peak (M^+^).

The presence of the proposed dinuclear species was corroborated by high-resolution electrospray ionization mass spectrometry (HR-ESI-MS), which shows a main peak of *m*/*z* 1153.2499 (Figure 8).

Further support for the dinuclear nature of **7** in solution was obtained by means of ^1^H DOSY NMR spectroscopy. The hydrodynamic radii of **7** and **4** were calculated by applying a modified Stokes–Einstein equation.^24^ The resonances of the hydride at δ −25.47 ppm for **7** and the methoxy group at δ 3.29 ppm for **4** were employed for the determination of the diffusion coefficients (*D*) in CD_2_Cl_2_ at 300 K. The *D* value calculated for **7** was significantly smaller than that calculated for **4**, 6.951 × 10^–10^ and 1.033 × 10^–9^ m^2^ s^–1^, respectively, which is in agreement with **7** being a larger molecule. In fact, we obtained values of 7.7 and 5.1 Å for the hydrodynamic radii (*r*_H_) of **7** and **4**, respectively. It is worth mentioning that the *r*_H_ value of **4** agrees well with the value of 5.5 Å estimated by single-crystal X-ray diffraction analysis.

The proposed structure would be similar to those reported by Williams^13a,23^ and Inagaki.^25^ In the case of the latter, this species was postulated as a resting state that can be transformed into an intermediate of the catalytic cycle upon UV irradiation. On these grounds, it is plausible that, in the absence of a coordinating solvent, **4** deactivates via formation of a dinuclear species, which may be inactive or, simply, less active than the mononuclear species. Moreover, other deactivation pathways that could derive from the formation of **7**, such as the generation of Ir(II) dinuclear complexes^26^ or polyhydrido clusters, may also be conceivable.^27^ This decomposition pathway also agrees with the fact that a 4/1 (v/v) DMC/HCOOH mixture allows higher TON numbers in comparison to the related 1/1 mixture, because a higher DMC/HCOOH ratio further prevents the formation of dinuclear species. In this regard, addition of 10 equiv of pyridine to a solution of **7** in CD_2_Cl_2_ with excess HCOOH does not generate species **II** or **III** at room temperature; however, prolonged heating (24 h) at 50 °C affords the monohydride species **II**. Therefore, it is plausible that the use of coordinating solvents prevents the formation of **7** (and further evolution to other inactive species) or allows **7** to re-enter the catalytic cycle.

In summary, the stoichiometric reactions in the presence of pyridine simulate the use of a coordinating solvent, such as DMC, which prevents the formation of the dinuclear species. On the other hand, in the absence of pyridine, the reaction conditions are more similar to those in neat FA, because the vacant coordination sites can be only occupied by HCOOH or formate, which leads to formation of the dinuclear species.

#### 2.4.2 DFT Studies

In order to shed light on the reaction mechanism that operates in the dehydrogenation of formic acid using precatalysts **3b** and **4**, a computational study at the DFT level was performed (see the Experimental Section in the Supporting Information for a further explanation of the computational details). We computed a feasible reaction mechanism for the reaction pathways based on **3b** in formic acid, as well as for **4**-based pathways in both formic acid and dimethylcarbonate. Note that, for the sake of clarity, the reaction intermediates and transition structures based on **3b** are referred to with the subscript *PCP*, and start with the letter **A**, which corresponds to **3b**, i.e., **A**_***PCP***_. For structures based on **4**, we also start with the letter **A** and use the subscripts *PCO-FA* and *PCO-DMC* for the reactions in formic acid and dimethyl carbonate, respectively.

**Figure 9:**
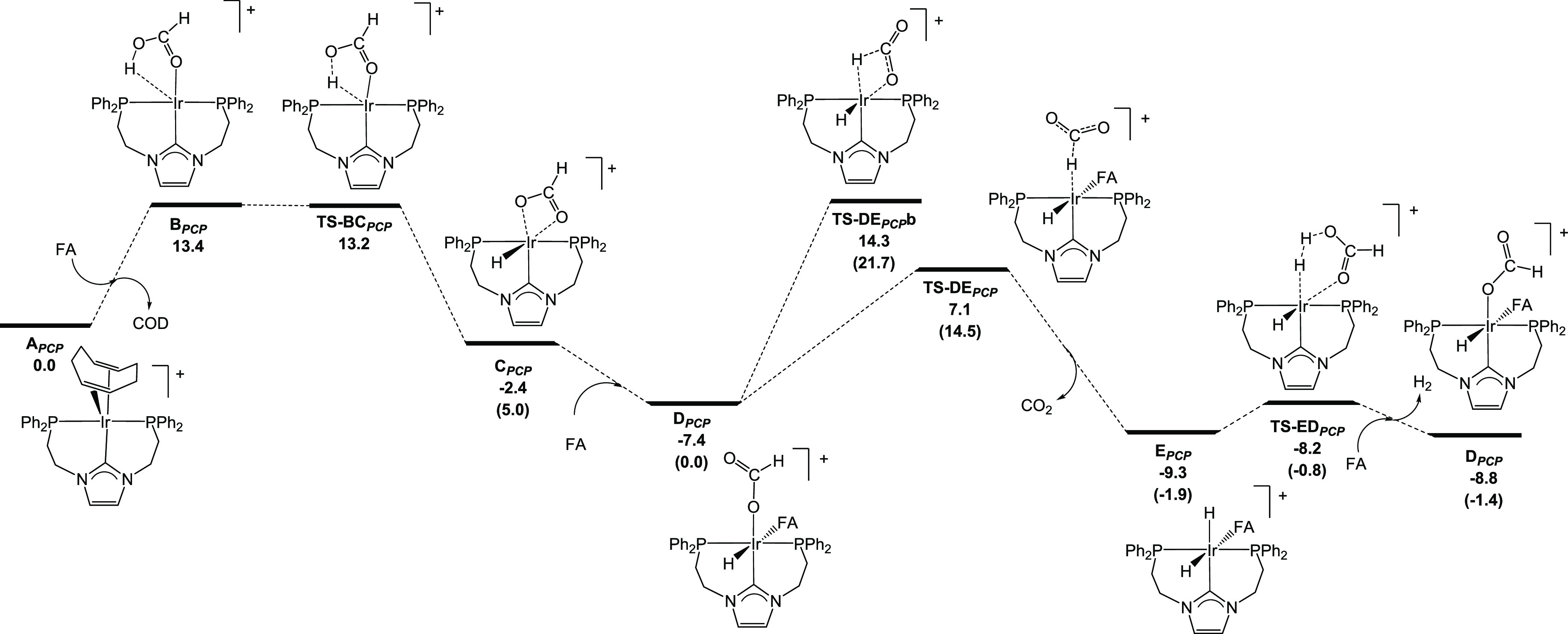
DFT-calculated Gibbs free energy profile (in kcal mol^–1^) for the solventless dehydrogenation of HCOOH (FA) catalyzed by **3b** (**A**_***PCP***_). Note that the values in parentheses correspond to energy values with respect to **D**_***PCP***_ (the catalytically active species).

In the case of precatalyst **3b** (**A**_***PCP***_ in Figure 9), the preactivation step would involve the direct COD dissociation to afford **B**_***PCP***_, which is 13.4 kcal mol^–1^ higher in energy than **A**_***PCP***_ and, thus, feasible under the reaction conditions (80 °C). This step would be followed by oxidative addition of the O–H moiety of the coordinated molecule of formic acid, which gives the monohydride species **C**_***PCP***_, with a relative Gibbs energy of −2.4 kcal mol^–1^ with respect to **A**_***PCP***_: that is, 15.8 kcal mol^–1^ more stable than **B**_***PCP***_. This process takes place via **TS-BC**_***PCP***_, with a relative Gibbs energy of 13.2 kcal mol^–1^, which is 0.2 kcal mol^–1^ lower than that of **B**_***PCP***_. Note that, although this value may seem meaningless, it is a consequence of the corrections included to obtain the Gibbs energy (*G*) from the electronic energy, which is 1.2 kcal mol^–1^ higher for **TS-BC**_***PCP***_ (see Table S1).

**C**_***PCP***_ can be stabilized by coordinating a HCOOH molecule, leading to **D**_***PCP***_, in which the formate ligand has changed its coordination mode from κ^2^ to κ^1^. This monohydride intermediate, with a Gibbs energy of −7.4 kcal mol^–1^ relative to **A**_***PCP***_, that is, 5.0 kcal mol^–1^ more stable than **C**_***PCP***_, constitutes the active species of the catalytic cycle. In order to facilitate an understanding of the text and reaction mechanism diagram (see Figure 9), we also indicate the relative energy with respect to **D**_***PCP***_ in parentheses. This species undergoes hydride abstraction via **TS-DE**_***PCP***_ (which is 14.5 kcal mol^–1^ higher in Gibbs energy than **D**_***PCP***_) to yield the dihydride intermediate **E**_***PCP***_ and a molecule of CO_2_. At this point we also considered the β-hydride elimination instead of the hydride abstraction process (**TS-DE**_***PCP***_**b**), which turned out to be 7.2 kcal mol^–1^ higher in Gibbs energy for the considered level of theory and was therefore discarded. Finally, protonation of one of the hydrides by the formic acid coordinated to the Ir center affords H_2_ and regenerates the monohydride species. This step takes place via **TS-ED**_***PCP***_, which is only 0.9 kcal mol^–1^ higher in Gibbs energy than **F**_***PCP***_.

According to the framework proposed by Kozuch and Shaik, the overall energy span for the catalytic cycle is 15.0 kcal mol^–1^ and corresponds to the hydrogen abstraction transition process. Namely, this value is dictated by the energy difference between **TS-DE**_***PCP***_ and **E**_***PCP***_ (16.4 kcal mol^–1^) and, as the TOF-determining intermediate appears after the transition structure, we would need to add the cycle Δ*G* (−1.4 kcal mol^–1^), yielding the aforementioned value of 15.0 kcal mol^–1^. Note that this value is very close to the energy difference between **D**_**PCP**_ and **TS-DE**_***PCP***_ (14.5 kcal mol^–1^), but within the aforementioned framework, the energy span corresponds to the highest effective barrier.^28^

**Figure 10:**
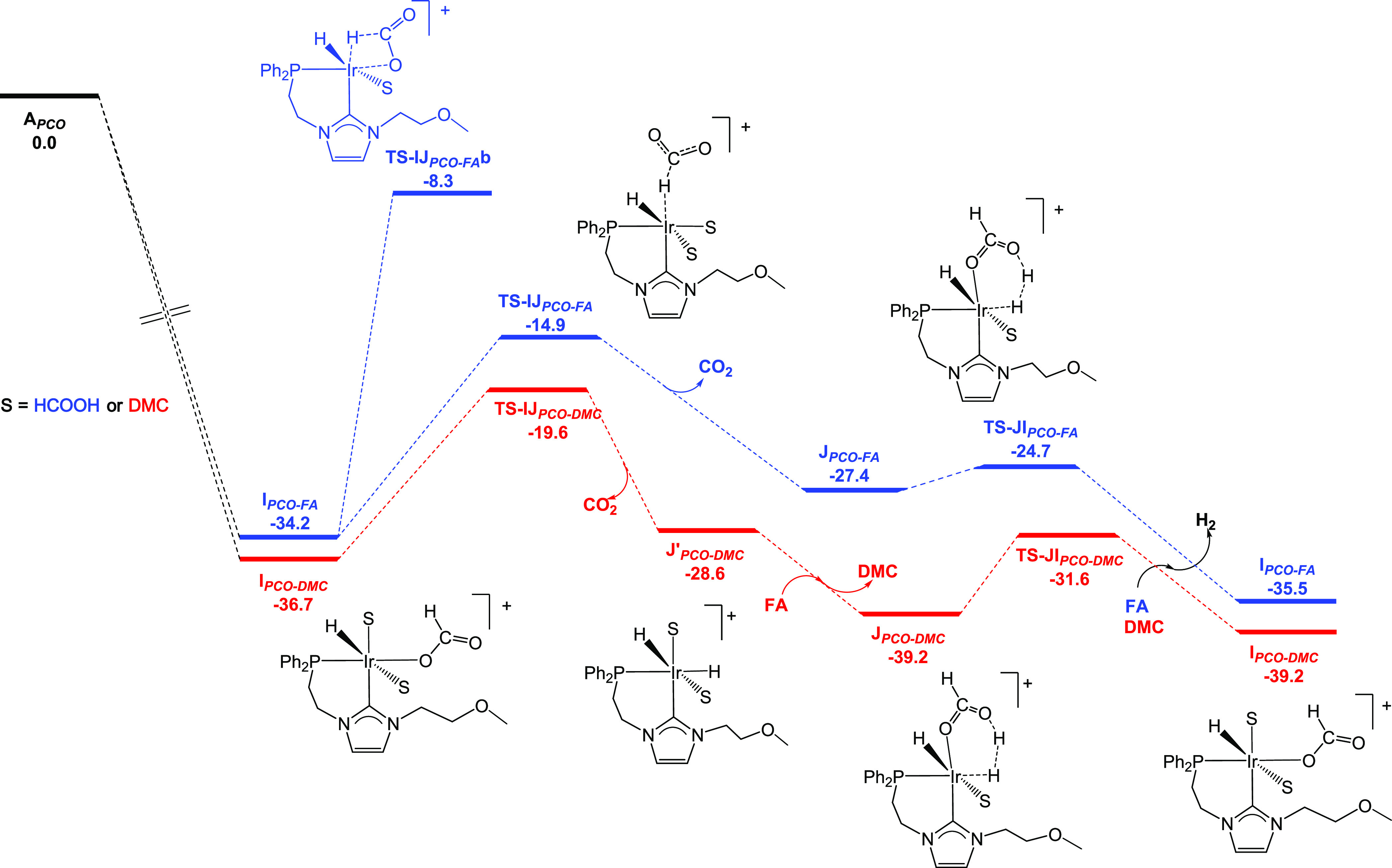
DFT-calculated Gibbs free energy profile (in kcal mol^–1^) for the solventless dehydrogenation of FA catalyzed by **4**. Note that, for the reaction in DMC (red profile), a DMC ligand needs to exchange with a formic acid molecule in **J′**_***PCO-DMC***_, leading to **J**_***PCO-DMC***_, in order to render the hydride protonation step (**TS-IJ**_***PCO-DMC***_).

In contrast with **3b**, **4** (**A**_***PCO-FA***_ in Figure S5) undergoes an activation process that results in the formation of the active species, the monohydride **I**_***PCO-FA***_ (related to species **II** in Scheme 3), by the hydrogenation of the COD ligand to COE (see the Supporting Information). The catalytic cycle involving the active species for the reaction in formic acid is depicted in blue in Figure 10. This species undergoes a hydride abstraction process, via **TS-IJ**_***PCO-FA***_, which is 19.3 kcal mol^–1^ higher in Gibbs energy than **I**_***PCO-FA***_. As a result, dihydride **J**_**PCO-FA**_ is produced, which is related to **III** and III* (Scheme 3). As for the **3b**-related catalytic cycle, we compared this transition structure with that resulting from the β-hydride elimination process (**TS-IJ**_***PCO-FA***_**b**; see Figure 10), which is 6.6 kcal mol^–1^ higher in Gibbs energy. In this way, we propose that CO_2_ release proceeds through a hydride abstraction. Subsequently, protonation of the hydride ligand occurs by **TS-JI**_***PCO-FA***_, thus producing a molecule of H_2_ alongside the regeneration of **I**_***PCO-FA***_ (Figure 10). The overall energy barrier of the process is 19.3 kcal mol^–1^, i.e., the Gibbs energy difference between **TS-IJ**_***PCO-F*****A**_ and **I**_***PCO-FA***_, which is 4.3 kcal mol^–1^ higher than that calculated for **3b** and may contribute to the comparatively lower activity of **4** under solventless conditions.

In order to evaluate the influence of the use of DMC as the solvent, the catalytic cycle was studied by considering DMC instead of formic acid coordination. This results in a drop in the overall activation energy of the process from 19.3 to 17.1 kcal mol^–1^. However, this difference may not justify the drastic activity difference, which is likely due to an improved catalyst stability. It is noteworthy that the coordination of DMC brings about a notable stabilization of the reaction intermediates, which plausibly inhibits the formation of dinuclear species. Complexes analogous to **7** have been described to form under HCOOH dehydrogenation conditions. The reaction pathway entails two steps: (i) dimerization of the dihydride complex (in this case a species related to **III** or **M**) to render a dinuclear complex with two terminal and two bridging hydrides, followed by (ii) protonation of one of the hydrides with HCOOH to give a complex with two terminal hydrides and one bridging hydride (in this case **7**).^13a,23^

It is worth noting that the DFT calculations show that HCOOH and DMC coordinate more strongly in comparison to the ether wingtip group at the PC^NHC^O ligand, thus discarding the initially postulated hemilabile behavior.

#### 2.4.3 KIE Measurements

In order to assess the postulation that the hydride abstraction is the rate-determining step, H/D kinetic isotope effect (KIE) experiments were carried out using **4** as the catalyst (Figure S3). The initial TOF_1h_ value was virtually the same in HCOOH and HCOOD (887 and 877 h^–1^, respectively). However, a drop in the TOF_1h_ value to 517 h^–1^ was observed upon replacing the HCOOH/HCOONa mixture by DCOOH/DCOONa. The KIE of 1.7 obtained upon changing HCOOH/HCOONa to DCOOH/DCOONa strongly suggests that the rate-determining step entails the C–H bond cleavage of the formate ligand, which occurs in the hydride abstraction step.

#### 4.2.4 Calculation of the Activation Energy

**Figure 11:**
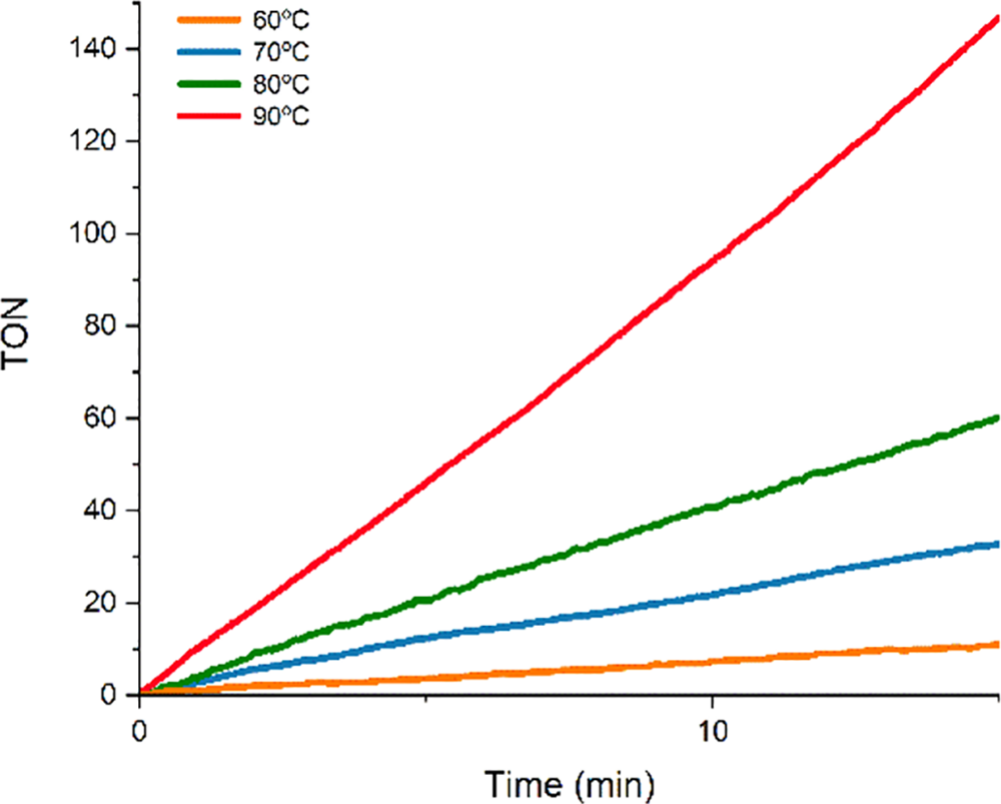
Reaction profiles for the dehydrogenation of FA in the temperature range 60–90 °C (DMC/HCOOH (1/1), 30 mol % of HCOONa, and 0.016 mol % of **4**).

**Figure 12:**
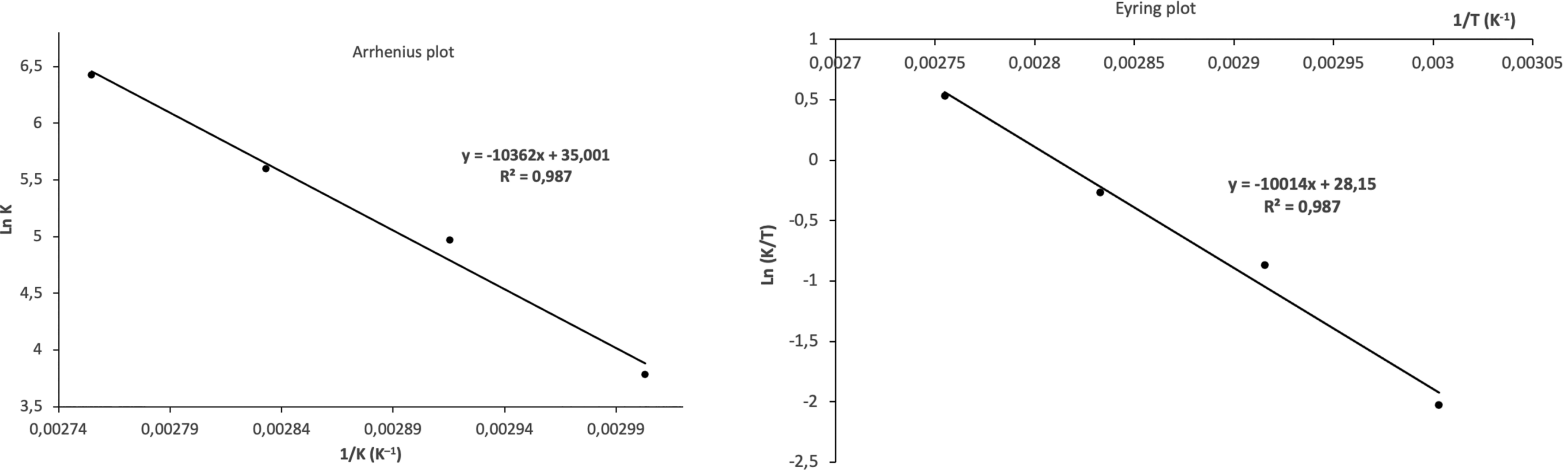
Arrhenius and Eyring plots for the dehydrogenation of FA using complex **4** as the catalyst.

The activation energy (*E*_a_) for the dehydrogenation of HCOOH in a 1/1 (v/v) DMC/HCOOH mixture using **4** as catalyst was estimated experimentally using the reaction rates measured at 60, 70, 80, and 90 °C (Figure 11). An *E*_a_ value of 21.4 ± 1.6 kcal mol^–1^ was calculated from the slope (−*E*_a_/*R*) of the Arrhenius plot (Figure 12). In addition, the Eyring model renders a Δ*H*^⧧^ value of 19.9 ± 1.6 kcal mol^–1^ and a Δ*S*^⧧^ value of 8.7 ± 3.9 cal K^–1^ mol^–1^, which results in a Δ*G*^⧧^ value of 17.3 ± 1.6 kcal mol^–1^ at 298 K. These experimental values compare well with that obtained by DFT calculations (17.1 kcal mol^–1^), especially the Δ*G*^⧧^ value calculated employing the Eyring model.

## 3 Conclusions

We have prepared two iridium catalysts, **3b** and **4**, that are based on a PC^NHC^P and a PC^NHC^O ligand, respectively. Both were active under solventless conditions, but a significant activity boost was observed in the case of **4** when organic carbonates were employed as solvent. This enhanced performance in DMC or PC can be ascribed to the stabilization of the catalyst, which precludes the deactivation via the formation of a dinuclear species. Remarkably, the improved activities cannot be attributed to a better solubility of the catalyst or sodium formate, since both are soluble in HCOOH but insoluble in DMC.

Key putative intermediates of the catalytic cycle, stabilized in the presence of excess pyridine, were identified in solution by the reaction of **4** with HCOOH. At long reaction times, the monohydride **II** was the main species in solution, which suggests that the hydride abstraction to yield **III** is the rate-determining step. KIE measurements further support this postulation, as an activity drop occurs when HCOOH is substituted by DCOOH, while the activity remains the same in the case of HCOOD.

The reaction mechanism was substantiated by DFT calculations. As suggested by the catalytic and stoichiometric experiments, in a 1/1 DMC/HCOOH mixture (v/v), using **4** as the catalyst, a preactivation step that involves the hydrogenation of COD to COE leads to the formation of an unsaturated Ir(I) species that undergoes oxidative addition of HCOOH. The resulting monohydride species turns into a dihydride species by hydride abstraction from the formate ligand (the rate-limiting step), with concomitant formation of CO_2_. Finally, protonation of the dihydride with HCOOH leads to the formation of H_2_ and the regeneration of the monohydride (the active species).

In summary, this work shows that the use of relatively small amounts of a “green” cosolvent, such as an organic carbonate, allows a remarkable enhancement of the catalytic performance. This behavior can be attributed to a stabilization of the active species, which prevents the deactivation of the catalyst via the formation of dinuclear species.

## Supporting Information Available

Experimental data and DFT calculation details (PDF)

## Accession Codes

CCDC 2068679 contains the supplementary crystallographic data for this paper. These data can be obtained free of charge via www.ccdc.cam.ac.uk/data_request/cif, or by emailing data_request@ccdc.cam.ac.uk, or by contacting The Cambridge Crystallographic Data Centre, 12 Union Road, Cambridge CB2 1EZ, UK; fax: +44 1223 336033.
