# Supplementary material for: Impact of Green Cosolvents on the Catalytic Dehydrogenation of Formic Acid: The Case of Iridium Catalysts Bearing NHC-phosphane Ligands
Source: Inorg Chem. 2021 Sep 24;60(20):15497–508. doi: 10.1021/acs.inorgchem.1c02132 (PMC8527458; doi:10.1021/acs.inorgchem.1c02132)
Supplement: Supplementary file 1 — ic1c02132_si_001.pdf [file ic1c02132_si_001.pdf]

Supporting Information for

Impact of green cosolvents on the catalytic dehydrogenation of formic acid: The case of iridium catalysts bearing NHC-phosphane ligands

Ana Luque-Gómez,[a] Susana García-Abellán,[a] Julen Munarriz,[b] Victor Polo,[c] Vincenzo Passarelli[a] and Manuel Iglesias[a]*

[a] Departamento Química Inorgánica–Instituto Síntesis Química y Catálisis Homogénea (ISQCH), Universidad de Zaragoza–CSIC, C/Pedro Cerbuna 12,50009 Zaragoza, Spain. E-mail: miglesia@unizar.es.

[b] Departamento Química Física y Analítica, Universidad de Oviedo, Avda. Julian Clavería 8, 33006, Oviedo, Spain.

[c] Departamento Química Física–Instituto de Biocomputación y Física de SistemasComplejos (BIFI), Universidad de Zaragoza, Pedro Cerbuna 12, 50009 Zaragoza, Spain.

Table of Contents:

**1. Experimental details**	**S2**
**2. NMR Spectra**	**S7**
**3. IR spectrum of the mixture of gases obtained from catalysis**	**S18**
**4. Gas Chromatography of the mixture of gases obtained from catalysis**	**S19**
**5. Kinetic profiles KIE**	**S20**
**6. Kinetic profiles at early stages**	**S21**
**7. Computational Details**	**S22**
**8. Mechanism for the activation of 4**	**S23**
**9. DOSY NMR Experiments**	**S27**
**10. Crystal structure determination of 4**	**S29**
**11. DFT energy data and optimized geometries**	**S30**

12. References S101

1. Experimental details

General:

All experiments were carried out under an inert atmosphere using standard Schlenk techniques. The solvents were dried by known procedures and distilled under argon prior to use or obtained oxygen and water-free from a Solvent Purification System (Innovative Technologies). All other commercially available starting materials were purchased from Sigma-Aldrich, Merck and J. T. Baker and were used without further purification. ^{1}H, $^{13}C\{^{1}H\}$, $^{31}P\{^{1}H\}$ and ^{19}F spectra were recorded either on a Bruker ARX 300 MHz or a Bruker Avance 400 MHz instruments. Chemical shifts (expressed in parts per million) are referenced to residual solvent peaks (^{1}H, $^{13}C\{^{1}H\}$). Coupling constants, J, are given in Hz. Spectral assignments were achieved by combination of ^{1}H-^{1}H COSY, ^{13}C APT and 1H-^{13}C HSQC/HMBC experiments. GC-MS spectra were recorded on a Hewlett-Packard GC-MS system. All the catalytic reactions were performed in a Man on the Moon series X102 kit micro-reactor with a total volume of 19 mL. Formic acid and water were degassed prior to use.

Synthesis of imidazolium salt 2

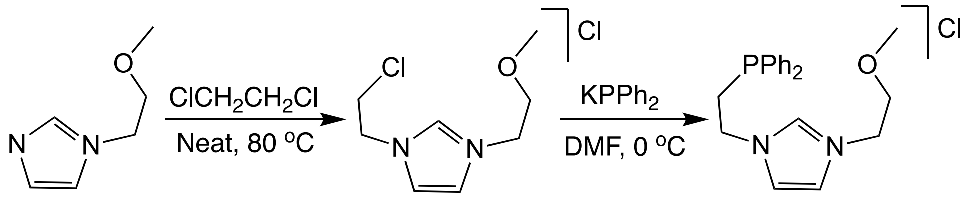

Scheme S1. Synthetic pathway for the preparation of **2**.

Synthesis of ClCO-Cl.

1-(2-methoxyethyl)imidazole (600 mg, 4.6 mmol) was dissolved in 20 mL of 1,2-dichloroethane and stirred at 80 °C for 48 h. Subsequently, the solvent was evaporated under reduced pressure and the residue was redissolved in the minimum amount of CH_2Cl_2 and precipitated with Et_2O. The dense oil thus obtained was washed with Et_2O (5 x 15 mL) to afford the title compound as a white waxy solid in 85 % yield (877 mg, 3.9 mmol). ^{1}H NMR (CD_2Cl_2, 400 MHz): δ 10.11 (s, 1H, NC*H*N), 8.01 (t, 1H, CH_{im}), 7.72 (t, 1H, CH_{im}), 4.74 (t, 2H, NCH_2^{Cl}), 4.47 (t, 2H, NCH_2^{OMe}), 4.05 (t, 2H, ClCH_2), 3.66 (t, 2H, OCH_2), 3.21 (s, 3H, OCH_3). $^{13}C\{^{1}H\}$ NMR APT, (CD_2Cl_2, 100 MHz): δ 138.0 (s, NCHN), 123.3 (s,

CH_{Im}), 123.2 (s, CH_{Im}), 70.5 (s, CH_2O), 59.1 (s, CH_3O), 51.5 (s, NCH_2^{Cl}), 50.0 (s, NCH_2^{OMe}) 43.6 (s, CH_2Cl). HRMS (ESI) m/z Calcd. for $C_8H_{14}ClN_2O$ (M–BF_4) 189.0789, found 189.0793.

Synthesis of PCO-Cl. ^tBuOK (386 mg, 3.45 mmol) was added as a solid over a solution of $HPPh_2$ (0.5 mL, 2.87 mmol) in 7 mL of DMF at 0 °C. The resulting orange solution was stirred for 1 h, after which time the solution of $KPPh_2$ was cannulated into a solution of **ClCO-Cl** (607 mg, 3.45 mmol) in 7 mL of DMF at 0 °C and stirred for ca. 18 h at room temperature (the reaction was monitored by ^{31}P NMR). Subsequently, the solvent is evaporated under reduced pressure at 60 °C. The residue was redissolved in dichloromethane (10 mL) and any precipitate filtered off. The thus obtained solution was evaporated under reduced pressure, washed with diethyl ether (3 x 10 mL) and dried under vacuum to give the title complex as a light brown oil in a 71 % yield (760 mg, 2.03 mmol). ^1H NMR (CD_2Cl_2, 400 MHz): δ 10.46 (s, 1H, NC*H*N), 7.45-7.42 (m, 1H, CH_{im}), 7.41-7.39 (m, 1H, CH_{im}), 7.39-7.34 (m, 4H, CH_{Ar}), 7.26-7.21 (m, 6H, CH_{Ar}), 4.41-4.35 (m, 2H, NCH_2^{OMe}), 4.34-4.26 (m, 2H, NCH_2^{PPh2}), 3.63-3.58 (m, 2H, OCH_2), 3.21 (s, 3H, OCH_3), 2.73-2.65 (m, 2H, PCH_2). $^{13}C\{^1H\}$ NMR APT, (CD_2Cl_2, 100 MHz): δ 137.4 (s, N*C*HN), 137.0 (d, $^1J_{C-P}$ = 14.0, $C_{ipso}P$), 133.2 (d, $^3J_{C-P}$ = 19.5, CH_{orto}), 129.6 (s, $C_{Ar-para}$), 129.2 (d, $^4J_{C-P}$ = 19.5, CH_{meta}), 123.3 (s, CH_{Im}), 122.2 (s, CH_{Im}), 70.6 (s, CH_2O), 59.1 (s, CH_3O), 49.9 (s, NCH_2^{OMe}), 47.9 (d, $^3J_{CP}$ = 23.3, NCH_2^{PPh2}) 29.2 (d, $^1J_{CP}$ = 16.1, CH_2P). $^{31}P\{^1H\}$ NMR (CD_2Cl_2, 162 MHz): δ –21.7 (s, PPh_2). HRMS (ESI) m/z Calcd. for $C_{20}H_{24}N_2OP$ (M–BF_4) 339.1621, found 339.1612.

Synthesis of complexes 3-5

Syhnthesis of 3a. Imidazolium salt **1** (50 mg, 0.095 mmol) and $[Ir(\mu\text{-}OCH_3)(COD)]_2$ (31 mg, 0.047 mmol) were dissolved in CH_2Cl_2 (10 mL) and the resulting solution stirred at room temperature for 30 minutes. Subsequently, the solvent is evaporated under reduced pressure and the residue thus obtained is washed with pentane (3 x 10 mL) and dried under vacuum. This yields the title complex as an off-white solid in a 72 % yield (57 mg, 0.068 mmol). ^1H NMR (CD_2Cl_2, 300 MHz): δ 7.61-7.45 (m, 10H, CH_{Ar}), 7.43 (s, 2H, CH_{Im}), 7.32-7.23 (m, 2H, CH_{Ar}), 7.16-7.08 (m, 2H, CH_{Ar}), 7.06-6.98 (m, 3H, CH_{Ar}), 6.85-6.74 (m, 3*H*, CH_{Ar}), 4.88-4.66 (m, 4H, CH_2N), 4.17 (bs, 2H, CH_{COD}), 3.51-3.34 (m, 2H, CH_2P), 2.72 (bs, 2H, CH_{COD}), 2.62-2.47 (m, 2H, CH_2P), 2.43-2.24 (m, 6H, $CH_{2\text{-}COD}$), 2.11-1.94 (m,

2H, $CH_{2\text{-COD}}$). $^{13}C\{^{1}H\}$ NMR APT (CD_2Cl_2, 100 MHz): δ 143.0 (t, $^{2}J_{C\text{-}P}$ = 14.2, N*C*N), 137.4 (dd, $^{1}J_{C\text{-}P}$ = 42.6, $^{3}J_{C\text{-}P}$ = 2.6, C_{ipso}P), 134.8 (dd, $^{1}J_{C\text{-}P}$ = 43.5, $^{3}J_{C\text{-}P}$ = 5.9, C_{ipso}P), 132.0 (t, $^{4}J_{C\text{-}P}$ = 5.2, $C_{Ar\text{-}meta}$), 131.1 (t, $^{4}J_{C\text{-}P}$ = 4.6, $C_{Ar\text{-}meta}$), 129.8 (s, $C_{Ar\text{-}para}$), 129.7 (s, $C_{Ar\text{-}para}$), 128.6 (t, $^{3}J_{C\text{-}P}$ = 4.8, $C_{Ar\text{-}orto}$), 128.1 (t, $^{3}J_{C\text{-}P}$ = 4.7, $C_{Ar\text{-}orto}$), 123.1 (s, CH_{Im}), 86.2 (bs, CH_{COD}), 52.0 (bs, CH_{COD}), 48.7 (s, CH_2N), 34.3 (bs, $CH_{2\text{-COD}}$), 30.3 (bs, CH_{2COD}), 30.0-29.6 (m, CH_2P). $^{31}P\{^{1}H\}$ NMR (CD_2Cl_2, 162 MHz): δ –21.0 (s, PPh_2). HRMS (ESI) m/z Calcd. for $C_{39}H_{42}IrN_2P_2$ (M–BF_4) 793.2449, found 793.2417.

Synthesis of 3b. $AgBF_4$ (18 mg, 0.095 mmol) was added as a solid over a solution of complex **3a** (40 mg, 0.047 mmol) in CH_2Cl_2 (10 mL). The resulting suspension was stirred for 2 h in the dark. The resulting white precipitate was filtered off, and the thus obtained solution evaporated under reduced pressure. The residue was washed with pentane (3 x 10 mL) and dried under vacuum to give the title complex as an off-white solid in a 92 % yield, (79 mg, 0.087 mmol). ^{1}H NMR (CD_2Cl_2, 400 MHz): δ 7.65-7.34 (m, 10H, CH_{Ar}), 7.18-7.09 (m, 2H, CH_{Ar}), 7.11 (s, 2H, CH_{Im}), 7.07-6.98 (m, 4H, CH_{Ar}), 6.85-6.74 (m, 4H, CH_{Ar}), 4.92-4.68 (m, 2H, CH_2N), 4.65-4.41 (m, 2H, CH_2N), 4.18 (bs, 2H, CH_{COD}), 3.52-3.34 (m, 2H, CH_2P), 2.73 (bs, 2H, CH_{COD}), 2.65-2.48 (m, 2H, CH_2P), 2.35 (bs, 2H, $CH_{2\text{-COD}}$), 2.03 (bs, 2H, $CH_{2\text{-COD}}$), 1.72-1.21 (m, 4H, $CH_{2\text{-COD}}$). $^{13}C\{^{1}H\}$ NMR APT (CD_2Cl_2, 100 MHz): δ 143.6 (t, $^{2}J_{C\text{-}P}$ = 14.7, N*C*N), 137.2 (dd, $^{1}J_{C\text{-}P}$ = 42.0, $^{3}J_{C\text{-}P}$ = 2.7, C_{ipso}P), 134.7 (dd, $^{1}J_{C\text{-}P}$ = 43.8, $^{3}J_{C\text{-}P}$ = 6.0, C_{ipso}P), 132.0 (t, $^{4}J_{C\text{-}P}$ = 5.8, $C_{Ar\text{-}meta}$), 131.0 (t, $^{4}J_{C\text{-}P}$ = 4.5, $C_{Ar\text{-}meta}$), 129.9 (s, $C_{Ar\text{-}para}$), 129.9 (s, $C_{Ar\text{-}para}$), 128.7 (t, $^{3}J_{C\text{-}P}$ = 4.5, $C_{Ar\text{-}orto}$), 128.2 (t, $^{3}J_{C\text{-}P}$ = 4.9, $C_{Ar\text{-}orto}$), 122.8 (s, CH_{Im}), 86.5 (bs, CH_{COD}), 51.7 (bs, CH_{COD}), 48.8 (s, CH_2N), 34.4 (bs, $CH_{2\text{-COD}}$), 30.3 (bs, $CH_{2\text{-COD}}$), 30.0-29.5 (m, CH_2P). $^{31}P\{^{1}H\}$ NMR (CD_2Cl_2, 162 MHz): δ –21.0 (s, PPh_2). ^{19}F NMR (CD_2Cl_2, 282 MHz): δ –152.0 (s, BF_4). HRMS (ESI) m/z Calcd. for $C_{39}H_{42}IrN_2P_2$ (M–BF_4) 793.2449, found 793.2441.

Synthesis of 4. A solution of **PCO-Cl** (64 mg, 0.17 mmol) in 5 mL of dichloromethane was reacted with $AgBF_4$ (33 mg, 0.17 mmol) at room temperature for 1h. The resulting suspension was filtered and added dropwise at 0 °C over a solution of $[Ir(\mu\text{-}OCH_3)(COD)]_2$ (56 mg, 0.085 mmol). The resulting red solution was stirred for 2 h at room temperature and the solvent evaporated under reduced pressure. The residue was washed with pentane (3 x 10 mL) and diethyl ether (3 x 10 mL) and dried under vacuum to give the title complex as a red solid in a 73 % yield (94 mg, 0.125). ^{1}H NMR (CD_2Cl_2, 400 MHz, 343

K): δ 7.58-7.50 (m, 4H, CH_{Ar}), 7.49-7.42 (m, 4H, CH_{Ar}), 7.23-7.25 (m, 2H, CH_{Ar}), 7.08 (bs, 2H, CH_{Im}), 5.14 (bs, 1H, CH_{COD}), 4.80-4.66 (m, 1H, C$H_2$$N^{PPh2}$), 4.64-4.52 (m, 1H, C$H_2$$N^{PPh2}$), 4.52-4.41 (m, 2H, C$H_{COD}$ + m, 1H, C$H_2$$N^{OMe}$), 4.36-4.13 (m, 1H, C$H_2$$N^{OMe}$), 3.69-3.60 (m, 1H, C$H_2$O), 3.51-3.42 (m, 1H, C$H_2$O), 3.26 (s, 3H, OC$H_3$), 3.09 (bs, 1H, C$H_{COD}$), 2.95-2.84 (m, 1H, C$H_2$P), 2.71-2.54 (m, 2H, C$H_{2\text{-}COD}$), 2.54-2.42 (m, 1H, C$H_2$P), 2.34-2.20 (m, 2H, C$H_{2\text{-}COD}$), 2.18-2.05 (m, 1H, C$H_{2\text{-}COD}$), 1.87-1.72 (m, 2H, C$H_{2\text{-}COD}$), 1.62-1.50 (m, 1H, C$H_{2\text{-}COD}$). $^{13}C\{^1H\}$ NMR APT (CD_2Cl_2, 100 MHz, 343 K): δ 171.6 (d, $^2J_{CP}$ = 13.7, C_{NHC}), 135.3 (d, J_{CP} = 14.6, CH_{Ar}), 134.1 (d, $^1J_{CP}$ = 53.2, $C_{Ar\text{-}ipso}$), 131.9 (s, CH_{Ar}), 131.2 (d, J_{CP} = 9.5, CH_{Ar}), 130.7(s, CH_{Ar}), 129.1 (d, J_{CP} = 11.2, CH_{Ar}), 128.6 (d, J_{CP} = 10.3, CH_{Ar}), 122.4 (s, CH_{im}), 121.7 (s, CH_{im}), 90.2 (d, $^2J_{CP}$ = 7.9, CH_{COD}), 83.2 (d, $^2J_{CP}$ = 15.6, CH_{COD}), 80.4 (s, CH_{COD}), 78.6 (s, CH_{COD}), 71.2 (s, CH_2O), 59.1 (s, OCH_3), 50.7 (s, CH_2N^{OMe}), 49.7 (s, CH_2N^{PPh2}), 36.1 (s, $CH_{2\text{-}COD}$), 36.0 ($CH_{2\text{-}COD}$), 27.0 (s, $CH_{2\text{-}COD}$), 26.6 (s, $CH_{2\text{-}COD}$), 25.2 (d, $^2J_{CP}$ = 39.4, CH_2P). $^{31}P\{^1H\}$ NMR (CD_2Cl_2, 162 MHz, 343 K): δ 17.9 (s, PPh_2). ^{19}F NMR (CD_2Cl_2, 376 MHz, 343 K): δ –152.0 (s, BF_4). HRMS (ESI) m/z Calcd. for $C_{28}H_{35}IrN_2OP$ (M–BF_4) 639.2112, found 639.2098.

Synthesis of 5. A solution of **3b** (100 mg, 0.113 mmol) in acetonitrile (5 mL) was reacted with CO (2 bar) in a Fisher-Porter tube and stirred for 48 h at room temperature. Subsequently, the CO atmosphere was removed and an atmosphere of H_2 (2 bar) was introduced. The resulting solution was stirred for 48 h at room temperature, after which time the solvent was evaporated under reduced pressure and the residue washed with diethyl ether and dried under vacuum to give a pale-yellow solid in a 43 % yield, (39 mg, 0.054 mmol). ^1H NMR (CD_2Cl_2, 400 MHz): δ 7.85-7.76 (m, 2H, CH_{Ar}), 7.60-7.52 (m, 2H, CH_{Ar}), 7.46-7.33 (m, 2H, CH_{Ar}), 7.13 (bs, 2H, CH_{Im}), 4.27-4.23 (m, 4H, CH_2N), 3.13-3.00 (m, 2H, CH_2P), 2.65-2.54 (m, 2H, CH_2P), –10.44 (1H, J_{HH} = 3.7, J_{HP} = 15.6, Ir-*H*), –11.52 (1H, J_{HH} = 3.7 J_{HP} = 14.2, Ir-*H*). $^{13}C\{^1H\}$ NMR APT (CD_2Cl_2, 100 MHz, 343 K): δ 171.6 (bs, *C*O), 144.7 (bs, C_{NHC}), 133.9 (d, J_{CP} = 6.8, CH_{Ar}), 133.4 (t, $^2J_{CP}$ = 28.5, $C_{Ar\text{-}ipso}$), 132.1 (d, $^1J_{CP}$ = 31.9, $C_{Ar\text{-}ipso}$), 131.6 (s, CH_{Ar}), 131.1 (s, CH_{Ar}), 131.0 (d, J_{CP} = 6.1, CH_{Ar}), 129.0 (d, J_{CP} = 6.0, CH_{Ar}), 122.5 (s, CH_{im}), 48.3 (s, CH_2N), 25.5 (d, $^2J_{CP}$ = 19.1, CH_2P). $^{31}P\{^1H\}$ NMR (CD_2Cl_2, 162 MHz, 343 K): δ –3.9 (s, PPh_2). ^{19}F NMR (CD_2Cl_2, 376 MHz, 343 K): δ –151.7 (s, BF_4).HRMS (ESI) m/z Calcd. for $C_{32}H_{30}IrN_2OP_2$ (M–2H–BF_4) 713.1459, found 713.1489. FTIR: ν_{CO} = 2002 cm^{-1}.

Reactivity experiments

Reactivity of 4 with CO followed by H_2 (preparation of **6**). The experimental procedure was analogous to that employed for the synthesis of **5**. In this case the acetonitrile was not evaporated to dryness to avoid the decomposition of **6**.

Reactivity of 3b or 4 with HCOOH and pyridine in CD_2Cl_2. An excess of HCOOH (5µL, 0,133 mmol) and pyridine (36 µL, 0,447 mmol) was added over a solution of **3b** (22 mg, 0.024 mmol) or **4** (18 mg, 0.024 mmol) in CD_2Cl_2 (0.5 mL). The reaction was monitored by NMR spectroscopy.

General procedure for the catalytic dehydrogenation of formic acid.

Catalysis in neat HCOOH. The reactor was placed in an oil bath at 80 ^oC with 450 µL of HCOOH and 265 mg of HCOONa (30 mol%). Once the pressure was stabilized, a solution of catalyst (0.002 mmol, 0.016 mol %) in 50 µL of HCOOH was added with a syringe. The amount of gases (CO_2 + H_2) produced during the reaction was calculated using the Ideal Gas Law.

Catalysis in a 1:1 (v/v) DMC:HCOOH mixture. The reactor was placed in an oil bath at 80 ^oC with 450 µL of HCOOH, 500 µL of DMC and 265 mg of HCOONa (30 mol%). Once the pressure was stabilized, a solution of catalyst (0.002 mmol, 0.016 mol %) in 50 µL of HCOOH was added with a syringe. The amount of gases (CO_2 + H_2) produced during the reaction was calculated using the Ideal Gas Law.

Catalysis in a 4:1 (v/v) DMC:HCOOH and 1:1 (v/v) H_2O:HCOOH mixture. An analogous procedure to that described above was employed.

Catalysis in a 1.5:1 (v/v) Et_3N:HCOOH. The reactor was placed in an oil bath at 80 ^oC with 450 µL of HCOOH and 725 µL of Et_3N. Once the pressure was stabilized, a solution of catalyst (0.002 mmol, 0.016 mol %) in 50 µL of HCOOH was added with a syringe. The amount of gases (CO_2 + H_2) produced during the reaction was calculated using the Ideal Gas Law.

2. NMR Spectra

2.1. Complex 3a

^{1}H NMR spectrum

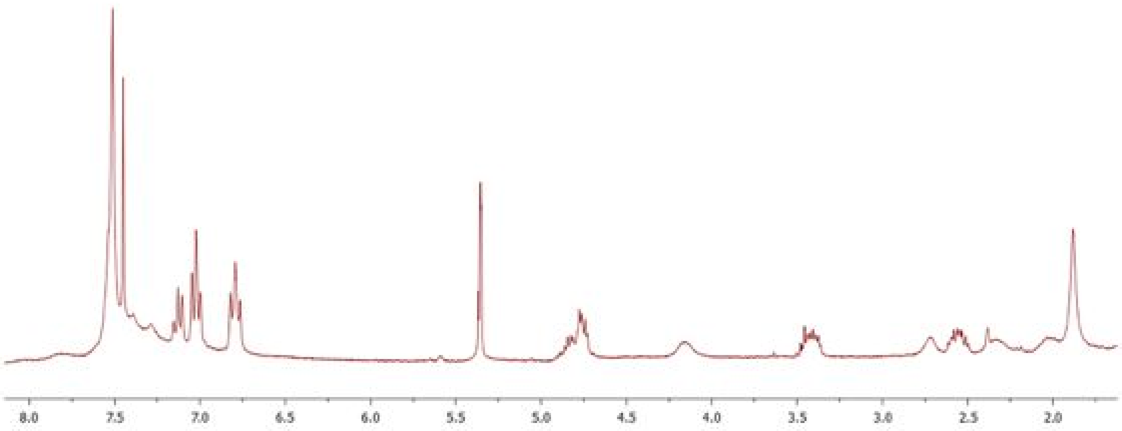

^{31}P NMR spectrum

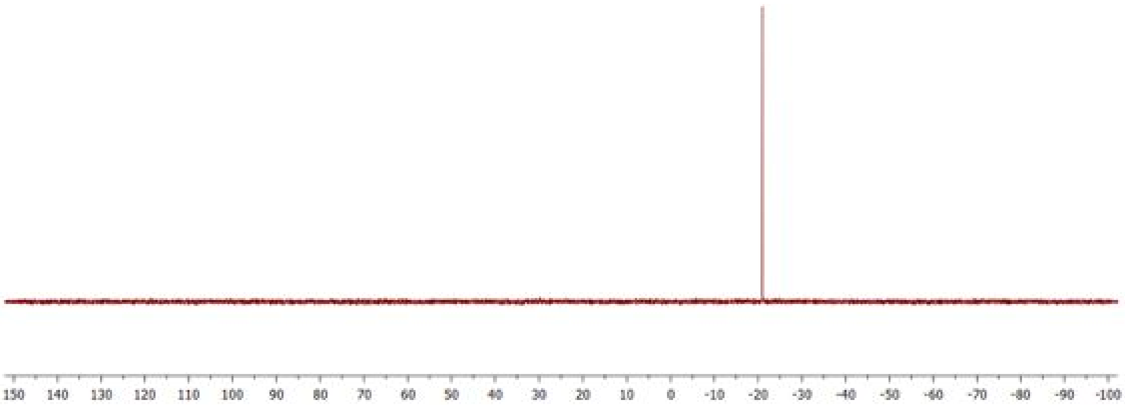

APT NMR spectrum

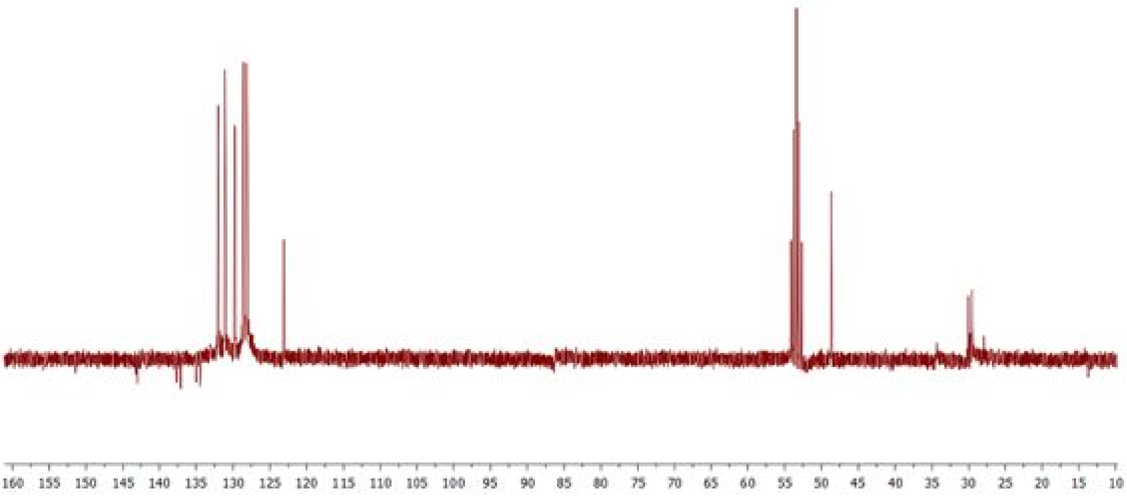

^{1}H-^{1}H COSY NMR spectrum

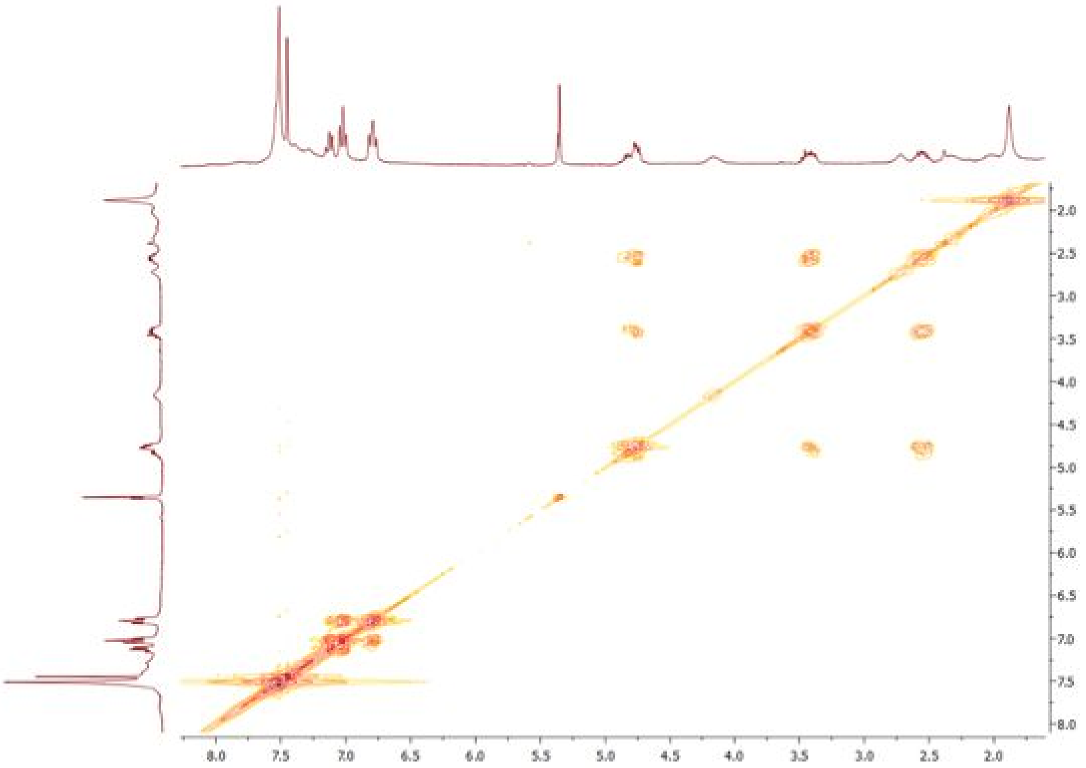

^{1}H-^{13}C HSQC NMR spectrum

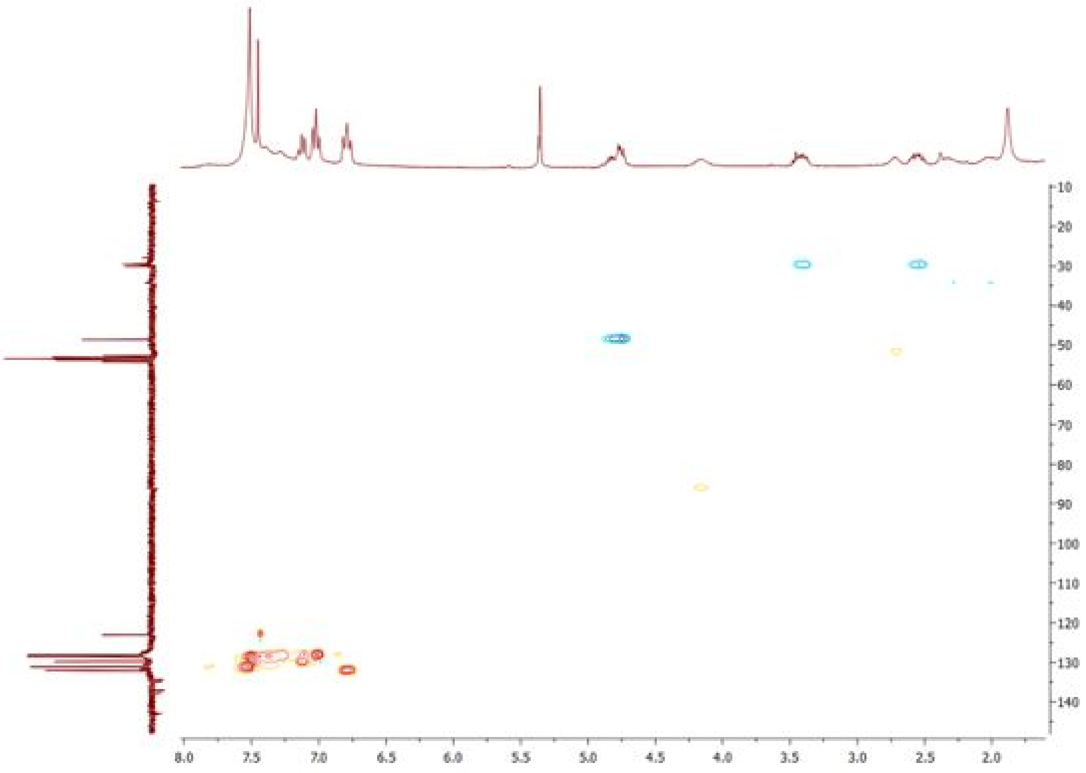

2.2. Complex 3b

^{1}H NMR spectrum

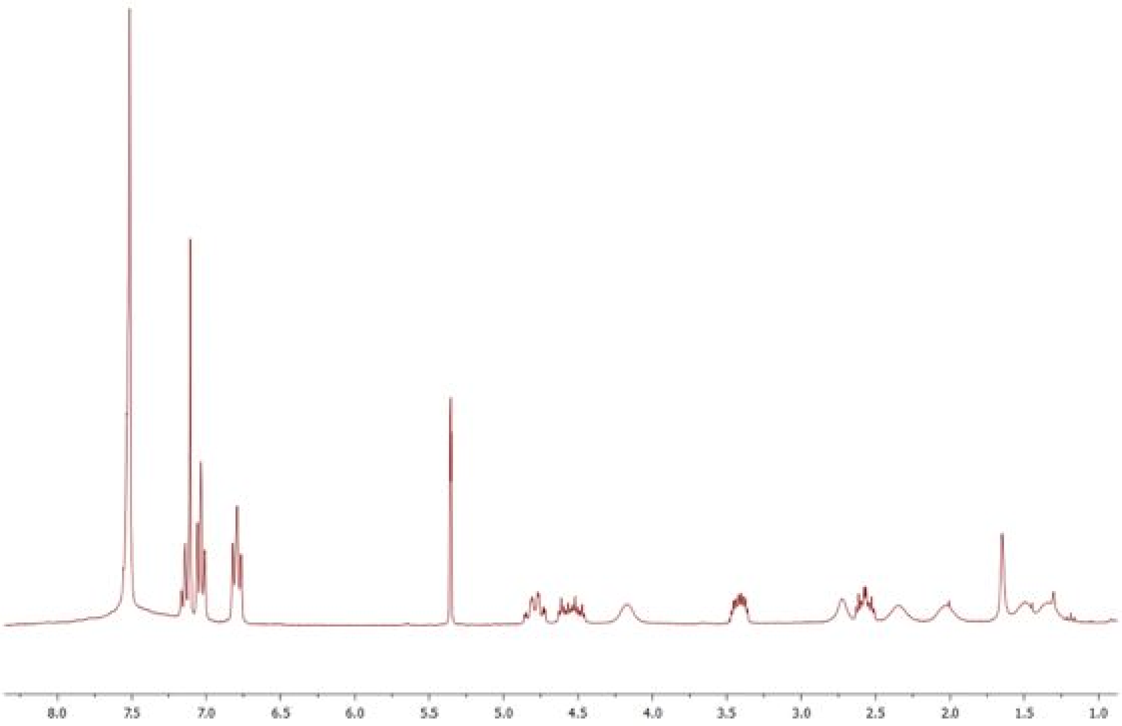

^{31}P NMR spectrum

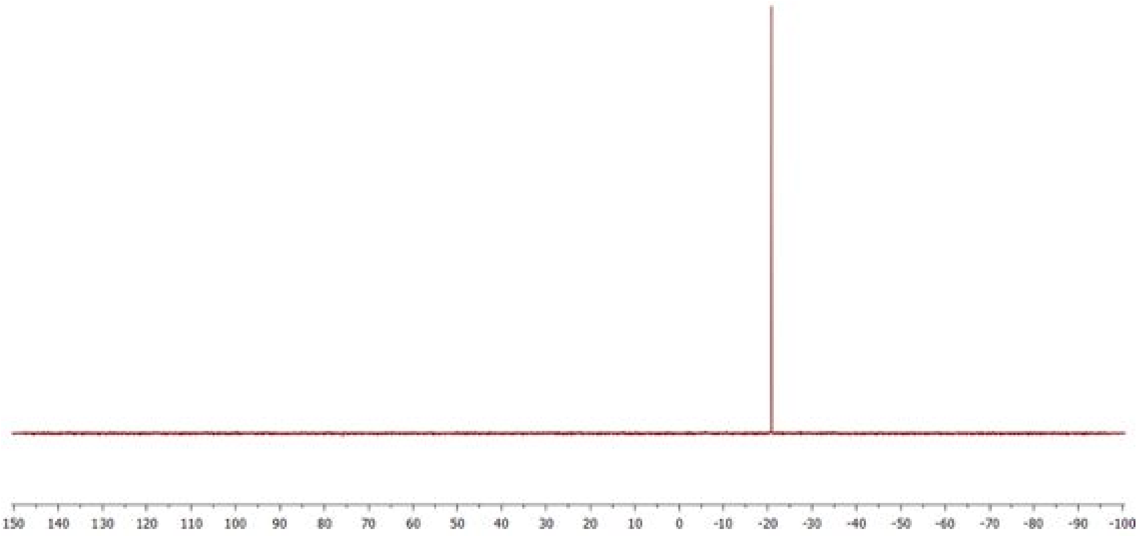

^{19}F NMR spectrum

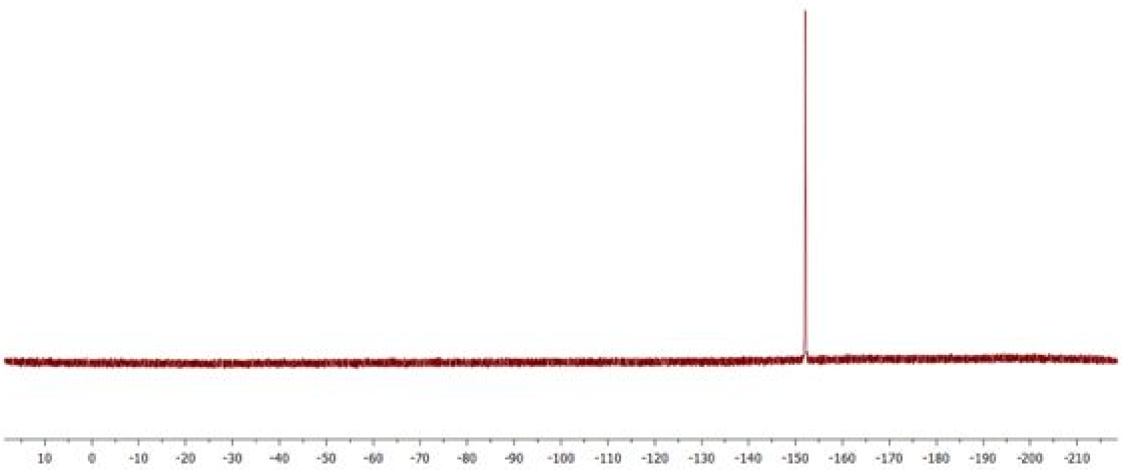

APT NMR spectrum

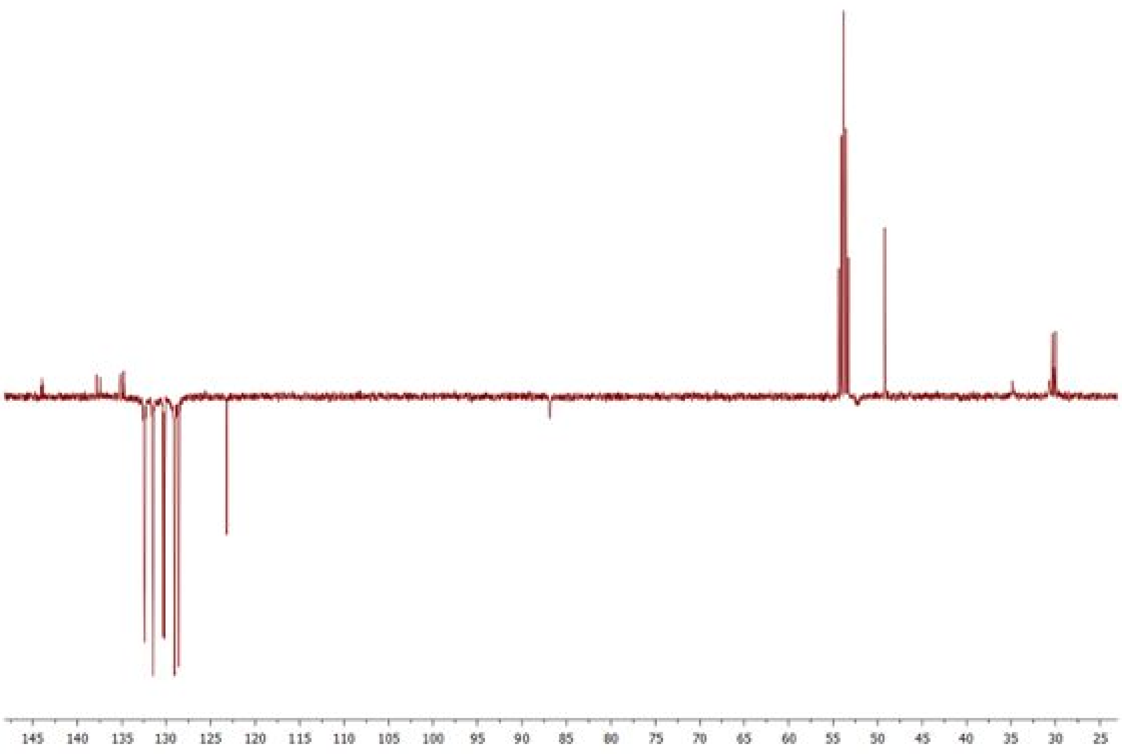

^{1}H-^{1}H COSY NMR spectrum

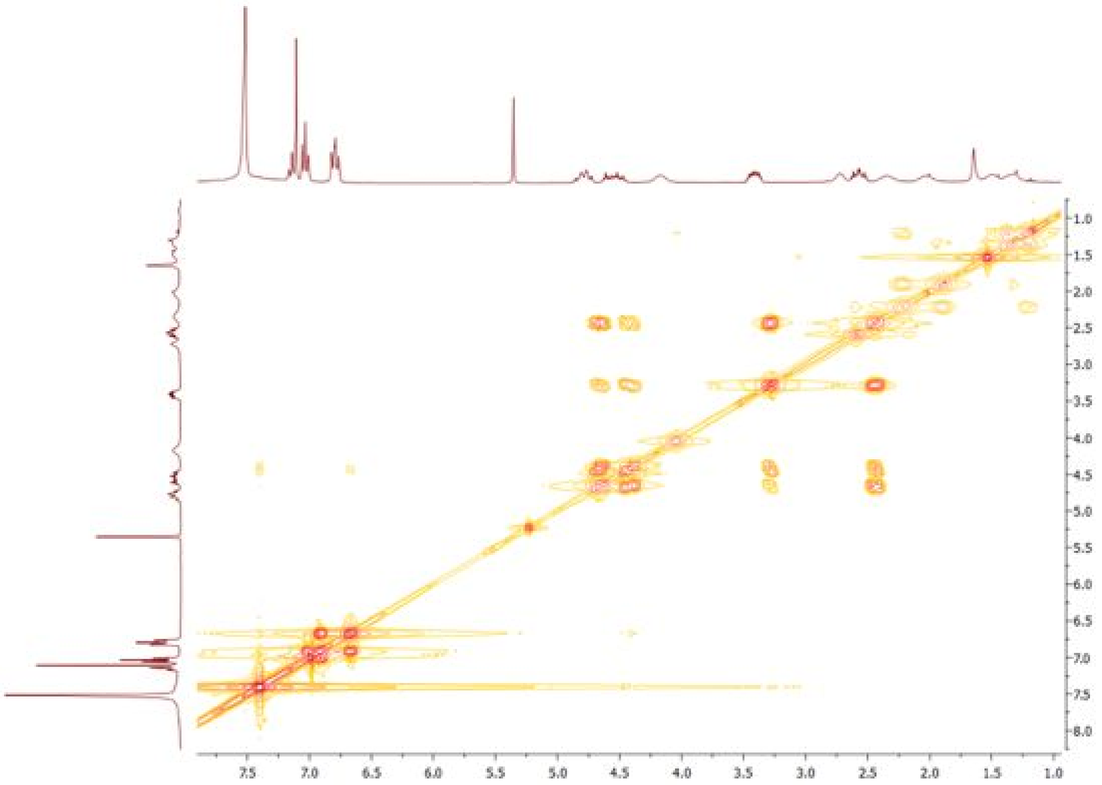

^{1}H-^{13}C HSQC NMR spectrum

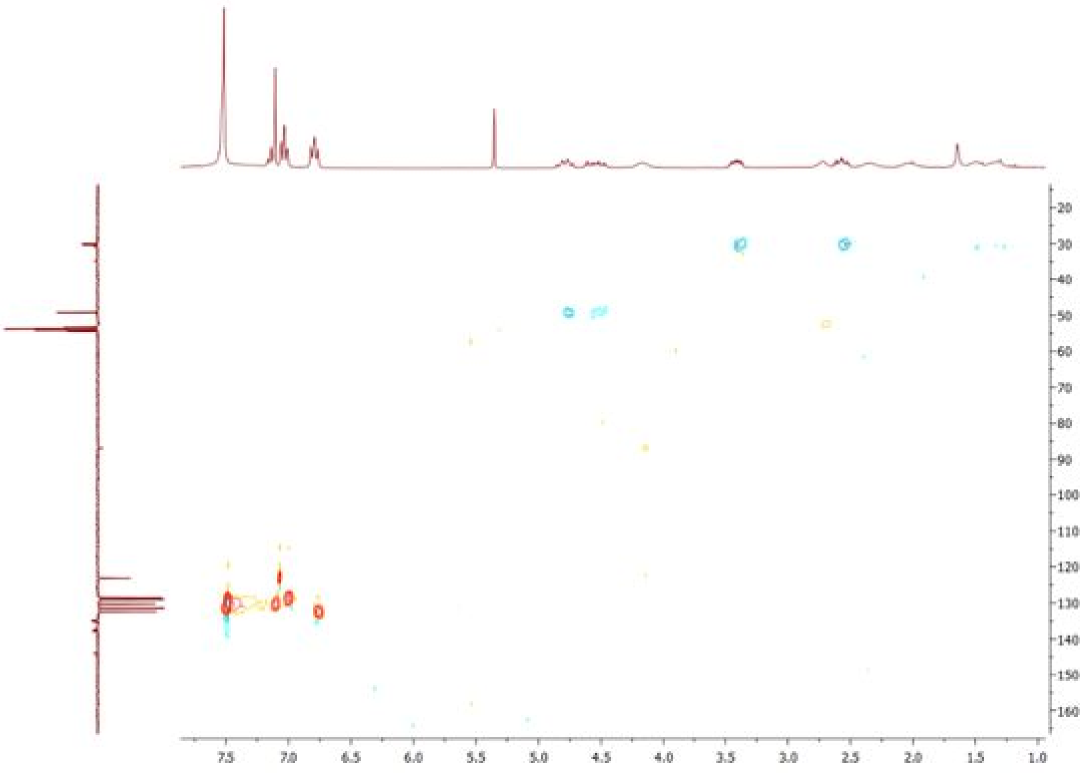

2.3. Complex 4

^1H NMR spectrum

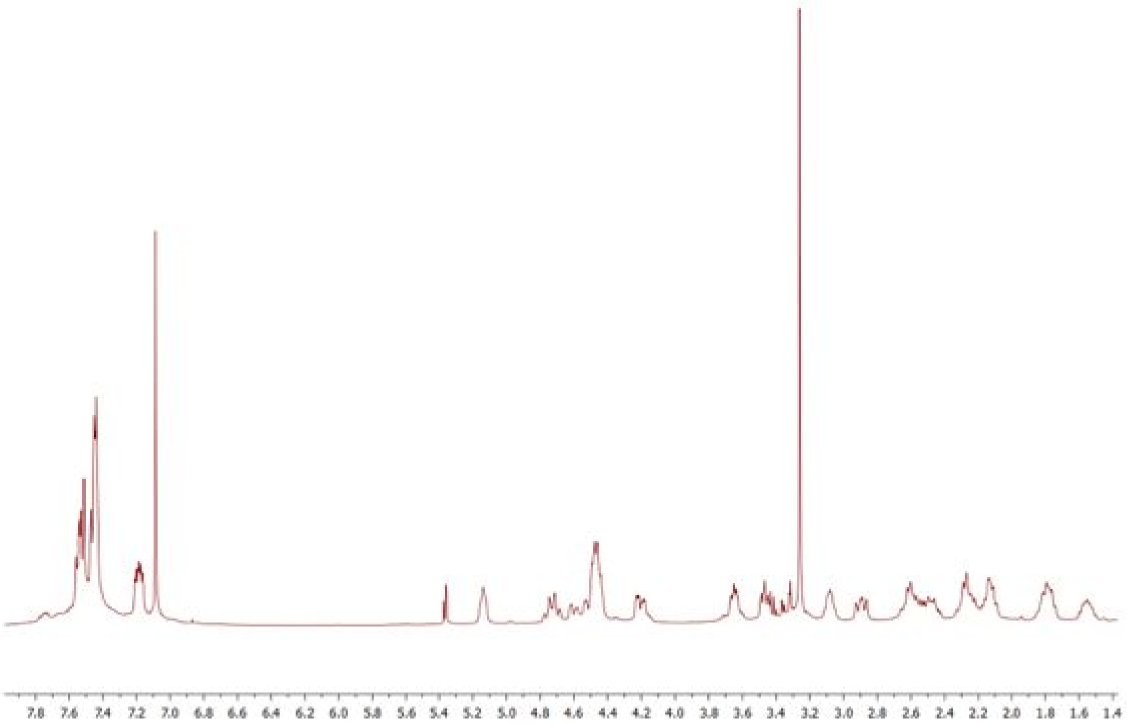

^{31}P NMR spectrum

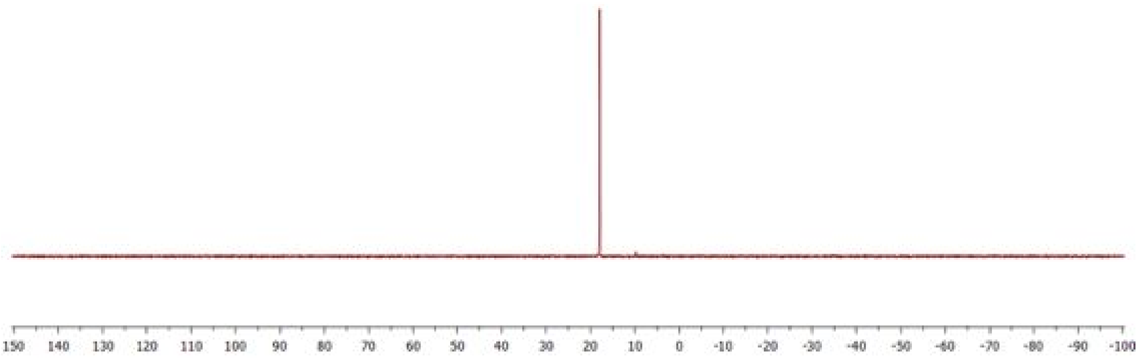

^{19}F NMR spectrum

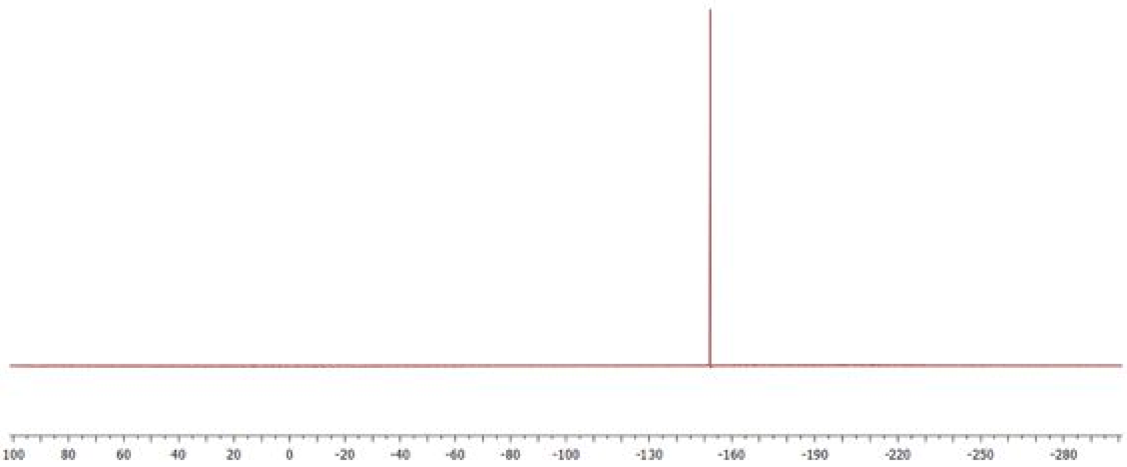

APT NMR spectrum

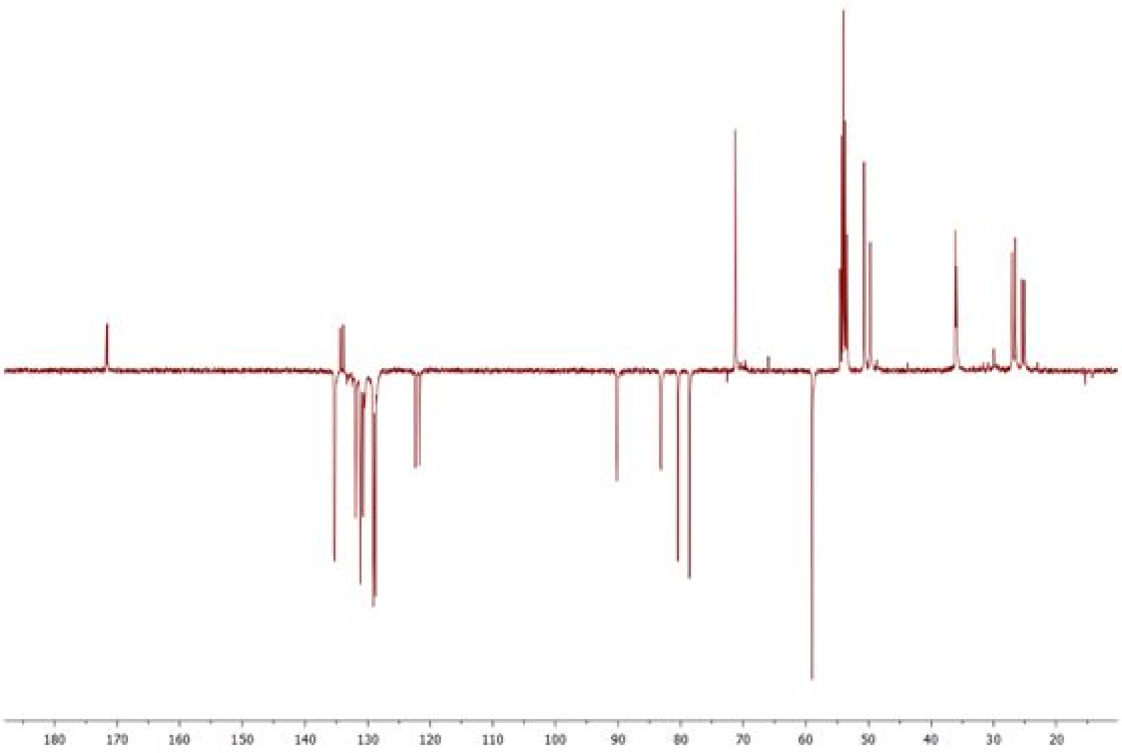

^{1}H-^{1}H COSY NMR spectrum

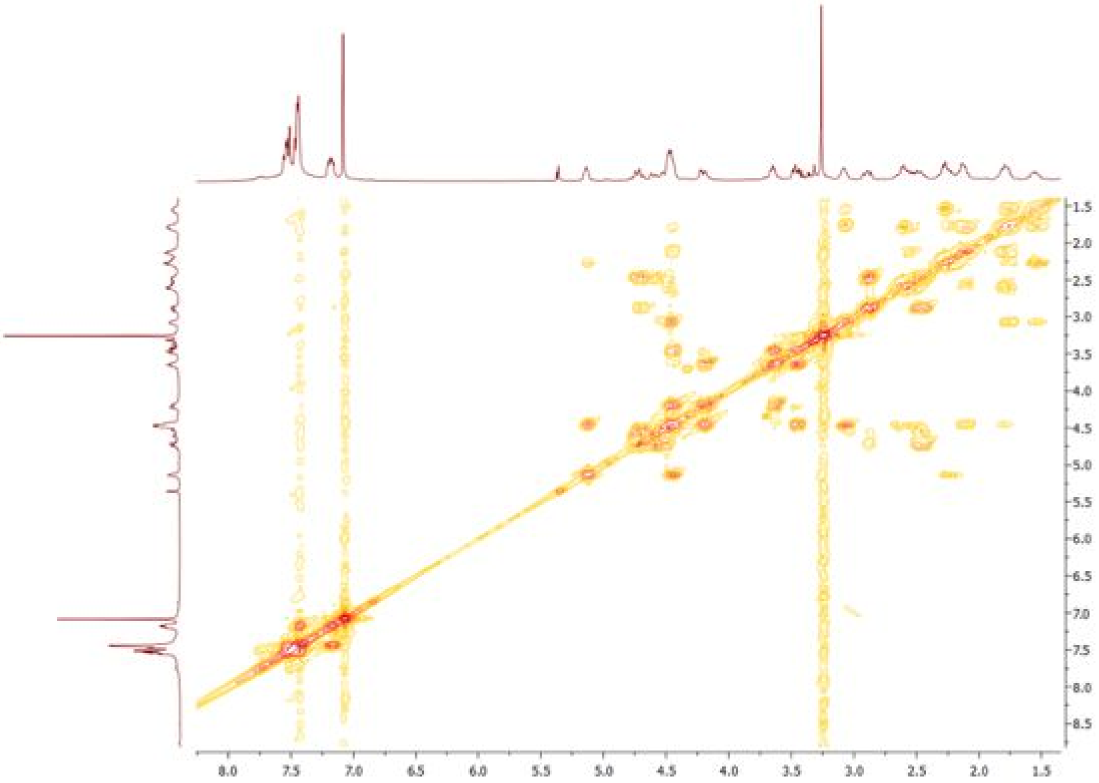

^{1}H-^{1}H HSQC NMR spectrum

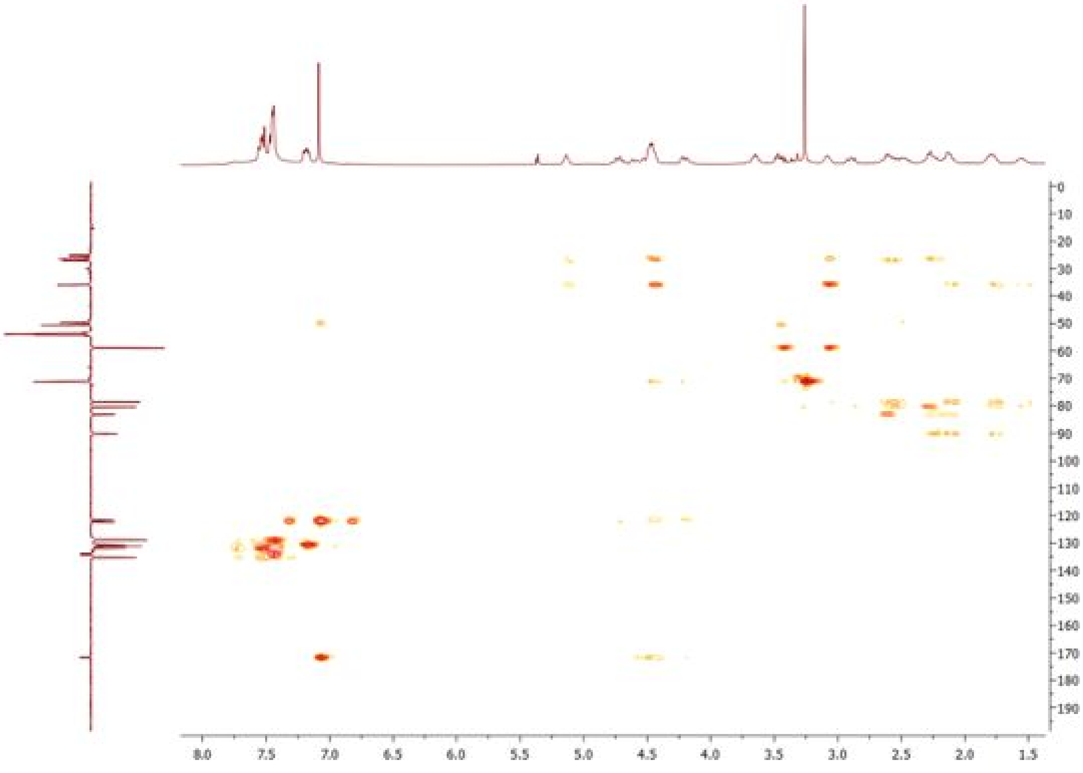

2.4. Complex 5

^1H NMR spectra

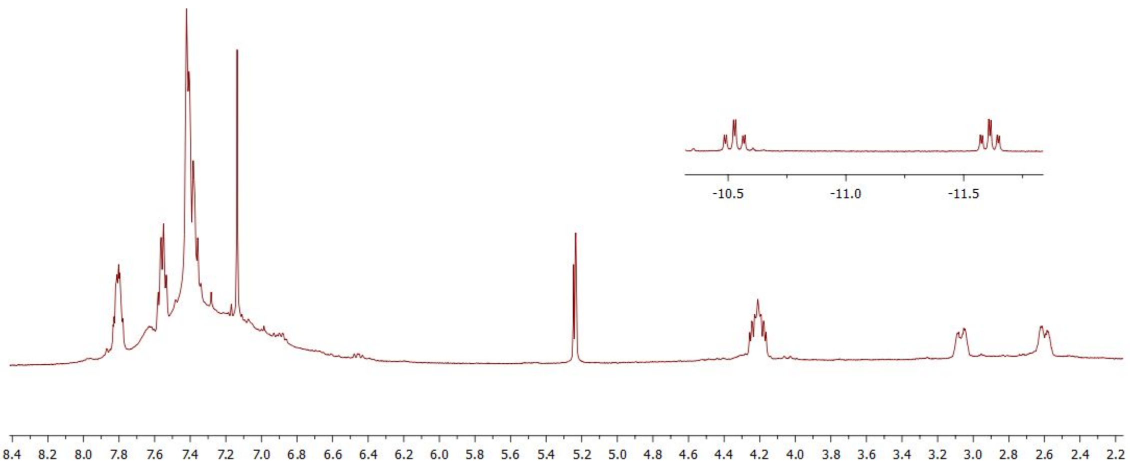

^{31}P NMR spectrum

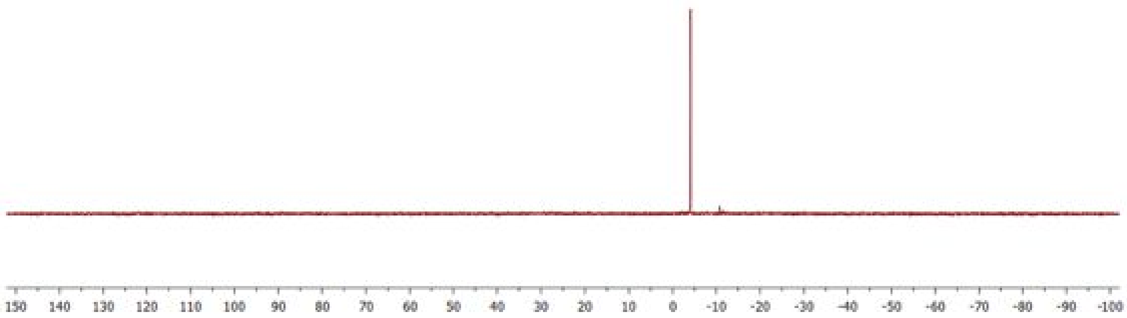

^{19}F NMR spectrum

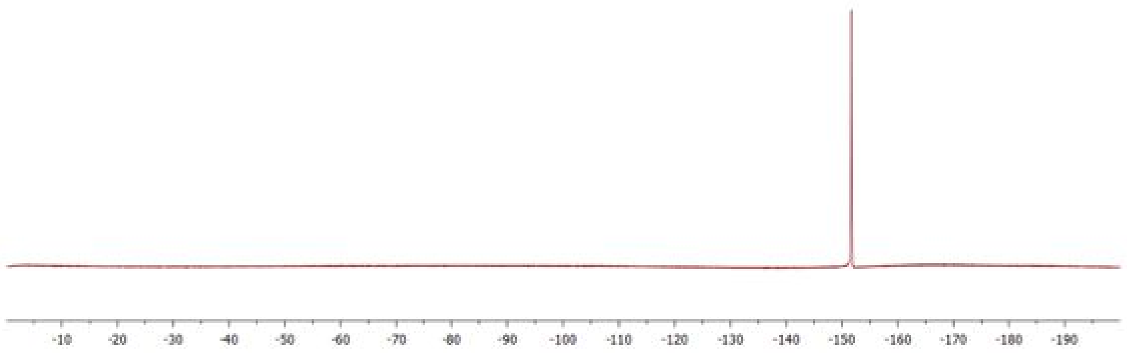

APT NMR spectrum

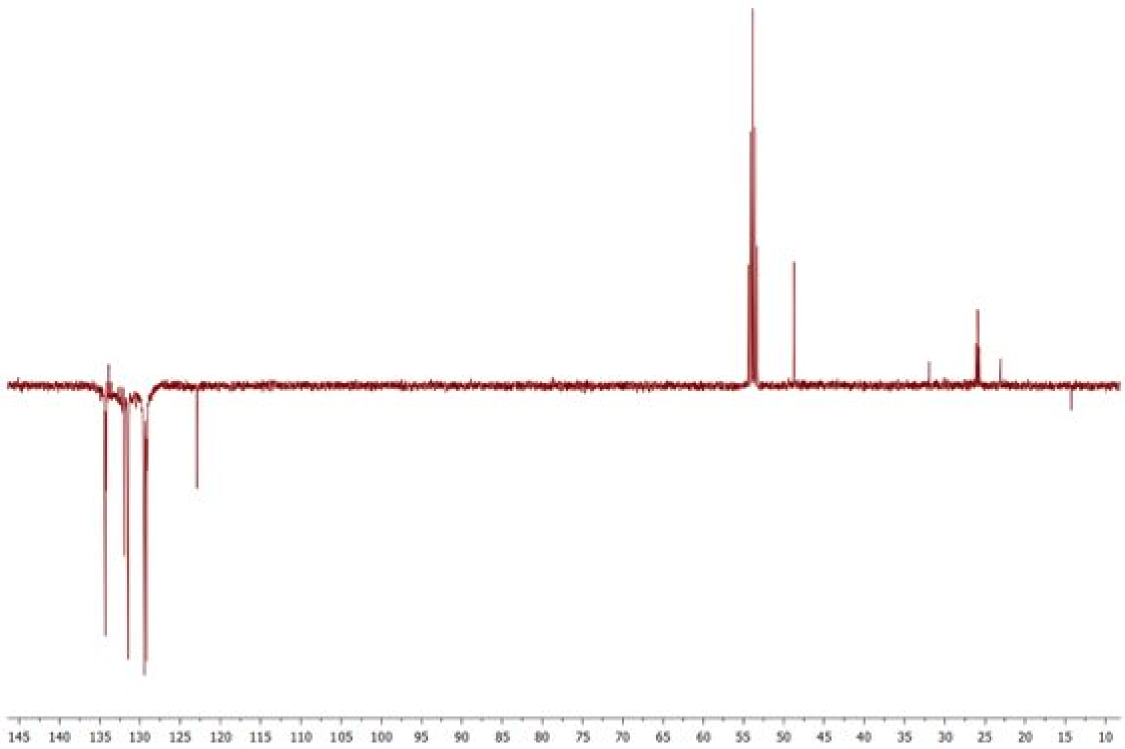

^{13}C NMR spectrum

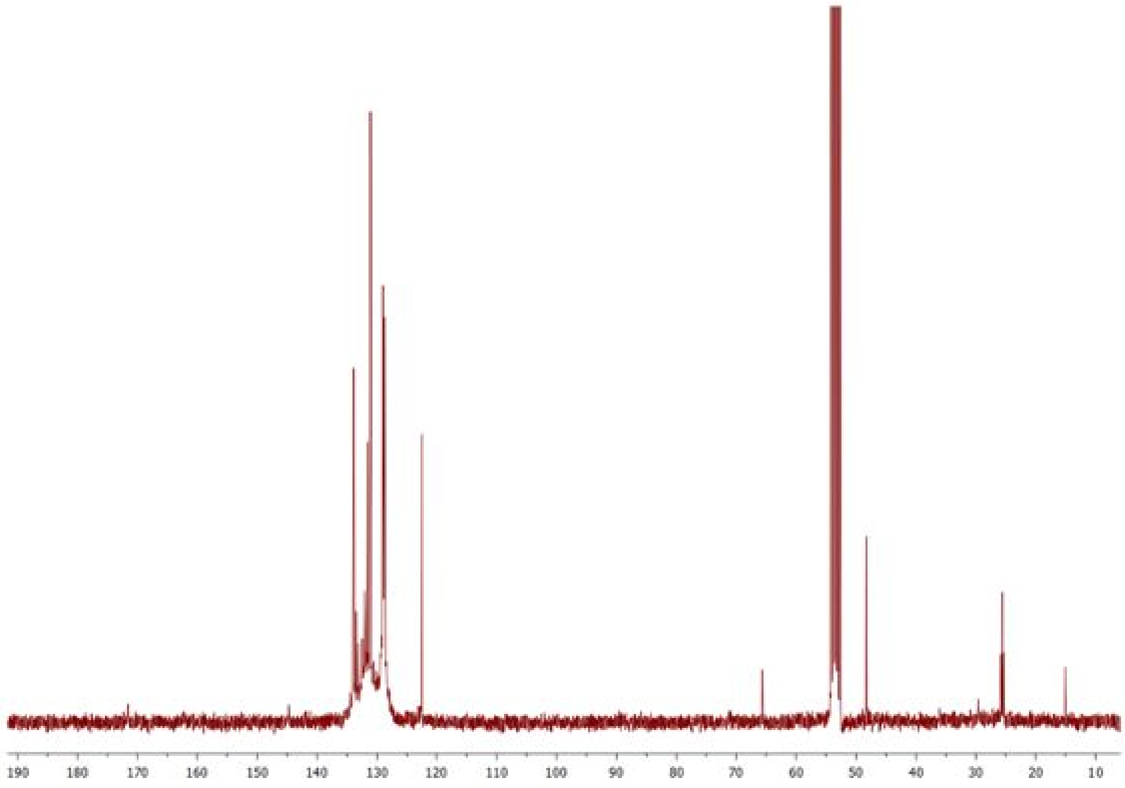

^{1}H-^{1}H COSY NMR spectrum

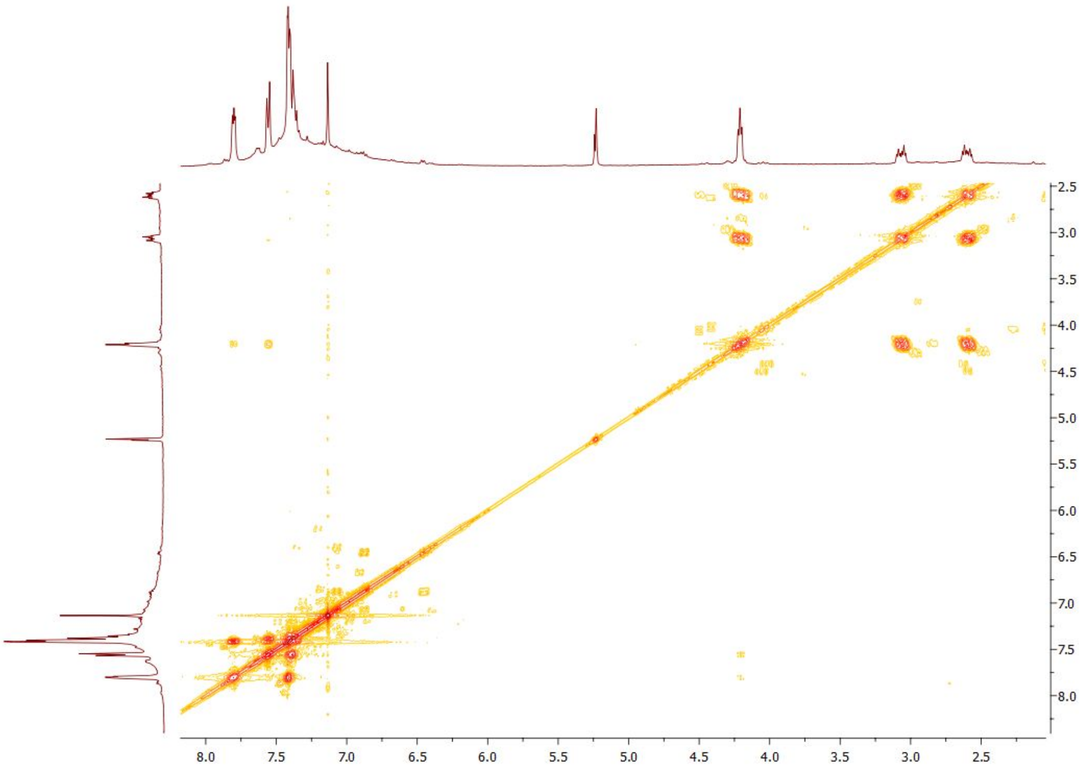

^{1}H-^{13}C HSQC NMR spectrum

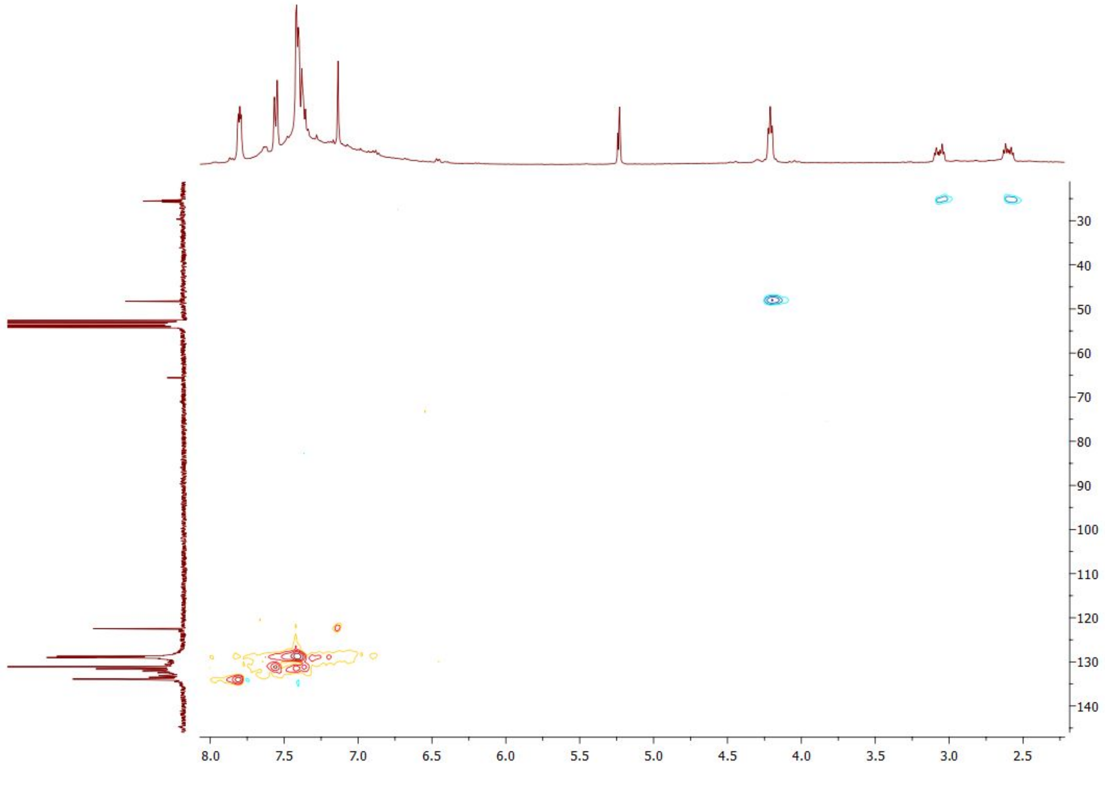

3. IR spectrum of the mixture of gases obtained from catalysis

The mixture of gases obtained from the reaction catalyzed by **4** in 1:1 (v/v) DMC:HCOOH was studied by IR spectroscopy, showing an excellent selectivity towards the dehydrogenation reaction since no traces CO were detected (the CO bands should appear between 2100 and 2200 cm^{-1}).

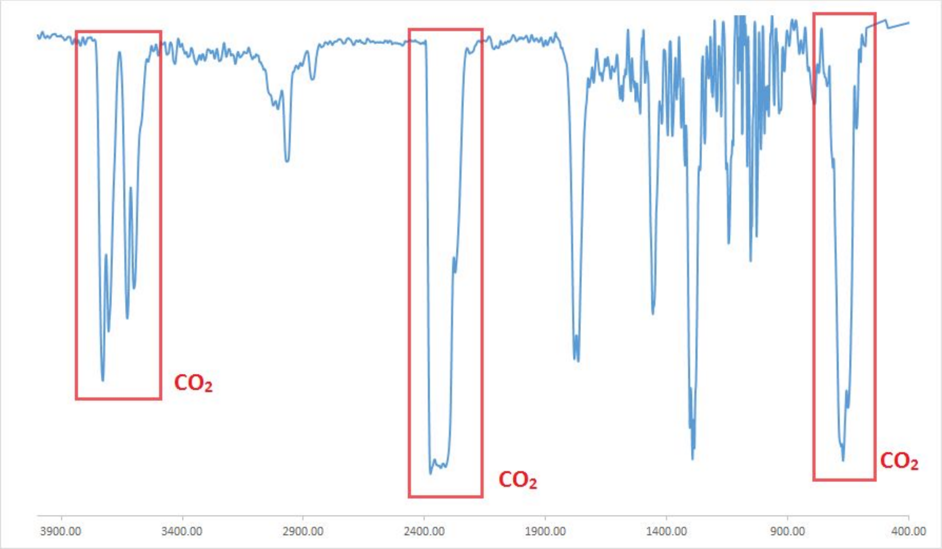

Figure S1. IR Spectrum of the gaseous products obtained in a 1:1 (v/v) DMC:HCOOH mixture (30 mol% of HCOONa and 0.016 mol% **4**).

4. Gas Chromatography of the mixture of gases obtained from catalysis

The mixture of gases obtained from the reaction catalyzed by **4** in 1:1 (v/v) DMC:HCOOH was studied by gas chromatography, showing no traces of CO.

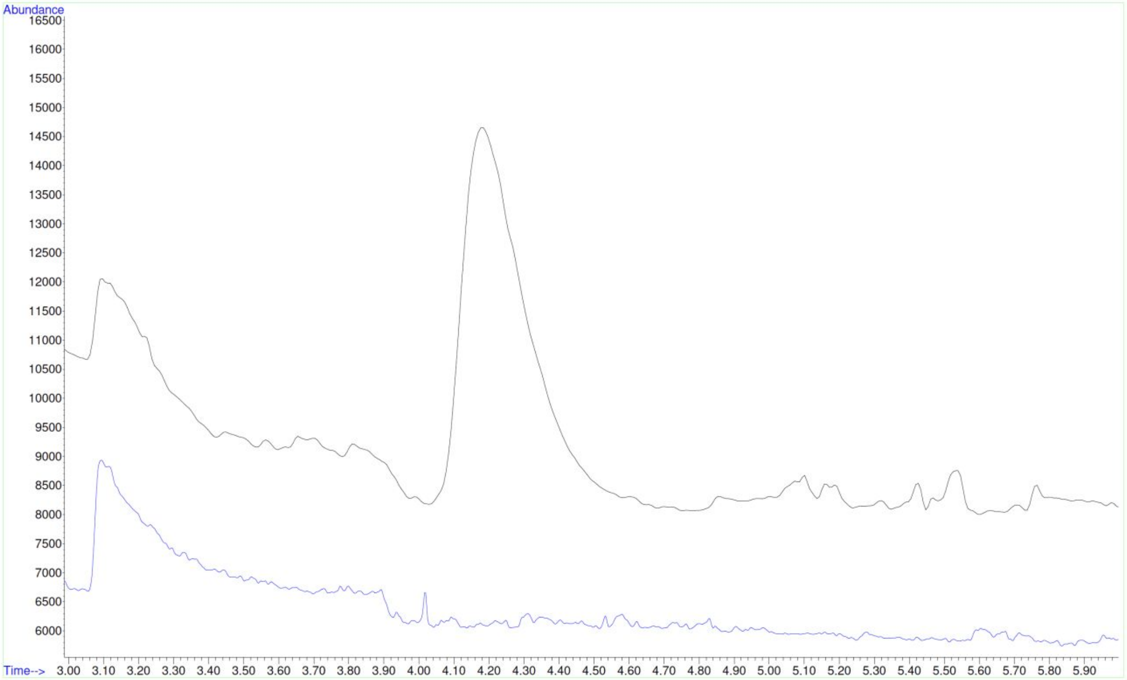

Figure S2. GC-MS of the gaseous products obtained in a 1:1 (v/v) DMC:HCOOH mixture (30 mol% of HCOONa and 0.016 mol% **4**) in blue (below) and a 50 ppm mixture of CO in CO_2 in black (top); CO peak at 4.20 s.

5. Kinetic profiles KIE

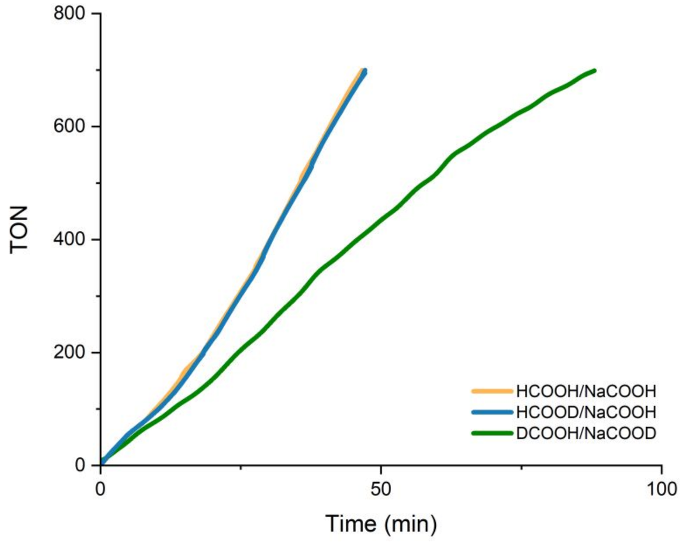

Figure S3. Kinetic profiles of TON versus time for the kinetic isotope effect experiments on the catalytic dehydrogenation of formic acid with **4** (30 mol% HCOONa, DMC:HCOOH (4:1), 0.075 mol% **4**).

6. Kinetic profiles at early stages

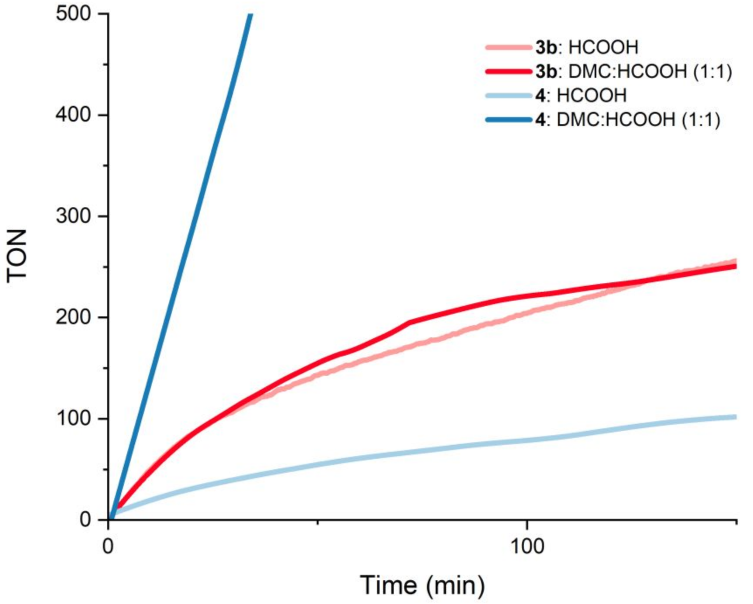

Figure S4. Early stage reaction profiles for the dehydrogenation of HCOOH (0.016 mol% of **3b** or **4**, 30 mol% of HCOONa at 80 °C) with and without DMC as solvent.

7. Computational Details

DFT calculations were performed by means of the Gaussian09 package, revision D.01.[1] B3LYP exchange-correlation hybrid functional,[2] in conjunction with D3BJ empirical dispersion scheme[3] (B3LYP-D3BJ) and an *"ultrafine"* integration grid were applied for the calculations (geometry optimizations and single points). With respect to the basis sets, we selected Ahlrichs def2-SVP for geometry optimizations and def2-TZVP for energy refinement via single point calculations on the optimized intermediates and transition structures.[4] Solvent effects were modelled through the Polarizable Continuum Model (PCM), as implemented in the Gaussian09 suite, which was applied to both, gradients (geometry optimizations) and energy calculations.[5] For the reactions in dimethyl carbonate (DMC), which is not included in the Gaussian09 database, we selected a relatively similar available solvent (butyl ethanoate) and exchanged its dielectric constant for that of DMC. Notice that, although this approximation may not be very rigorous, we expect it to have a low impact on the results accuracy, as both, DMC and butyl ethanoate are quite non-polar solvents. Moreover, we considered that, as the solvent is in great excess, it would occupy the coordination vacancies.

Analytical frequency analysis allowed to confirm the nature of stationary points. In addition, when computing Gibbs energies, we removed the translational entropy contribution, as proposed by Morokuma and co-workers.[6] Molecular representations of the intermediates and transition structures shown in this document were performed by means of CylView software.[7]

We note that we selected the aforementioned methodology on the basis of our previous studies on Ir-based catalysts for the dehydrogenation of formic acid, in which it proved to be suitable for making reasonable proposals in agreement with the experimental observations (see reference 10 in the main text).

8. Mechanism for the activation of 4

The preactivation of **4** (**A**$_{PCO}$) was substantiated by a computational study in both, formic acid (**A**$_{PCO-FA}$) and dimethyl carbonate (**A**$_{PCO-DMC}$); whose outcome is in agreement with the experimental observations, as depicted in Figures S5 and S6. First, we describe the results for the catalyst in formic acid (**A**$_{PCO-FA}$), and then we compare them with dimethyl carbonate.

As a first step, one of the olefins of the COD ligand dissociates, thus allowing the coordination of a formic acid molecule and the ensuing oxidative addition of the O–H bond to afford monohydride **B**$_{PCO-FA}$, 0.5 kcal·mol^{-1} more stable than **A**$_{PCO-FA}$. The process takes place via **TS-AB**$_{PCO-FA}$, requiring to surmount an energy barrier of 11.8 kcal·mol^{-1}. Subsequently, the lowest energy pathway entails the formation of **C**$_{PCO-FA}$ by complete recoordination of the COD olefin, which decreases the energy by 2.0 kcal·mol^{-1}. Then, migratory insertion of one of the coordinated olefins of the COD ligand into the Ir–H bond affords **D**$_{PCO-FA}$ via **TS-CD**$_{PCO-FA}$, with an energy barrier of 10.8 kcal·mol^{-1}. The coordination of a formic acid molecule to **D**$_{PCO-FA}$ lowers the energy by 7.4 kcal·mol^{-1}, yielding **E**$_{PCO-FA}$, with a relative energy of -1.4 kcal·mol^{-1}.

The formate ligand in **D**$_{PCO-FA}$ undergoes a β-hydride abstraction process to yield **F**$_{PCO-FA}$ and a CO_2 molecule. This step requires to surmount an energy barrier of 7.6 kcal·mol^{-1} (with respect to **E**$_{PCO-FA}$), dictated by **TS-EF**$_{PCO-FA}$. As a result, the mono-hydride **F**$_{PCO-FA}$ is produced, with a relative energy of -1.9 kcal·mol^{-1}. At this step, we also considered the β-hydride elimination process (**TS-EF**$_{PCO-FA}$**b**) instead of the hydride abstraction one. However, we discarded such possibility, as it is 3.2 kcal·mol^{-1} higher in energy. Also note that coordination of pyridine drastically stabilizes intermediate **F**$_{PCO-FA}$, leading to **F-py**$_{PCO-FA}$, which likely allows its identification by NMR in the stoichiometric experiments described in the manuscript.

Then, reductive elimination of the alkyl and hydride ligands in **F**$_{PCO-FA}$, through **TS-FG**$_{PCO-FA}$, leads to the formation of the Ir(I) species **G**$_{PCO-FA}$ upon coordination of HCOOH and dissociation of COE. This step bears an energy barrier of 12.9 kcal·mol^{-1} (with respect to **F**$_{PCO-FA}$), and leads to an energy decrease of 3.8

kcal·mol^{-1}, relative to **F*PCO-FA***. The final preactivation step implies oxidative addition of HCOOH, and affords the monohydride **H*PCO-FA*** by means of **TS-GH*PCO-FA***. Then, a molecule of HCOOH coordinates upon a switch of the coordination mode of the formate from κ-*O,O'* to κ-*O* to give **I*PCO-FA***. Remarkably, this step is significantly favourable from a thermodynamic point of view, as **I*PCO-FA*** presents a relative Gibbs energy of -34.2 kcal·mol^{-1}, and is 28.5 kcal·mol^{-1} more stable than **G*PCO-FA***.

Overall, the pre-activation step bears an effective energy barrier of 13.5 kcal·mol^{-1}, dictated by the energy difference between transition state **TS-FG*PCO-FA*** and intermediate **C*PCO-FA***. At this point it is important to note that we also considered the possibility that **B*PCO-FA*** undertakes a hydride transfer (**TS- BC'*PCO-FA***) or a β-hydride elimination of the formate moiety (**TS- BC'*PCO-FA*b**) instead of direct hydride migratory insertion into the COD ligand (depicted in purple and red in Figure S5). However, such processes are higher in energy (3.7 kcal·mol^{-1} for the case of **TS- BC'*PCO-FA* and** is 5.3 kcal·mol^{-1} for **TS- BC'*PCO-FA*b**).

As previously introduced, the same activation pathway was computed for the reaction in dimethyl carbonate (DMC). Notice that complex **4** is now referred to as **A*PCO-DMC***. As shown in Figure S6, the Gibbs energy profile is very similar to that observed for the reaction in formic acid, indicating that the same pre-activation steps are expected to be operative when the reaction is performed in dimethyl carbonate. However, although the rate-determining transition state is the same (**TS-FG*PCO-DMC***), the energy span is higher, 17.2 kcal·mol^{-1} (vs 13.5 kcal·mol^{-1} for the reaction in formic acid), which is also affordable under the reaction conditions. In this sense, it is remarkable that the DMC significantly stabilizes many reaction intermediates with respect to FA, such as **F*PCO*** (which has a relative energy that is 6.2 kcal·mol^{-1} lower in DMC than in FA). The stabilization of the intermediates may be directly related to the higher stability of the catalytic systems in DMC, as pointed out in the main text.

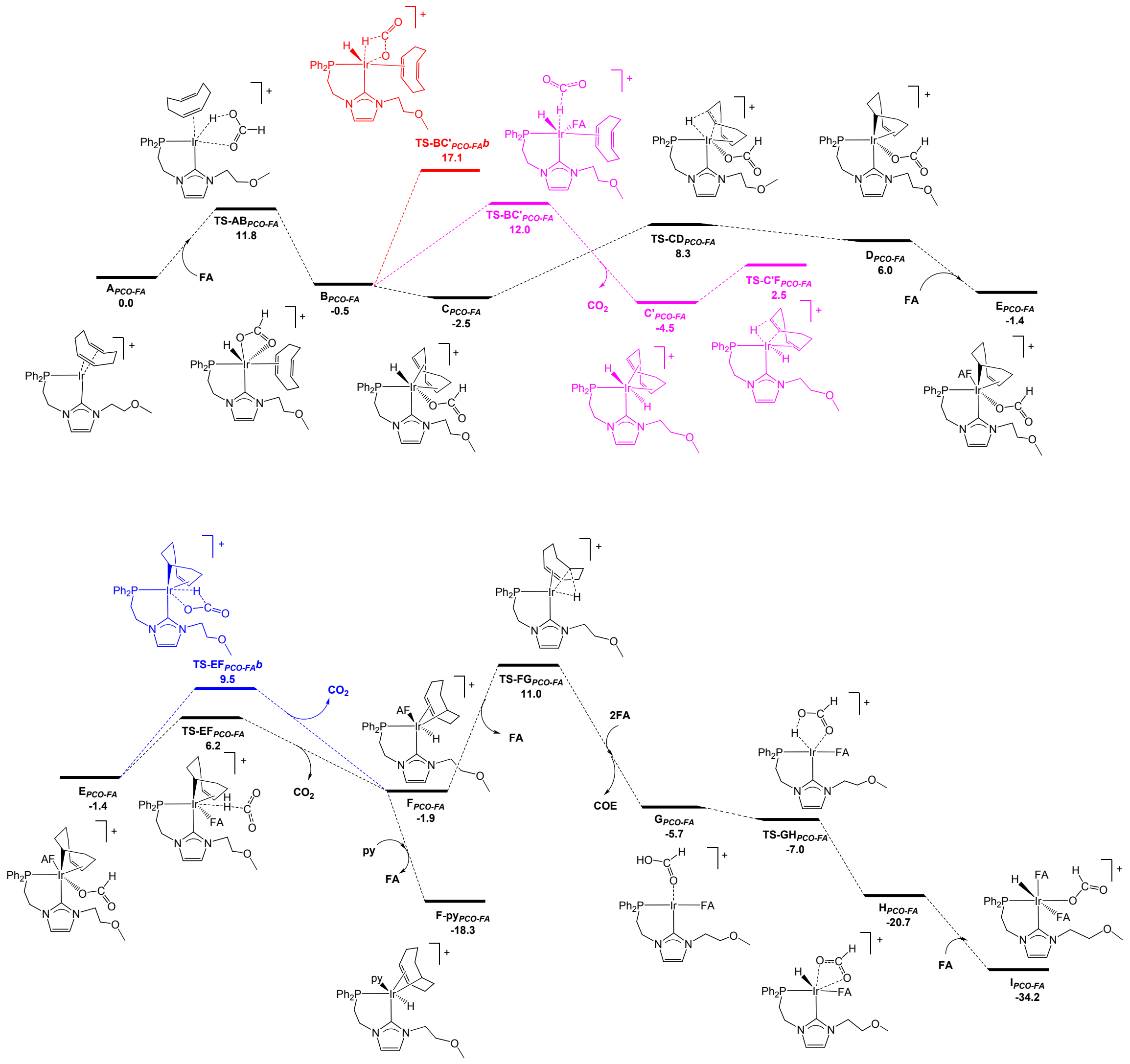

Figure S5. DFT calculated Gibbs free energy profile (in kcal mol^{-1}) for the activation of **4** (**A*PCO-FA***) in HCOOH.

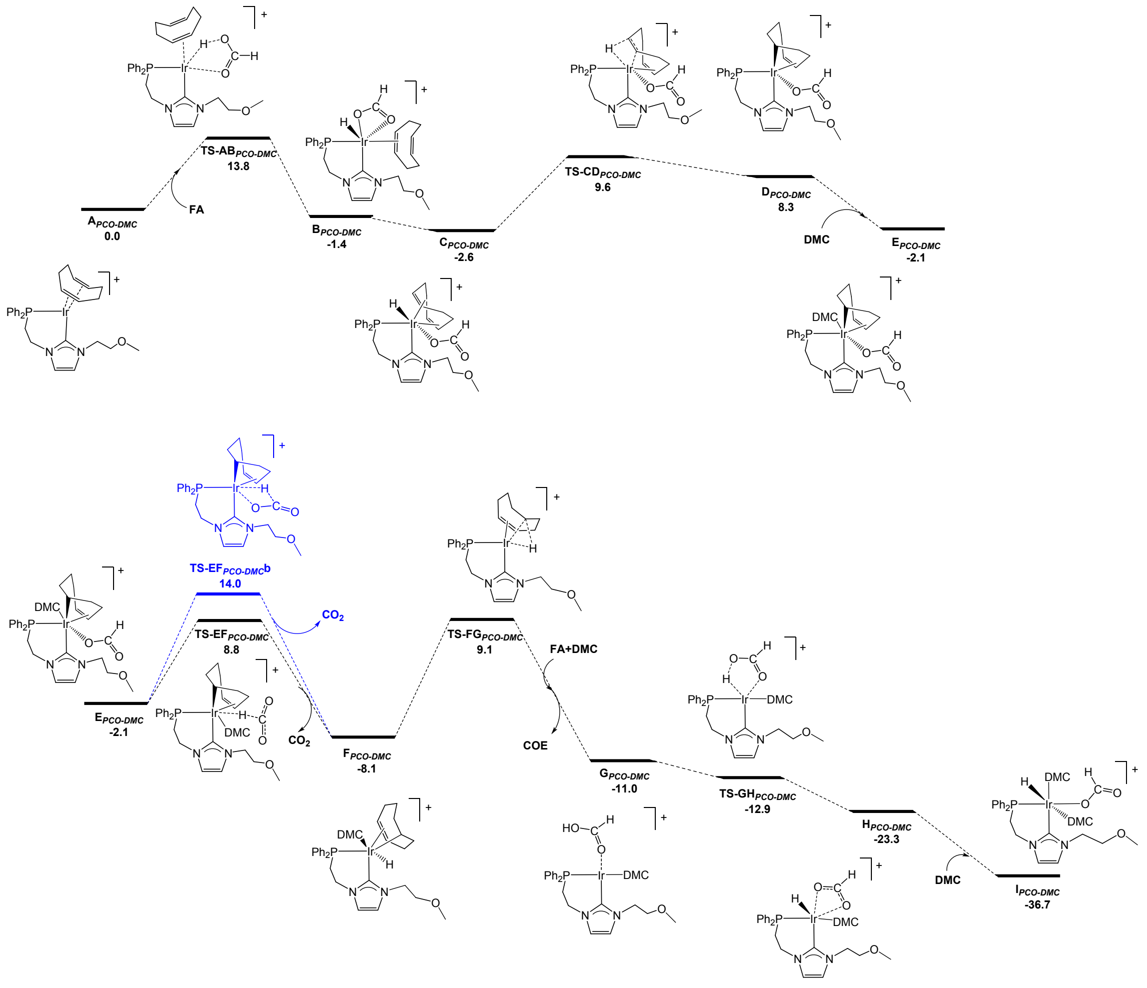

Figure S6. DFT calculated Gibbs free energy profile (in kcal mol^{-1}) for the activation of **4** (**A**$_{PCO\text{-}DMC}$) in dimethyl carbonate.

9. DOSY NMR Experiments

The diffusion coefficients for complexes **4** and **7** were estimated on a Bruker Avance 300 MHz spectrometer employing pulsed gradient spin-echo (PGSE) NMR methods at 300 K spinning the sample with an air-flow of 400 L h^{-1} to prevent convection effects. The value of D20 (diffusion time) employed in the ^{1}H DOSY experiments was set to 100 ms, while P30 (gradient duration) was optimized using the stebpgp1s sequence by progressive decay of the signal intensities of the selected resonances until 1 to 5% of the initial value with maximum strength (gpz6 = 95%). Values of 700 µs and 800 µs were for obtained for 45 mM solutions of **4** and **7** in CD_2Cl_2, respectively.

The gradient strength was varied between 2% and 95% in 16 square spaced increments. The variation of the intensity (I) of the selected resonance is related to the strength of the gradient (G) by Equation S1.

$$\boldsymbol{I = I_0 \exp[(-D\gamma^2 G^2 \delta^2(\Delta\text{-}/3))]}$$

Equation S1. Calculation of *D*.

Where *I* is the intensity of a peak, *D* is the diffusivity, *G* is the pulse gradient strength, δ is the length of the gradient pulse, and Δ is the diffusion time.

Plotting *I vs*. *G* for a given peak gives a diffusion curve which is fit by TopSpin software to give *D*.

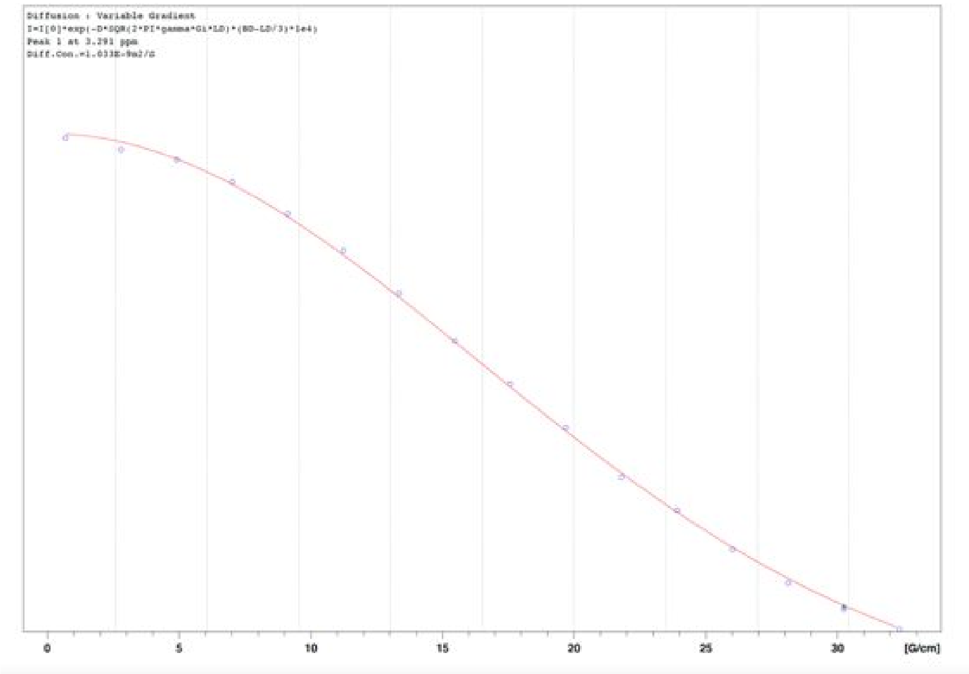

Figure S7. *TopSpin* display after fitting with *SimFit* (T1/T2 Analysis). The fitting curve for the peak at δ 3.29 of 4 in CD_2Cl_2 is shown.

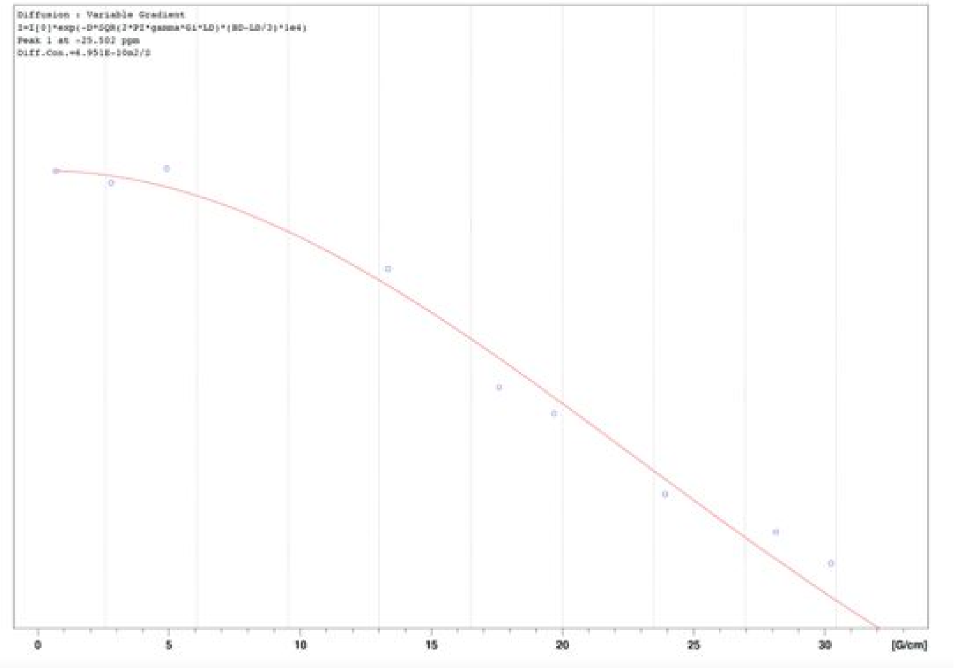

Figure S8. *TopSpin* display after fitting with *SimFit* (T1/T2 Analysis). The fitting curve for the peak at δ –25.47 ppm of 7 in CD_2Cl_2 is shown.

The hydrodynamic radii (rH) of complexes **4** and **7** were calculated the Equation S2.

$$\boldsymbol{r_H = kT / 6\eta\pi D}$$

Equation S2. Modified Stokes-Einstein equation.

Where: k = 1.38064852(79)·10^{-23} J·K^{-1}, T = temperature (K), η = viscosity of dichlomethane at 25 °C (0.413 cP).

10. Crystal structure determination of 4

Crystal Structure Determination. Single crystals of **4** suitable for the X-ray diffraction studies were grown by slow diffusion of hexane into a THF solution of the compound. X-ray diffraction data were collected at 100(2) K on a Bruker APEX SMART CCD diffractometer with graphite-monochromated Mo−Kα radiation (λ = 0.71073 Å) using 0.6° ω rotations. Intensities were integrated and corrected for absorption effects with SAINT–PLUS[8] and SADABS[9] programs, both included in APEX2 package. The structures were solved by the Patterson method with SHELXS-97[10] and refined by full matrix least-squares on F^2 with SHELXL-2014,[11] under WinGX.[12]

Crystal data and structure refinement for 4. $C_{28}H_{35}BF_4IrN_2OP$, **4**·THF, 725.56 g mol^{-1}, triclinic, P–1, a = 9.3189(5) Å, b = 11.4266(6) Å, c = 13.0194(7) Å, α = 95.5030(10)°, β = 92.4020(10)°, γ = 96.9640(10)°, V = 1367.77(13) Å^3, Z = 2, D_{calc} = 1.762 g·cm^{-3}, μ = 4.992 mm^{-1}, F(000) = 716, yellow prism, 0.270 x 0.200 x 0.065 mm^3, $\theta_{min}/\theta_{max}$ 1.805/28.299°, index ranges −12≤h≤12, −15≤k≤15, −17≤l≤17, reflections collected/independent 19786/6405 [R(int) = 0.0222], T_{min}/T_{max} 0.4543/0.2705, data/restraints/parameters 6405/0/344, GooF(F^2) 1.058, R_1 = 0.0206 [I>2σ(I)], wR_2 = 0.0491 (all data), largest diff. peak/hole 2.079/–1.002 e·Å^{-3}. CCDC deposit number: 2068679.

11. DFT energy data and optimized geometries

Energy data

Energy data for 3b-based reaction pathways

Table S1. Geometrical optimizations and thermochemistry corrections with def2-SVP basis set, E(DZ), and further refinement by single point calculations with def2-TZVP, E(TZ). G and G_{trans} are Gibbs free energy corrections with and without translational contribution to the energy, respectively. Absolute energies are in a.u. and relative energies (with respect to $\mathbf{A}_{PCP}$ = **3b**) are in kcal·mol^{-1}.

	E (DZ)	Corr. G	Corr. Gtrans	E (TZ)	G $(TZ)_{trans}$	ΔG $(TZ)_{trans}$
**A**$_{PCP}$	-2406.91178	0.65055	0.67236	-2408.90105	-2408.22869	0.0
**B**$_{PCP}$	-2284.66469	0.49974	0.52143	-2286.54278	-2286.02135	13.4
**TS-BC**$_{PCP}$	-2284.66140	0.49749	0.51918	-2286.54091	-2286.02174	13.2
**C**$_{PCP}$	-2284.68747	0.50151	0.52320	-2286.56972	-2286.04652	-2.4
**D**$_{PCP}$	-2474.33203	0.53061	0.55238	-2476.43814	-2475.88575	-7.4
**TS-DE**$_{PCP}$	-2474.30450	0.52529	0.54707	-2476.40965	-2475.86258	7.1
**TS-DE**$_{PCP}$**b**	-2284.65002	0.49402	0.51571	-2286.53562	-2286.01991	14.3
**E**$_{PCP}$	-2285.88403	0.52317	0.54486	-2287.76826	-2287.22339	-9.3
**TS-ED**$_{PCP}$	-2285.87403	0.51669	0.53838	-2287.75998	-2287.22160	-8.2
**D**$_{PCP}$ **+ products**	-2474.33203	0.53061	0.55238	-2476.43814	-2475.88575	-8.8
**COD**	-311.84503	0.14805	0.16703	-312.18413	-312.01710	
**FA**	-189.62895	0.00975	0.02753	-189.85868	-189.83116	
**CO_2**	-188.44724	-0.00902	0.00869	-188.67399	-188.66530	
**H_2**	-1.17410	-0.00161	0.01173	-1.17976	-1.16802	

Energy data for 4-based reaction pathways in formic acid

Table S2. Geometrical optimizations and thermochemistry corrections with def2-SVP basis set, E(DZ), and further refinement by single point calculations with def2-TZVP, E(TZ). G and G_{trans} are Gibbs free energy corrections with and without translational contribution to the energy, respectively. Absolute energies are in a.u. and relative energies (with respect to $\mathbf{A}_{PCO\text{-}FA}$ = **4**) are in kcal·mol^{-1}. Notice that the energy of COD, H_2, CO_2 and formic acid has already been included in Table S1.

	E (DZ)	Corr. G	Corr. G_{trans}	E (TZ)	G $(TZ)_{trans}$	ΔG $(TZ)_{trans}$
**A**$_{PCO\text{-}FA}$	-1717.61221	0.51296	0.53446	-1719.12085	-1718.58639	0.0
**TS-AB**$_{PCO\text{-}FA}$	-1907.22622	0.53875	0.56035	-1908.95916	-1908.39880	11.8
**B**$_{PCO\text{-}FA}$	-1907.24701	0.54344	0.56504	-1908.98344	-1908.41841	-0.5
**C**$_{PCO\text{-}FA}$	-1907.25509	0.54726	0.56886	-1908.99036	-1908.42150	-2.5
**TS-CD**$_{PCO\text{-}FA}$	-1907.23378	0.54324	0.56483	-1908.96908	-1908.40424	8.3
**D**$_{PCO\text{-}FA}$	-1907.24105	0.54692	0.56852	-1908.97652	-1908.40800	6.0
**E**$_{PCO\text{-}FA}$	-2096.90069	0.58105	0.60274	-2098.85372	-2098.25099	-1.4
**TS-EF**$_{PCO\text{-}FA}$	-2096.88323	0.57428	0.59597	-2098.83483	-2098.23886	6.2
**TS-EF**$_{PCO\text{-}FA}$**b**	-1907.23796	0.54453	0.56613	-1908.96853	-1908.40240	9.5
**TS-BC'**$_{PCO\text{-}FA}$	-2096.85731	0.56611	0.58780	-2098.81734	-2098.22954	12.0
**TS-BC'**$_{PCO\text{-}FA}$**b**	-1907.21020	0.53733	0.55893	-1908.94920	-1908.39027	17.1
**C'**$_{PCO\text{-}FA}$	-1718.79339	0.53241	0.55391	-1720.31329	-1719.75938	-4.5
**TS-C'F**$_{PCO\text{-}FA}$	-1718.78197	0.53047	0.55197	-1720.30019	-1719.74823	2.5
**F**$_{PCO\text{-}FA}$	-1908.44176	0.56581	0.58742	-1910.17392	-1909.58651	-1.9
**F-py**$_{PCO\text{-}FA}$	-1966.96372	0.61928	0.64094	-1968.74306	-1968.10211	-18.3
**TS-FG**$_{PCO\text{-}FA}$	-1718.77564	0.53135	0.55285	-1720.28756	-1719.73471	11.0
**G**$_{PCO\text{-}FA}$	-1785.00233	0.39974	0.42120	-1786.62045	-1786.19925	-5.7
**TS-GH**$_{PCO\text{-}FA}$	-1784.99976	0.39627	0.41773	-1786.61905	-1786.20132	-7.0
**H**$_{PCO\text{-}FA}$	-1785.02248	0.40001	0.42147	-1786.64459	-1786.22311	-20.7
**I**$_{PCO\text{-}FA}$	-1974.67896	0.42680	0.44836	-1976.52410	-1976.07574	-34.2
**TS-IJ**$_{PCO\text{-}FA}$	-1974.64848	0.42736	0.44892	-1976.49398	-1976.04505	-14.9
**TS-IJ**$_{PCO\text{-}FA}$**b**	-1784.98999	0.39156	0.41303	-1786.61647	-1786.20344	-8.3
**J**$_{PCO\text{-}FA}$	-1786.21781	0.41861	0.44008	-1787.83980	-1787.39972	-27.4
**TS-JI**$_{PCO\text{-}FA}$	-1786.20566	0.41414	0.43561	-1787.83091	-1787.39530	-24.7
**I**$_{PCO\text{-}FA}$ **+ products**	-1974.67896	0.42680	0.44836	-1976.52410	-1976.07574	-35.5
**cis-COE**	-313.07617	0.17178	0.19079	-313.41519	-313.22441	
**HCOO**$^-$	-189.14644	-0.00350	0.01424	-189.39352	-189.37928	
**py**	-248.12687	0.06140	0.07994	-248.40063	-248.32069	

Energy data for 4-based reaction pathways in dimethyl carbonate

Table S3. Geometrical optimizations and thermochemistry corrections with def2-SVP basis set, E(DZ), and further refinement by single point calculations with def2-TZVP, E(TZ). G and G_{trans} are Gibbs free energy corrections with and without translational contribution to the energy, respectively. Absolute energies are in a.u. and relative energies (with respect to $\mathbf{A}_{PCO\text{-}DMC}$ = **4**) are in kcal·mol^{-1}.

	E (DZ)	Corr. G	Corr. G_{trans}	E (TZ)	G $(TZ)_{trans}$	ΔG $(TZ)_{trans}$
$\mathbf{A}_{PCO\text{-}DMC}$	-1717.59440	0.51557	0.53707	-1719.10260	-1718.56553	0.0
$\mathbf{TS\text{-}AB}_{PCO\text{-}DMC}$	-1907.20549	0.54429	0.56589	-1908.93766	-1908.37177	13.8
$\mathbf{B}_{PCO\text{-}DMC}$	-1907.22613	0.54432	0.56592	-1908.96193	-1908.39601	-1.4
$\mathbf{C}_{PCO\text{-}DMC}$	-1907.23321	0.54784	0.56943	-1908.96733	-1908.39790	-2.6
$\mathbf{TS\text{-}CD}_{PCO\text{-}DMC}$	-1907.20943	0.54339	0.56499	-1908.94340	-1908.37841	9.6
$\mathbf{D}_{PCO\text{-}DMC}$	-1907.21624	0.54795	0.56955	-1908.95003	-1908.38048	8.3
$\mathbf{E}_{PCO\text{-}DMC}$	-2250.62026	0.63480	0.65657	-2252.74425	-2252.08768	-2.1
$\mathbf{TS\text{-}EF}_{PCO\text{-}DMC}$	-2250.60626	0.63632	0.65809	-2252.72828	-2252.07018	8.8
$\mathbf{TS\text{-}EF}_{PCO\text{-}DMC}\mathbf{b}$	-1907.20663	0.54494	0.56654	-1908.93799	-1908.37145	14.0
$\mathbf{F}_{PCO\text{-}DMC}$	-2062.17002	0.61966	0.64135	-2064.07423	-2063.43288	-8.1
$\mathbf{TS\text{-}FG}_{PCO\text{-}DMC}$	-1718.75664	0.53147	0.55297	-1720.26799	-1719.71502	9.1
$\mathbf{G}_{PCO\text{-}DMC}$	-1938.72718	0.45440	0.47596	-1940.51806	-1940.04210	-11.0
$\mathbf{TS\text{-}GH}_{PCO\text{-}DMC}$	-1938.72646	0.45148	0.47304	-1940.51817	-1940.04513	-12.9
$\mathbf{H}_{PCO\text{-}DMC}$	-1938.74524	0.45654	0.47810	-1940.53969	-1940.06159	-23.3
$\mathbf{I}_{PCO\text{-}DMC}$	-2282.14815	0.54238	0.56412	-2284.33771	-2283.77359	-36.7
$\mathbf{TS\text{-}IJ}_{PCO\text{-}DMC}$	-2282.12212	0.54251	0.56425	-2284.31054	-2283.74629	-19.6
$\mathbf{TS\text{-}IJ}_{PCO\text{-}DMC}\mathbf{b}$	-1938.72012	0.45171	0.47327	-1940.51825	-1940.04497	-12.8
$\mathbf{J'}_{PCO\text{-}DMC}$	-2093.67368	0.52493	0.54658	-2095.64301	-2095.09643	-28.6
$\mathbf{J}_{PCO\text{-}DMC}$	-1939.94982	0.47084	0.49240	-1941.74342	-1941.25102	-39.2
$\mathbf{TS\text{-}JI}_{PCO\text{-}DMC}$	-1939.93739	0.47310	0.49466	-1941.73350	-1941.23884	-31.6
$\mathbf{I}_{PCO\text{-}DMC}$ + **products**	-2282.14815	0.54238	0.56412	-2284.33771	-2283.77359	-39.2
**DMC**	-343.37017	0.06406	0.08278	-343.77332	-343.69054	
**COD**	-311.84413	0.14818	0.16716	-312.18302	-312.01587	
**cis-COE**	-313.07578	0.17196	0.19097	-313.41468	-313.22372	
**FA**	-189.62634	0.00982	0.02759	-189.85578	-189.82820	
$\mathbf{CO_2}$	-188.44617	-0.00898	0.00873	-188.67296	-188.66423	
$\mathbf{H_2}$	-1.17403	-0.00160	0.01174	-1.17969	-1.16795	

Optimized geometries

The graphical representations of the intermediates and transition states involved in the computed reaction pathways are provided below. Notice that non-relevant hydrogen atoms have been omitted for clarity.

Optimized geometries for 3b-based reaction pathways

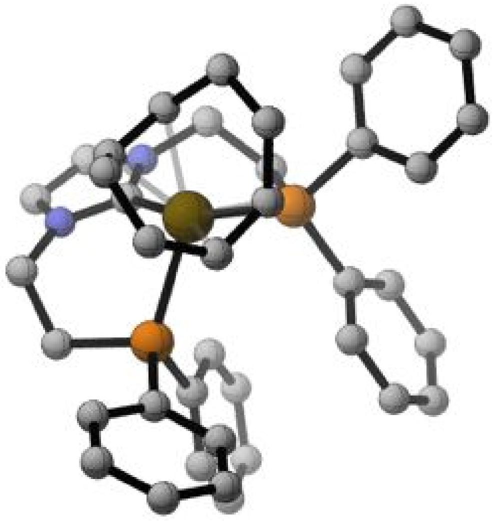

A$_{PCP}$

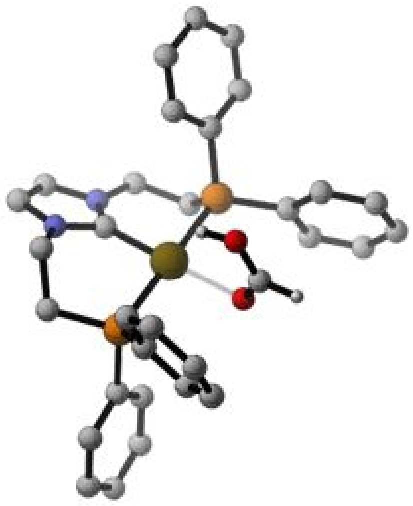

B$_{PCP}$

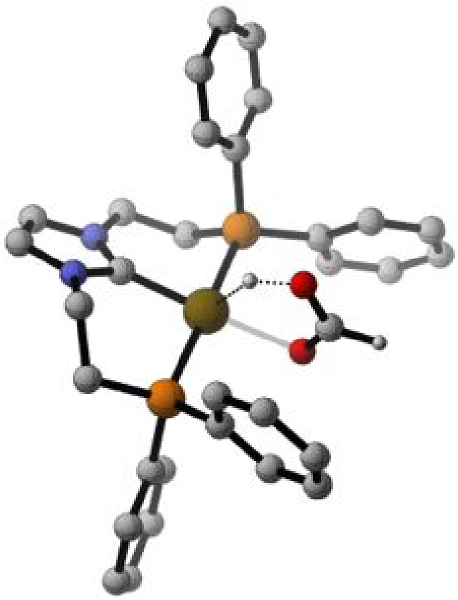

TS-BC$_{PCP}$

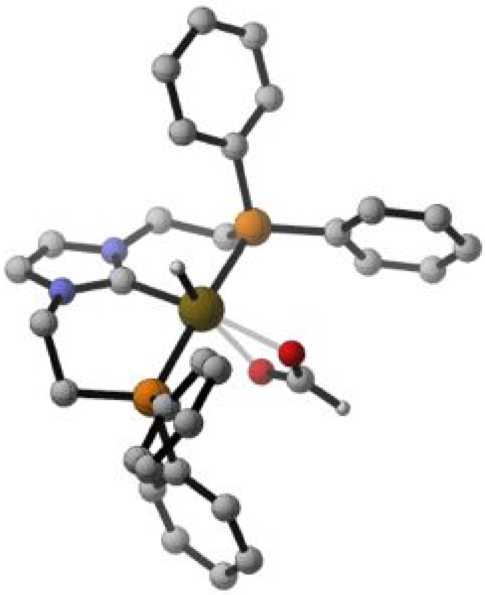

C$_{PCP}$

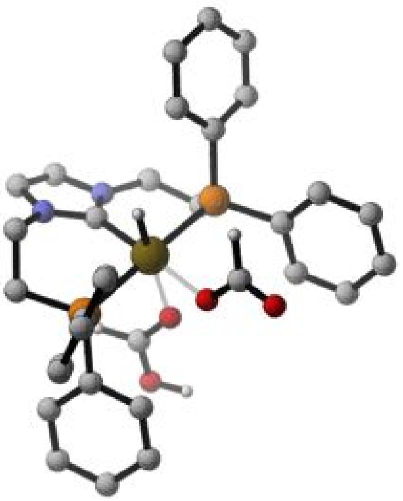

D$_{PCP}$

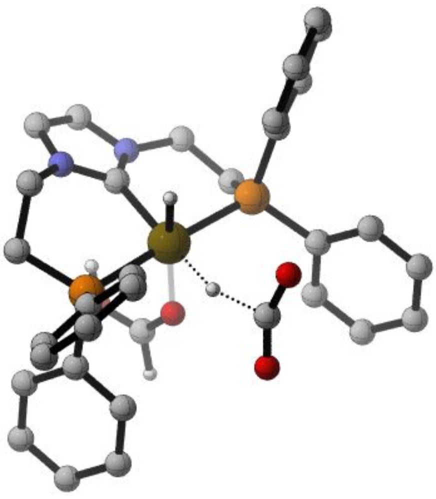

TS-DE$_{PCP}$

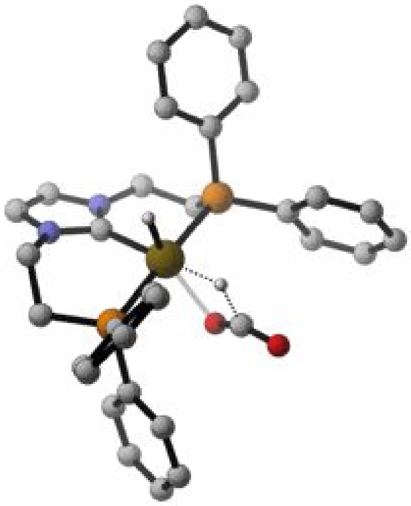

TS-DE$_{PCP}$**b**

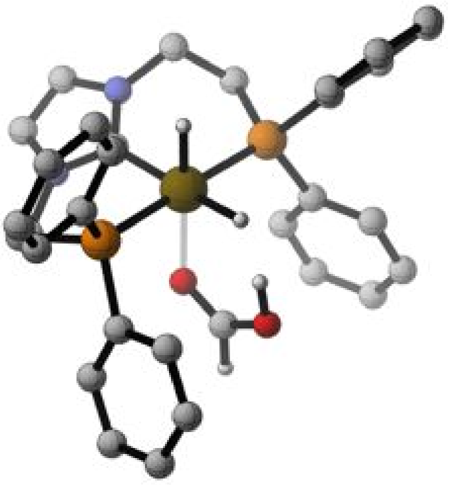

E$_{PCP}$

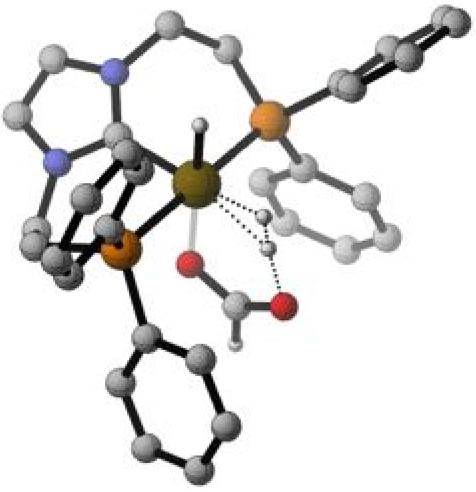

TS-ED$_{PCP}$

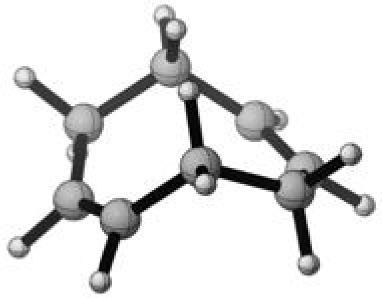

COD

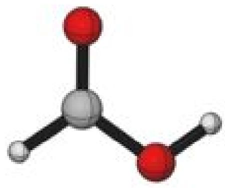

Formic acid

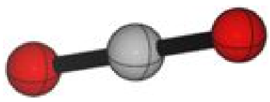

CO_2

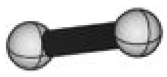

H_2

Optimized geometries for 4-based reaction pathways in formic acid

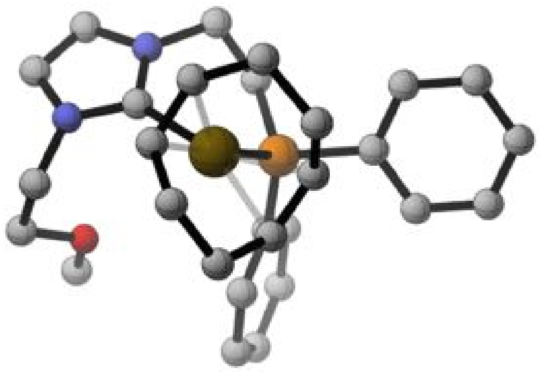

A$_{PCO\text{-}FA}$

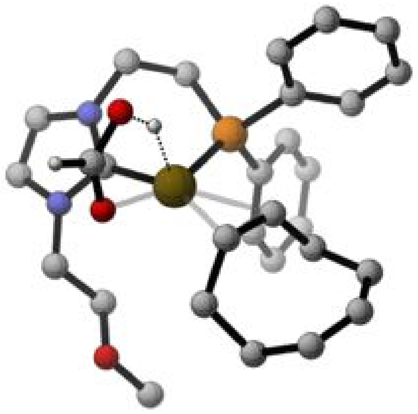

TS-AB$_{PCO\text{-}FA}$

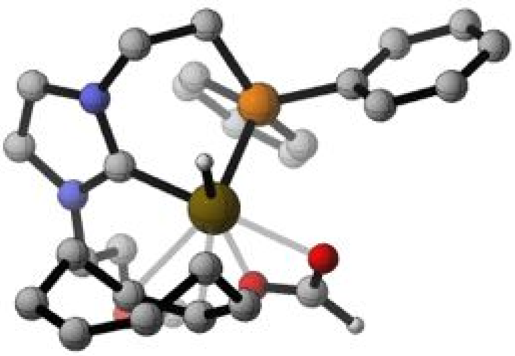

B$_{PCO\text{-}FA}$

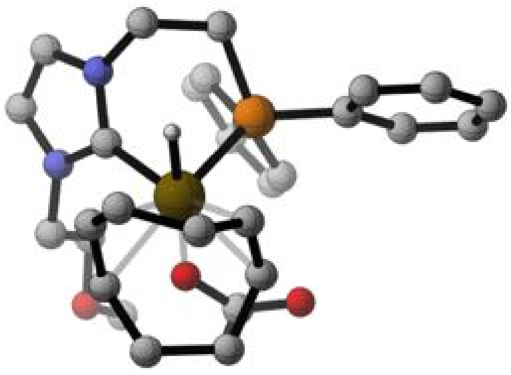

C$_{PCO\text{-}FA}$

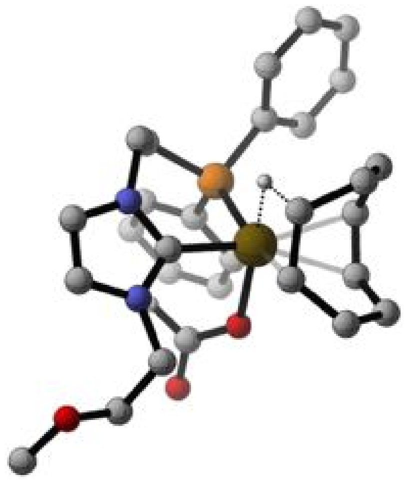

TS-CD*PCO-FA*

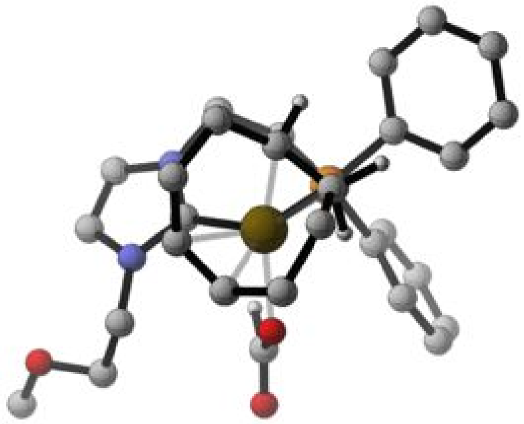

D*PCO-FA*

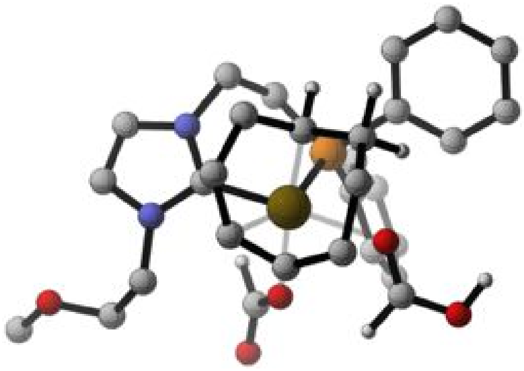

E*PCO-FA*

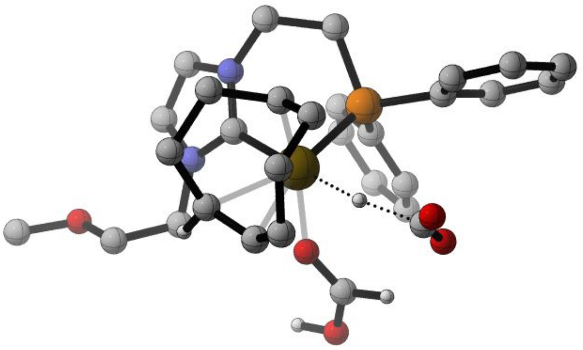

TS-EF*PCO-FA*

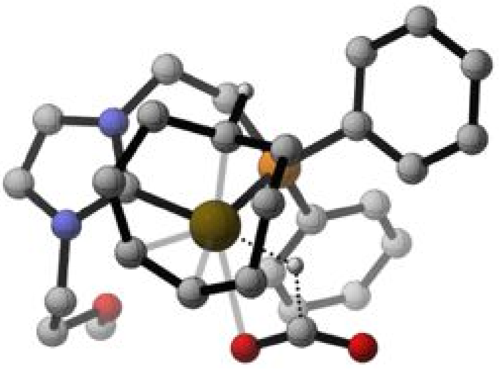

TS-EF*PCO-FA*b

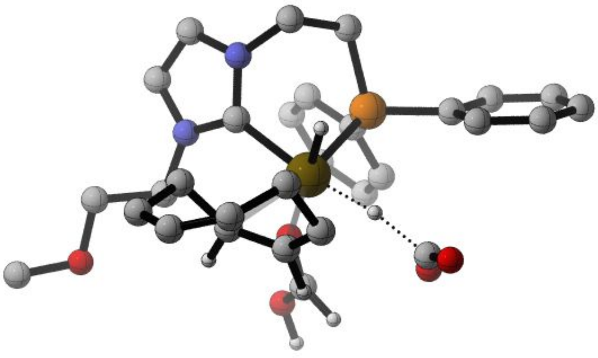

TS-BC'*PCO-FA*

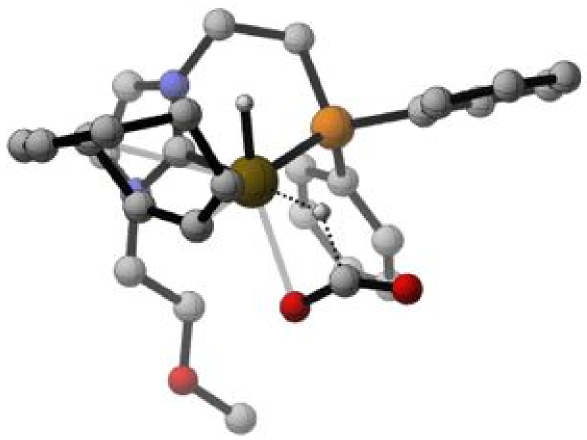

TS-BC’$_{PCO\text{-}FA}$b

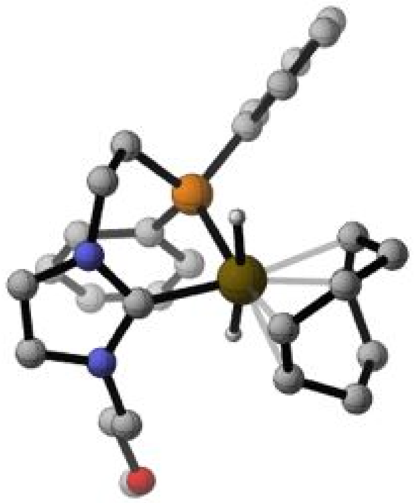

C’$_{PCO\text{-}FA}$

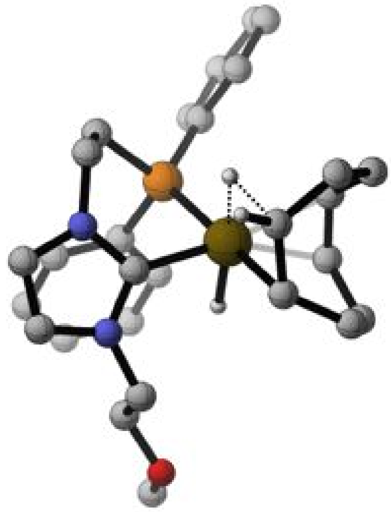

TS-C’F$_{PCO\text{-}FA}$

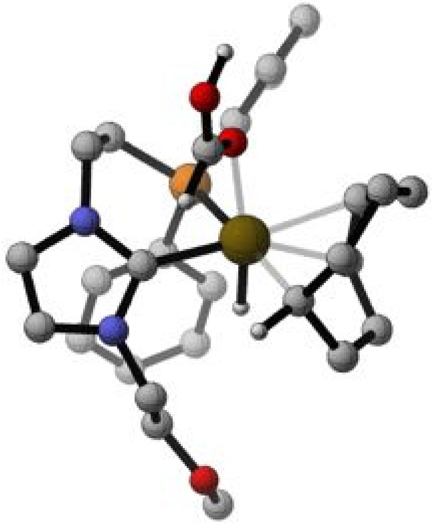

F$_{PCO\text{-}FA}$

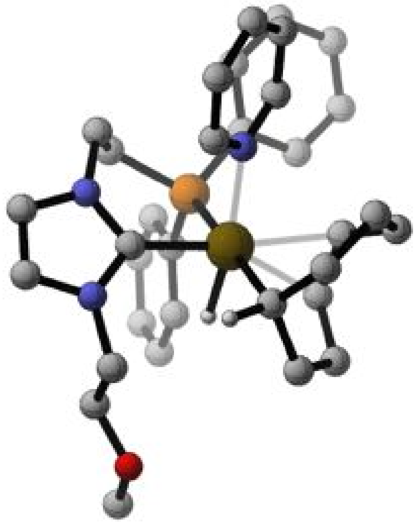

F-py$_{PCO\text{-}FA}$

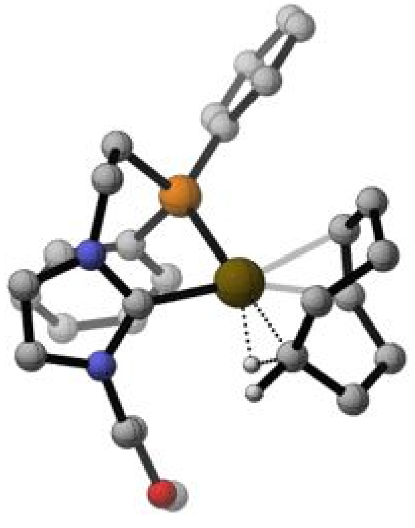

TS-FG$_{PCO\text{-}FA}$

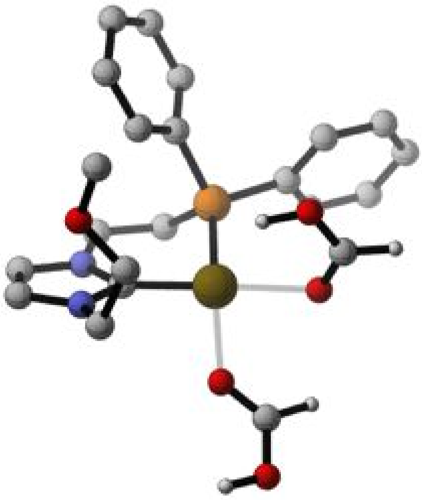

G*PCO-FA*

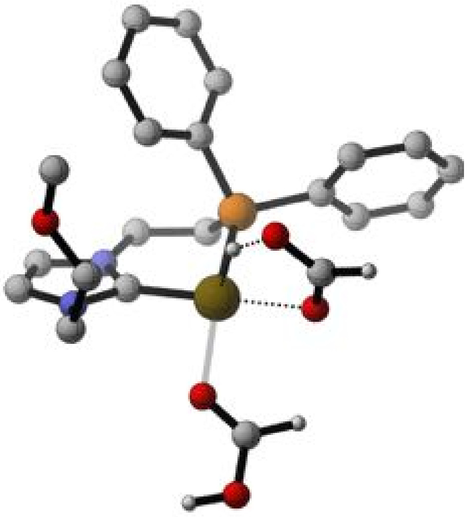

TS-GH*PCO-FA*

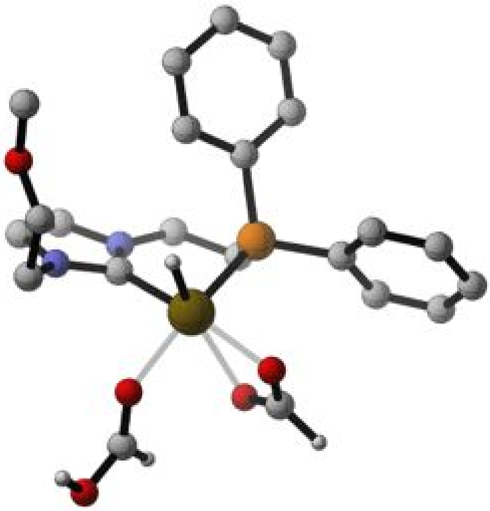

H*PCO-FA*

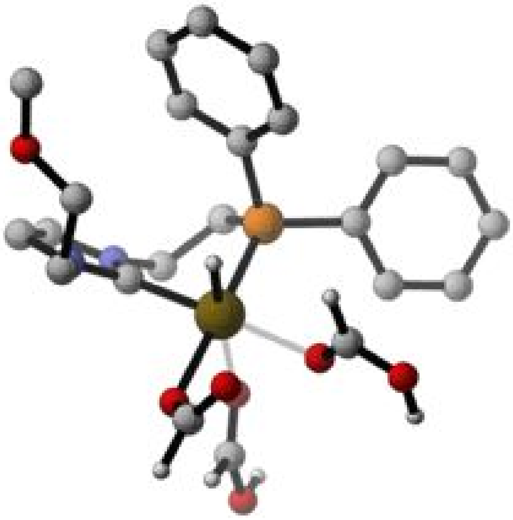

I*PCO-FA*

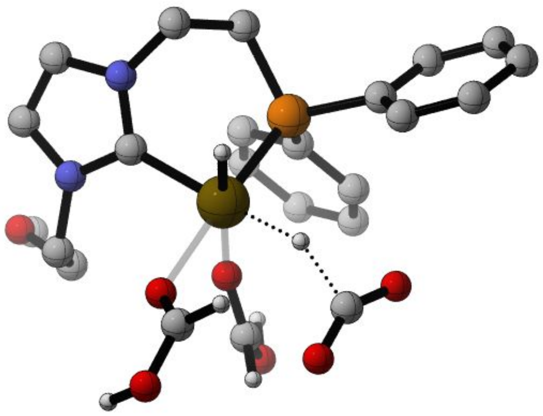

TS-IJ*PCO-FA*

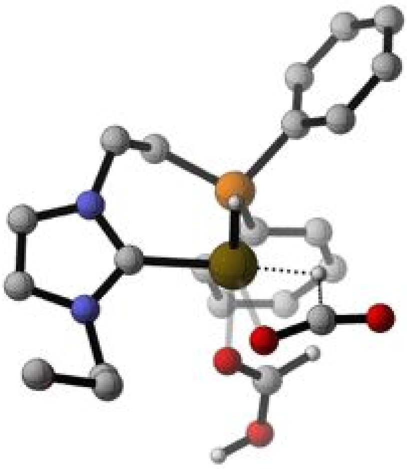

TS-IJ*PCO-FA***b**

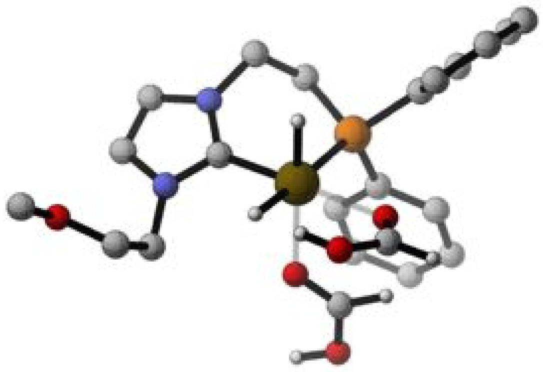

$\mathbf{J}_{PCO\text{-}FA}$

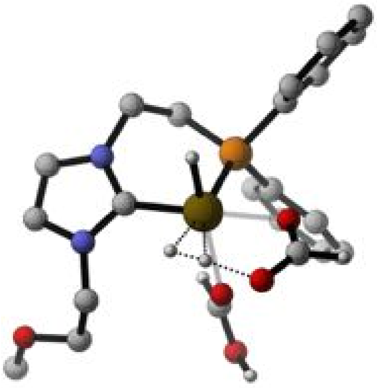

$\mathbf{TS\text{-}JI}_{PCO\text{-}FA}$

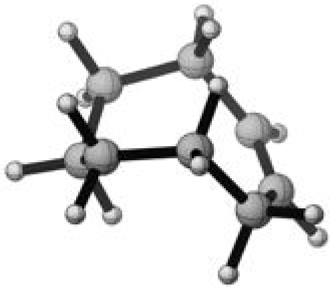

cis-COE

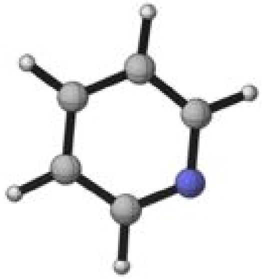

py

Optimized geometries for 4-based reaction pathways in dimethyl carbonate

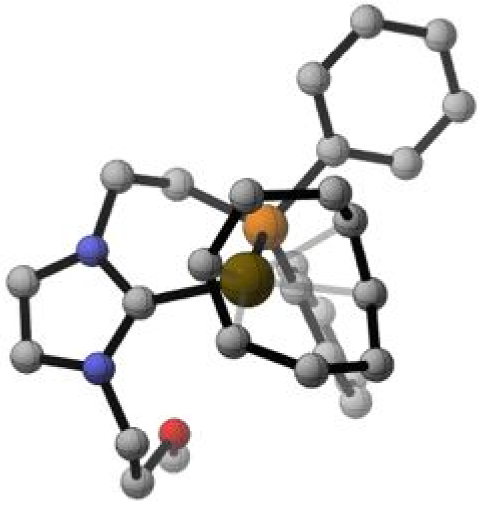

$\mathbf{A}_{PCO\text{-}DMC}$

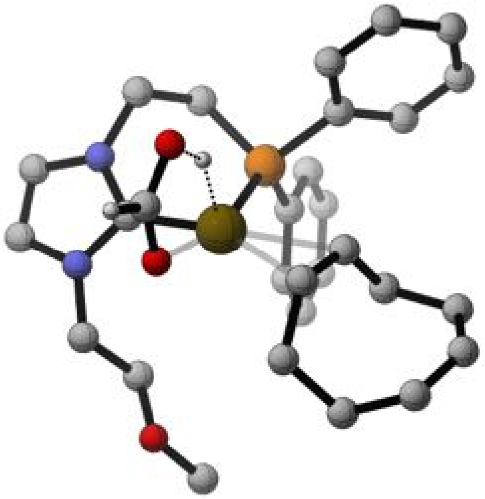

$\mathbf{TS\text{-}AB}_{PCO\text{-}DMC}$

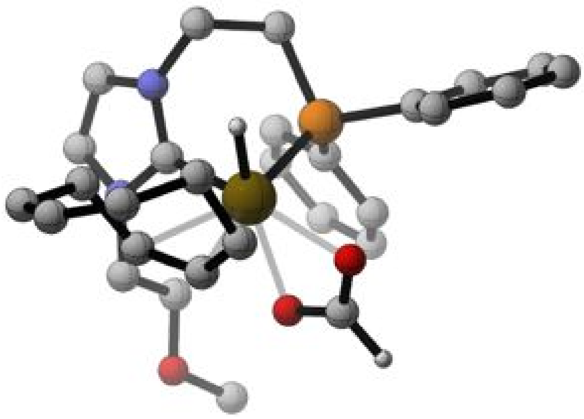

B*PCO-DMC*

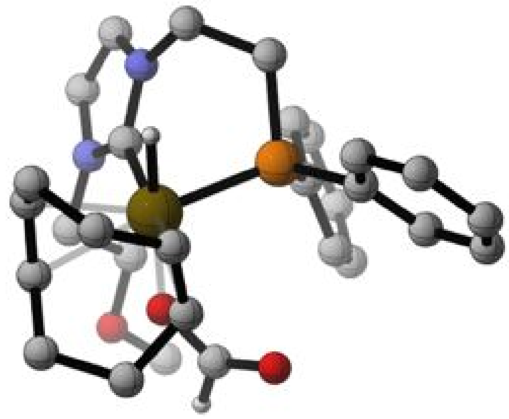

C*PCO-DMC*

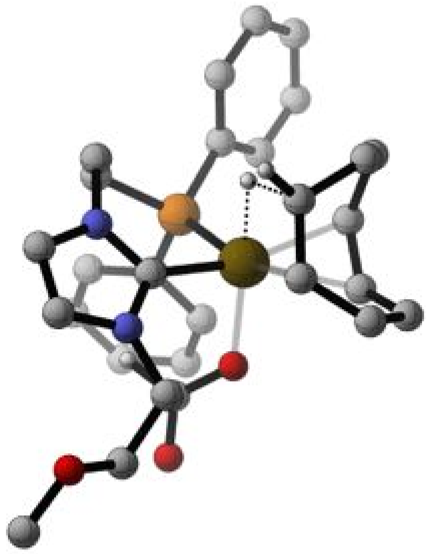

TS-CD*PCO-DMC*

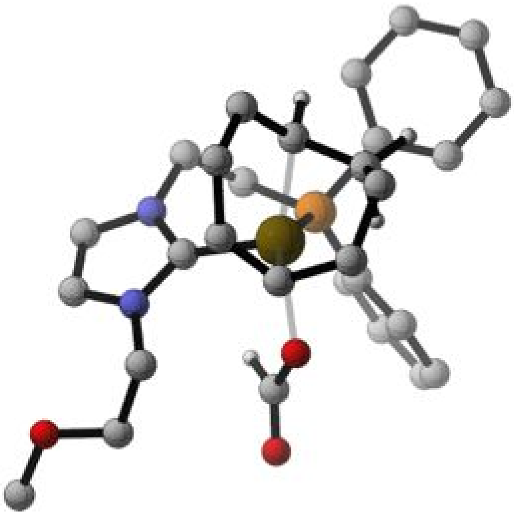

D*PCO-DMC*

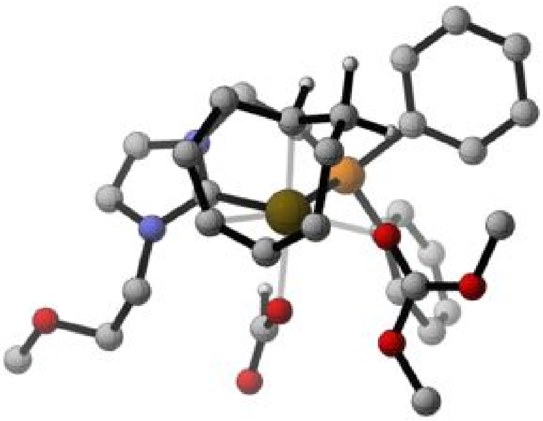

E*PCO-DMC*

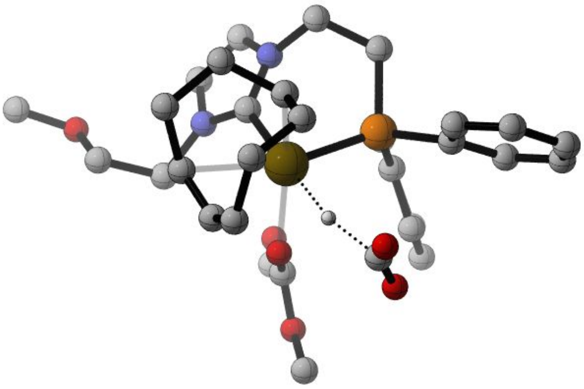

TS-EF*PCO-DMC*

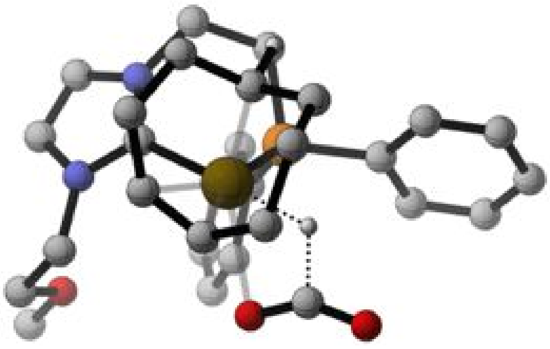

TS-EF$_{PCO\text{-}DMC}$**b**

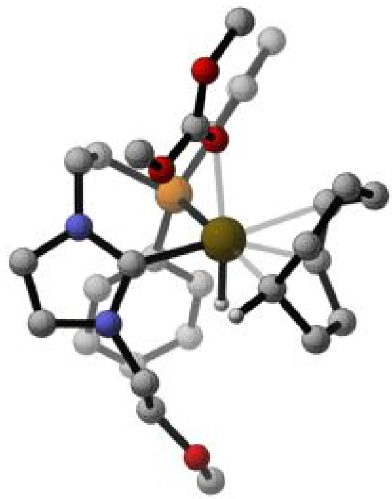

F$_{PCO\text{-}DMC}$

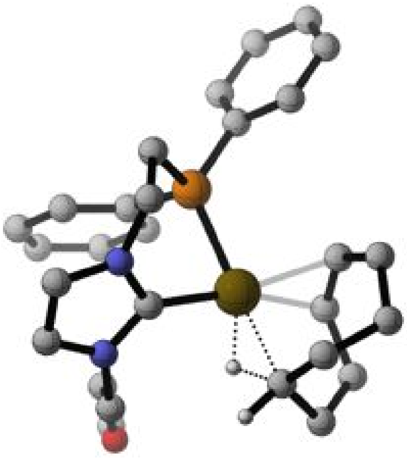

TS-FG$_{PCO\text{-}DMC}$

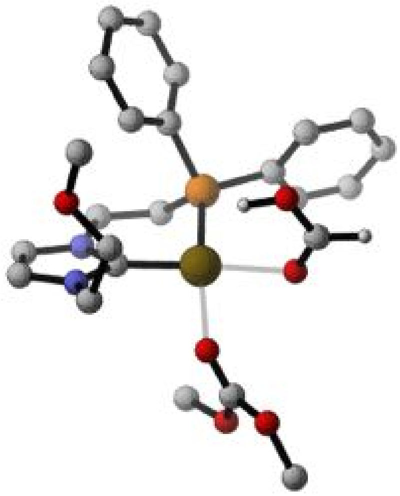

G$_{PCO\text{-}DMC}$

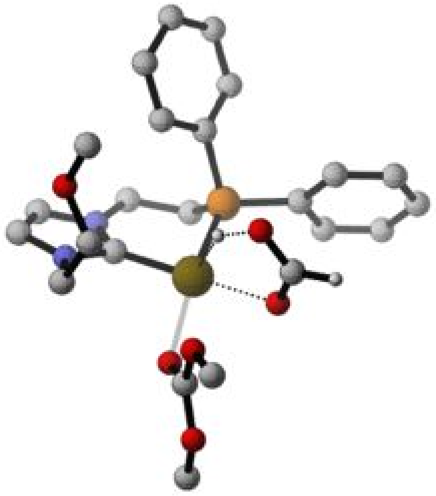

TS-GH$_{PCO\text{-}DMC}$

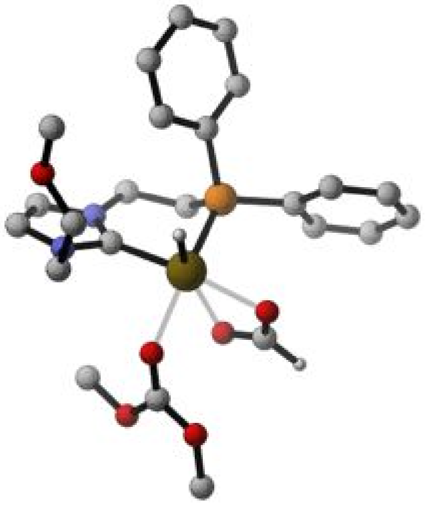

H$_{PCO\text{-}DMC}$

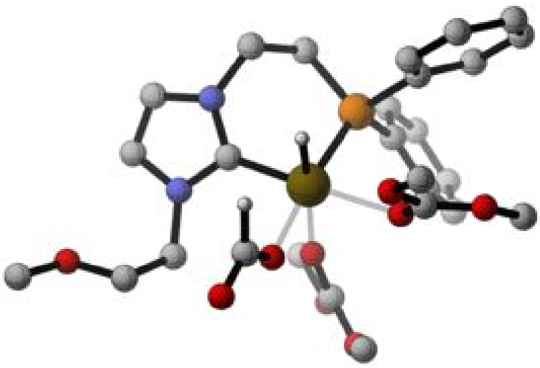

I*PCO-DMC*

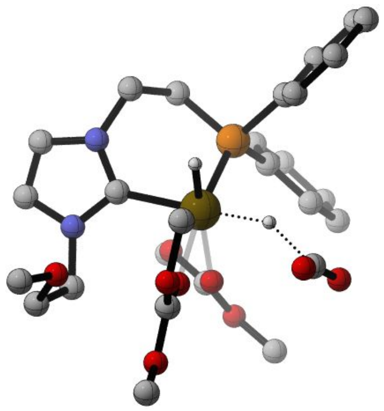

TS-IJ*PCO-DMC*

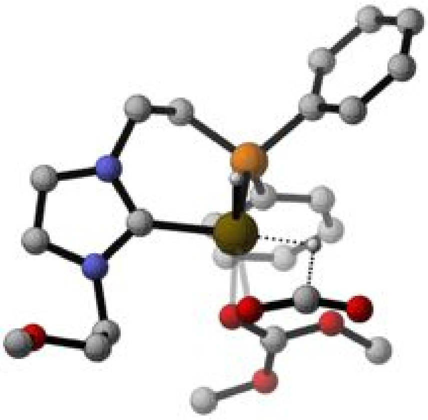

TS-IJ*PCO-DMC*b

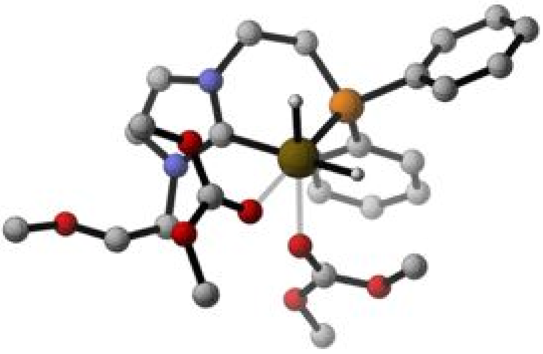

J'*PCO-DMC*

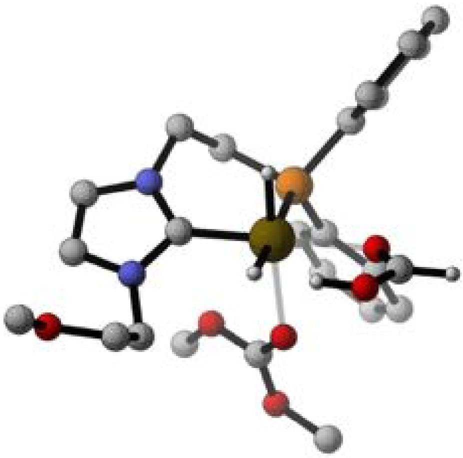

J*PCO-DMC*

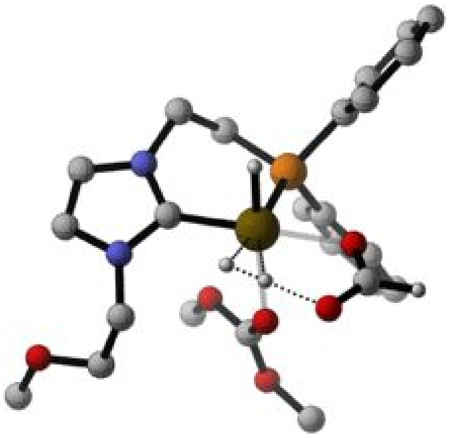

TS-JI*PCO-DMC*

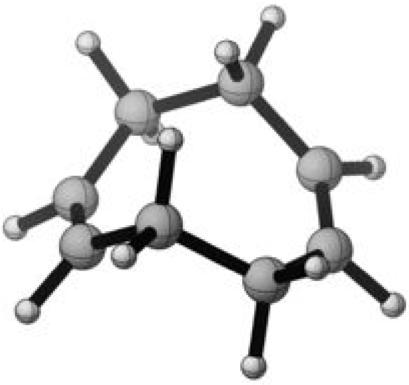

COD

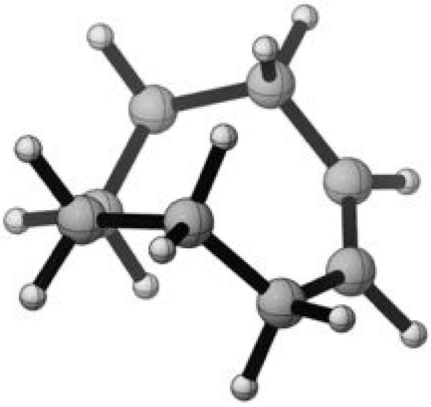

cis-COE

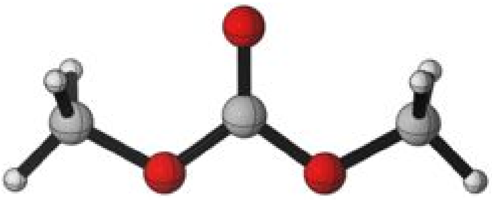

Dimethyl carbonate

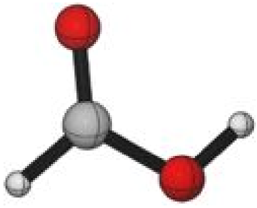

Formic acid

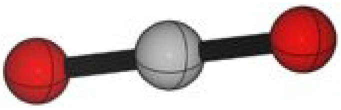

CO_2

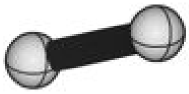

H_2

Cartesian coordinates

Cartesian coordinates (xyz format) for 3b-based reaction pathways

A*PCP*

6 0.157234 -0.976612 2.239988
6 2.845949 -2.654886 -0.615144
6 0.487187 -3.201984 0.360102
6 -3.093501 -0.594036 -1.172486
6 1.145014 -1.262593 -1.959888
6 0.867777 2.582678 -0.414154
6 3.174851 0.971275 -0.686209
6 -2.402632 1.495629 0.712769
6 -0.252532 4.921118 -1.485572
6 -0.659961 3.670559 -1.959191
6 -2.057121 -2.120031 2.429691
6 -3.065682 0.281102 -2.274538
6 2.354568 -2.184173 -1.996846
6 3.243384 1.425546 -2.014395
6 -2.914668 -1.089050 1.711082
6 -0.629041 -1.307476 4.339765
6 -1.815282 3.433135 2.062945
6 0.552598 -0.644601 4.446119
6 -0.174324 -1.693340 -1.956573
6 -4.530931 -1.187725 -3.524462
6 -0.103676 2.509291 -1.419873
6 -0.636639 -3.132379 -1.924387
6 -4.558627 -2.068308 -2.440864
6 -1.563156 2.128047 1.640959
6 -3.843143 -1.776290 -1.275463
6 -3.524723 2.188409 0.234820
6 0.081343 -3.996539 -0.879845
6 -3.780762 -0.010492 -3.436870
6 -2.918368 4.128814 1.558383
6 -3.773969 3.500240 0.649734
6 1.787993 -2.576615 0.467014
1 3.690173 -2.025500 -0.306548
1 3.245580 -3.684694 -0.683630
1 0.095667 -3.607811 1.297773
1 4.461407 1.678876 -3.775474
1 2.105334 -3.050016 -2.630727
1 3.169799 -1.652850 -2.508876
1 2.367356 1.873594 -2.486087
1 -1.141880 3.910315 2.778262
1 -1.325162 -1.653260 5.097817
1 -3.211146 -0.296905 2.412120
1 -3.838014 -1.558901 1.348657
1 -1.760593 -2.924378 1.746406
1 -2.490533 1.205762 -2.225455
1 -2.612222 -2.565738 3.264950
1 -0.694716 5.832288 -1.894724
1 -1.420311 3.599067 -2.740266
1 1.314011 -0.254907 -2.338647
1 1.103372 -0.299774 5.315825
1 -0.910488 -0.991284 -2.348808
1 -0.430124 1.528744 -1.768861
1 -5.090052 -1.417086 -4.434217
1 -5.137077 -2.993003 -2.498663
1 -0.702589 1.591730 2.038319
1 -1.712943 -3.123555 -1.705931
1 -0.537583 -3.563146 -2.939387
1 -3.871170 -2.491929 -0.452795
1 -4.212565 1.711824 -0.463472
1 0.971251 -4.477088 -1.319258
1 -0.584782 -4.821051 -0.583652
1 -3.750073 0.686528 -4.277209
1 -3.112068 5.155956 1.875632
1 -4.645587 4.031352 0.260033
1 2.220770 -2.573694 1.474784
77 0.306620 -1.035552 0.210150
7 1.020806 -0.453662 3.152516
7 -0.849628 -1.511037 2.985258

15 1.569890 1.012718 0.226769
15 -2.023797 -0.260284 0.281852
6 1.288086 3.840661 0.042887
6 4.332674 0.434529 -0.100290
6 0.722406 5.003286 -0.485484
6 4.430709 1.323134 -2.743043
6 2.284880 0.207329 2.839038
6 2.111211 1.440162 1.953305
1 2.055805 3.924467 0.814093
6 5.522584 0.340869 -0.826410
1 4.323348 0.075653 0.928729
1 1.045239 5.978969 -0.114982
6 5.574433 0.776712 -2.153355
1 2.736923 0.500483 3.794764
1 2.952736 -0.530474 2.374117
1 3.059830 1.993198 1.916264
1 1.361862 2.110744 2.396889
1 6.411494 -0.079121 -0.350287
1 6.503494 0.698155 -2.722309

B$_{PCP}$

6 -0.303160 -2.111953 -0.253445
6 3.015798 0.569605 1.517439
6 -2.665236 1.879378 -0.999999
6 -3.469923 -0.457182 0.502217
6 3.437160 -0.288064 -1.207978
6 -3.240230 4.583626 -1.472212
6 -2.967292 3.727498 -2.542489
6 1.421076 -2.888209 1.408450
6 3.473462 1.848740 1.160762
6 -3.110028 -1.208200 1.630689
6 2.675405 -2.258826 0.803121
6 -0.093065 -4.360347 0.065233
6 3.710127 0.195173 -3.570995
6 -1.086935 -4.170627 -0.836604
6 3.784251 2.407079 3.498430
6 -2.677507 2.379147 -2.310269
6 3.330066 1.134965 3.859547
6 2.881057 0.018521 -2.458569
6 2.942389 0.222126 2.875854
6 4.830737 -0.415074 -1.078598
6 3.854991 2.761249 2.147373
6 5.095087 0.065083 -3.438165
6 5.654752 -0.240626 -2.191286
1 -3.813000 -2.321429 3.343013
1 -2.052342 -1.371843 1.848229
1 3.271847 0.436113 -4.542073
1 0.321318 -5.269881 0.489628
1 3.002941 -2.843168 -0.069042
1 3.496385 -2.259917 1.534052
1 1.013324 -2.263064 2.216212
1 3.527706 2.135185 0.108847
1 1.663843 -3.868356 1.838512
1 -3.465502 5.636325 -1.657084
1 -2.980059 4.108257 -3.566225
1 -1.720893 -4.881862 -1.357446
1 -2.460557 1.726166 -3.157258
1 4.082664 3.121620 4.268759
1 3.272381 0.850934 4.912675
1 1.796670 0.119070 -2.552247
1 2.575696 -0.759659 3.180481
1 5.274070 -0.643065 -0.106177
1 4.210268 3.752913 1.858116
1 5.742916 0.203886 -4.306854
1 6.737520 -0.340419 -2.086658
77 0.050711 -0.169494 -0.118822
7 -1.211730 -2.798868 -1.027482
7 0.371246 -3.103094 0.416661
15 -2.167732 0.168573 -0.618676
15 2.331685 -0.534230 0.225644
6 -2.936468 2.745042 0.073796
6 -4.828063 -0.235757 0.217857
6 -3.223831 4.089845 -0.162605
6 -4.098967 -1.737982 2.465004

6 -2.330748 -2.314153 -1.855472
6 -2.391556 -0.822053 -2.156271
1 -2.921497 2.364948 1.098271
6 -5.812603 -0.765136 1.053524
1 -5.116114 0.355675 -0.654670
1 -3.435366 4.754971 0.677648
6 -5.448697 -1.517568 2.177059
1 -2.294119 -2.869391 -2.803862
1 -3.257691 -2.611063 -1.340983
1 -3.361613 -0.616586 -2.634008
1 -1.592511 -0.521506 -2.849854
1 -6.867330 -0.590120 0.829531
1 -6.221282 -1.930017 2.830121
6 0.275801 2.469460 1.184459
1 0.440378 3.536284 1.399786
8 0.440655 2.000795 0.057060
8 -0.108129 1.758794 2.204608
1 -0.197980 0.803194 1.842608

TS-BC$_{PCP}$

6 -0.301981 -2.089888 -0.250587
6 3.053955 0.604536 1.486904
6 -2.702560 1.887052 -0.983925
6 -3.458443 -0.478191 0.502362
6 3.433728 -0.291660 -1.233176
6 -3.346162 4.578840 -1.428631
6 -3.113848 3.726002 -2.510862
6 1.410279 -2.838908 1.430800
6 3.509769 1.878177 1.110405
6 -3.082461 -1.248726 1.612400
6 2.666833 -2.226896 0.811834
6 -0.109262 -4.329448 0.108859
6 3.701686 0.175630 -3.599769
6 -1.093097 -4.148810 -0.806741
6 3.887804 2.445011 3.435652
6 -2.790440 2.382836 -2.292651
6 3.432114 1.179230 3.816116
6 2.876255 0.024716 -2.480798
6 3.011724 0.264308 2.847890
6 4.824703 -0.454408 -1.114168
6 3.925330 2.792048 2.081242
6 5.083644 0.010248 -3.477013
6 5.644582 -0.305532 -2.233345
1 -3.762080 -2.402610 3.306346
1 -2.023142 -1.409501 1.820921
1 3.262205 0.424442 -4.568226
1 0.294314 -5.234135 0.553530
1 2.984795 -2.824586 -0.054756
1 3.491309 -2.228564 1.538628
1 1.004052 -2.196737 2.225694
1 3.536492 2.158582 0.055889
1 1.649399 -3.811260 1.879650
1 -3.598895 5.627134 -1.602718
1 -3.185556 4.104631 -3.532907
1 -1.726771 -4.864290 -1.321997
1 -2.608205 1.731794 -3.149196
1 4.212921 3.160915 4.193880
1 3.398901 0.901710 4.872011
1 1.795695 0.155666 -2.575871
1 2.643966 -0.712213 3.167590
1 5.269299 -0.691161 -0.144483
1 4.280298 3.779509 1.777669
1 5.728026 0.129242 -4.351113
1 6.725049 -0.433215 -2.136117
77 0.052917 -0.134683 -0.125271
7 -1.205903 -2.779955 -1.021580
7 0.360312 -3.069523 0.441929
15 -2.177647 0.183537 -0.619043
15 2.344399 -0.504051 0.216657
6 -2.930494 2.749910 0.102308
6 -4.820573 -0.261842 0.233505
6 -3.253370 4.088428 -0.120712
6 -4.060298 -1.803987 2.442846
6 -2.304599 -2.296061 -1.877310

6 -2.368097 -0.800998 -2.164561
1 -2.853348 2.371801 1.124752
6 -5.793681 -0.817399 1.065444
1 -5.120448 0.345631 -0.623739
1 -3.431727 4.751963 0.728398
6 -5.414157 -1.589844 2.169891
1 -2.237812 -2.841525 -2.829655
1 -3.241975 -2.604624 -1.389999
1 -3.330484 -0.598412 -2.658302
1 -1.560411 -0.488291 -2.843622
1 -6.851540 -0.646902 0.853326
1 -6.177761 -2.022807 2.820178
6 0.220026 2.318419 1.354761
1 0.363009 3.362148 1.685519
8 0.418039 2.021706 0.156511
8 -0.148452 1.473086 2.235681
1 -0.176445 0.435202 1.546556

C_{PCP}

6 -0.367712 -1.919218 -0.964360
6 3.029981 -0.404484 1.773135
6 -2.622634 2.130736 -0.016673
6 -3.503641 -0.571744 0.606260
6 3.340631 0.402238 -0.999843
6 -3.028976 4.852794 0.505693
6 -3.020993 3.931716 1.559567
6 1.495636 -3.269188 0.066351
6 2.214630 0.014125 2.834272
6 -4.846519 -0.157338 0.567357
6 2.686537 -2.394549 -0.322043
6 -0.129826 -4.068434 -1.657374
6 4.058522 0.983738 -3.244198
6 -1.184205 -3.540508 -2.325037
6 4.117904 -0.055190 4.331192
6 -2.814291 2.576504 1.302015
6 4.936737 -0.470413 3.274061
6 3.396602 0.116495 -2.373775
6 4.396977 -0.643896 1.999569
6 3.931075 1.579185 -0.514034
6 2.759871 0.186884 4.110146
6 4.654597 2.151028 -2.754775
6 4.586001 2.448144 -1.390693
1 -6.872455 -0.592708 1.166699
1 -5.122045 0.764253 0.049751
1 4.103215 0.749878 -4.310145
1 0.320665 -5.055382 -1.698699
1 2.880707 -2.480963 -1.400047
1 3.590106 -2.722660 0.210068
1 1.178436 -3.073655 1.101477
1 1.155736 0.205674 2.658764
1 1.770561 -4.328864 0.002091
1 -3.188435 5.914117 0.708763
1 -3.173181 4.271215 2.586428
1 -1.851703 -3.976778 -3.061867
1 -2.802569 1.861875 2.128332
1 4.542447 0.080973 5.328474
1 5.999409 -0.657308 3.443401
1 2.913314 -0.776601 -2.775641
1 5.045887 -0.955935 1.177715
1 3.876732 1.821322 0.548321
1 2.119319 0.512947 4.932451
1 5.168813 2.830529 -3.438112
1 5.044727 3.360836 -1.003888
77 0.025323 -0.175010 -0.061746
7 -1.326121 -2.226169 -1.894212
7 0.352258 -3.072076 -0.823381
15 -2.232515 0.377444 -0.289876
15 2.328856 -0.636622 0.104156
6 -2.626643 3.056198 -1.070492
6 -3.159571 -1.752485 1.280799
6 -2.831384 4.413638 -0.806082
6 -5.830838 -0.919788 1.197220
6 -2.511654 -1.472500 -2.345299
6 -2.537069 0.022981 -2.068098

1 -2.462456 2.731458 -2.098204
6 -4.149943 -2.514166 1.907537
1 -2.117483 -2.072677 1.317782
1 -2.836264 5.129162 -1.631204
6 -5.483661 -2.099151 1.866802
1 -2.593000 -1.633064 -3.429154
1 -3.388893 -1.946335 -1.878407
1 -3.519237 0.405453 -2.383525
1 -1.756786 0.551603 -2.633878
1 -3.875780 -3.431866 2.432276
1 -6.256121 -2.693563 2.359993
1 -0.185382 -0.997151 1.247582
8 0.358506 1.572087 -1.609744
6 0.610196 2.324573 -0.635937
1 0.868661 3.388660 -0.800942
8 0.572377 1.882810 0.553132

D$_{PCP}$

6 -0.414900 -2.090346 -0.713704
6 2.836449 -0.258420 2.066230
6 -2.653917 2.054697 -0.414675
6 -3.582873 -0.558632 0.531656
6 3.386276 0.146493 -0.744619
6 -2.990128 4.833451 -0.289542
6 -3.652479 4.007097 0.621604
6 1.322626 -3.302038 0.651201
6 1.937839 -0.161689 3.139095
6 -4.931165 -0.400603 0.162564
6 2.566333 -2.514256 0.240717
6 -0.210083 -4.328883 -1.040939
6 5.121744 0.189618 -2.440851
6 -1.208019 -3.889556 -1.846460
6 3.768554 0.358002 4.637712
6 -3.484400 2.620740 0.563627
6 4.668599 0.264431 3.570481
6 4.322859 -0.526566 -1.542806
6 4.206723 -0.041869 2.289161
6 3.261261 1.545395 -0.847991
6 2.404714 0.143559 4.420295
6 4.991088 1.575692 -2.545377
6 4.061621 2.251658 -1.745034
1 -6.977760 -0.940864 0.576503
1 -5.202476 0.250119 -0.671845
1 5.846747 -0.342872 -3.060133
1 0.206590 -5.321164 -0.897145
1 2.816391 -2.754302 -0.802056
1 3.422616 -2.791866 0.871185
1 0.927201 -2.953231 1.616535
1 0.871738 -0.316424 2.971037
1 1.574529 -4.363235 0.764762
1 -3.119908 5.916923 -0.240295
1 -4.300965 4.440769 1.386038
1 -1.848334 -4.422157 -2.542907
1 -3.996984 1.983611 1.285836
1 4.131336 0.602667 5.638639
1 5.734923 0.434457 3.735325
1 4.443052 -1.608246 -1.475138
1 4.913969 -0.106318 1.459659
1 2.509461 2.062296 -0.246937
1 1.697439 0.220153 5.249047
1 5.611360 2.131570 -3.252041
1 3.954652 3.335645 -1.826952
77 -0.041651 -0.233745 -0.013961
7 -1.329877 -2.520146 -1.637865
7 0.258400 -3.222804 -0.350170
15 -2.278697 0.274312 -0.428873
15 2.242141 -0.704612 0.394781
6 -1.978375 2.889287 -1.321838
6 -3.253656 -1.393908 1.608451
6 -2.153116 4.271702 -1.260462
6 -5.932798 -1.068788 0.867024
6 -2.479268 -1.828999 -2.257159
6 -2.545238 -0.311131 -2.153967
1 -1.292930 2.463545 -2.056419

6 -4.260484 -2.061671 2.311739
1 -2.208506 -1.521938 1.892028
1 -1.625877 4.915184 -1.968277
6 -5.598138 -1.900147 1.942592
1 -2.486071 -2.113768 -3.318732
1 -3.383598 -2.258279 -1.799753
1 -3.530995 0.005848 -2.524288
1 -1.781601 0.172355 -2.776410
1 -3.996192 -2.710041 3.149815
1 -6.383998 -2.422872 2.492505
1 -0.380312 -0.745162 1.414625
8 0.421914 1.731805 0.641472
6 -0.146684 2.336676 1.634038
1 -0.912644 1.722950 2.174904
8 0.097069 3.479323 1.990596
8 0.491440 0.649011 -2.104982
6 1.189456 0.126541 -2.960964
1 1.455477 -0.942965 -2.967872
8 1.699800 0.770856 -3.978936
1 1.463145 1.717912 -3.924627

TS-DE$_{PCP}$

6 -0.256440 -2.288516 -0.529445
6 2.821946 0.085754 2.083639
6 -2.748246 1.781446 -0.863273
6 -3.483439 -0.509920 0.758406
6 3.355514 0.320298 -0.741048
6 -3.282413 4.489755 -1.380082
6 -3.897215 3.847939 -0.297860
6 1.595975 -3.185451 0.916401
6 1.896242 0.457614 3.071336
6 -4.716467 -1.071565 0.394566
6 2.757424 -2.314462 0.428843
6 0.163525 -4.520947 -0.653456
6 5.049605 0.379136 -2.478202
6 -0.883964 -4.253245 -1.475419
6 3.711452 0.992917 4.580947
6 -3.633745 2.497424 -0.032861
6 4.634333 0.636136 3.600843
6 4.297227 -0.334745 -1.549728
6 4.201011 0.183260 2.356559
6 3.184389 1.718260 -0.871598
6 2.335346 0.906512 4.312137
6 4.877187 1.756237 -2.609138
6 3.944840 2.423893 -1.806086
1 -6.577629 -1.919304 1.091029
1 -4.989393 -1.183424 -0.650912
1 5.777640 -0.144759 -3.107227
1 0.679667 -5.445794 -0.428161
1 2.998043 -2.599782 -0.608487
1 3.649123 -2.472585 1.042996
1 1.170720 -2.793278 1.849682
1 0.824797 0.396672 2.854061
1 1.964850 -4.201194 1.122494
1 -3.490888 5.542508 -1.581554
1 -4.576396 4.396903 0.358651
1 -1.476291 -4.909438 -2.101322
1 -4.110252 2.014077 0.825698
1 4.053722 1.352139 5.552196
1 5.708204 0.706267 3.800727
1 4.451020 -1.416408 -1.461498
1 4.932880 -0.087615 1.588777
1 2.438561 2.242189 -0.270359
1 1.610071 1.195862 5.075166
1 5.461473 2.317171 -3.340084
1 3.805083 3.508203 -1.902789
77 -0.016051 -0.287609 -0.021041
7 -1.136517 -2.888016 -1.390715
7 0.529491 -3.308510 -0.076484
15 -2.274876 0.058895 -0.481754
15 2.267682 -0.535382 0.450211
6 -2.135671 2.429706 -1.954905
6 -3.170050 -0.333332 2.116377
6 -2.405940 3.771872 -2.212911

6 -5.620381 -1.474774 1.380360
6 -2.416902 -2.382309 -1.922969
6 -2.604201 -0.874997 -2.040248
1 -1.429106 1.883916 -2.594501
6 -4.082377 -0.726977 3.096155
1 -2.223134 0.129794 2.395477
1 -1.928682 4.267693 -3.065410
6 -5.301407 -1.305414 2.735594
1 -2.548067 -2.833275 -2.911471
1 -3.206854 -2.788547 -1.270717
1 -3.630134 -0.694030 -2.393642
1 -1.927179 -0.441677 -2.794101
1 -3.838445 -0.584519 4.155642
1 -6.012092 -1.622928 3.505986
1 -0.363841 -0.706583 1.441893
8 0.453147 3.422197 0.433482
6 -0.195969 2.674873 1.105550
8 -0.959626 2.470186 1.993986
1 0.139019 1.303099 0.523120
8 0.548644 0.560702 -2.128016
6 1.115371 0.194390 -3.147530
1 1.506720 0.921131 -3.883573
8 1.347993 -1.052124 -3.524624
1 0.989164 -1.695903 -2.878417

TS-DE$_{PCP}$b

6 -0.400534 -1.914022 -1.108854
6 3.057503 -0.610155 1.715854
6 -2.632220 2.114664 0.087908
6 -3.504431 -0.610278 0.648545
6 3.307084 0.476161 -0.968071
6 -2.959533 4.827752 0.702918
6 -2.866609 3.883471 1.732233
6 1.504814 -3.298387 -0.216105
6 2.294886 -0.235024 2.830939
6 -4.832861 -0.156587 0.720286
6 2.662976 -2.366328 -0.565612
6 -0.182593 -4.024052 -1.913337
6 3.937212 1.292312 -3.165366
6 -1.267804 -3.471239 -2.509750
6 4.228257 -0.523985 4.261479
6 -2.697631 2.533237 1.428147
6 4.995731 -0.895405 3.150811
6 3.276623 0.357492 -2.367577
6 4.414967 -0.938355 1.883540
6 3.983912 1.555839 -0.380359
6 2.880732 -0.193815 4.100128
6 4.620660 2.361223 -2.575176
6 4.639055 2.492760 -1.184066
1 -6.835041 -0.582424 1.400458
1 -5.106769 0.811183 0.294542
1 3.913370 1.188861 -4.252377
1 0.269781 -5.005189 -2.020844
1 2.808289 -2.338474 -1.654463
1 3.594173 -2.733238 -0.112659
1 1.214369 -3.194571 0.840198
1 1.243839 0.026182 2.704448
1 1.803841 -4.342002 -0.371420
1 -3.084402 5.886126 0.942094
1 -2.919930 4.201596 2.775646
1 -1.963948 -3.875307 -3.238481
1 -2.612158 1.800891 2.234613
1 4.684725 -0.490973 5.253352
1 6.050762 -1.150109 3.272708
1 2.725538 -0.457063 -2.842375
1 5.026735 -1.216862 1.022244
1 3.998949 1.669321 0.704702
1 2.279464 0.098347 4.963752
1 5.134738 3.093965 -3.201195
1 5.165720 3.328700 -0.718473
77 0.010302 -0.182783 -0.063929
7 -1.393811 -2.181114 -2.009045
7 0.329796 -3.064320 -1.055789
15 -2.254253 0.367333 -0.244719

15 2.310689 -0.663454 0.050597
6 -2.728623 3.062074 -0.941334
6 -3.161270 -1.850923 1.207079
6 -2.889599 4.415663 -0.629962
6 -5.804647 -0.940118 1.344187
6 -2.593423 -1.404056 -2.372268
6 -2.596966 0.077877 -2.029289
1 -2.667329 2.760639 -1.987344
6 -4.139526 -2.633157 1.826832
1 -2.129937 -2.203283 1.159797
1 -2.960934 5.149217 -1.435886
6 -5.459449 -2.179056 1.896416
1 -2.724663 -1.515428 -3.457290
1 -3.452454 -1.893500 -1.887955
1 -3.582361 0.480317 -2.307862
1 -1.827538 0.623052 -2.595735
1 -3.866546 -3.597602 2.260340
1 -6.222111 -2.789716 2.384928
1 -0.209929 -1.056790 1.201056
8 0.368232 1.749906 -1.456517
6 0.564475 2.283994 -0.376399
1 0.333194 1.302409 0.759793
8 0.859250 3.289893 0.180920

E$_{PCP}$

6 -0.023435 -0.529406 2.296631
6 -3.334687 -1.410897 -0.545836
6 3.142173 1.203199 -0.116176
6 3.047165 -1.548428 -1.031641
6 -2.878221 1.426770 -0.715561
6 4.128933 3.776044 -0.651512
6 4.097027 2.812624 -1.663869
6 -2.059357 0.802860 2.865531
6 -4.718569 -1.234430 -0.721577
6 4.441646 -1.728467 -1.077101
6 -3.033791 0.113400 1.905371
6 -0.616530 -0.481068 4.484058
6 -3.126322 2.535183 -2.863167
6 0.500488 -1.250462 4.380678
6 -4.892368 -3.471417 -1.632654
6 3.603797 1.532610 -1.400393
6 -3.516909 -3.649138 -1.463623
6 -2.845538 1.389220 -2.120013
6 -2.738065 -2.621979 -0.921352
6 -3.189904 2.631149 -0.069103
6 -5.492595 -2.262371 -1.260879
6 -3.429828 3.736774 -2.212863
6 -3.460861 3.781840 -0.817097
1 6.091664 -2.734918 -2.034505
1 5.092776 -1.175840 -0.395456
1 -3.101492 2.492891 -3.954326
1 -1.214309 -0.203475 5.347120
1 -3.984367 0.664772 1.868449
1 -3.256472 -0.900695 2.269210
1 -2.573616 1.039830 3.805312
1 -5.191676 -0.289116 -0.445745
1 -1.678506 1.744318 2.446212
1 4.513595 4.777025 -0.859278
1 4.455317 3.057666 -2.666218
1 1.073707 -1.780690 5.135347
1 3.574099 0.789869 -2.199335
1 -5.499488 -4.273782 -2.058156
1 -3.044501 -4.589264 -1.756930
1 -2.592028 0.458843 -2.633289
1 -1.663193 -2.755393 -0.791941
1 -3.219112 2.692320 1.018987
1 -6.567087 -2.119137 -1.395298
1 -3.641722 4.636500 -2.794772
1 -3.698443 4.715399 -0.302395
77 -0.006611 -0.225104 0.235908
7 0.844000 -1.270916 3.036164
7 -0.917121 -0.046496 3.200714
15 2.319053 -0.400511 0.192877
15 -2.343917 -0.068562 0.196659

6 3.167306 2.178269 0.894822
6 2.232521 -2.252827 -1.928506
6 3.663115 3.455969 0.628121
6 5.007816 -2.603130 -2.004502
6 2.013779 -1.962819 2.496926
6 2.985743 -0.998314 1.815720
1 2.779015 1.954023 1.890161
6 2.803268 -3.130610 -2.855947
1 1.152757 -2.106836 -1.898944
1 3.679674 4.205881 1.422031
6 4.188257 -3.307281 -2.894994
1 2.511443 -2.461955 3.337706
1 1.677776 -2.745282 1.799709
1 3.950393 -1.496112 1.646850
1 3.167188 -0.133629 2.469279
1 2.160499 -3.676399 -3.550261
1 4.632909 -3.992486 -3.620416
1 -0.055561 -1.768334 -0.003105
8 0.029269 2.063772 0.595838
6 0.168374 2.812798 -0.370059
1 0.212600 3.907568 -0.246939
8 0.277639 2.417688 -1.602166
1 0.200235 1.404213 -1.604019
1 -0.012079 -0.072431 -1.437127

TS-ED$_{PCP}$

6 -0.035331 0.518501 -2.220957
6 -3.431968 1.297833 0.398497
6 3.204009 -1.101410 -0.019526
6 3.004196 1.568299 1.122837
6 -2.836695 -1.496302 0.796719
6 4.305025 -3.664892 0.271419
6 4.220834 -2.809091 1.373487
6 -1.908042 -1.029948 -2.813024
6 -4.814142 1.073728 0.530814
6 4.399491 1.702400 1.246627
6 -2.980882 -0.397418 -1.923730
6 -0.562194 0.376791 -4.414101
6 -3.130234 -2.446375 3.011088
6 0.457745 1.269111 -4.295118
6 -5.112572 3.353597 1.290217
6 3.668585 -1.534237 1.232022
6 -3.739518 3.578217 1.164182
6 -2.895561 -1.342661 2.191475
6 -2.898661 2.553437 0.718643
6 -3.006193 -2.769680 0.235333
6 -5.649289 2.099942 0.972681
6 -3.296092 -3.716205 2.447295
6 -3.233689 -3.874909 1.060584
1 6.024603 2.637741 2.311342
1 5.067372 1.141764 0.588360
1 -3.178830 -2.316098 4.094424
1 -1.103706 0.023976 -5.286598
1 -3.865810 -1.047220 -1.870153
1 -3.302321 0.562887 -2.353022
1 -2.353318 -1.351290 -3.762503
1 -5.237732 0.094303 0.296775
1 -1.449907 -1.905350 -2.333992
1 4.736435 -4.662087 0.383910
1 4.583227 -3.135111 2.350908
1 0.988479 1.850841 -5.042666
1 3.595806 -0.878544 2.100909
1 -5.767933 4.154637 1.639739
1 -3.317116 4.553620 1.415236
1 -2.754777 -0.356092 2.639168
1 -1.825776 2.724539 0.621728
1 -2.955347 -2.918515 -0.843366
1 -6.721991 1.920752 1.073425
1 -3.473889 -4.581563 3.089635
1 -3.362928 -4.863283 0.614220
77 -0.019994 0.219780 -0.208861
7 0.761685 1.347304 -2.944121
7 -0.845930 -0.076227 -3.134339
15 2.320529 0.486998 -0.181853

15 -2.367320 -0.049726 -0.214553
6 3.276544 -1.970518 -1.120193
6 2.163731 2.289798 1.981512
6 3.830834 -3.243252 -0.975147
6 4.940483 2.542290 2.219656
6 1.854020 2.144635 -2.389267
6 2.923889 1.255191 -1.756768
1 2.885163 -1.668109 -2.093198
6 2.710153 3.132223 2.955083
1 1.081909 2.197783 1.886301
1 3.886149 -3.910121 -1.838308
6 4.095695 3.258494 3.076290
1 2.284963 2.725699 -3.213678
1 1.442317 2.855781 -1.658381
1 3.822341 1.846167 -1.532221
1 3.212774 0.462360 -2.460552
1 2.047227 3.690550 3.619609
1 4.521028 3.915450 3.838372
1 -0.135489 1.779923 -0.010434
8 0.125104 -1.991551 -0.522047
6 0.306318 -2.658310 0.542201
1 0.412039 -3.756172 0.412959
8 0.376254 -2.188868 1.696153
1 0.126094 -0.696253 1.498628
1 -0.073717 0.215039 1.543153

Cartesian coordinates (xyz format) for 4-based reaction pathways in formic acid

A$_{PCO-FA}$

6 -1.696807 -0.566040 1.125109
6 0.997959 -3.762916 0.389553
6 -1.150120 -2.920159 -0.821316
6 1.745818 -1.835954 -1.095304
6 0.484723 2.501764 -0.263713
6 2.686833 0.882285 0.620558
6 0.029248 4.860445 -1.706623
6 -0.227925 3.619825 -2.297034
6 -3.652926 -0.473829 -0.413711
6 1.934732 -3.300279 -0.742140
6 3.442192 1.544127 -0.358891
6 -4.099086 0.946315 -0.734235
6 -3.715581 -0.220645 2.083844
6 -2.773414 -0.061417 3.051723
6 0.904955 -1.346606 -2.092330
6 -0.007114 2.443866 -1.575529
6 -0.007678 -2.166581 -2.977383
6 -0.741877 -3.303816 -2.236620
6 -0.373784 -3.133199 0.336852
1 1.444578 -3.501863 1.361726
1 0.900315 -4.864862 0.380886
1 -2.227371 -2.830740 -0.657701
1 5.412981 1.924971 -1.153211
1 1.795358 -3.923326 -1.636367
1 2.976821 -3.451578 -0.424038
1 2.946642 2.171190 -1.101666
1 -4.797690 -0.140979 2.126732
1 -4.920554 1.249920 -0.055403
1 -4.501960 0.973324 -1.766153
1 -2.892578 -0.799334 -1.133876
1 -4.509632 -1.161325 -0.460465
1 -0.148833 5.780616 -2.267654
1 -0.613839 3.565941 -3.317507
1 2.541262 -1.172117 -0.755588
1 -2.866988 0.170703 4.108509
1 1.131286 -0.344900 -2.475208
1 -0.227489 1.474745 -2.024689
1 -0.753107 -1.479078 -3.407715
1 0.563455 -2.571425 -3.833906
1 -0.124841 -4.213770 -2.219391
1 -1.646013 -3.565352 -2.805920
1 -0.919725 -3.192936 1.285167
77 -0.183702 -1.016955 -0.163003
7 -1.544094 -0.276509 2.444472
7 -3.039154 -0.530590 0.910148
15 0.854571 0.937867 0.604682
6 0.742544 3.750661 0.325154
6 3.341027 0.072699 1.565047
6 0.509333 4.925176 -0.393004
6 4.833369 1.403669 -0.387636
6 -0.243291 0.001645 3.048461
6 0.402420 1.228070 2.393139
1 1.136312 3.811849 1.342054
6 4.729921 -0.060950 1.536176
1 2.768000 -0.468909 2.321375
1 0.708545 5.894269 0.071489
6 5.479690 0.603842 0.558118
1 -0.403977 0.187474 4.118151
1 0.392117 -0.889292 2.950374
1 1.304514 1.520476 2.947775
1 -0.302655 2.071351 2.431502
1 5.228450 -0.689577 2.277468
1 6.566497 0.496560 0.534226
8 -2.995637 1.799972 -0.582099
6 -3.312536 3.171051 -0.633518
1 -3.701293 3.467074 -1.626782
1 -4.069548 3.437930 0.128772
1 -2.390478 3.732099 -0.436028

TS-AB$_{PCO-FA}$

6 -1.810442 -1.598727 -0.330592

6	-1.166901	3.049471	-0.752210
6	1.074355	1.730952	-0.741477
6	0.510469	4.965702	-0.391319
6	1.360134	-1.094304	2.036847
6	2.716426	-0.985802	-0.553844
6	1.710283	-0.930759	4.816164
6	0.779450	-0.106454	4.179064
6	-3.780663	-0.277832	0.468371
6	-0.675328	4.207536	0.149555
6	3.833131	-0.498622	0.138598
6	-3.178968	0.406439	1.690901
6	-3.629093	-2.789730	0.291283
6	-2.674765	-3.676960	-0.103548
6	1.806227	4.647674	-0.250248
6	0.606061	-0.189091	2.795030
6	2.424681	3.470055	0.463822
6	1.574242	2.189916	0.603048
6	-0.096529	2.140533	-1.340083
1	-1.727185	3.466997	-1.601753
1	-1.906974	2.468821	-0.176971
1	1.839822	1.318109	-1.403803
1	5.897671	0.115515	0.010624
1	-0.459018	3.828357	1.156867
1	-1.521595	4.901328	0.272940
1	3.774735	-0.293750	1.207846
1	-4.639970	-2.945138	0.655953
1	-2.075182	0.383640	1.606969
1	-3.446477	-0.132776	2.621129
1	-3.703332	0.397047	-0.392230
1	-4.840186	-0.513810	0.628346
1	1.848100	-0.867238	5.897901
1	0.184119	0.603455	4.757313
1	0.278539	5.863529	-0.976332
1	-2.682683	-4.761691	-0.150454
1	2.535091	5.327010	-0.708325
1	-0.118899	0.455380	2.299550
1	3.355127	3.205559	-0.067686
1	2.753035	3.780907	1.473658
1	0.746165	2.339234	1.302419
1	2.214628	1.420825	1.051547
1	-0.136466	2.023095	-2.426184
77	-0.591691	-0.045278	-0.716429
7	-1.575420	-2.925463	-0.487345
7	-3.086532	-1.521859	0.144684
15	1.077595	-1.237540	0.232829
6	2.290066	-1.926893	2.685093
6	2.820010	-1.242750	-1.932448
6	2.464789	-1.841478	4.067154
6	5.035420	-0.268502	-0.538893
6	-0.292729	-3.454848	-0.940039
6	0.814520	-3.083500	0.038010
1	2.884761	-2.642370	2.112999
6	4.022847	-1.020936	-2.603141
1	1.953543	-1.603046	-2.491160
1	3.191590	-2.489311	4.562047
6	5.133443	-0.528893	-1.907494
1	-0.385994	-4.546151	-1.008490
1	-0.091544	-3.071795	-1.950213
1	1.765257	-3.531116	-0.282567
1	0.571434	-3.486057	1.031516
1	4.091488	-1.225595	-3.673768
1	6.073122	-0.348012	-2.434085
8	-3.647301	1.729797	1.709656
6	-3.134214	2.486125	2.782768
1	-3.418852	2.052539	3.760269
1	-2.029795	2.550824	2.742250
1	-3.549429	3.500577	2.707048
6	-2.021193	0.045493	-3.231894
1	-2.739395	0.190812	-4.059063
8	-2.261267	0.561400	-2.118917
8	-0.981131	-0.647851	-3.483006
1	-0.432074	-0.644604	-2.377582

$\mathbf{B}_{PCO-FA}$

6 0.459528 1.233149 -1.168895
6 3.387695 -1.481921 1.400901
6 2.832482 0.935713 0.567713
6 5.650639 -1.018167 0.337749
6 -2.702782 0.016827 -0.481238
6 -1.676717 -2.733921 -0.353131
6 -4.761788 1.793944 0.197785
6 -4.389493 0.779982 1.087446
6 0.156275 3.242004 0.305565
6 4.349441 -1.784087 0.242477
6 -3.003119 -3.187985 -0.425090
6 -1.301023 3.310911 0.745137
6 0.058937 3.206684 -2.197264
6 0.072991 2.244679 -3.157737
6 5.925823 0.256500 0.021278
6 -3.363014 -0.103550 0.754069
6 5.062686 1.333034 -0.577416
6 3.565544 1.038409 -0.750696
6 2.761558 -0.111250 1.454648
1 3.930374 -1.606662 2.354878
1 2.583725 -2.234099 1.437325
1 2.508879 1.899410 0.970984
1 -4.328395 -4.875810 -0.204608
1 3.839807 -1.630355 -0.718153
1 4.580971 -2.859047 0.283576
1 -3.808274 -2.501650 -0.692346
1 -0.089342 4.280223 -2.263864
1 -1.701865 2.286659 0.825995
1 -1.909622 3.851875 -0.007008
1 0.719063 2.685370 1.060498
1 0.585638 4.249504 0.222728
1 -5.561978 2.487634 0.464131
1 -4.898236 0.678926 2.048298
1 6.477583 -1.588933 0.777035
1 -0.058725 2.304472 -4.233747
1 6.953451 0.589484 0.213669
1 -3.075836 -0.886868 1.457366
1 5.184685 2.249107 0.028986
1 5.477829 1.598455 -1.567522
1 3.424800 0.144951 -1.366681
1 3.123169 1.870807 -1.316366
1 2.350624 0.143627 2.436078
77 0.616557 -0.186612 0.226363
7 0.318166 1.042393 -2.508732
7 0.291714 2.571834 -0.983177
15 -1.245181 -1.025511 -0.800039
6 -3.074630 1.039572 -1.367193
6 -0.646601 -3.627218 -0.017517
6 -4.103429 1.921676 -1.027421
6 -3.295005 -4.526467 -0.151836
6 0.245251 -0.280974 -3.122031
6 -0.983265 -1.063572 -2.641860
1 -2.561905 1.171224 -2.320690
6 -0.945398 -4.964203 0.248276
1 0.383433 -3.273146 0.039395
1 -4.383490 2.715427 -1.722847
6 -2.268583 -5.413994 0.185488
1 0.204607 -0.146386 -4.209749
1 1.166821 -0.826326 -2.883533
1 -0.862819 -2.121456 -2.912347
1 -1.901058 -0.708025 -3.130232
1 -0.142262 -5.655771 0.511581
1 -2.500310 -6.459603 0.400471
8 -1.326486 3.963806 1.987668
6 -2.576946 3.877813 2.630242
1 -3.384405 4.329238 2.021513
1 -2.853324 2.826676 2.836473
1 -2.504185 4.423202 3.581949
6 -0.280714 -0.556897 2.664931
1 -0.648574 -0.778521 3.686584
8 0.278201 -1.483244 2.002360
8 -0.417419 0.591108 2.173906
1 1.400386 -1.014905 -0.833548

C$_{PCO-FA}$

6 -1.024953 -0.949281 1.409554
6 2.475202 -3.330695 0.918381
6 0.446895 -3.116477 -0.658568
6 2.896303 -1.171399 -0.432309
6 -1.059784 2.159761 0.351251
6 1.911645 2.438884 0.190041
6 -3.595600 3.221169 -0.265742
6 -2.622738 3.093836 -1.260696
6 -2.971108 -1.766352 0.017073
6 3.423315 -2.527627 0.005551
6 1.736208 3.537097 -0.664377
6 -3.721918 -0.588187 -0.591563
6 -3.025453 -1.190077 2.433289
6 -2.171040 -0.653828 3.342931
6 2.193937 -0.951023 -1.598341
6 -1.368340 2.559815 -0.960075
6 1.809031 -2.030862 -2.580729
6 1.211013 -3.311483 -1.956310
6 1.007116 -3.162401 0.607988
1 2.632980 -3.031551 1.964558
1 2.724490 -4.405748 0.864703
1 -0.633157 -3.251344 -0.719287
1 2.685038 5.153537 -1.727349
1 3.672061 -3.112578 -0.890957
1 4.370802 -2.371366 0.540415
1 0.744753 3.815602 -1.014995
1 -4.072014 -1.467364 2.514251
1 -3.022648 0.256726 -0.708113
1 -4.541449 -0.255524 0.077706
1 -2.216845 -2.090200 -0.700885
1 -3.653234 -2.597148 0.243450
1 -4.580022 3.627890 -0.506754
1 -2.841954 3.398768 -2.286291
1 3.348326 -0.303331 0.044672
1 -2.315336 -0.369060 4.380531
1 2.107266 0.070463 -1.972602
1 -0.646341 2.419641 -1.760990
1 1.100636 -1.598904 -3.295955
1 2.705166 -2.296757 -3.169981
1 2.003205 -4.054315 -1.782647
1 0.525815 -3.765181 -2.685700
1 0.332985 -3.361449 1.445532
7 -0.953527 -0.508245 2.699329
7 -2.310585 -1.362101 1.255447
15 0.542688 1.353274 0.731881
6 -2.044351 2.292270 1.346559
6 3.205120 2.131765 0.651946
6 -3.302331 2.814458 1.037472
6 2.837388 4.300346 -1.062890
6 0.224889 0.115749 3.301221
6 0.666215 1.400904 2.586117
1 -1.857269 1.985392 2.373234
6 4.300869 2.897191 0.252790
1 3.366549 1.299975 1.340418
1 -4.054005 2.899561 1.824897
6 4.120217 3.980920 -0.612506
1 -0.020919 0.338449 4.346531
1 1.040960 -0.617466 3.302161
1 1.722060 1.580400 2.826341
1 0.112342 2.279418 2.942281
1 5.297308 2.645740 0.621879
1 4.977741 4.579060 -0.928121
8 -4.225138 -1.005360 -1.834718
6 -4.883897 0.024441 -2.534082
1 -5.750222 0.416172 -1.965888
1 -4.204458 0.871639 -2.746279
1 -5.243320 -0.391136 -3.486142
77 0.595986 -0.920475 0.139912
6 -0.702985 0.024769 -2.628115
1 -1.568647 -0.139711 -3.319579
8 -0.792144 -0.690319 -1.570741
8 0.177074 0.828611 -2.936283
1 1.465376 -1.013936 1.438842

TS-CD$_{PCO\text{-}FA}$

6 -1.498748 -0.330177 1.193250
6 1.293313 2.568757 -0.194879
6 3.122020 0.534537 0.755117
6 1.282837 4.992408 -1.608505
6 1.276203 4.994794 -0.210402
6 -3.637446 -0.583089 -0.132811
6 4.013193 0.893277 -0.268954
6 -4.643245 0.498248 -0.485198
6 -3.436147 0.072790 2.282070
6 -2.423702 0.251632 3.170260
6 1.288329 3.789777 0.496911
1 6.026306 0.758764 -1.028709
1 3.677583 1.519150 -1.097295
1 -4.511822 0.174341 2.372402
1 -5.003128 0.304326 -1.514850
1 -4.160533 1.492886 -0.495812
1 -2.935511 -0.713175 -0.959740
1 -4.174782 -1.530217 0.026519
1 1.269783 5.936925 -2.156850
1 1.265530 5.940147 0.336449
1 -2.432999 0.535003 4.218313
1 1.299106 3.816741 1.587062
7 -1.245248 -0.007045 2.487056
7 -2.853416 -0.282442 1.071346
15 1.337538 0.947197 0.642963
6 1.310343 2.573593 -1.600493
6 3.582304 -0.271254 1.809985
6 1.304838 3.779219 -2.302869
6 5.343054 0.466775 -0.228276
6 0.087633 0.143477 3.065762
6 0.857377 1.285673 2.405043
1 1.296532 1.630770 -2.148873
6 4.913754 -0.691977 1.849171
1 2.909448 -0.585001 2.609310
1 1.307726 3.769300 -3.394744
6 5.797140 -0.326017 0.829504
1 -0.045844 0.358001 4.132950
1 0.620175 -0.814864 2.992286
1 1.757985 1.521304 2.988150
1 0.217815 2.178981 2.411273
1 5.258228 -1.312279 2.679369
1 6.837000 -0.658423 0.859287
8 -5.714062 0.462004 0.430571
6 -6.662021 1.484218 0.222055
1 -6.208736 2.488377 0.328039
1 -7.116858 1.416154 -0.784683
1 -7.451810 1.368235 0.977136
6 0.035289 -2.829690 -2.755879
6 1.854020 -1.694652 -1.323782
6 -1.134416 -2.704737 -0.468645
6 -1.171713 -3.241067 -1.885394
6 -0.046973 -3.022392 0.462384
6 1.187456 -3.802606 -0.002222
6 2.310771 -2.904028 -0.542759
6 0.832165 -1.646423 -2.242761
1 -0.324208 -2.572998 -3.761921
1 0.713639 -3.685954 -2.897321
1 2.554342 -0.860162 -1.314821
1 -1.267962 -4.341420 -1.858895
1 -2.095717 -2.862831 -2.346477
1 -2.118423 -2.729363 0.007434
1 -0.390348 -3.282427 1.471767
1 1.578663 -4.389165 0.840122
1 0.861471 -4.529670 -0.759951
1 2.997721 -3.494358 -1.174493
1 2.916476 -2.538598 0.299909
1 0.752528 -0.730039 -2.831460
77 -0.099959 -0.852743 -0.191046
1 0.514968 -1.864601 0.985284
8 -1.026123 0.409976 -1.611427
6 -1.565096 1.562981 -1.353236
1 -1.430835 1.923265 -0.304792
8 -2.194102 2.225308 -2.161739

D$_{PCO\text{-}FA}$

6 -1.454106 -0.404684 1.067804
6 1.362970 2.512577 -0.083468
6 3.096892 0.380582 0.748921
6 1.502454 5.021078 -1.322684
6 1.509830 4.928248 0.071910
6 -3.606316 -0.411935 -0.280452
6 3.917401 0.631297 -0.363136
6 -4.612853 0.714812 -0.437320
6 -3.399271 -0.140038 2.196270
6 -2.391970 -0.087794 3.104379
6 1.444457 3.678676 0.693374
1 5.877423 0.420318 -1.233514
1 3.525054 1.158043 -1.233655
1 -4.475397 -0.049659 2.293530
1 -4.961799 0.712510 -1.488506
1 -4.132037 1.694721 -0.261689
1 -2.913148 -0.384747 -1.122637
1 -4.148598 -1.369708 -0.284831
1 1.549210 5.999743 -1.805296
1 1.567490 5.831494 0.682908
1 -2.405235 0.050188 4.181223
1 1.461790 3.627237 1.782765
7 -1.211383 -0.255349 2.398519
7 -2.811929 -0.321232 0.949554
15 1.328566 0.866832 0.696605
6 1.362476 2.609670 -1.484838
6 3.635275 -0.287793 1.860841
6 1.431930 3.860233 -2.099038
6 5.250287 0.216169 -0.363235
6 0.112242 -0.137330 2.999872
6 0.836475 1.095763 2.457960
1 1.272699 1.713039 -2.098143
6 4.970012 -0.700222 1.856722
1 3.028194 -0.497428 2.742070
1 1.421412 3.927107 -3.188800
6 5.779316 -0.451921 0.745108
1 -0.027539 -0.041568 4.082973
1 0.674018 -1.062859 2.818549
1 1.726402 1.325374 3.058762
1 0.161825 1.960903 2.516759
1 5.375371 -1.217419 2.728749
1 6.822409 -0.775304 0.743786
8 -5.692960 0.510142 0.444509
6 -6.646058 1.548441 0.408586
1 -6.202190 2.519405 0.700338
1 -7.085478 1.659833 -0.601060
1 -7.445591 1.294261 1.118118
6 -0.627211 -3.979811 -0.139702
6 -1.032426 -2.217320 -1.950707
6 1.226425 -2.250387 0.286420
6 0.585123 -3.561196 0.712245
6 1.789636 -2.054753 -1.118448
6 1.415154 -3.027373 -2.244135
6 0.071901 -2.656593 -2.889426
6 -1.350591 -2.803558 -0.749207
1 -1.338028 -4.539662 0.483985
1 -0.318617 -4.661909 -0.944360
1 -1.757534 -1.529608 -2.392274
1 1.334347 -4.372958 0.738281
1 0.256078 -3.421466 1.754004
1 1.981923 -1.979467 1.030405
1 2.867836 -1.845683 -1.078371
1 2.191639 -2.990099 -3.021528
1 1.423338 -4.055046 -1.857346
1 -0.314071 -3.499589 -3.490574
1 0.241545 -1.835263 -3.602596
1 -2.306814 -2.544129 -0.297826
77 -0.031792 -0.724411 -0.325174
8 -1.065816 0.747946 -1.625797
6 -1.581524 1.869115 -1.253213
1 -1.427300 2.126174 -0.173090
8 -2.223122 2.630471 -1.966677
1 1.449652 -1.005687 -1.532971

E$_{PCO\text{-}FA}$

6 -1.632946 -0.405373 1.053082
6 1.300938 2.634336 0.300263
6 2.874254 0.379368 1.058327
6 1.725854 5.249580 -0.629838
6 1.823012 4.966169 0.736286
6 -3.691061 -0.322721 -0.461868
6 3.762439 0.666090 0.009335
6 -4.655289 0.841865 -0.624715
6 -3.675202 -0.155884 2.017926
6 -2.751780 -0.107828 3.007849
6 1.617008 3.666121 1.201541
1 5.757901 0.431439 -0.770838
1 3.433236 1.262909 -0.841042
1 -4.755522 -0.064731 2.023021
1 -4.902771 0.930895 -1.700244
1 -4.174643 1.792090 -0.327006
1 -2.945860 -0.281450 -1.257671
1 -4.265639 -1.257460 -0.549474
1 1.886091 6.268068 -0.990185
1 2.063531 5.760296 1.446340
1 -2.857508 0.020207 4.080551
1 1.714849 3.467256 2.269403
7 -1.512544 -0.257526 2.405274
7 -2.982124 -0.317758 0.823907
15 1.129173 0.918623 0.916795
6 1.213598 2.925511 -1.068258
6 3.332179 -0.357537 2.162138
6 1.423140 4.226108 -1.531058
6 5.077569 0.201383 0.051948
6 -0.265200 -0.100259 3.145659
6 0.516337 1.109940 2.646161
1 0.953902 2.142403 -1.773413
6 4.649831 -0.819980 2.202077
1 2.676587 -0.577809 3.004800
1 1.342231 4.436627 -2.599342
6 5.522508 -0.549021 1.144846
1 -0.533858 0.050990 4.197819
1 0.318341 -1.025353 3.091899
1 1.366050 1.315127 3.311145
1 -0.140178 1.991670 2.660068
1 4.993027 -1.392419 3.066375
1 6.551530 -0.913674 1.176931
8 -5.818501 0.601549 0.134048
6 -6.740572 1.667331 0.093721
1 -6.306090 2.598021 0.506062
1 -7.077152 1.872606 -0.940382
1 -7.610581 1.380763 0.700576
6 -1.298326 -3.645817 0.305958
6 -0.654739 -2.459801 -1.889477
6 0.802822 -2.208651 0.802034
6 -0.239741 -3.199290 1.341436
6 1.900518 -2.892581 -0.031595
6 1.438554 -3.852978 -1.133491
6 0.576079 -3.253709 -2.252231
6 -1.491892 -2.661365 -0.822958
1 -2.265980 -3.784851 0.808220
1 -1.033218 -4.622860 -0.124190
1 -1.051324 -1.833360 -2.696744
1 0.269621 -4.087429 1.757225
1 -0.767855 -2.738622 2.187462
1 1.328865 -1.811717 1.677189
1 2.516917 -3.485051 0.669926
1 2.330883 -4.287801 -1.612898
1 0.916595 -4.705826 -0.676586
1 0.207101 -4.077200 -2.893560
1 1.207095 -2.652466 -2.920225
1 -2.480432 -2.222617 -0.908881
77 -0.129262 -0.619811 -0.266116
8 -1.074390 0.780264 -1.706949
6 -1.650851 1.897745 -1.408625
1 -1.586694 2.179593 -0.325749
8 -2.247538 2.623414 -2.191472
1 2.576899 -2.134016 -0.450223

8 1.530780 -0.501706 -1.773516
6 1.388792 -0.068321 -2.912842
1 0.428016 0.321380 -3.285196
8 2.362532 -0.020535 -3.780738
1 3.184203 -0.378988 -3.389164

TS-EF$_{PCO\text{-}FA}$

6 1.545954 0.208838 -0.963796
6 -0.869333 2.501482 -0.318061
6 -3.133250 0.706627 -0.746758
6 -0.031511 4.964380 0.731246
6 0.370622 4.574331 -0.553146
6 3.365095 0.656972 0.763141
6 -3.730615 -0.547138 -0.969776
6 4.717275 -0.032264 0.807909
6 3.414043 1.333219 -1.615299
6 2.585872 1.281848 -2.685099
6 -0.049564 3.348903 -1.080770
1 -5.574321 -1.657090 -1.110295
1 -3.114690 -1.428946 -1.140176
1 4.406048 1.753494 -1.480253
1 4.657470 -1.019560 0.304055
1 4.973418 -0.221181 1.871147
1 3.475581 1.698960 1.098647
1 2.666273 0.180027 1.441260
1 0.301290 5.918970 1.140312
1 1.015510 5.226370 -1.151475
1 2.694173 1.662636 -3.694946
1 0.278210 3.065582 -2.078124
7 1.444498 0.603715 -2.272927
7 2.769472 0.681284 -0.576335
15 -1.308174 0.822500 -0.879027
6 -1.275672 2.903769 0.965627
6 -3.940966 1.836715 -0.541965
6 -0.864422 4.128734 1.485647
6 -5.118253 -0.675987 -0.947770
6 0.334583 0.380831 -3.206632
6 -1.066689 0.764608 -2.699696
1 -1.924598 2.249539 1.555968
6 -5.330419 1.702185 -0.517374
1 -3.493929 2.822871 -0.397059
1 -1.180843 4.425229 2.486934
6 -5.923920 0.447631 -0.714454
1 0.568880 0.993866 -4.084066
1 0.348583 -0.664048 -3.533002
1 -1.777633 -0.002320 -3.039109
1 -1.415350 1.717133 -3.125240
1 -5.954297 2.587021 -0.348061
1 -7.010148 0.348391 -0.694788
8 5.691858 0.780421 0.199580
6 6.967525 0.184648 0.144147
1 7.348620 -0.048410 1.159289
1 6.952583 -0.752349 -0.441899
1 7.650463 0.896270 -0.340303
6 -0.187171 -3.765344 1.515511
6 1.753155 -2.290253 0.595480
6 -1.266280 -3.343065 -0.766535
6 -0.616465 -4.357582 0.173756
6 -0.347159 -2.227659 -1.282338
6 0.986905 -2.784066 -1.807950
6 2.068765 -2.983407 -0.716019
6 0.752887 -2.592602 1.502056
1 -1.080139 -3.491629 2.092491
1 0.320123 -4.548952 2.115075
1 2.560832 -1.677466 0.990302
1 0.231546 -4.850742 -0.321113
1 -1.340148 -5.165595 0.381139
1 -2.144950 -2.919940 -0.263629
1 1.401559 -2.110630 -2.567539
1 0.797840 -3.739284 -2.332065
1 2.250152 -4.054390 -0.525026
1 3.023255 -2.578124 -1.078114
1 0.854015 -2.103323 2.475258
1 -1.644326 -3.890177 -1.650527

1 -0.867269 -1.773359 -2.140438
77 0.036862 -0.740768 0.163056
8 -3.053653 -2.184885 1.846741
6 -2.550873 -1.105125 1.950332
8 -2.604646 0.001745 2.401425
1 -1.297376 -1.193078 1.153797
8 0.654750 0.683144 1.964203
6 0.138785 1.079553 2.995954
1 -0.883636 0.802810 3.306081
8 0.735892 1.885533 3.844017
1 1.626674 2.115717 3.508489

TS-EF$_{PCO\text{-}FA}$b

6 -1.531135 -0.993118 1.017704
6 0.156822 2.742442 0.122928
6 2.479564 1.166229 0.876477
6 -0.683885 5.229439 -0.848629
6 0.583096 5.097006 -0.272190
6 -3.475033 -1.187752 -0.551366
6 3.179093 0.868012 2.054125
6 -4.490154 -0.057281 -0.623507
6 -3.572009 -1.287587 1.950873
6 -2.700366 -1.020437 2.956580
6 1.005405 3.857957 0.214349
1 5.110963 0.581302 2.970307
1 2.651897 0.664487 2.986576
1 -4.637732 -1.492337 1.973178
1 -5.354131 -0.264052 0.037440
1 -4.880509 -0.004744 -1.658645
1 -2.681732 -1.004757 -1.284010
1 -3.966925 -2.143174 -0.784847
1 -1.009628 6.197749 -1.235363
1 1.248059 5.960526 -0.201352
1 -2.847041 -0.947151 4.029949
1 1.996064 3.762028 0.660614
7 -1.457008 -0.833674 2.367107
7 -2.845155 -1.249790 0.768724
15 0.656654 1.119780 0.788448
6 -1.120427 2.877938 -0.445758
6 3.195731 1.417384 -0.307775
6 -1.532983 4.121374 -0.930840
6 4.576874 0.819844 2.048231
6 -0.311228 -0.267932 3.077409
6 0.026920 1.119300 2.527666
1 -1.795084 2.023173 -0.516520
6 4.589586 1.380431 -0.305109
1 2.668463 1.634177 -1.238364
1 -2.522041 4.222299 -1.382612
6 5.283586 1.077036 0.871779
1 -0.595941 -0.182868 4.132948
1 0.540192 -0.956425 3.028732
1 0.755436 1.631233 3.171351
1 -0.883299 1.736286 2.519695
1 5.136223 1.581620 -1.228812
1 6.375128 1.040361 0.869777
8 -3.858460 1.137869 -0.243582
6 -4.737032 2.235230 -0.129633
1 -5.207474 2.485189 -1.099182
1 -5.538579 2.033388 0.605488
1 -4.148913 3.096589 0.214207
8 -0.850892 0.500631 -2.420522
6 0.351189 0.602676 -2.572092
1 1.067590 -0.240802 -1.619825
8 1.220028 1.122054 -3.204515
6 1.450842 -2.836350 -2.415101
6 -0.694854 -2.981729 -0.937580
6 2.609542 -2.191415 -0.191311
6 2.462769 -3.206261 -1.326213
6 1.369026 -1.930854 0.670528
6 0.699373 -3.205554 1.191813
6 -0.191482 -3.912083 0.147435
6 0.038607 -2.488010 -2.002138
1 1.844900 -2.000842 -3.014686
1 1.360812 -3.684521 -3.119868

1 -1.776849 -2.921278 -1.038990
1 2.226020 -4.195413 -0.908633
1 3.443223 -3.325858 -1.814658
1 2.993175 -1.237115 -0.582214
1 0.078443 -2.953306 2.061657
1 1.475460 -3.897033 1.564685
1 0.344399 -4.748579 -0.325093
1 -1.063119 -4.352092 0.651216
1 -0.551701 -2.039697 -2.807903
77 -0.037568 -0.762473 -0.385496
1 3.394121 -2.566959 0.492134
1 1.718124 -1.353439 1.534319

TS-BC'$_{PCO\text{-}FA}$

6 1.037623 -0.673934 1.302619
6 0.959177 3.658534 -0.672167
6 2.258068 1.372269 -0.586949
6 3.107689 4.601738 0.270350
6 -1.978871 -2.083915 0.282342
6 -3.444401 0.448585 0.551289
6 -1.897842 -4.669895 -0.798760
6 -2.463129 -3.625507 -1.530552
6 2.570864 -2.041703 -0.163671
6 1.677355 4.218726 0.564528
6 -4.634906 -0.295017 0.489598
6 4.062128 -2.302019 0.018805
6 2.126201 -2.414304 2.261784
6 1.355136 -1.839664 3.224022
6 4.205437 3.839716 0.174663
6 -2.501172 -2.333406 -1.001885
6 4.395683 2.361550 0.403715
6 3.149405 1.503543 0.634605
6 1.360088 2.274952 -1.131743
1 1.141172 4.334748 -1.526619
1 -0.130086 3.678600 -0.533209
1 2.605506 0.617007 -1.300563
1 -6.779046 -0.221456 0.261931
1 1.587875 3.508868 1.400741
1 1.134946 5.121989 0.886123
1 -4.615280 -1.383346 0.568355
1 2.797073 -3.265208 2.300164
1 4.244337 -3.236856 0.583432
1 4.516856 -1.475473 0.604727
1 2.069054 -2.915787 -0.607398
1 2.445426 -1.217288 -0.863965
1 -1.865781 -5.681140 -1.218251
1 -2.868353 -3.810227 -2.529759
1 3.243427 5.665238 0.034008
1 1.223412 -2.087945 4.271477
1 5.133860 4.356085 -0.103233
1 -2.909736 -1.512669 -1.597497
1 4.968788 1.950106 -0.453018
1 5.070893 2.230714 1.273085
1 2.589376 1.864135 1.504919
1 3.494199 0.492535 0.902237
1 1.089170 2.075498 -2.172936
77 0.065886 0.600163 -0.022505
7 0.700226 -0.781764 2.623511
7 1.919072 -1.687079 1.095824
15 -1.839812 -0.362214 0.862392
6 -1.400004 -3.135978 1.006355
6 -3.500298 1.849436 0.468564
6 -1.359396 -4.426403 0.464098
6 -5.857488 0.359372 0.310808
6 -0.368153 -0.007904 3.258678
6 -1.742612 -0.408599 2.718810
1 -0.958106 -2.967446 1.987788
6 -4.723695 2.496903 0.283515
1 -2.577829 2.436716 0.530983
1 -0.902717 -5.236507 1.039741
6 -5.900730 1.753476 0.197583
1 -0.312703 -0.191849 4.338942
1 -0.183910 1.059415 3.081036
1 -2.500655 0.297854 3.088943

1 -2.037731 -1.411856 3.072099
1 -4.746901 3.585540 0.188692
1 -6.857779 2.258655 0.045370
8 4.627060 -2.379690 -1.262152
6 6.024085 -2.588277 -1.239174
1 6.547766 -1.766713 -0.714044
1 6.282364 -3.539526 -0.732469
1 6.379732 -2.632968 -2.277634
1 -0.069792 1.690603 1.084459
8 -2.178194 0.766153 -2.631398
6 -1.864109 1.807851 -2.122627
8 -1.939513 2.998076 -2.082073
1 -0.970455 1.490122 -0.999403
8 0.258960 -0.998994 -1.560898
6 0.103351 -1.181337 -2.753924
1 -0.197261 -0.381174 -3.453813
8 0.286863 -2.385257 -3.251885
1 0.133442 -2.391439 -4.208214

TS-BC'$_{PCO-FA}$b

6 0.570624 -1.306511 1.197152
6 3.318374 1.843226 -1.357275
6 2.900302 -0.617253 -0.549681
6 5.633753 1.445127 -0.386893
6 -2.653216 -0.311807 0.490759
6 -1.953292 2.520156 0.490290
6 -4.514880 -2.251296 -0.300429
6 -4.248039 -1.157054 -1.130622
6 0.498807 -3.294481 -0.328048
6 4.311450 2.161854 -0.229264
6 -3.309053 2.814628 0.713481
6 -0.901517 -3.306282 -0.929334
6 0.299306 -3.329233 2.169995
6 0.213824 -2.391976 3.152166
6 5.967923 0.181558 -0.084280
6 -3.321408 -0.190044 -0.740290
6 5.166749 -0.921407 0.550591
6 3.662333 -0.691934 0.754920
6 2.745386 0.443723 -1.419261
1 3.825335 2.001596 -2.325505
1 2.497252 2.575400 -1.342747
1 2.676633 -1.595502 -0.983570
1 -4.843476 4.328522 0.649998
1 3.846041 1.977413 0.748134
1 4.502811 3.244938 -0.264144
1 -3.992082 2.037963 1.062260
1 0.231089 -4.412209 2.208331
1 -1.298016 -2.276509 -0.952522
1 -1.587991 -3.910857 -0.304559
1 1.167428 -2.754723 -1.005620
1 0.885207 -4.315617 -0.214098
1 -5.238004 -3.008574 -0.610351
1 -4.762095 -1.056518 -2.088640
1 6.417215 2.046000 -0.864058
1 0.058328 -2.488270 4.222359
1 6.996670 -0.116164 -0.322628
1 -3.119943 0.659612 -1.395643
1 5.313026 -1.839279 -0.047563
1 5.615951 -1.156264 1.533580
1 3.491190 0.190466 1.379473
1 3.269048 -1.546095 1.324074
1 2.365266 0.170612 -2.408619
77 0.638831 0.237747 -0.175696
7 0.381906 -1.161135 2.534902
7 0.516126 -2.647892 0.979479
15 -1.312002 0.859946 0.871464
6 -2.919387 -1.413674 1.317836
6 -1.084145 3.526922 0.044165
6 -3.851453 -2.376224 0.922660
6 -3.788001 4.105001 0.480784
6 0.211994 0.138453 3.179792
6 -1.066017 0.845755 2.714169
1 -2.399225 -1.543459 2.267559
6 -1.569153 4.816486 -0.182886

1 -0.030935 3.301883 -0.128949
1 -4.051423 -3.230396 1.572683
6 -2.919971 5.105808 0.031627
1 0.177306 -0.023854 4.263962
1 1.094926 0.752023 2.962013
1 -1.026707 1.898407 3.026418
1 -1.960177 0.404468 3.175901
1 -0.889214 5.595352 -0.534219
1 -3.298570 6.113605 -0.153054
8 -0.790720 -3.830486 -2.226138
6 -1.979451 -3.705727 -2.973085
1 -2.824149 -4.235611 -2.492030
1 -2.266642 -2.644746 -3.097038
1 -1.801341 -4.147803 -3.963563
1 1.313566 1.109187 0.909783
8 -0.332161 -0.363692 -2.346541
6 -0.428571 0.852942 -2.375901
1 0.466624 1.469959 -1.372336
8 -0.949214 1.776939 -2.917065

C' $_{PCO\text{-}FA}$

6 -1.168924 -0.744594 1.470283
6 1.986122 -3.703667 0.350389
6 -0.292279 -3.053957 -0.679491
6 2.373082 -1.494541 -0.906150
6 -0.588603 2.277827 -0.029379
6 2.338653 1.983951 0.064002
6 -2.742382 3.710351 -1.126579
6 -1.792050 3.117765 -1.967101
6 -3.241750 -1.234732 0.112141
6 2.812902 -2.937968 -0.697725
6 2.403569 3.107602 -0.772022
6 -3.676211 0.023439 -0.628903
6 -3.182978 -0.482817 2.475622
6 -2.238543 -0.041884 3.346094
6 1.453246 -1.085929 -1.875285
6 -0.732780 2.392728 -1.424333
6 0.715338 -2.017687 -2.814135
6 0.128205 -3.266942 -2.128681
6 0.539985 -3.264229 0.427931
1 2.434371 -3.559479 1.343368
1 2.019143 -4.789901 0.145413
1 -1.360587 -3.144455 -0.487351
1 3.684131 4.508278 -1.796486
1 2.788092 -3.462225 -1.664166
1 3.867212 -2.934116 -0.385430
1 1.492037 3.587028 -1.127786
1 -4.263484 -0.540643 2.564134
1 -2.787190 0.640788 -0.850338
1 -4.353687 0.634370 0.001298
1 -2.593549 -1.818635 -0.542331
1 -4.114644 -1.845747 0.379808
1 -3.580837 4.264656 -1.553424
1 -1.885360 3.207752 -3.051337
1 3.075913 -0.737986 -0.561158
1 -2.321437 0.361588 4.350582
1 1.533363 -0.053488 -2.226288
1 -0.025579 1.898072 -2.092343
1 -0.099905 -1.445241 -3.278317
1 1.394993 -2.317165 -3.633266
1 0.844009 -4.101139 -2.172231
1 -0.753210 -3.598399 -2.696115
1 0.044973 -3.481675 1.378134
7 -1.016389 -0.206821 2.714717
7 -2.515755 -0.901732 1.331735
15 0.764317 1.228101 0.610392
6 -1.547270 2.866537 0.806018
6 3.531826 1.403871 0.533553
6 -2.622785 3.574048 0.256743
6 3.645129 3.632488 -1.145089
6 0.255170 0.269772 3.249616
6 0.808771 1.456434 2.455345
1 -1.484622 2.771019 1.889798
6 4.767254 1.934608 0.162689

1 3.496026 0.536340 1.196678
1 -3.368409 4.017217 0.920100
6 4.826362 3.047668 -0.683431
1 0.091444 0.559289 4.294891
1 0.969555 -0.561935 3.226676
1 1.863998 1.597348 2.724657
1 0.292720 2.391067 2.711602
1 5.686118 1.476143 0.534287
1 5.793779 3.460723 -0.977392
8 -4.317115 -0.395711 -1.806192
6 -4.740094 0.671102 -2.622800
1 -5.452077 1.334216 -2.093630
1 -3.888003 1.289371 -2.961439
1 -5.241852 0.242589 -3.501955
77 0.379401 -1.025026 0.120768
1 1.413899 -1.246645 1.409218
1 -0.741843 -0.555415 -1.004450

TS-C'F$_{PCO-FA}$

6 -1.215246 -0.618996 1.451525
6 2.091616 -3.615674 0.418374
6 -0.374345 -3.091417 -0.200041
6 2.093244 -1.576429 -1.201528
6 -0.380836 2.365596 -0.019948
6 2.515814 1.849786 0.023779
6 -2.499403 3.896616 -1.056039
6 -1.549119 3.336950 -1.918845
6 -3.318214 -1.028877 0.148129
6 2.676637 -2.940652 -0.839461
6 2.670441 3.069339 -0.650381
6 -3.647623 0.228223 -0.647602
6 -3.173752 -0.238241 2.516594
6 -2.175141 0.132211 3.361900
6 1.012498 -1.397696 -2.061636
6 -0.505926 2.566553 -1.406999
6 0.172645 -2.515762 -2.645417
6 -0.198724 -3.612343 -1.630287
6 0.697786 -3.127384 0.771006
1 2.748086 -3.427117 1.280397
1 2.054597 -4.711279 0.286564
1 -1.364585 -3.273098 0.226372
1 4.056440 4.462267 -1.540980
1 2.564606 -3.605123 -1.708917
1 3.759269 -2.811641 -0.698716
1 1.801381 3.683681 -0.886666
1 -4.252618 -0.224633 2.637891
1 -2.713573 0.781077 -0.857141
1 -4.300820 0.900872 -0.055382
1 -2.706539 -1.695937 -0.466675
1 -4.236370 -1.556695 0.439707
1 -3.324531 4.487610 -1.459160
1 -1.629042 3.490508 -2.997165
1 2.779215 -0.734234 -1.109465
1 -2.201971 0.530466 4.371701
1 0.954718 -0.445545 -2.594062
1 0.206596 2.105223 -2.093925
1 -0.749775 -2.062898 -3.037609
1 0.701971 -2.947666 -3.515519
1 0.546681 -4.422629 -1.644732
1 -1.143034 -4.078250 -1.947456
1 0.393487 -3.350603 1.798745
7 -0.984880 -0.109224 2.692844
7 -2.568980 -0.696598 1.353544
15 0.895638 1.193705 0.566414
6 -1.338764 2.924499 0.836981
6 3.654808 1.086564 0.339417
6 -2.394563 3.683206 0.318976
6 3.946351 3.511221 -1.015121
6 0.344758 0.234704 3.191933
6 0.981186 1.392453 2.417704
1 -1.290407 2.764289 1.914083
6 4.926195 1.535295 -0.019510
1 3.546004 0.138719 0.872868
1 -3.138292 4.103497 0.999348

6 5.073746 2.747022 -0.703983
1 0.246950 0.495225 4.253094
1 0.978210 -0.658708 3.120022
1 2.047842 1.439629 2.676381
1 0.543126 2.358607 2.702815
1 5.803321 0.935117 0.232018
1 6.068252 3.095519 -0.991352
8 -4.277244 -0.179144 -1.834692
6 -4.593449 0.894130 -2.690039
1 -5.285702 1.611956 -2.208015
1 -3.688260 1.451093 -2.997795
1 -5.079491 0.479856 -3.584726
77 0.229060 -1.016273 0.047221
1 1.212863 -1.680183 1.257181
1 -0.841500 -0.352983 -0.990005

F*PCO-FA*

6 0.976490 -0.832285 -1.489754
6 -2.360402 -3.285718 1.358455
6 0.221195 -2.673169 1.027642
6 -1.894229 -0.778701 1.852408
6 0.857585 2.438501 -0.246657
6 -2.079573 2.482783 -0.056605
6 3.328603 3.559232 0.508903
6 2.304037 3.414354 1.451063
6 3.137788 -1.498424 -0.396090
6 -2.500006 -2.095705 2.303739
6 -1.985061 3.821225 0.352212
6 3.685085 -0.270838 0.324415
6 2.838185 -0.868921 -2.789633
6 1.820246 -0.401804 -3.557869
6 -0.682332 -0.312404 2.344647
6 1.081531 2.854056 1.078944
6 0.221706 -1.183096 3.193078
6 0.857982 -2.357843 2.403319
6 -0.911907 -3.726871 1.089849
1 -2.871837 -3.032845 0.421177
1 -2.917672 -4.137753 1.782279
1 1.008133 -3.159556 0.430621
1 -3.047082 5.554380 1.076236
1 -2.075093 -2.368097 3.284413
1 -3.572858 -1.912381 2.476192
1 -1.020714 4.330656 0.350743
1 3.883717 -1.048547 -3.021142
1 2.861194 0.439201 0.520488
1 4.425399 0.260043 -0.307033
1 2.530735 -2.081058 0.298018
1 3.955986 -2.136227 -0.756194
1 4.288092 3.989677 0.803284
1 2.458753 3.733990 2.483916
1 -2.603115 -0.034415 1.485840
1 1.793039 -0.094935 -4.599009
1 -0.527887 0.766567 2.407896
1 0.299804 2.736993 1.831788
1 1.015664 -0.555737 3.623753
1 -0.366416 -1.562016 4.046899
1 0.871635 -3.264127 3.036936
1 1.912980 -2.093298 2.233991
1 -0.626740 -4.457800 1.871401
7 0.691211 -0.380382 -2.752149
7 2.307956 -1.127845 -1.535227
15 -0.658753 1.487541 -0.639606
6 1.893562 2.581979 -1.182505
6 -3.337470 1.852984 -0.068949
6 3.119280 3.140201 -0.806135
6 -3.130670 4.512917 0.757266
6 -0.570168 0.233876 -3.173121
6 -0.833675 1.578703 -2.492147
1 1.769945 2.243978 -2.211623
6 -4.480218 2.550192 0.327152
1 -3.422566 0.814788 -0.400259
1 3.914997 3.237753 -1.547852
6 -4.377245 3.880444 0.748133
1 -0.520747 0.363666 -4.261312

1 -1.393933 -0.450644 -2.960527
1 -1.871686 1.874273 -2.697386
1 -0.188468 2.371717 -2.894762
1 -5.451986 2.051766 0.311853
1 -5.269529 4.424391 1.066145
8 4.263568 -0.714550 1.525171
6 4.716214 0.341054 2.342007
1 5.493738 0.944723 1.835343
1 3.888452 1.019368 2.623080
1 5.145012 -0.099124 3.253143
77 -0.272117 -0.770646 0.145984
1 0.981702 -0.196855 0.848263
1 -0.908777 -4.313345 0.154337
8 -1.913641 -1.777414 -1.215629
6 -1.652336 -2.769799 -1.876916
1 -0.640459 -3.198988 -1.961899
8 -2.540580 -3.441605 -2.567979
1 -3.421227 -3.028956 -2.467695

F-py$_{PCO-FA}$

6 -0.963179 -1.083008 1.494567
6 1.791557 -2.834924 -2.310487
6 -0.671972 -2.465427 -1.342137
6 1.095095 -0.302079 -2.284914
6 -0.799908 2.916809 0.267657
6 1.981465 2.300134 0.120475
6 -2.616205 5.013624 -0.168251
6 -1.409122 5.258234 0.498100
6 -3.225449 -1.941594 0.815521
6 1.707061 -1.484500 -3.022964
6 2.086511 2.892504 -1.151347
6 -4.124859 -0.722399 0.636565
6 -2.370061 -1.765983 3.142227
6 -1.282804 -1.218629 3.741709
6 -0.227588 0.095464 -2.476590
6 -0.504437 4.217884 0.712841
6 -1.206489 -0.763245 -3.251475
6 -1.627737 -2.037423 -2.480550
6 0.454970 -3.412172 -1.818169
1 2.495319 -2.751850 -1.474876
1 2.254342 -3.555641 -3.005568
1 -1.265710 -3.084778 -0.653447
1 3.378513 3.832851 -2.599378
1 1.154455 -1.624757 -3.967782
1 2.727444 -1.187350 -3.313346
1 1.201117 2.987524 -1.784139
1 -3.266463 -2.215154 3.558890
1 -3.493709 0.178825 0.516776
1 -4.757015 -0.562425 1.533204
1 -2.776058 -2.189947 -0.144581
1 -3.800272 -2.809897 1.164263
1 -3.321764 5.830067 -0.338477
1 -1.171201 6.264930 0.849282
1 1.802829 0.480409 -2.003043
1 -1.032142 -1.092000 4.790445
1 -0.468033 1.157593 -2.388881
1 0.439574 4.421147 1.223512
1 -2.096228 -0.164516 -3.495418
1 -0.742406 -1.031121 -4.216803
1 -1.777725 -2.869779 -3.193678
1 -2.616553 -1.833557 -2.041771
1 0.026991 -4.033981 -2.628556
7 -0.435644 -0.800321 2.725369
7 -2.162623 -1.672318 1.773661
15 0.379124 1.547969 0.599898
6 -2.009150 2.680304 -0.400593
6 3.130671 2.202926 0.920815
6 -2.913403 3.725172 -0.617837
6 3.313700 3.372996 -1.610752
6 0.705524 0.078814 2.985066
6 0.433211 1.488859 2.457439
1 -2.241314 1.675236 -0.753115
6 4.359241 2.684143 0.457973
1 3.086986 1.742504 1.908654

1 -3.851203 3.527670 -1.142207
6 4.454966 3.267551 -0.807918
1 0.844533 0.107084 4.072895
1 1.616528 -0.349098 2.554428
1 1.184399 2.193091 2.840147
1 -0.546982 1.829587 2.821495
1 5.243971 2.600497 1.092967
1 5.415489 3.642279 -1.168259
8 -4.910183 -0.945705 -0.505977
6 -5.780231 0.121560 -0.803345
1 -6.499774 0.303389 0.018104
1 -5.224543 1.061436 -0.986772
1 -6.338371 -0.142971 -1.712292
77 -0.112582 -0.635463 -0.329572
1 -1.572956 -0.143655 -0.660771
1 0.686556 -4.123924 -1.008933
6 1.684424 -2.702564 1.167121
6 3.001260 -1.019311 0.288608
6 2.791731 -3.336436 1.724144
1 0.683972 -3.119072 1.279646
6 4.157367 -1.584263 0.818589
1 3.052352 -0.107704 -0.301380
6 4.054968 -2.767141 1.552760
1 2.655707 -4.263087 2.283031
1 5.118166 -1.096726 0.649190
1 4.942263 -3.238486 1.980014
7 1.781921 -1.559238 0.464377

TS-FG$_{PCO\text{-}FA}$

6 -1.322160 -0.236219 1.477291
6 1.170101 -4.229938 0.086286
6 -1.106730 -2.955043 -0.130094
6 1.729388 -1.900933 -0.969374
6 0.096523 2.387957 -0.046752
6 2.851010 1.373476 -0.015645
6 -1.664202 4.309578 -1.094613
6 -0.891922 3.521976 -1.956369
6 -3.513772 -0.172799 0.258792
6 2.172661 -3.100726 -0.164311
6 3.194692 2.514329 -0.754831
6 -3.256630 0.883160 -0.807138
6 -3.098922 0.605754 2.605725
6 -2.015586 0.711877 3.420772
6 0.733761 -1.804400 -1.936891
6 -0.026391 2.559618 -1.438053
6 -0.095391 -2.965477 -2.483365
6 -1.255660 -3.440354 -1.579469
6 -0.160482 -3.818864 0.724236
1 1.654335 -4.958116 0.757085
1 0.975675 -4.782195 -0.845065
1 -2.086298 -2.971457 0.368578
1 4.778028 3.619025 -1.717042
1 3.071958 -3.518654 -0.656524
1 2.530012 -2.723747 0.810653
1 2.434381 3.247739 -1.023466
1 -4.137087 0.884037 2.759544
1 -2.181727 0.880639 -1.064629
1 -3.501475 1.890953 -0.418549
1 -3.333431 -1.168421 -0.165636
1 -4.559146 -0.139203 0.591684
1 -2.350711 5.055000 -1.501657
1 -0.975058 3.647890 -3.037847
1 2.489131 -1.117694 -1.007693
1 -1.915260 1.095556 4.431603
1 0.798600 -0.933037 -2.595376
1 0.545798 1.926689 -2.119709
1 -0.501719 -2.656940 -3.456927
1 0.590749 -3.799832 -2.696032
1 -1.346853 -4.540180 -1.600629
1 -2.202341 -3.046803 -1.981643
1 -0.724827 -4.745748 0.946233
7 -0.942416 0.189481 2.714960
7 -2.660951 0.018486 1.425360
15 1.146062 1.017455 0.554718

6 -0.684993 3.174370 0.810854
6 3.856515 0.454106 0.336836
6 -1.562530 4.129132 0.286103
6 4.521571 2.725779 -1.143084
6 0.447124 0.180855 3.163867
6 1.301914 1.196800 2.402240
1 -0.635493 3.046096 1.892195
6 5.179720 0.671230 -0.048712
1 3.610118 -0.434407 0.923521
1 -2.170830 4.730080 0.965447
6 5.514252 1.806669 -0.794835
1 0.457989 0.398846 4.239272
1 0.845940 -0.833077 3.018700
1 2.359142 1.022319 2.643781
1 1.073153 2.228871 2.702195
1 5.950469 -0.049666 0.232364
1 6.549187 1.975187 -1.100683
8 -4.046835 0.554175 -1.921495
6 -3.844088 1.417098 -3.015810
1 -4.081986 2.467574 -2.759315
1 -2.796648 1.385349 -3.372180
1 -4.507175 1.090688 -3.829531
77 -0.059276 -0.966660 0.045382
1 -1.373836 -1.547614 -0.620021
1 0.020194 -3.337968 1.699896

G$_{PCO-FA}$

6 1.599565 0.245038 1.115878
6 -2.909221 -0.179007 0.264991
6 -1.191817 2.139181 -0.016102
6 -5.386991 -1.299221 -0.422638
6 -4.839517 -1.532353 0.842543
6 3.640599 -0.141985 -0.322991
6 -0.033524 2.640205 -0.628809
6 3.187686 0.427033 -1.656579
6 3.486305 1.415135 1.618499
6 2.514119 1.837857 2.462732
6 -3.602717 -0.979561 1.185476
1 0.921151 4.353270 -1.529210
1 0.836144 1.996496 -0.763199
1 4.524706 1.715877 1.519728
1 2.136477 0.134303 -1.848428
1 3.810975 -0.017741 -2.457570
1 4.706100 0.074183 -0.168874
1 3.505513 -1.228433 -0.322721
1 -6.352171 -1.735201 -0.689992
1 -5.375435 -2.149593 1.567025
1 2.529242 2.580753 3.254530
1 -3.181896 -1.181143 2.172193
77 0.378508 -1.000776 0.187811
7 1.365344 1.119549 2.151852
7 2.924498 0.439780 0.811685
15 -1.226712 0.425112 0.635448
6 -3.463590 0.053310 -1.004357
6 -2.298466 2.986013 0.167489
6 -4.696621 -0.506364 -1.345476
6 0.015282 3.970985 -1.054574
6 0.107448 1.484589 2.826822
6 -1.132861 0.679333 2.460430
1 -2.930231 0.670133 -1.730274
6 -2.247770 4.312701 -0.263617
1 -3.206101 2.606972 0.642827
1 -5.120272 -0.321142 -2.335074
6 -1.090188 4.806929 -0.875842
1 0.281815 1.414522 3.910569
1 -0.075176 2.544652 2.593922
1 -2.014452 1.219402 2.837318
1 -1.111482 -0.319231 2.919349
1 -3.113812 4.963125 -0.121624
1 -1.051511 5.845266 -1.213141
6 -1.154514 -2.092578 -2.095296
1 -1.787986 -2.711810 -2.749776
8 -0.890113 -2.430130 -0.938816
8 -0.717223 -0.993911 -2.635073

1 -0.158772 -0.546789 -1.896019
8 3.313617 1.828210 -1.630306
6 2.976144 2.440622 -2.853411
1 3.624668 2.088019 -3.678181
1 1.923815 2.241137 -3.133044
1 3.111718 3.524864 -2.735074
8 1.862599 -2.661863 0.072662
6 1.588997 -3.852111 -0.022930
1 0.561372 -4.229925 -0.145789
8 2.486499 -4.802410 0.001524
1 3.379159 -4.417872 0.109232

TS-GH$_{PCO\text{-}FA}$

6 1.559944 0.292483 1.152331
6 -2.931202 -0.247553 0.224052
6 -1.245333 2.086524 -0.050834
6 -5.390231 -1.370384 -0.514276
6 -4.931574 -1.492062 0.800385
6 3.634971 -0.051867 -0.252370
6 -0.085206 2.600916 -0.648747
6 3.216722 0.492644 -1.607907
6 3.393275 1.540797 1.649213
6 2.398098 1.939514 2.478784
6 -3.703327 -0.936314 1.170731
1 0.849406 4.318451 -1.560355
1 0.801268 1.976190 -0.757445
1 4.419773 1.880598 1.552967
1 2.191676 0.157033 -1.854041
1 3.895411 0.070423 -2.375421
1 4.691174 0.183894 -0.067679
1 3.518091 -1.140197 -0.240578
1 -6.349621 -1.806711 -0.801147
1 -5.530995 -2.022050 1.543881
1 2.377393 2.695720 3.257667
1 -3.355007 -1.046414 2.199056
7 1.281572 1.171966 2.170154
7 2.876725 0.531410 0.854816
15 -1.272297 0.387108 0.629639
6 -3.394237 -0.129447 -1.097093
6 -2.375092 2.910396 0.092653
6 -4.618959 -0.689382 -1.462923
6 -0.057158 3.924760 -1.096884
6 0.004659 1.491667 2.833047
6 -1.204085 0.645549 2.452165
1 -2.793556 0.397381 -1.841659
6 -2.343345 4.230608 -0.359033
1 -3.284844 2.518512 0.553154
1 -4.973127 -0.593545 -2.491695
6 -1.183559 4.739454 -0.954635
1 0.169267 1.424614 3.918309
1 -0.210698 2.545074 2.599396
1 -2.106137 1.155494 2.821912
1 -1.156710 -0.352829 2.911096
1 -3.226090 4.864022 -0.246654
1 -1.159643 5.772677 -1.308465
8 3.284492 1.897793 -1.584121
6 2.971867 2.490658 -2.823698
1 1.940123 2.247876 -3.142784
1 3.060332 3.579856 -2.706887
1 3.665990 2.160013 -3.619834
77 0.378467 -1.012572 0.226550
6 -0.872037 -2.029697 -2.162775
1 -1.430021 -2.615829 -2.914053
8 -0.805196 -2.438875 -0.983163
8 -0.323459 -0.946946 -2.558517
1 0.182092 -0.569946 -1.515422
8 1.919259 -2.622526 0.129110
6 1.680442 -3.808231 -0.071871
1 0.673037 -4.190134 -0.301700
8 2.594885 -4.740589 -0.045083
1 3.468208 -4.349457 0.156938

H$_{PCO-FA}$

6 1.543084 -0.166679 0.920012
6 -2.954986 0.414189 -0.067629
6 -0.794076 2.367434 0.050596
6 -5.557960 -0.078950 -0.967485
6 -4.636971 0.557503 -1.807472
6 3.557726 -0.604738 -0.550955
6 -1.759788 3.368007 0.259433
6 3.528248 0.440880 -1.655517
6 3.568813 0.512532 1.672582
6 2.644623 0.924030 2.574097
6 -3.337580 0.800809 -1.362658
1 -2.188417 5.481103 0.220409
1 -2.770590 3.099654 0.573328
1 4.646680 0.634437 1.648487
1 2.500739 0.551728 -2.053022
1 4.161871 0.079233 -2.489354
1 4.596599 -0.827822 -0.273841
1 3.093489 -1.529340 -0.905649
1 -6.574747 -0.270023 -1.317676
1 -4.931652 0.863299 -2.813593
1 2.747931 1.476210 3.503176
1 -2.619769 1.293513 -2.022404
77 0.119907 -1.024507 -0.199462
7 1.404839 0.507813 2.102714
7 2.884831 -0.153652 0.668600
15 -1.225119 0.638143 0.432341
6 -3.877241 -0.227739 0.771405
6 0.499863 2.727088 -0.357088
6 -5.177078 -0.470649 0.318819
6 -1.432159 4.709829 0.059782
6 0.194801 0.969817 2.808620
6 -1.150221 0.505874 2.263910
1 -3.592378 -0.548859 1.773743
6 0.822807 4.072532 -0.550286
1 1.262322 1.967304 -0.519346
1 -5.893477 -0.967505 0.976395
6 -0.140508 5.063768 -0.344457
1 0.285187 0.654564 3.857805
1 0.230003 2.069315 2.792230
1 -1.934435 1.117697 2.733317
1 -1.342057 -0.550487 2.500246
1 1.831871 4.344210 -0.865114
1 0.114668 6.114344 -0.499884
1 0.723873 -0.166459 -1.343393
8 -1.282603 -2.573537 0.986779
6 -1.854790 -2.773937 -0.115012
1 -2.704547 -3.482613 -0.183286
8 -1.476994 -2.177666 -1.167983
8 3.994796 1.664974 -1.147587
6 4.058996 2.686899 -2.115908
1 4.766486 2.434925 -2.928733
1 3.068287 2.879511 -2.570422
1 4.404311 3.601778 -1.614540
8 1.361616 -2.813009 -0.686117
6 1.338691 -3.864293 -0.052575
1 0.681884 -4.028472 0.816825
8 2.079801 -4.895591 -0.348054
1 2.633413 -4.702077 -1.131031

I$_{PCO-FA}$

6 1.224981 -1.006053 1.098482
6 -2.107156 2.119816 0.398158
6 0.811363 2.265025 0.109755
6 -4.553501 3.432449 0.029347
6 -3.364284 4.167046 0.068810
6 3.004025 -1.735940 -0.511008
6 2.001372 2.410777 0.836720
6 3.629186 -0.567359 -1.251271
6 3.320500 -1.205526 1.920852
6 2.530627 -0.754866 2.931975
6 -2.142511 3.515978 0.256419
1 4.011945 3.190371 0.850448
1 2.104579 1.987656 1.836371

1 4.382356 -1.422436 1.881542
1 2.890002 0.248743 -1.372879
1 3.912955 -0.914861 -2.264444
1 3.751202 -2.530069 -0.373955
1 2.166986 -2.142523 -1.084185
1 -5.506800 3.944412 -0.120296
1 -3.385991 5.253090 -0.045316
1 2.757674 -0.516557 3.966640
1 -1.219697 4.097090 0.290782
77 -0.345449 -0.886950 -0.112126
7 1.248799 -0.641029 2.411874
7 2.505247 -1.357917 0.808304
15 -0.555407 1.220318 0.724317
6 -3.304424 1.385032 0.367110
6 0.729843 2.788978 -1.191724
6 -4.522426 2.042618 0.183757
6 3.088006 3.089059 0.277573
6 0.081841 -0.132401 3.132179
6 -0.413172 1.208651 2.579200
1 -3.281859 0.301316 0.488359
6 1.813052 3.473939 -1.742672
1 -0.182041 2.658381 -1.778076
1 -5.450005 1.466544 0.157950
6 2.993868 3.628259 -1.007421
1 -0.715663 -0.881022 3.064495
1 0.362110 -0.027269 4.187523
1 0.224868 2.044943 2.897220
1 -1.420736 1.402986 2.971649
1 1.736892 3.885362 -2.751470
1 3.841785 4.163057 -1.440750
1 0.604933 -0.274657 -1.173367
8 -0.117205 -2.891249 -0.801852
6 -0.088473 -3.212372 -2.052367
1 -0.034935 -4.320687 -2.198874
8 -0.108399 -2.462738 -3.022054
8 -1.893640 -1.840999 1.353453
6 -2.324904 -2.973969 1.191949
1 -1.947574 -3.649308 0.407197
8 -3.267272 -3.503424 1.932248
1 -3.557421 -2.859692 2.608537
8 4.754867 -0.122230 -0.534415
6 5.532148 0.821546 -1.234445
1 5.965484 0.385859 -2.155675
1 4.939333 1.709507 -1.517618
1 6.350331 1.139214 -0.572817
8 -2.074741 -0.845833 -1.450733
6 -1.998052 -0.508612 -2.628144
1 -1.121586 -0.012784 -3.065326
8 -2.975358 -0.675082 -3.477643
1 -3.724250 -1.123662 -3.037395

TS-IJ$_{PCO\text{-}FA}$

6 -1.393240 0.259218 -1.597977
6 0.670447 -2.119026 0.439011
6 3.072887 -0.832466 -0.620065
6 -0.508725 -3.629930 2.484379
6 0.674840 -2.922080 2.727756
6 -3.453921 0.720439 -0.235358
6 3.786946 -2.034701 -0.477617
6 -3.860270 -0.420188 0.680758
6 -3.372968 -0.415840 -2.464360
6 -2.396983 -0.813595 -3.323456
6 1.261998 -2.164888 1.711166
1 5.731554 -2.952485 -0.306988
1 3.255895 -2.985945 -0.400520
1 -4.448750 -0.549527 -2.485910
1 -4.241458 0.003095 1.632151
1 -2.973088 -1.034575 0.921390
1 -2.807985 1.427323 0.291541
1 -4.350196 1.261371 -0.571729
1 -0.967719 -4.220050 3.280734
1 1.137252 -2.950919 3.716722
1 -2.448082 -1.349410 -4.266497
1 2.157153 -1.576896 1.918851

77 0.111027 1.011105 -0.394634
7 -1.194541 -0.388903 -2.778082
7 -2.740921 0.240114 -1.416787
15 1.257270 -0.881325 -0.767170
6 -0.515199 -2.829658 0.197543
6 3.766468 0.381083 -0.717678
6 -1.098693 -3.585811 1.215841
6 5.181531 -2.016235 -0.424431
6 0.120963 -0.660338 -3.351982
6 0.950827 -1.584278 -2.462656
1 -1.010627 -2.778502 -0.773727
6 5.162735 0.393548 -0.669049
1 3.212537 1.314037 -0.824272
1 -2.020608 -4.137165 1.020279
6 5.870794 -0.802139 -0.519643
1 -0.034844 -1.128267 -4.332103
1 0.639428 0.294266 -3.514746
1 1.932681 -1.751626 -2.926115
1 0.465558 -2.565122 -2.364735
1 5.697889 1.342747 -0.743123
1 6.962239 -0.789567 -0.476069
1 0.790114 1.630135 -1.653480
8 -4.845730 -1.192251 0.037741
6 -5.182867 -2.367197 0.736367
1 -4.305896 -3.029698 0.866654
1 -5.594532 -2.143573 1.739583
1 -5.945881 -2.899677 0.151507
8 -0.861828 0.149331 1.529559
6 -0.919076 0.496030 2.697337
1 -0.725011 1.525362 3.044199
8 -1.212943 -0.323046 3.677881
1 -1.310610 -1.233351 3.326738
8 2.446427 1.015153 2.264999
6 1.764336 1.934132 1.923029
8 1.248946 2.978088 2.215301
1 1.423053 1.684982 0.486184
8 -1.010821 2.837225 0.110451
6 -0.499681 3.945316 0.245207
1 0.539611 4.174123 -0.030099
8 -1.146377 4.975285 0.715938
1 -2.059882 4.718545 0.952291
6 -1.393240 0.259218 -1.597977
6 0.670447 -2.119026 0.439011
6 3.072887 -0.832466 -0.620065
6 -0.508725 -3.629930 2.484379
6 0.674840 -2.922080 2.727756
6 -3.453921 0.720439 -0.235358
6 3.786946 -2.034701 -0.477617
6 -3.860270 -0.420188 0.680758
6 -3.372968 -0.415840 -2.464360
6 -2.396983 -0.813595 -3.323456
6 1.261998 -2.164888 1.711166
1 5.731554 -2.952485 -0.306988
1 3.255895 -2.985945 -0.400520
1 -4.448750 -0.549527 -2.485910
1 -4.241458 0.003095 1.632151
1 -2.973088 -1.034575 0.921390
1 -2.807985 1.427323 0.291541
1 -4.350196 1.261371 -0.571729
1 -0.967719 -4.220050 3.280734
1 1.137252 -2.950919 3.716722
1 -2.448082 -1.349410 -4.266497
1 2.157153 -1.576896 1.918851
77 0.111027 1.011105 -0.394634
7 -1.194541 -0.388903 -2.778082
7 -2.740921 0.240114 -1.416787
15 1.257270 -0.881325 -0.767170
6 -0.515199 -2.829658 0.197543
6 3.766468 0.381083 -0.717678
6 -1.098693 -3.585811 1.215841
6 5.181531 -2.016235 -0.424431
6 0.120963 -0.660338 -3.351982
6 0.950827 -1.584278 -2.462656
1 -1.010627 -2.778502 -0.773727

6 5.162735 0.393548 -0.669049
1 3.212537 1.314037 -0.824272
1 -2.020608 -4.137165 1.020279
6 5.870794 -0.802139 -0.519643
1 -0.034844 -1.128267 -4.332103
1 0.639428 0.294266 -3.514746
1 1.932681 -1.751626 -2.926115
1 0.465558 -2.565122 -2.364735
1 5.697889 1.342747 -0.743123
1 6.962239 -0.789567 -0.476069
1 0.790114 1.630135 -1.653480
8 -4.845730 -1.192251 0.037741
6 -5.182867 -2.367197 0.736367
1 -4.305896 -3.029698 0.866654
1 -5.594532 -2.143573 1.739583
1 -5.945881 -2.899677 0.151507
8 -0.861828 0.149331 1.529559
6 -0.919076 0.496030 2.697337
1 -0.725011 1.525362 3.044199
8 -1.212943 -0.323046 3.677881
1 -1.310610 -1.233351 3.326738
8 2.446427 1.015153 2.264999
6 1.764336 1.934132 1.923029
8 1.248946 2.978088 2.215301
1 1.423053 1.684982 0.486184
8 -1.010821 2.837225 0.110451
6 -0.499681 3.945316 0.245207
1 0.539611 4.174123 -0.030099
8 -1.146377 4.975285 0.715938
1 -2.059882 4.718545 0.952291

TS-IJ$_{PCO\text{-}FA}$b

6 -1.600785 -0.321804 1.102636
6 2.282387 1.279314 0.758322
6 2.731933 -1.534224 0.207284
6 3.364691 3.859765 0.579492
6 3.896678 2.822553 -0.192140
6 -3.561052 0.960564 0.206010
6 3.908066 -1.632122 0.973327
6 -4.511952 0.062294 -0.563686
6 -3.378366 -0.080283 2.480595
6 -2.451700 -0.828281 3.137689
6 3.357790 1.535923 -0.107179
1 5.822885 -2.595451 1.203399
1 4.051588 -1.000667 1.853306
1 -4.369987 0.250716 2.769654
1 -3.952759 -0.786689 -1.001482
1 -4.945309 0.639211 -1.405300
1 -4.126285 1.766683 0.694845
1 -2.832335 1.418687 -0.469570
1 3.784384 4.865390 0.508063
1 4.733564 3.013857 -0.867011
1 -2.460931 -1.268370 4.130406
1 3.771861 0.733859 -0.720169
77 -0.378512 -0.268967 -0.562327
7 -1.373786 -0.967853 2.275679
7 -2.835730 0.229719 1.242819
15 1.447550 -0.342934 0.722967
6 1.746550 2.326412 1.527065
6 2.579387 -2.337051 -0.930924
6 2.289064 3.609731 1.438238
6 4.912117 -2.526976 0.604727
6 -0.141290 -1.682118 2.600798
6 1.078636 -0.772297 2.482453
1 0.889323 2.155201 2.180785
6 3.588429 -3.233655 -1.295796
1 1.674462 -2.258398 -1.533034
1 1.864521 4.418216 2.036753
6 4.752484 -3.330863 -0.530149
1 -0.235200 -2.045537 3.631321
1 -0.049640 -2.561830 1.946085
1 1.961439 -1.265292 2.911637
1 0.906494 0.154097 3.047348
1 3.461069 -3.856975 -2.183422

1 5.539584 -4.031743 -0.817202
1 -0.349778 -1.827963 -0.516428
8 -1.813129 -0.239858 -2.345671
6 -0.821959 -0.469879 -3.013234
1 0.509345 -0.236775 -2.020292
8 -0.333343 -0.749164 -4.046951
8 -5.512794 -0.390286 0.314407
6 -6.380738 -1.338661 -0.259297
1 -6.921847 -0.924173 -1.131989
1 -5.834982 -2.242714 -0.592550
1 -7.114350 -1.629416 0.505768
8 -0.601805 2.023472 -0.717017
6 0.198167 2.827219 -1.173467
1 1.178497 2.546073 -1.592392
8 -0.013983 4.117497 -1.218380
1 -0.887593 4.324770 -0.830993

J$_{PCO\text{-}FA}$

6 1.691024 -0.315397 -0.997277
6 -2.104680 1.485755 -0.776353
6 -2.828756 -1.318986 -0.513882
6 -2.940289 4.148534 -0.442979
6 -3.592013 3.118316 0.239830
6 3.609032 0.971619 0.009614
6 -4.034366 -1.142542 -1.216761
6 4.680173 0.077890 0.606125
6 3.410712 0.224163 -2.369641
6 2.507307 -0.471904 -3.106774
6 -3.175709 1.793619 0.078813
1 -6.035150 -1.872345 -1.559593
1 -4.143471 -0.314481 -1.921174
1 4.374886 0.647981 -2.627920
1 4.224171 -0.870885 0.950083
1 5.110917 0.580285 1.495754
1 4.070196 1.886700 -0.388873
1 2.881683 1.260447 0.772889
1 -3.264356 5.183374 -0.313336
1 -4.427902 3.343602 0.905785
1 2.510426 -0.764375 -4.152344
1 -3.685676 0.998166 0.624960
7 1.462915 -0.799949 -2.252237
7 2.894476 0.319845 -1.085004
15 -1.410484 -0.205515 -0.819731
6 -1.444100 2.528995 -1.447830
6 -2.706507 -2.384502 0.389183
6 -1.863819 3.850575 -1.285055
6 -5.101221 -2.017678 -1.012062
6 0.238350 -1.468198 -2.684356
6 -0.971398 -0.536854 -2.592189
1 -0.583739 2.320111 -2.086778
6 -3.777548 -3.261111 0.590287
1 -1.773524 -2.525585 0.934163
1 -1.342529 4.651391 -1.814009
6 -4.973974 -3.078687 -0.107192
1 0.390798 -1.786901 -3.723150
1 0.088787 -2.372852 -2.077569
1 -1.838295 -0.990500 -3.092147
1 -0.749999 0.410852 -3.102329
1 -3.674149 -4.088524 1.295822
1 -5.810461 -3.762986 0.052458
1 0.447160 -2.010900 0.314095
8 5.666058 -0.161069 -0.368785
6 6.647066 -1.086304 0.034557
1 7.358776 -1.205078 -0.794619
1 7.198213 -0.736069 0.928956
1 6.205314 -2.074129 0.269795
77 0.491591 -0.470747 0.574294
8 -0.839540 -0.582015 2.343355
6 -0.410094 -0.953182 3.440006
1 -1.079572 -1.024709 4.312695
8 0.818822 -1.283785 3.675822
1 1.336867 -1.144921 2.811210
1 1.846364 -0.741210 1.499915
8 0.594384 1.797437 1.027698

6 -0.248367 2.431299 1.643910
1 -1.108334 1.969518 2.154833
8 -0.230087 3.735518 1.772017
1 0.533569 4.103770 1.285743

TS-JI$_{PCO\text{-}FA}$

6 1.463447 -1.266928 -0.738017
6 -1.356303 1.916089 -0.691689
6 -3.066934 -0.408960 -0.708938
6 -1.306571 4.682505 -0.245708
6 -1.691753 3.814525 0.783355
6 3.615978 -0.919885 0.530863
6 -4.046967 0.369068 -1.350104
6 4.464482 0.325715 0.346562
6 3.398944 -1.597129 -1.869484
6 2.377231 -1.817476 -2.736523
6 -1.719865 2.437866 0.564512
1 -6.142984 0.590626 -1.807301
1 -3.766647 1.283539 -1.877846
1 4.474946 -1.623016 -1.997012
1 4.878204 0.616761 1.332436
1 3.834871 1.166583 -0.006189
1 2.929872 -0.767656 1.368401
1 4.267168 -1.773381 0.771769
1 -1.279341 5.760101 -0.070408
1 -1.965164 4.212458 1.762880
1 2.378493 -2.098102 -3.785453
1 -1.997948 1.757667 1.372677
77 0.088937 -0.905442 0.690472
7 1.201792 -1.615660 -2.028102
7 2.824561 -1.254585 -0.651269
15 -1.315751 0.098857 -0.836738
6 -0.978150 2.788054 -1.722349
6 -3.443921 -1.570593 -0.020400
6 -0.955400 4.168835 -1.495879
6 -5.386660 -0.018898 -1.307950
6 -0.130733 -1.678227 -2.624451
6 -0.821890 -0.315650 -2.565013
1 -0.697987 2.408886 -2.705345
6 -4.787403 -1.954051 0.018731
1 -2.691291 -2.166103 0.494991
1 -0.658753 4.841847 -2.302982
6 -5.758521 -1.181773 -0.623855
1 -0.005848 -1.986665 -3.669307
1 -0.721228 -2.455190 -2.117850
1 -1.718068 -0.308454 -3.200554
1 -0.128943 0.450647 -2.938840
1 -5.074683 -2.858710 0.559066
1 -6.808127 -1.482441 -0.588645
1 -0.576755 -2.251608 0.280188
6 6.272311 1.190764 -0.890593
1 5.660367 1.991671 -1.348139
1 6.769400 1.606448 0.006631
1 7.041506 0.876157 -1.609282
8 5.492592 0.060006 -0.573751
8 1.076197 1.015149 1.512807
6 1.381006 2.056680 0.951255
1 1.318602 2.211162 -0.137893
8 1.828231 3.116168 1.576278
1 1.862311 2.945263 2.538539
8 -1.399666 -0.485198 2.184116
6 -1.127967 -0.831298 3.380441
1 -1.903788 -0.573280 4.131759
8 -0.097015 -1.416890 3.754080
1 0.676721 -1.636124 2.429747
1 1.179863 -1.860463 1.717453

COD

6 -1.920611 0.005548 -0.012824
6 0.016321 -1.695740 -0.226489
6 -0.016325 1.695750 -0.226489
6 -1.078381 1.099995 0.668249
6 1.211210 1.229669 -0.502875
6 1.920626 -0.005560 -0.012834

6 1.078367 -1.099997 0.668281
6 -1.211207 -1.229673 -0.502885
1 -2.446331 0.451732 -0.876555
1 -2.729624 -0.303218 0.677085
1 0.309785 -2.621377 -0.736860
1 -0.645865 0.714385 1.600682
1 -1.767094 1.904136 0.972198
1 -0.309740 2.621416 -0.736831
1 1.814822 1.832980 -1.192984
1 2.446350 -0.451762 -0.876551
1 2.729629 0.303241 0.677086
1 0.645841 -0.714320 1.600672
1 1.767044 -1.904153 0.972231
1 -1.814811 -1.833011 -1.192977

cis-COE

6 1.816783 0.358339 -0.147920
6 -0.372854 1.757144 -0.081823
6 0.336132 -1.777181 0.028208
6 1.314196 -0.805590 0.715871
6 -0.805695 -1.171830 -0.798783
6 -1.845474 -0.343935 -0.038610
6 -1.297130 0.879501 0.729729
6 0.897383 1.517876 -0.445853
1 2.181849 -0.039594 -1.115269
1 2.722623 0.780570 0.326540
1 -0.813812 2.690295 -0.452978
1 0.888531 -0.412938 1.650252
1 2.194719 -1.391912 1.027840
1 0.922891 -2.422235 -0.649658
1 -1.334181 -1.999723 -1.302344
1 -2.602322 0.002519 -0.763876
1 -2.381291 -0.990832 0.678645
1 -0.797445 0.536500 1.646131
1 -2.153304 1.483570 1.069360
1 1.375883 2.288769 -1.064410
1 -0.085215 -2.452671 0.794391
1 -0.378974 -0.558252 -1.609538

FA

6 0.000000 0.417805 0.000000
1 -0.372068 1.463319 0.000000
8 1.161387 0.100898 0.000000
8 -1.029876 -0.429547 0.000000
1 -0.680024 -1.340958 0.000000

H_2

1 0.000000 0.000000 0.380606
1 0.000000 0.000000 -0.380606

CO_2

6 0.000000 0.000000 0.000000
8 0.000000 0.000000 1.162967
8 0.000000 0.000000 -1.162967

Py

6 1.200571 0.672627 0.000003
6 -0.000154 1.386325 -0.000047
6 -1.200705 0.672412 0.000026
6 -1.144856 -0.724820 -0.000014
6 1.145026 -0.724569 0.000049
1 2.164723 1.185124 0.000023
1 -0.000221 2.478938 -0.000012
1 -2.164994 1.184651 0.000096
1 -2.072335 -1.309492 0.000091
1 2.072587 -1.309102 0.000043
7 0.000135 -1.417424 -0.0000

Cartesian coordinates (xyz format) for 4-based reaction pathways in dimethyl carbonate

A*PCO-DMC*

6 -1.637356 -0.751479 1.103802
6 1.251419 -3.776363 0.342782
6 -0.821259 -2.970426 -1.001904
6 2.000421 -1.698518 -0.923709
6 0.166371 2.496805 -0.282928
6 2.555850 1.200576 0.652332
6 -0.569197 4.743176 -1.785852
6 -0.716199 3.462989 -2.325789
6 -3.563840 -0.825946 -0.471319
6 2.250636 -3.173524 -0.661649
6 3.221597 1.872152 -0.383859
6 -4.166545 0.543613 -0.759323
6 -3.700995 -0.611172 2.022815
6 -2.800308 -0.355749 3.008466
6 1.231609 -1.180090 -1.965363
6 -0.356835 2.341421 -1.574635
6 0.455435 -1.981062 -2.986128
6 -0.267320 -3.211719 -2.398213
6 -0.149859 -3.233622 0.201427
1 1.589345 -3.561778 1.368749
1 1.231260 -4.878569 0.251286
1 -1.911137 -2.932685 -0.936069
1 5.128185 2.426600 -1.228897
1 2.230877 -3.731917 -1.607392
1 3.268875 -3.289645 -0.262547
1 2.650824 2.378676 -1.164045
1 -4.786266 -0.641047 2.045655
1 -5.048808 0.718496 -0.110669
1 -4.526479 0.565172 -1.807685
1 -2.753308 -1.032490 -1.179382
1 -4.330692 -1.609163 -0.561807
1 -0.853722 5.619820 -2.372060
1 -1.121919 3.334985 -3.331591
1 2.722680 -1.021464 -0.465951
1 -2.939115 -0.134444 4.062656
1 1.444627 -0.147318 -2.264271
1 -0.493363 1.338856 -1.982330
1 -0.290127 -1.307189 -3.437631
1 1.123146 -2.290388 -3.811709
1 0.402082 -4.083318 -2.389920
1 -1.100306 -3.480752 -3.064124
1 -0.776104 -3.381335 1.087972
77 -0.034154 -1.050229 -0.127646
7 -1.543693 -0.444336 2.426655
7 -2.973236 -0.851117 0.863779
15 0.733391 1.020729 0.627066
6 0.311601 3.785469 0.256711
6 3.307103 0.552362 1.647414
6 -0.061902 4.903541 -0.491140
6 4.618827 1.899304 -0.419260
6 -0.296887 -0.015170 3.056120
6 0.219418 1.274021 2.404315
1 0.728329 3.922132 1.257299
6 4.701753 0.586775 1.611396
1 2.808766 0.004076 2.450066
1 0.051624 5.903443 -0.066665
6 5.360777 1.259201 0.576450
1 -0.502761 0.155339 4.121154
1 0.439436 -0.827098 2.978349
1 1.072587 1.670715 2.972067
1 -0.577059 2.032477 2.419666
1 5.276690 0.083549 2.391907
1 6.452273 1.282964 0.547432
8 -3.180922 1.510473 -0.522704
6 -3.648424 2.838268 -0.559618
1 -4.017621 3.116874 -1.564807
1 -4.467454 2.996294 0.168245
1 -2.805873 3.492187 -0.301651

TS-AB$_{PCO-DMC}$

6 -1.771601 -1.629049 -0.356765
6 -1.259149 3.043806 -0.687758
6 1.017554 1.784138 -0.685510
6 0.363892 4.987290 -0.245300
6 1.396377 -1.167001 1.987611
6 2.748604 -0.894761 -0.591976
6 1.744984 -1.121968 4.771391
6 0.796436 -0.289859 4.172273
6 -3.756787 -0.368670 0.502270
6 -0.807259 4.181922 0.257969
6 3.843934 -0.393986 0.124697
6 -3.130917 0.333698 1.702472
6 -3.555449 -2.874034 0.254260
6 -2.592462 -3.729379 -0.188038
6 1.665606 4.700274 -0.097915
6 0.623744 -0.313495 2.786096
6 2.308035 3.519582 0.589140
6 1.492258 2.212292 0.678198
6 -0.157473 2.179093 -1.285209
1 -1.809637 3.478211 -1.535045
1 -2.005360 2.431843 -0.152608
1 1.800502 1.411285 -1.351130
1 5.885822 0.297405 0.029046
1 -0.591075 3.776848 1.255163
1 -1.672544 4.848827 0.395572
1 3.774035 -0.235124 1.201209
1 -4.557661 -3.062383 0.627752
1 -2.029243 0.349901 1.575980
1 -3.339131 -0.218279 2.641055
1 -3.736101 0.311430 -0.357805
1 -4.801242 -0.636548 0.705205
1 1.882337 -1.104357 5.854752
1 0.187980 0.380087 4.783624
1 0.114603 5.895715 -0.805537
1 -2.584777 -4.811764 -0.274886
1 2.380570 5.412232 -0.526357
1 -0.114975 0.336999 2.318858
1 3.251637 3.295637 0.062309
1 2.615149 3.808392 1.612047
1 0.654050 2.317342 1.374490
1 2.148994 1.445127 1.106616
1 -0.184608 2.083500 -2.373842
77 -0.591458 -0.042070 -0.715616
7 -1.513512 -2.944172 -0.562876
7 -3.039182 -1.591375 0.143470
15 1.120232 -1.228651 0.179266
6 2.345360 -2.006925 2.597808
6 2.864053 -1.091813 -1.979488
6 2.518618 -1.980798 3.982175
6 5.038700 -0.094341 -0.538144
6 -0.232249 -3.422659 -1.073012
6 0.897628 -3.069813 -0.112685
1 2.958072 -2.680104 1.993986
6 4.059721 -0.799377 -2.635341
1 2.012550 -1.459342 -2.556668
1 3.260065 -2.633546 4.447830
6 5.149442 -0.296347 -1.915436
1 -0.302849 -4.512117 -1.188459
1 -0.071805 -2.989707 -2.070403
1 1.848516 -3.470608 -0.490057
1 0.701515 -3.531364 0.865563
1 4.139281 -0.957316 -3.712991
1 6.083557 -0.061474 -2.430210
8 -3.641470 1.636465 1.742405
6 -3.155886 2.395857 2.824963
1 -3.428315 1.941842 3.796711
1 -2.053344 2.496727 2.787377
1 -3.602978 3.396863 2.759430
6 -2.049285 0.091493 -3.198679
1 -2.792657 0.254993 -4.000935
8 -2.284106 0.562542 -2.059832
8 -1.004002 -0.564147 -3.496122
1 -0.415210 -0.579073 -2.350318

B$_{PCO\text{-}DMC}$

6 0.478580 -1.219948 1.163960
6 3.389069 1.567738 -1.379863
6 2.872280 -0.852734 -0.532376
6 5.650494 1.156124 -0.291597
6 -2.689042 -0.126455 0.491623
6 -1.807671 2.665125 0.335833
6 -4.683815 -1.988870 -0.150864
6 -4.350035 -0.978310 -1.058909
6 0.245758 -3.221920 -0.332321
6 4.334178 1.898222 -0.215620
6 -3.152446 3.050911 0.461080
6 -1.197599 -3.317237 -0.812601
6 0.102437 -3.209620 2.170269
6 0.080983 -2.256066 3.139261
6 5.947150 -0.110317 0.036054
6 -3.356311 -0.052110 -0.743396
6 5.100146 -1.199932 0.633967
6 3.596210 -0.931633 0.792763
6 2.791130 0.185333 -1.428492
1 3.939158 1.692885 -2.329440
1 2.570180 2.302640 -1.435357
1 2.574061 -1.825676 -0.933155
1 -4.570721 4.666535 0.283644
1 3.819068 1.744677 0.742415
1 4.546293 2.976858 -0.264198
1 -3.909734 2.326082 0.764975
1 -0.022222 -4.286968 2.226734
1 -1.616030 -2.300571 -0.892395
1 -1.816424 -3.879999 -0.084038
1 0.811257 -2.646890 -1.070984
1 0.694054 -4.221519 -0.250788
1 -5.460780 -2.714052 -0.401617
1 -4.864305 -0.912727 -2.019830
1 6.471248 1.739616 -0.724708
1 -0.061793 -2.330372 4.213187
1 6.982803 -0.423845 -0.143229
1 -3.099766 0.729208 -1.460388
1 5.244162 -2.117292 0.034198
1 5.511827 -1.451482 1.629135
1 3.435322 -0.034352 1.398323
1 3.162745 -1.766113 1.363210
1 2.392257 -0.085438 -2.410770
77 0.622288 0.214897 -0.216802
7 0.312227 -1.043240 2.504108
7 0.341628 -2.560422 0.965725
15 -1.279706 0.987307 0.787622
6 -3.021883 -1.146842 1.395906
6 -0.840514 3.605133 -0.054025
6 -4.018891 -2.071060 1.074644
6 -3.524132 4.369156 0.189834
6 0.209073 0.274209 3.125327
6 -1.019188 1.050679 2.630440
1 -2.504956 -1.242448 2.351634
6 -1.220083 4.921636 -0.318960
1 0.201629 3.302611 -0.158068
1 -4.271777 -2.860441 1.785455
6 -2.559993 5.303731 -0.200394
1 0.155585 0.131180 4.212017
1 1.127310 0.833101 2.905081
1 -0.898132 2.112767 2.885135
1 -1.940520 0.706127 3.120943
1 -0.467120 5.649913 -0.627255
1 -2.854532 6.333447 -0.414769
8 -1.164759 -3.953652 -2.060209
6 -2.341423 -3.772827 -2.812168
1 -3.231028 -4.176541 -2.290234
1 -2.520395 -2.701986 -3.022930
1 -2.216322 -4.310669 -3.762467
6 -0.245941 0.586689 -2.658015
1 -0.599204 0.808368 -3.684532
8 0.296396 1.514087 -1.983169
8 -0.381793 -0.563308 -2.170413

1 1.379345 1.052484 0.857838

C$_{PCO\text{-}DMC}$

6 -1.016709 -0.985727 1.390882
6 2.568019 -3.263384 0.927212
6 0.548378 -3.106629 -0.666911
6 2.933765 -1.085386 -0.412978
6 -1.140771 2.140356 0.352100
6 1.839236 2.471677 0.191743
6 -3.706816 3.158681 -0.223365
6 -2.736561 3.080560 -1.224936
6 -2.921079 -1.848248 -0.033291
6 3.498022 -2.426815 0.025670
6 1.664892 3.532114 -0.708359
6 -3.689715 -0.683124 -0.646658
6 -3.023689 -1.288288 2.385351
6 -2.200643 -0.725650 3.307736
6 2.237398 -0.880381 -1.584768
6 -1.467751 2.568857 -0.945945
6 1.885286 -1.964055 -2.573977
6 1.327800 -3.267616 -1.960046
6 1.097869 -3.140056 0.604219
1 2.706688 -2.963363 1.976193
1 2.853514 -4.329453 0.873716
1 -0.527000 -3.270334 -0.740096
1 2.608497 5.129854 -1.802265
1 3.772306 -3.000680 -0.870536
1 4.437333 -2.245720 0.567592
1 0.680020 3.770760 -1.102335
1 -4.061796 -1.599624 2.450580
1 -3.011176 0.182724 -0.735124
1 -4.533270 -0.379321 0.008191
1 -2.150265 -2.147178 -0.744674
1 -3.587686 -2.695924 0.176905
1 -4.701029 3.550834 -0.447754
1 -2.966832 3.409243 -2.240686
1 3.355739 -0.203263 0.066552
1 -2.369146 -0.449328 4.344152
1 2.123849 0.141153 -1.952393
1 -0.749847 2.463871 -1.755275
1 1.163561 -1.550415 -3.286810
1 2.788962 -2.195592 -3.166096
1 2.143233 -3.984781 -1.785318
1 0.663008 -3.739935 -2.696269
1 0.422711 -3.367045 1.434132
7 -0.978402 -0.541416 2.682000
7 -2.287083 -1.437413 1.218292
15 0.483490 1.365958 0.719797
6 -2.124115 2.226539 1.354991
6 3.126076 2.208391 0.697614
6 -3.395671 2.725981 1.067105
6 2.759775 4.306510 -1.101132
6 0.167499 0.126215 3.298781
6 0.595500 1.414789 2.578076
1 -1.924806 1.902067 2.373633
6 4.215528 2.984896 0.303827
1 3.286488 1.398302 1.412545
1 -4.143602 2.776447 1.861167
6 4.035100 4.034494 -0.602327
1 -0.109106 0.354558 4.335873
1 1.003545 -0.583556 3.329268
1 1.648017 1.605778 2.823026
1 0.032629 2.289049 2.930384
1 5.207125 2.769681 0.707404
1 4.887605 4.641564 -0.914161
8 -4.149062 -1.103244 -1.901443
6 -4.817873 -0.091072 -2.615031
1 -5.711650 0.272216 -2.070398
1 -4.157735 0.776590 -2.806026
1 -5.137880 -0.514294 -3.577328
77 0.618033 -0.908244 0.147088
6 -0.648489 0.043264 -2.586746
1 -1.461697 -0.136028 -3.333456
8 -0.745393 -0.738750 -1.574146

8 0.193284 0.915090 -2.789134
1 1.485026 -0.967735 1.455680

TS-CD$_{PCO\text{-}DMC}$

6 -1.785071 -1.621153 -0.346291
6 -1.230603 3.045800 -0.703002
6 1.034274 1.770433 -0.699152
6 0.415326 4.982009 -0.303913
6 1.392511 -1.147052 2.004116
6 2.734100 -0.928163 -0.587501
6 1.748732 -1.063429 4.785662
6 0.803648 -0.234235 4.176971
6 -3.762691 -0.338838 0.497725
6 -0.764167 4.200077 0.216854
6 3.838401 -0.427032 0.115007
6 -3.135277 0.362184 1.698095
6 -3.583217 -2.847037 0.261841
6 -2.624901 -3.713186 -0.169327
6 1.714042 4.683236 -0.152996
6 0.627015 -0.277279 2.791832
6 2.343946 3.506507 0.552210
6 1.513939 2.209407 0.659229
6 -0.137330 2.171827 -1.300594
1 -1.797734 3.463705 -1.547336
1 -1.965672 2.441012 -0.144972
1 1.813479 1.386443 -1.362905
1 5.885617 0.244966 -0.003655
1 -0.552568 3.814462 1.223010
1 -1.621364 4.879815 0.341684
1 3.776802 -0.252356 1.189620
1 -4.589138 -3.024409 0.630621
1 -2.032707 0.365788 1.578215
1 -3.354822 -0.184060 2.637471
1 -3.733761 0.336824 -0.365571
1 -4.810281 -0.596729 0.697751
1 1.889220 -1.030750 5.868272
1 0.201008 0.448283 4.780094
1 0.175613 5.884153 -0.878251
1 -2.626406 -4.796030 -0.250210
1 2.436202 5.380133 -0.593927
1 -0.109022 0.370228 2.316115
1 3.286204 3.264907 0.030835
1 2.652403 3.807106 1.571329
1 0.676173 2.335684 1.352511
1 2.161194 1.440645 1.098932
1 -0.167885 2.068360 -2.388355
77 -0.592912 -0.045233 -0.710549
7 -1.536763 -2.939691 -0.542221
7 -3.054690 -1.569811 0.147976
15 1.107508 -1.234624 0.198744
6 2.337452 -1.984443 2.623847
6 2.838406 -1.145301 -1.972812
6 2.514652 -1.938893 4.007233
6 5.031345 -0.147207 -0.559725
6 -0.253973 -3.434673 -1.032486
6 0.868870 -3.077610 -0.065279
1 2.943799 -2.670478 2.028129
6 4.032208 -0.872606 -2.640451
1 1.979690 -1.512769 -2.539150
1 3.253007 -2.589581 4.480602
6 5.131071 -0.369247 -1.934792
1 -0.331971 -4.524929 -1.134750
1 -0.079158 -3.016171 -2.033697
1 1.819453 -3.493066 -0.427511
1 0.659907 -3.524173 0.917312
1 4.103132 -1.045987 -3.716334
1 6.063642 -0.149591 -2.458995
8 -3.633033 1.670136 1.730766
6 -3.145116 2.429410 2.812387
1 -3.426351 1.982237 3.784802
1 -2.041510 2.520675 2.779113
1 -3.583018 3.434078 2.740807
6 -2.042933 0.082773 -3.199104
1 -2.783004 0.247211 -4.004263

8 -2.276763 0.563545 -2.064002
8 -1.002629 -0.583551 -3.489278
1 -0.416922 -0.595534 -2.339897

D*PCO-DMC*

6 -1.459360 -0.371581 1.067995
6 1.397924 2.509015 -0.101073
6 3.100817 0.358134 0.726378
6 1.598790 5.018416 -1.326971
6 1.672043 4.912703 0.064736
6 -3.614916 -0.371816 -0.283084
6 3.914587 0.606699 -0.391023
6 -4.623646 0.751680 -0.450929
6 -3.401364 -0.044553 2.186357
6 -2.395652 0.008790 3.096004
6 1.576380 3.662547 0.679248
1 5.867660 0.385409 -1.275618
1 3.517018 1.143622 -1.253630
1 -4.476521 0.069743 2.275031
1 -4.978916 0.729025 -1.499407
1 -4.143721 1.735945 -0.301739
1 -2.923496 -0.355560 -1.127389
1 -4.159788 -1.328549 -0.268598
1 1.666042 5.998299 -1.804336
1 1.804245 5.806768 0.677627
1 -2.410290 0.171439 4.169547
1 1.647722 3.599315 1.766187
7 -1.216191 -0.199300 2.396999
7 -2.815475 -0.266511 0.945601
15 1.339035 0.865127 0.678047
6 1.335321 2.618236 -1.499787
6 3.642448 -0.317902 1.831737
6 1.433759 3.870346 -2.106824
6 5.243965 0.181081 -0.402907
6 0.108699 -0.098286 2.996615
6 0.856941 1.114841 2.442089
1 1.167661 1.733568 -2.113633
6 4.973963 -0.739939 1.816635
1 3.039247 -0.524480 2.716787
1 1.366784 3.948713 -3.193566
6 5.776271 -0.494208 0.699420
1 -0.029510 0.008212 4.079569
1 0.654296 -1.034989 2.820885
1 1.752973 1.332980 3.038938
1 0.200152 1.994630 2.491182
1 5.384064 -1.261211 2.684100
1 6.817388 -0.823508 0.690091
8 -5.695204 0.557058 0.443550
6 -6.677947 1.565455 0.362962
1 -6.261650 2.561428 0.607055
1 -7.121594 1.616604 -0.649399
1 -7.468584 1.320828 1.085528
6 -0.670313 -3.976061 -0.095360
6 -1.051337 -2.230358 -1.927767
6 1.203912 -2.264485 0.316722
6 0.548293 -3.565927 0.752360
6 1.773701 -2.092804 -1.089777
6 1.387666 -3.074267 -2.204460
6 0.050810 -2.693080 -2.857467
6 -1.377352 -2.796351 -0.718834
1 -1.388798 -4.521676 0.532325
1 -0.371260 -4.671408 -0.892269
1 -1.760950 -1.532242 -2.378202
1 1.286066 -4.388126 0.785054
1 0.221512 -3.415372 1.793756
1 1.964235 -1.997219 1.057427
1 2.855306 -1.900650 -1.048046
1 2.165920 -3.059584 -2.981140
1 1.381038 -4.097160 -1.804895
1 -0.343621 -3.536673 -3.452530
1 0.231459 -1.881074 -3.578563
1 -2.330542 -2.517889 -0.271910
77 -0.038506 -0.728562 -0.313404
8 -1.059349 0.714366 -1.619462

6 -1.582840 1.842084 -1.256904
1 -1.376821 2.135037 -0.192660
8 -2.275849 2.560229 -1.956543
1 1.450225 -1.044862 -1.517280

$E_{PCO\text{-}DMC}$

6 1.899508 -0.373930 -1.070499
6 -1.131379 2.634719 -0.539952
6 -2.589668 0.448452 -1.629434
6 -1.693517 5.185457 0.478258
6 -1.692542 4.968124 -0.903237
6 3.816256 -0.347252 0.639444
6 -3.646628 0.856868 -0.800943
6 4.835738 0.752716 0.889356
6 4.030423 -0.119288 -1.822349
6 3.212014 -0.038326 -2.897873
6 -1.418281 3.698017 -1.412997
1 -5.771096 0.798126 -0.434631
1 -3.448603 1.495895 0.060056
1 5.106888 -0.041355 -1.713429
1 4.989247 0.823941 1.983069
1 4.444914 1.733290 0.563385
1 3.008055 -0.250792 1.366642
1 4.324324 -1.315214 0.770098
1 -1.905227 6.180933 0.874590
1 -1.908558 5.790524 -1.588591
1 3.427080 0.113679 -3.951034
1 -1.439823 3.545509 -2.493620
7 1.917370 -0.192411 -2.425860
7 3.221605 -0.304267 -0.707058
15 -0.875242 0.957120 -1.217466
6 -1.138179 2.856106 0.845236
6 -2.872945 -0.342377 -2.754775
6 -1.417219 4.128485 1.348878
6 -4.956996 0.465751 -1.082452
6 0.754830 -0.009099 -3.286433
6 -0.061552 1.206425 -2.855590
1 -0.884971 2.048462 1.527843
6 -4.183885 -0.736816 -3.030991
1 -2.081379 -0.653431 -3.436434
1 -1.404162 4.294144 2.428189
6 -5.228448 -0.338757 -2.192898
1 1.131617 0.143139 -4.305326
1 0.156885 -0.927223 -3.296731
1 -0.829085 1.437347 -3.606759
1 0.600880 2.080248 -2.769524
1 -4.387965 -1.352022 -3.909838
1 -6.253914 -0.643700 -2.412240
8 6.045749 0.433970 0.237505
6 7.046538 1.410408 0.416196
1 6.737504 2.391863 0.007931
1 7.292468 1.545916 1.486641
1 7.946169 1.069532 -0.114657
6 1.506555 -3.601542 -0.301487
6 0.574705 -2.381535 1.772218
6 -0.534166 -2.209222 -1.085888
6 0.589816 -3.182747 -1.475677
6 -1.714528 -2.919347 -0.401786
6 -1.387768 -3.829413 0.789983
6 -0.674523 -3.196900 1.995003
6 1.541220 -2.593329 0.823341
1 2.533335 -3.739392 -0.669496
1 1.196452 -4.575278 0.105345
1 0.847470 -1.718971 2.600830
1 0.161631 -4.085373 -1.948651
1 1.218319 -2.715117 -2.246863
1 -0.952245 -1.832838 -2.026786
1 -2.194490 -3.563078 -1.162542
1 -2.328514 -4.268413 1.162905
1 -0.802542 -4.688499 0.432920
1 -0.367022 -4.009833 2.680769
1 -1.384444 -2.607358 2.590640
1 2.502570 -2.136777 1.027248
77 0.253634 -0.591933 0.065026

8 1.151631 0.795176 1.501209
6 1.745808 1.905916 1.217009
1 1.612653 2.252643 0.156995
8 2.433774 2.563273 1.982759
1 -2.473226 -2.177489 -0.121999
8 -1.645319 -0.517907 1.269780
6 -1.935063 -0.230380 2.430750
8 -1.089634 0.353095 3.235240
8 -3.114550 -0.481262 2.957746
6 -4.074866 -1.233121 2.189069
1 -5.011629 -1.173036 2.752123
1 -4.188935 -0.800282 1.189436
1 -3.745350 -2.277324 2.108631
6 -1.454635 0.675280 4.590960
1 -2.301430 1.374135 4.599632
1 -1.717416 -0.237897 5.141117
1 -0.563346 1.143357 5.021026

TS-EF$_{PCO\text{-}DMC}$

6 1.523073 -0.166307 -1.151143
6 -1.143658 2.216475 -0.807028
6 -3.214751 0.200973 -0.993580
6 -0.796833 4.946362 -0.263845
6 -0.247013 4.386511 -1.418683
6 3.459225 0.456424 0.381074
6 -3.736242 -1.100956 -0.967754
6 4.696659 -0.426581 0.413113
6 3.321100 0.905702 -2.046029
6 2.447082 0.675289 -3.056100
6 -0.415180 3.025507 -1.689308
1 -5.505244 -2.329549 -0.938766
1 -3.069438 -1.958404 -0.930767
1 4.306083 1.358223 -2.028173
1 4.452840 -1.449125 0.062136
1 5.044601 -0.512944 1.462320
1 3.772506 1.496145 0.538423
1 2.763052 0.201305 1.181522
1 -0.673578 6.012484 -0.060454
1 0.312394 5.010573 -2.118999
1 2.498380 0.906715 -4.115609
1 0.027762 2.613036 -2.596267
7 1.358563 0.023327 -2.495679
7 2.746636 0.387187 -0.892559
15 -1.399578 0.430467 -1.099577
6 -1.665582 2.776398 0.373255
6 -4.096676 1.292251 -1.058350
6 -1.494373 4.135134 0.637815
6 -5.115491 -1.310236 -0.974881
6 0.227878 -0.445304 -3.291554
6 -1.116273 0.174674 -2.914260
1 -2.201013 2.143770 1.082576
6 -5.476703 1.077864 -1.065275
1 -3.716237 2.312051 -1.102043
1 -1.911321 4.562507 1.552293
6 -5.989592 -0.221027 -1.017612
1 0.459174 -0.214297 -4.338940
1 0.178080 -1.535975 -3.210154
1 -1.918415 -0.495020 -3.252648
1 -1.283150 1.137016 -3.417687
1 -6.153135 1.934339 -1.107657
1 -7.069551 -0.384134 -1.016113
8 5.683139 0.153822 -0.400523
6 6.858926 -0.616479 -0.506905
1 7.328293 -0.780165 0.481874
1 6.658627 -1.604972 -0.963202
1 7.561965 -0.065694 -1.146477
6 -0.217459 -3.472794 2.267010
6 1.743369 -2.371046 0.984847
6 -1.296168 -3.578188 -0.066478
6 -0.706187 -4.354164 1.113005
6 -0.374547 -2.629239 -0.853441
6 0.959337 -3.276112 -1.257244
6 1.947787 -3.426298 -0.080811
6 0.781880 -2.384582 1.970965

1 -1.089488 -3.016210 2.751669
1 0.250815 -4.109834 3.042490
1 2.557805 -1.669752 1.130078
1 0.083423 -5.037296 0.771967
1 -1.496834 -5.006646 1.517087
1 -2.163914 -3.026442 0.313753
1 1.443243 -2.655259 -2.024187
1 0.777093 -4.257459 -1.731944
1 1.856093 -4.419769 0.376758
1 2.980424 -3.359907 -0.453199
1 0.856375 -1.632202 2.762501
1 -1.691336 -4.315760 -0.790085
1 -0.918844 -2.401026 -1.781329
77 0.041536 -0.835319 0.183394
8 -3.038205 -1.870349 2.061022
6 -2.501781 -0.803662 2.029274
8 -2.538732 0.350451 2.352866
1 -1.248817 -0.972341 1.331705
8 0.536442 0.897570 1.735824
6 0.866086 2.065900 1.880401
8 0.631687 2.761412 2.974551
8 1.528761 2.723761 0.950669
6 -0.114479 2.107601 4.022608
1 0.523637 1.352991 4.503502
1 -0.365654 2.890434 4.745935
1 -1.012106 1.627459 3.613136
6 2.039263 4.042072 1.211086
1 1.243766 4.703229 1.571073
1 2.849425 3.989777 1.952348
1 2.420420 4.400728 0.249244

TS-EF$_{PCO\text{-}DMC}$b

6 -0.885620 -1.342313 0.924779
6 -1.643048 1.827199 0.404377
6 1.120882 2.761221 0.397944
6 -4.219007 2.723016 -0.265007
6 -3.753455 2.845684 1.047358
6 -2.201666 -2.405972 -0.926562
6 2.476886 2.735699 0.767395
6 -3.639454 -2.288493 -1.395901
6 -2.989904 -2.002584 1.437255
6 -2.477712 -1.394451 2.536888
6 -2.471595 2.406473 1.380992
1 4.365116 3.774296 0.724069
1 2.888675 1.884165 1.311781
1 -3.965081 -2.446184 1.273076
1 -4.296764 -2.987030 -0.838241
1 -3.670063 -2.614003 -2.455125
1 -1.556010 -1.787588 -1.562386
1 -1.890363 -3.456893 -1.023471
1 -5.219778 3.075389 -0.525108
1 -4.387296 3.291986 1.816595
1 -2.909917 -1.212947 3.516061
1 -2.132635 2.529376 2.408958
7 -1.191898 -0.989634 2.208151
7 -2.008653 -1.955577 0.457570
15 0.074343 1.319532 0.777007
6 -2.124377 1.691153 -0.907673
6 0.615598 3.856666 -0.316783
6 -3.401629 2.145824 -1.238223
6 3.313203 3.799488 0.432041
6 -0.330759 -0.240937 3.127810
6 0.141207 1.132970 2.610781
1 -1.505680 1.229112 -1.674681
6 1.461709 4.916123 -0.656661
1 -0.433902 3.885475 -0.611697
1 -3.759677 2.034212 -2.263220
6 2.807175 4.890292 -0.283845
1 -0.903674 -0.109566 4.053113
1 0.534469 -0.865028 3.375349
1 1.187956 1.292411 2.900096
1 -0.424128 1.955265 3.066028
1 1.063484 5.765108 -1.216231
1 3.465195 5.719460 -0.552439

8 -4.068052 -0.966307 -1.243864
6 -5.412805 -0.761849 -1.608098
1 -5.587065 -0.993071 -2.676257
1 -6.097293 -1.387083 -1.002608
1 -5.647243 0.295448 -1.428421
8 0.062441 0.097347 -2.512499
6 1.189970 0.550755 -2.473972
1 1.826785 0.206882 -1.206015
8 2.025331 1.171947 -3.045406
6 3.315870 -2.087659 -1.619044
6 1.108108 -2.988528 -0.556037
6 3.607227 -0.912812 0.667265
6 4.120272 -1.960728 -0.321766
6 2.199124 -1.126296 1.243037
6 1.974976 -2.538541 1.797299
6 1.681293 -3.592518 0.709369
6 1.822324 -2.299732 -1.523480
1 3.492389 -1.201546 -2.247761
1 3.710863 -2.941412 -2.201362
1 0.136758 -3.369291 -0.864953
1 4.194319 -2.935877 0.180444
1 5.154035 -1.699560 -0.598452
1 3.672884 0.088685 0.214064
1 1.121717 -2.522875 2.488821
1 2.848692 -2.840576 2.401522
1 2.588874 -4.158352 0.453194
1 0.963693 -4.328244 1.098689
1 1.303282 -2.168404 -2.478540
77 0.729491 -0.692489 -0.203937
1 4.302860 -0.900574 1.527525
1 2.095889 -0.414831 2.070468

$F_{PCO-DMC}$

6 -0.331086 -1.310853 1.331160
6 3.096752 -1.211340 -2.351418
6 0.760551 -2.178924 -1.519723
6 1.230771 0.599689 -2.328173
6 -2.271171 1.627059 0.663025
6 0.142489 3.256618 0.198363
6 -5.051649 1.301908 0.358484
6 -4.296711 1.894423 -0.659572
6 -1.950508 -2.995420 0.396826
6 2.307017 -0.141020 -3.101924
6 -0.675106 4.393979 0.127584
6 -3.214876 -2.236929 0.004612
6 -1.542963 -2.537217 2.811434
6 -0.824057 -1.636298 3.529213
6 -0.119475 0.350080 -2.516702
6 -2.918023 2.052429 -0.511388
6 -0.609621 -0.826597 -3.332141
6 -0.179940 -2.205804 -2.755340
6 2.249238 -2.400677 -1.865791
1 3.610267 -0.729208 -1.511188
1 3.897070 -1.583287 -3.011952
1 0.501948 -3.058251 -0.911528
1 -0.773116 6.506897 -0.300062
1 1.856430 -0.596730 -3.998704
1 3.012949 0.620795 -3.472001
1 -1.737349 4.324136 0.366449
1 -2.235896 -3.311125 3.127324
1 -2.976243 -1.163720 -0.111606
1 -3.984625 -2.309676 0.800522
1 -1.292902 -3.058224 -0.470520
1 -2.189577 -4.017810 0.719539
1 -6.129890 1.175956 0.239924
1 -4.783546 2.235190 -1.576042
1 1.511033 1.582713 -1.946031
1 -0.755865 -1.466479 4.599587
1 -0.829862 1.163164 -2.352754
1 -2.343842 2.510152 -1.319266
1 -1.707077 -0.777842 -3.384957
1 -0.248969 -0.704398 -4.368828
1 0.269337 -2.818752 -3.556982
1 -1.106545 -2.725873 -2.468235

1 2.282159 -3.184726 -2.646720
7 -0.094485 -0.892556 2.613084
7 -1.233186 -2.326893 1.476180
15 -0.442030 1.604608 0.723391
6 -3.036072 1.025520 1.674996
6 1.514522 3.371750 -0.091713
6 -4.416835 0.868661 1.524093
6 -0.129331 5.626169 -0.243981
6 0.668988 0.296526 2.981699
6 -0.011047 1.595660 2.541916
1 -2.564072 0.651850 2.584774
6 2.056240 4.606909 -0.452293
1 2.156417 2.489547 -0.024407
1 -4.995387 0.397736 2.321918
6 1.233307 5.734980 -0.535750
1 0.792036 0.283155 4.072607
1 1.662532 0.233480 2.534742
1 0.683521 2.430533 2.708815
1 -0.908613 1.800846 3.142002
1 3.122514 4.688642 -0.675341
1 1.655117 6.699724 -0.826269
8 -3.672381 -2.785223 -1.201623
6 -4.769797 -2.090144 -1.745266
1 -5.642523 -2.105937 -1.063749
1 -4.517140 -1.032073 -1.951152
1 -5.045924 -2.583647 -2.687299
77 0.291614 -0.403918 -0.400643
1 -1.177897 -0.658514 -0.805928
1 2.748287 -2.847914 -0.991772
6 3.227044 -0.868725 0.982416
8 2.482519 -0.063731 0.437482
8 2.787510 -2.013036 1.455417
8 4.516707 -0.661453 1.165397
6 3.696236 -2.951436 2.060110
1 4.180951 -2.503063 2.937367
1 3.076339 -3.804117 2.356416
1 4.457033 -3.265176 1.333258
6 5.064332 0.589026 0.708616
1 6.132888 0.545326 0.942860
1 4.910437 0.698065 -0.372851
1 4.587306 1.424846 1.238209

TS-FG$_{PCO\text{-}DMC}$

6 1.324209 0.207051 1.481346
6 -1.090488 4.255669 0.075155
6 1.159848 2.932104 -0.110035
6 -1.680998 1.936991 -0.986792
6 -0.153564 -2.381914 -0.052961
6 -2.885621 -1.314502 -0.014610
6 1.557097 -4.339631 -1.115490
6 0.801627 -3.529472 -1.970949
6 3.516508 0.104921 0.268039
6 -2.112444 3.148047 -0.192063
6 -3.255465 -2.459403 -0.734661
6 3.243706 -0.945086 -0.800365
6 3.082581 -0.666354 2.615039
6 1.995926 -0.752650 3.428011
6 -0.677565 1.822981 -1.943240
6 -0.039340 -2.549743 -1.445377
6 0.182765 2.966316 -2.477457
6 1.340812 3.413546 -1.556986
6 0.221410 3.815360 0.731061
1 -1.568861 4.994210 0.738454
1 -0.870965 4.804133 -0.852533
1 2.132347 2.923857 0.402323
1 -4.864619 -3.539961 -1.681131
1 -2.992142 3.583874 -0.703573
1 -2.499714 2.784088 0.776721
1 -2.512517 -3.215574 -0.988716
1 4.115690 -0.961123 2.773382
1 2.167097 -0.931474 -1.052998
1 3.478975 -1.956781 -0.414210
1 3.354094 1.103352 -0.157033
1 4.561258 0.054697 0.601071

1 2.221756 -5.101546 -1.528027
1 0.876698 -3.654133 -3.053186
1 -2.454210 1.167362 -1.034009
1 1.888389 -1.130647 4.440525
1 -0.749693 0.951323 -2.600767
1 -0.600875 -1.901246 -2.121318
1 0.594977 2.651091 -3.446204
1 -0.482578 3.815116 -2.698176
1 1.458912 4.510693 -1.576532
1 2.283541 2.998013 -1.946171
1 0.802756 4.729307 0.961998
7 0.933813 -0.211214 2.719105
7 2.658401 -0.071553 1.433779
15 -1.175045 -0.993735 0.554272
6 0.611598 -3.190737 0.798161
6 -3.868555 -0.365018 0.320459
6 1.463906 -4.163904 0.266258
6 -4.586396 -2.644035 -1.121854
6 -0.455532 -0.175120 3.164401
6 -1.329562 -1.175257 2.403131
1 0.566678 -3.067661 1.880417
6 -5.195920 -0.555869 -0.063420
1 -3.599977 0.527353 0.891601
1 2.057050 -4.785744 0.940201
6 -5.556290 -1.695012 -0.791223
1 -0.473102 -0.387516 4.241174
1 -0.833155 0.847057 3.015435
1 -2.384177 -0.986201 2.646774
1 -1.116779 -2.211962 2.699831
1 -5.949940 0.187779 0.203226
1 -6.594369 -1.843004 -1.096598
8 4.034030 -0.617530 -1.911254
6 3.855933 -1.491983 -2.999456
1 4.113246 -2.535627 -2.733011
1 2.810507 -1.483201 -3.363772
1 4.518643 -1.158396 -3.810094
77 0.084419 0.960466 0.044359
1 1.413596 1.505847 -0.614280
1 0.018059 3.338627 1.704578

G*PCO-DMC*

6 -0.426054 1.539278 -1.017197
6 1.784035 -2.439394 -0.271857
6 2.783888 0.277856 -0.087679
6 2.277648 -5.116619 0.401406
6 1.694582 -4.799902 -0.828789
6 -1.784464 3.041392 0.491619
6 2.601421 1.540655 0.496066
6 -0.938775 3.039617 1.754481
6 -0.599148 3.735411 -1.590203
6 0.231386 3.150922 -2.486980
6 1.443293 -3.467075 -1.164069
1 3.557497 3.308187 1.279852
1 1.595118 1.917537 0.681491
1 -0.937678 4.762511 -1.496874
1 -0.580508 2.011419 1.963346
1 -1.579393 3.347879 2.604813
1 -2.227442 4.035271 0.343432
1 -2.593298 2.310259 0.593876
1 2.472166 -6.159258 0.662286
1 1.432239 -5.593740 -1.531582
1 0.763663 3.562254 -3.339476
1 0.975752 -3.235962 -2.122989
77 -0.694095 -0.143167 -0.020633
7 0.332991 1.809675 -2.132796
7 -1.002928 2.746585 -0.708772
15 1.333300 -0.706841 -0.623615
6 2.368797 -2.762494 0.963620
6 4.086707 -0.187773 -0.336126
6 2.613270 -4.096018 1.297040
6 3.709038 2.326256 0.827549
6 1.317128 0.970413 -2.837273
6 1.365885 -0.504648 -2.459197
1 2.633476 -1.971381 1.668115

6 5.189799 0.597812 0.001643
1 4.241217 -1.171240 -0.786152
1 3.071347 -4.338791 2.258507
6 5.002114 1.856445 0.583928
1 1.117794 1.065474 -3.915052
1 2.305378 1.417391 -2.648285
1 2.280354 -0.936646 -2.893181
1 0.498310 -1.049702 -2.857460
1 6.199344 0.226727 -0.188794
1 5.866387 2.470113 0.847937
6 -0.628151 -2.004315 2.280299
1 -0.771790 -2.869661 2.947111
8 -1.108453 -1.971913 1.147814
8 0.084003 -1.040618 2.790064
1 0.125607 -0.328081 2.061253
8 0.144020 3.918107 1.582491
6 0.940402 4.061868 2.734937
1 0.356832 4.460041 3.587047
1 1.389856 3.098410 3.044296
1 1.747317 4.769338 2.497920
8 -2.862855 0.146609 0.434681
6 -3.831795 -0.542191 0.119612
8 -4.103455 -0.973509 -1.093483
8 -4.711482 -0.888796 1.027895
6 -3.265489 -0.561473 -2.187762
1 -2.218745 -0.847704 -1.990933
1 -3.660605 -1.075707 -3.069852
1 -3.325868 0.528333 -2.314244
6 -5.875461 -1.652874 0.660392
1 -6.469044 -1.111163 -0.088223
1 -5.577601 -2.631590 0.260720
1 -6.446849 -1.775806 1.586567

TS-GH$_{PCO-DMC}$

6 -0.542208 1.404061 -1.321301
6 2.501228 -1.871502 -0.164162
6 2.553011 0.978242 0.344420
6 3.640165 -4.325450 0.561812
6 3.352917 -4.056978 -0.779288
6 -2.605173 2.304745 -0.172719
6 1.864675 2.060088 0.913980
6 -2.192389 2.301387 1.290491
6 -1.141711 3.554498 -1.754949
6 0.005372 3.312201 -2.434355
6 2.780602 -2.835347 -1.143771
1 2.030057 3.921814 1.989400
1 0.777759 2.115809 0.855708
1 -1.750639 4.450051 -1.681652
1 -1.667404 1.362250 1.541185
1 -3.113035 2.334330 1.908134
1 -3.235541 3.180524 -0.375910
1 -3.188219 1.406139 -0.397386
1 4.087495 -5.281091 0.844072
1 3.574444 -4.801324 -1.547219
1 0.600477 3.950967 -3.079909
1 2.552279 -2.643459 -2.193831
7 0.364875 1.996626 -2.166935
7 -1.472549 2.385010 -1.092791
15 1.651683 -0.317149 -0.583053
6 2.787675 -2.145933 1.183383
6 3.955741 0.929899 0.424135
6 3.355197 -3.369085 1.542349
6 2.573284 3.082187 1.551506
6 1.658644 1.507580 -2.674947
6 2.034675 0.068358 -2.343840
1 2.563461 -1.403211 1.951772
6 4.658067 1.951088 1.065573
1 4.501693 0.087276 -0.006624
1 3.578601 -3.575603 2.591431
6 3.967188 3.029435 1.629497
1 1.657389 1.642377 -3.766937
1 2.427773 2.178321 -2.262913
1 3.106117 -0.061335 -2.558993
1 1.468453 -0.647599 -2.957255

1 5.747552 1.904578 1.127621
1 4.518019 3.827979 2.131669
8 -1.370125 3.414046 1.533463
6 -0.951007 3.513137 2.875122
1 -0.357486 2.630625 3.182266
1 -0.325368 4.411930 2.965275
1 -1.811605 3.609492 3.564598
77 -0.547557 -0.406515 -0.498586
6 -0.472824 -2.112951 1.809409
1 -0.493031 -2.968147 2.509280
8 -0.664730 -2.316898 0.589784
8 -0.248603 -0.961310 2.304242
1 -0.308200 -0.273855 1.259491
8 -2.708117 -0.845173 -0.764980
6 -3.476867 -1.240191 0.111927
8 -4.509184 -2.013795 -0.121544
8 -3.321127 -0.881737 1.364702
6 -4.710032 -2.476302 -1.473011
1 -3.837303 -3.055753 -1.801756
1 -5.604031 -3.106881 -1.436367
1 -4.864310 -1.620308 -2.142921
6 -4.208910 -1.376909 2.386014
1 -5.233661 -1.028943 2.200439
1 -4.185355 -2.474212 2.408893
1 -3.824847 -0.963921 3.324478

H*PCO-DMC*
6 0.262581 1.528527 0.792694
6 -1.499670 -2.703197 0.054898
6 -2.854909 -0.117741 0.119951
6 -1.690955 -5.385098 -0.714758
6 -2.081520 -4.374567 -1.600528
6 1.168961 3.284810 -0.784233
6 -4.054688 -0.815234 0.345763
6 0.086921 3.498722 -1.832772
6 0.250819 3.681250 1.500424
6 -0.352692 2.938418 2.459853
6 -1.982014 -3.036794 -1.221370
1 -6.209218 -0.722161 0.347055
1 -4.032715 -1.864787 0.645808
1 0.440385 4.747928 1.437841
1 -0.304595 2.524973 -2.185156
1 0.547888 3.999206 -2.707547
1 1.676516 4.235565 -0.573079
1 1.913106 2.570299 -1.149667
1 -1.766442 -6.432573 -1.014678
1 -2.459376 -4.630150 -2.592633
1 -0.794933 3.221416 3.410228
1 -2.276350 -2.249230 -1.918575
77 0.628001 -0.096919 -0.307486
7 -0.345214 1.619856 2.016029
7 0.625206 2.809265 0.490258
15 -1.269776 -0.951185 0.457977
6 -1.101914 -3.716321 0.939172
6 -2.899843 1.228896 -0.275711
6 -1.201293 -5.055293 0.551599
6 -5.281557 -0.171965 0.175821
6 -1.112444 0.611656 2.768555
6 -1.026493 -0.828537 2.279586
1 -0.705124 -3.475056 1.925791
6 -4.131290 1.867778 -0.438009
1 -1.982762 1.788514 -0.454292
1 -0.894201 -5.842349 1.243512
6 -5.321493 1.170117 -0.215207
1 -0.776552 0.657903 3.814769
1 -2.163916 0.937145 2.747278
1 -1.790244 -1.412201 2.814697
1 -0.040249 -1.270084 2.486062
1 -4.157536 2.915148 -0.744007
1 -6.282430 1.671702 -0.348934
1 -0.136232 0.677782 -1.414174
8 1.896004 -1.787673 0.813448
6 1.841950 -2.427769 -0.271815
1 2.323215 -3.422558 -0.357159

8 1.246423 -1.953276 -1.280920
8 -0.942257 4.274639 -1.275827
6 -1.977401 4.574015 -2.183847
1 -1.608611 5.165567 -3.043220
1 -2.452118 3.654376 -2.577073
1 -2.733006 5.163422 -1.646149
8 2.723949 0.585187 -0.782430
6 3.762430 0.069042 -0.364563
8 4.447736 -0.752966 -1.116653
8 4.291725 0.300832 0.815301
6 5.647106 -1.371981 -0.611199
1 6.398220 -0.608316 -0.369894
1 5.413359 -1.963367 0.284183
1 6.002861 -2.018910 -1.419805
6 3.563169 1.121653 1.742857
1 3.299813 2.080802 1.279744
1 2.654648 0.595136 2.062916
1 4.235986 1.275251 2.592554

I*PCO-DMC*

6 1.753057 0.122995 -1.339652
6 -1.840407 2.204164 -0.491609
6 -2.796463 -0.348953 -1.435407
6 -2.690461 4.536863 0.814676
6 -2.525939 3.349847 1.538318
6 3.707747 0.498520 0.222083
6 -2.704274 -1.722082 -1.722458
6 4.850487 -0.494261 0.316029
6 3.692495 0.844467 -2.257437
6 2.791846 0.713999 -3.265177
6 -2.106925 2.189098 0.889972
1 -3.774814 -3.563531 -2.063141
1 -1.727108 -2.193777 -1.832239
1 4.738768 1.128258 -2.251342
1 4.502337 -1.507561 0.041034
1 5.178829 -0.546848 1.373073
1 4.090823 1.515747 0.394898
1 2.961646 0.258600 0.980385
1 -3.012123 5.447689 1.324079
1 -2.716598 3.332317 2.613345
1 2.884008 0.888809 -4.332870
1 -1.974984 1.265245 1.454705
77 0.345938 -0.464800 -0.076484
7 1.610587 0.265641 -2.690009
7 3.043400 0.479642 -1.084073
15 -1.263041 0.645920 -1.246295
6 -2.021190 3.391625 -1.214450
6 -4.064188 0.232382 -1.273247
6 -2.443642 4.554034 -0.559928
6 -3.859292 -2.497920 -1.838187
6 0.366835 0.071783 -3.429867
6 -0.717215 1.048022 -2.967194
1 -1.839747 3.425691 -2.289559
6 -5.217611 -0.549374 -1.384813
1 -4.155551 1.295946 -1.048766
1 -2.578491 5.475683 -1.129942
6 -5.118451 -1.914913 -1.663449
1 0.591426 0.236734 -4.491243
1 0.040057 -0.972340 -3.321527
1 -1.581174 1.018194 -3.645779
1 -0.305480 2.067530 -2.976722
1 -6.197882 -0.086776 -1.250601
1 -6.020776 -2.524673 -1.745705
1 0.101805 -1.682279 -1.001631
8 5.902444 -0.074134 -0.520668
6 6.980143 -0.979546 -0.568242
1 6.665502 -1.969532 -0.951223
1 7.740205 -0.561744 -1.243142
1 7.432934 -1.126925 0.431062
8 1.671814 -1.526356 1.139876
6 2.161464 -2.705519 0.879516
1 1.773951 -3.164038 -0.065934
8 2.972842 -3.284557 1.575586
6 -1.804562 -2.200757 1.344121

8 -1.281944 -1.084593 1.297847
8 -1.217139 -3.256325 0.845473
8 -2.978052 -2.419377 1.888295
6 -1.866437 -4.542076 0.893353
1 -2.817805 -4.503578 0.346628
1 -1.169278 -5.234837 0.411114
1 -2.043654 -4.838835 1.935138
6 -3.731312 -1.283814 2.354860
1 -4.661236 -1.696215 2.759144
1 -3.172993 -0.754673 3.137932
1 -3.941912 -0.608602 1.515163
6 0.806885 1.986446 2.344636
8 0.829483 1.346931 1.301139
8 0.926205 3.299992 2.391570
8 0.678345 1.502051 3.557220
6 0.574535 0.073666 3.719389
1 0.742096 -0.111056 4.785765
1 -0.430012 -0.256387 3.426039
1 1.314358 -0.451668 3.101194
6 1.096361 3.981799 1.141014
1 0.214277 3.840970 0.503971
1 1.215771 5.040191 1.395858
1 1.990402 3.610045 0.621263

TS-IJ$_{PCO\text{-}DMC}$

6 0.995536 -0.575163 1.751375
6 -2.987952 -0.683258 0.038461
6 -2.612126 2.085010 0.664299
6 -4.786106 -2.368270 -1.311095
6 -3.965967 -1.489951 -2.032885
6 2.824369 -2.046431 0.857562
6 -4.003508 2.172833 0.868322
6 4.252158 -1.536236 0.993420
6 2.082779 -1.892072 3.242973
6 1.184279 -1.149142 3.940184
6 -3.074410 -0.644003 -1.365534
1 -5.732703 3.457898 0.944821
1 -4.586142 1.271812 1.082881
1 2.799508 -2.634364 3.579759
1 4.841221 -1.920093 0.141627
1 4.715017 -1.943060 1.919987
1 2.832390 -3.145216 0.927825
1 2.393972 -1.764420 -0.106151
1 -5.485266 -3.019351 -1.835099
1 -4.023579 -1.459145 -3.120331
1 0.948769 -1.126162 5.000135
1 -2.438189 0.024415 -1.944164
77 0.279818 0.348464 0.035292
7 0.531992 -0.352370 3.014961
7 1.951328 -1.531929 1.906673
15 -1.799041 0.452868 0.827740
6 -3.813572 -1.557722 0.759345
6 -1.892486 3.248660 0.369955
6 -4.711906 -2.396515 0.084780
6 -4.654811 3.403841 0.787599
6 -0.584185 0.529569 3.341378
6 -1.870879 0.088681 2.639800
1 -3.774381 -1.599536 1.850349
6 -2.544214 4.480201 0.288866
1 -0.817566 3.185223 0.184669
1 -5.354848 -3.064095 0.652807
6 -3.925312 4.563089 0.498151
1 -0.722926 0.488221 4.429516
1 -0.320943 1.569889 3.084063
1 -2.741709 0.597236 3.071561
1 -1.996902 -0.989738 2.777665
1 -1.976310 5.380801 0.054701
1 -4.437213 5.525725 0.432487
1 0.499478 1.705501 0.755355
8 4.263813 -0.131638 1.026786
6 5.527556 0.413129 1.337264
1 5.836539 0.152510 2.367087
1 6.308326 0.043918 0.643521
1 5.458663 1.505530 1.245410

8 0.079697 -1.541904 -1.220730
8 -0.888148 0.850125 -3.409897
6 -0.079543 1.514499 -2.844386
8 0.794403 2.324649 -2.832353
1 -0.332848 1.141246 -1.341862
8 2.214117 0.283487 -0.994745
6 -0.546811 -2.576910 -1.024778
8 -1.028206 -3.326486 -2.001734
8 -0.782008 -3.029547 0.188448
6 -1.547730 -4.236530 0.371696
1 -1.650425 -4.349855 1.458300
1 -2.532559 -4.131596 -0.102041
1 -1.010851 -5.097320 -0.060737
6 -0.825747 -2.851902 -3.349551
1 -1.485462 -3.463136 -3.980783
1 -1.078446 -1.788018 -3.419351
1 0.232203 -2.993392 -3.642197
6 3.269146 0.804636 -1.327855
8 4.114975 0.113750 -2.072688
8 3.681987 2.022252 -1.048153
6 2.844877 2.895477 -0.271681
1 3.378318 3.852120 -0.239054
1 1.871995 3.012813 -0.765655
1 2.719658 2.488768 0.743214
6 5.320503 0.727874 -2.546026
1 5.841693 -0.056117 -3.116032
1 5.083204 1.581680 -3.199925
1 5.946034 1.066796 -1.708686

TS-IJ$_{PCO\text{-}DMC}$b

6 1.698013 -0.836204 -0.962146
6 -2.080903 0.721139 -1.301689
6 -2.729384 -1.796633 0.002678
6 -3.055408 3.242175 -2.049906
6 -3.741947 2.478232 -1.102443
6 3.553432 0.732199 -0.349420
6 -3.853006 -2.082464 -0.795067
6 4.560188 0.105717 0.598198
6 3.515909 -0.833626 -2.311233
6 2.654144 -1.770295 -2.790741
6 -3.255574 1.223956 -0.723767
1 -5.794212 -3.016263 -0.889080
1 -3.903557 -1.733465 -1.829459
1 4.505227 -0.532783 -2.638424
1 4.061918 -0.668953 1.212740
1 4.930533 0.886772 1.293000
1 4.062201 1.456876 -1.001643
1 2.774796 1.259245 0.210125
1 -3.437791 4.221767 -2.344636
1 -4.662269 2.858306 -0.653830
1 2.728841 -2.441158 -3.641552
1 -3.791603 0.638604 0.024562
77 0.404456 -0.476568 0.608708
7 1.550170 -1.758493 -1.949210
7 2.908820 -0.265003 -1.200911
15 -1.346804 -0.849375 -0.720892
6 -1.388898 1.496820 -2.247470
6 -2.696635 -2.235316 1.332850
6 -1.877309 2.749240 -2.621623
6 -4.924284 -2.800591 -0.265089
6 0.371010 -2.605280 -2.107404
6 -0.899678 -1.774834 -2.268542
1 -0.455537 1.134290 -2.683371
6 -3.772665 -2.956371 1.859159
1 -1.830691 -2.009382 1.955022
1 -1.338338 3.342668 -3.363421
6 -4.884515 -3.239944 1.063413
1 0.528006 -3.223565 -3.000538
1 0.295949 -3.282666 -1.243542
1 -1.742269 -2.428558 -2.532176
1 -0.771114 -1.053238 -3.087314
1 -3.738540 -3.296081 2.896444
1 -5.724149 -3.803150 1.476780
1 0.404539 -2.009285 0.906725

8 1.769953 -0.003930 2.368950
6 0.766085 0.012349 3.055758
1 -0.529185 -0.218960 2.005190
8 0.238652 0.122673 4.098935
8 5.606723 -0.441081 -0.162257
6 6.545958 -1.163172 0.598479
1 7.039492 -0.522512 1.354671
1 6.075632 -2.016459 1.124554
1 7.310129 -1.548745 -0.090653
6 -0.207279 2.678055 0.635064
8 0.547792 1.792205 0.241978
8 -1.169884 2.437439 1.490280
8 -0.115709 3.930638 0.242804
6 -2.043127 3.496137 1.926867
1 -2.740487 3.023848 2.626477
1 -1.465368 4.281848 2.430837
1 -2.583788 3.915015 1.068734
6 0.927359 4.261678 -0.690597
1 0.805433 5.328893 -0.902308
1 1.910311 4.067426 -0.240497
1 0.812750 3.669174 -1.607255

J'$_{PCO\text{-}DMC}$

6 1.151395 -0.160723 -1.323759
6 -2.428490 1.097670 -0.829944
6 -3.270783 -1.616719 -0.272367
6 -3.232589 3.787495 -0.886532
6 -4.012751 2.836105 -0.225632
6 2.790132 1.527097 -0.452555
6 -4.418624 -1.593022 -1.086444
6 4.239037 1.137682 -0.228737
6 2.503822 0.848326 -2.849151
6 1.701532 -0.015441 -3.526348
6 -3.614839 1.496109 -0.197282
1 -6.434254 -2.306712 -1.369108
1 -4.440699 -0.985103 -1.994085
1 3.302071 1.503591 -3.179537
1 4.316321 0.046132 -0.056183
1 4.606407 1.642259 0.688482
1 2.739154 2.592656 -0.723239
1 2.207385 1.388650 0.461911
1 -3.549480 4.832454 -0.914586
1 -4.939815 3.134480 0.268844
1 1.642595 -0.253962 -4.584267
1 -4.235768 0.761443 0.316980
77 0.079287 -0.789293 0.414105
7 0.889295 -0.621893 -2.578430
7 2.150966 0.747584 -1.508027
15 -1.804529 -0.630383 -0.752882
6 -1.639341 2.063598 -1.479002
6 -3.270980 -2.390291 0.894913
6 -2.041394 3.398510 -1.509621
6 -5.546663 -2.332923 -0.733034
6 -0.154842 -1.597857 -2.874119
6 -1.553059 -1.071962 -2.557248
1 -0.697620 1.776895 -1.951034
6 -4.404069 -3.131952 1.245740
1 -2.376602 -2.402226 1.519879
1 -1.419621 4.139239 -2.016912
6 -5.540581 -3.104840 0.435054
1 -0.086689 -1.843035 -3.942761
1 0.052217 -2.514741 -2.305369
1 -2.290712 -1.842670 -2.820007
1 -1.772101 -0.183106 -3.165821
1 -4.394788 -3.734701 2.156699
1 -6.424422 -3.684957 0.710172
1 0.131197 -2.270607 -0.052265
1 -0.698250 -1.335625 1.750215
8 5.004756 1.505801 -1.346883
6 6.343773 1.077691 -1.279609
1 6.870227 1.515589 -0.409479
1 6.414918 -0.025563 -1.205052
1 6.848498 1.403862 -2.199295
6 -0.473402 2.414838 1.570880

8 0.153975 1.417663 1.258514
8 -1.554245 2.444494 2.328386
8 -0.072638 3.607783 1.170705
6 -2.004404 1.213386 2.917589
1 -1.197562 0.761893 3.509187
1 -2.852081 1.486140 3.555098
1 -2.314561 0.506460 2.140541
6 -0.765339 4.787694 1.609714
1 -0.230742 5.627973 1.152839
1 -1.807960 4.767685 1.267917
1 -0.734459 4.866242 2.705059
8 1.936435 -0.934868 1.598049
6 2.926963 -1.626462 1.383485
8 3.031798 -2.390915 0.322797
8 3.972501 -1.647064 2.184794
6 4.213111 -3.186113 0.111503
1 4.375628 -3.863910 0.959592
1 4.018277 -3.755688 -0.803228
1 5.090563 -2.538357 -0.022372
6 3.911882 -0.844737 3.378865
1 3.788933 0.214396 3.115992
1 3.071783 -1.169121 4.006976
1 4.865380 -1.008592 3.891524

$J_{PCO\text{-}DMC}$

6 1.650963 -0.575641 -0.945673
6 -2.299519 1.246200 -0.550646
6 -2.833386 -1.576807 -0.521914
6 -3.516193 3.734237 -0.088032
6 -3.233485 2.881364 0.984766
6 3.631378 0.563536 0.108552
6 -4.098429 -1.314950 -1.076038
6 4.750951 -0.340900 0.589776
6 3.397467 -0.068701 -2.299635
6 2.449867 -0.665564 -3.066989
6 -2.627700 1.645141 0.757861
1 -6.110610 -2.033408 -1.375130
1 -4.281591 -0.376915 -1.605217
1 4.390922 0.296949 -2.534290
1 4.334801 -1.331273 0.861483
1 5.195491 0.095621 1.507451
1 4.048641 1.526307 -0.222347
1 2.925200 0.749459 0.922843
1 -3.987378 4.703073 0.091339
1 -3.487599 3.181816 2.003931
1 2.436830 -0.909446 -4.124977
1 -2.399107 0.984116 1.596803
7 1.389808 -0.977116 -2.223660
7 2.893224 -0.017289 -1.007553
15 -1.466932 -0.372827 -0.732176
6 -2.596255 2.099572 -1.622970
6 -2.619852 -2.775797 0.173122
6 -3.201330 3.339271 -1.390376
6 -5.129804 -2.244958 -0.943291
6 0.122131 -1.532341 -2.688716
6 -1.019900 -0.525545 -2.521354
1 -2.360663 1.809131 -2.647833
6 -3.655849 -3.705596 0.303771
1 -1.644066 -2.975643 0.616504
1 -3.429475 3.995936 -2.232940
6 -4.909389 -3.442779 -0.253498
1 0.249817 -1.787858 -3.748414
1 -0.086731 -2.466273 -2.146330
1 -1.899905 -0.839117 -3.100643
1 -0.688871 0.454013 -2.895028
1 -3.481806 -4.637065 0.847187
1 -5.718805 -4.168696 -0.147634
1 0.375340 -2.265785 0.317095
8 5.708178 -0.456685 -0.432199
6 6.734920 -1.377364 -0.148594
1 7.418802 -1.392143 -1.008566
1 7.305834 -1.088214 0.754981
1 6.335907 -2.398191 0.008392
77 0.449671 -0.735606 0.619086

8 -0.894677 -0.888341 2.372833
6 -0.465992 -1.250872 3.470510
1 -1.139135 -1.330669 4.340501
8 0.765383 -1.566689 3.716972
1 1.285410 -1.437691 2.853283
1 1.796459 -1.086883 1.524445
6 0.522886 2.460930 0.505657
8 0.548726 1.485172 1.246190
8 0.718028 2.348681 -0.791749
8 0.314178 3.691638 0.921196
6 0.660290 3.511121 -1.638179
1 1.438352 4.230615 -1.351011
1 0.840108 3.137824 -2.652042
1 -0.330431 3.978022 -1.570455
6 0.076054 3.887106 2.326826
1 -0.086038 4.962731 2.450678
1 -0.813525 3.323806 2.635638
1 0.948429 3.556583 2.906237

TS-JI$_{PCO-DMC}$

6 1.260970 -1.599768 -0.620489
6 -1.520187 1.753117 -0.765081
6 -3.200239 -0.584869 -0.651757
6 -1.629121 4.549054 -0.598407
6 -1.781407 3.771745 0.555064
6 3.450579 -1.352406 0.610043
6 -4.141499 0.008640 -1.511825
6 4.428641 -0.224680 0.329837
6 3.151592 -2.165347 -1.738107
6 2.106848 -2.353750 -2.584030
6 -1.731027 2.379438 0.476513
1 -6.207889 0.097023 -2.122274
1 -3.831845 0.776285 -2.225181
1 4.220110 -2.293990 -1.866121
1 4.894230 0.076693 1.289485
1 3.885529 0.654108 -0.066453
1 2.788661 -1.052527 1.427276
1 4.005269 -2.248934 0.925910
1 -1.668817 5.638555 -0.533913
1 -1.944102 4.253399 1.521842
1 2.075045 -2.706603 -3.610449
1 -1.844796 1.768656 1.374436
77 -0.049169 -1.015447 0.790385
7 0.958753 -2.008435 -1.884027
7 2.619928 -1.696366 -0.542509
15 -1.449319 -0.071334 -0.779461
6 -1.378352 2.534132 -1.921120
6 -3.620454 -1.546361 0.277308
6 -1.434436 3.928711 -1.835041
6 -5.482585 -0.368394 -1.451380
6 -0.376948 -1.987155 -2.473514
6 -0.946718 -0.567211 -2.482519
1 -1.222884 2.069204 -2.895806
6 -4.966685 -1.918902 0.335013
1 -2.901036 -1.988800 0.964409
1 -1.325722 4.530774 -2.739787
6 -5.896727 -1.334879 -0.527530
1 -0.286103 -2.363759 -3.499910
1 -1.032391 -2.679950 -1.924804
1 -1.815429 -0.503036 -3.152271
1 -0.175407 0.127778 -2.843599
1 -5.287965 -2.667073 1.062892
1 -6.948033 -1.627204 -0.477957
1 -0.806189 -2.356231 0.553295
6 6.287754 0.359650 -0.990919
1 5.752257 1.192277 -1.488654
1 6.850710 0.774684 -0.133145
1 6.999985 -0.079961 -1.702335
8 5.400557 -0.656927 -0.586887
8 -1.448000 -0.346660 2.280187
6 -1.121839 -0.541277 3.499293
1 -1.845765 -0.151131 4.245364
8 -0.094556 -1.112879 3.896556
1 0.573298 -1.574616 2.576950

1 0.998571 -1.951204 1.878260
6 1.444267 1.809043 0.532685
8 1.028411 0.952072 1.303516
8 1.623827 1.557384 -0.748659
8 1.759639 3.029913 0.899271
6 2.096898 2.593542 -1.631348
1 3.113439 2.897851 -1.347145
1 2.099712 2.143935 -2.629791
1 1.419440 3.455077 -1.600028
6 1.548009 3.383865 2.280261
1 1.877767 4.423737 2.369873
1 0.482603 3.291292 2.526276
1 2.139747 2.727832 2.931812

COD

6 -1.919914 0.003780 -0.015568
6 0.018135 -1.696419 -0.223920
6 -0.018134 1.696417 -0.223930
6 -1.080304 1.098098 0.668670
6 1.208688 1.231385 -0.502452
6 1.919913 -0.003778 -0.015553
6 1.080301 -1.098095 0.668681
6 -1.208684 -1.231386 -0.502454
1 -2.441242 0.450349 -0.881841
1 -2.731806 -0.305353 0.671029
1 0.311692 -2.622865 -0.732453
1 -0.648721 0.711512 1.601187
1 -1.769993 1.901083 0.974022
1 -0.311694 2.622856 -0.732474
1 1.810604 1.836325 -1.192388
1 2.441250 -0.450350 -0.881819
1 2.731801 0.305357 0.671049
1 0.648714 -0.711512 1.601199
1 1.769990 -1.901079 0.974039
1 -1.810598 -1.836331 -1.192388

cis-COE

6 1.816568 0.359674 -0.147180
6 -0.374044 1.756157 -0.082597
6 0.337363 -1.776764 0.027229
6 1.314533 -0.804927 0.715874
6 -0.804984 -1.171317 -0.799046
6 -1.845203 -0.345420 -0.037577
6 -1.297244 0.878631 0.730002
6 0.895916 1.517846 -0.446333
1 2.183314 -0.038289 -1.114020
1 2.721147 0.783037 0.328789
1 -0.815187 2.688940 -0.453841
1 0.888107 -0.412281 1.649865
1 2.195048 -1.390872 1.028820
1 0.924498 -2.420780 -0.651308
1 -1.332659 -1.998995 -1.303769
1 -2.602777 0.000154 -0.762379
1 -2.380139 -0.993191 0.679696
1 -0.796583 0.537048 1.646408
1 -2.153439 1.482305 1.070358
1 1.373552 2.289132 -1.064808
1 -0.082950 -2.453901 0.792574
1 -0.379357 -0.555595 -1.608618

FA

6 0.000000 0.418382 0.000000
1 -0.377190 1.462650 0.000000
8 1.160107 0.104526 0.000000
8 -1.029255 -0.433582 0.000000
1 -0.669624 -1.340491 0.000000

DMC

6 -0.000001 0.076170 -0.000020
8 -0.000006 1.283890 -0.000014
8 1.081777 -0.704122 -0.000016
8 -1.081775 -0.704124 -0.000014
6 2.337578 -0.023626 0.000023

1 2.439636 0.608280 0.894762
1 3.105581 -0.806007 -0.000042
1 2.439616 0.608413 -0.894623
6 -2.337574 -0.023629 0.000024
1 -3.105577 -0.806009 0.000210
1 -2.439532 0.608474 0.894634
1 -2.439713 0.608213 -0.894752

H_2

1 0.000000 0.000000 0.380535
1 0.000000 0.000000 -0.380535

CO_2

6 0.000000 0.000000 0.000000
8 0.000000 0.000000 1.162967
8 0.000000 0.000000 -1.162967

12. References

1. Frisch, M. J.; Trucks, G. W.; Schlegel, H. B.; Scuseria, G. E.; Robb, M. A.; Cheeseman, J. R.; Scalmani, G.; Barone, V.; Mennucci, B.; Petersson, G. A.; Nakatsuji, H.; Caricato, M.; Li, X.; Hratchian, H. P.; Izmaylov, A. F.; Bloino, J.; Zheng, G.; Sonnenberg, J. L.; Hada, M.; Ehara, M.; Toyota, K.; Fukuda, R.; Hasegawa, J.; Ishida, M.; Nakajima, T.; Honda, Y.; Kitao, O.; Nakai, H.; Vreven, T.; Montgomery, J. A., Jr.; Peralta, J. E.; Ogliaro, F.; Bearpark, M.; Heyd, J. J.; Brothers, E.; Kudin, K. N.; Staroverov, V. N.; Kobayashi, R.; Normand, J.; Raghavachari, K.; Rendell, A.; Burant, J. C.; Iyengar, S. S.; Tomasi, J.; Cossi, M.; Rega, N.; Millam, J. M.; Klene, M.; Knox, J. E.; Cross, J. B.; Bakken, V.; Adamo, C.; Jaramillo, J.; Gomperts, R.; Stratmann, R. E.; Yazyev, O.; Austin, A. J.; Cammi, R.; Pomelli, C.; Ochterski, J. W.; Martin, R. L.; Morokuma, K.; Zakrzewski, V. G.; Voth, G. A.; Salvador, P.; Dannenberg, J. J.; Dapprich, S.; Daniels, A. D.; Farkas, Ö.; Foresman, J. B.; Ortiz, J. V.; Cioslowski, J.; Fox, D. J. *Gaussian 09*, Revision D.01; Gaussian, Inc.: Wallingford CT, 2009.
2. Becke, A. D. A new mixing of Hartree–Fock and local density-functional theories, *J. Chem. Phys.* **1993**, *98*, 1372−1377.
3. (a) Grimme, S.; Antony, J.; Ehrlich, S.; Krieg, H. A consistent and accurate *ab initio* parametrization of density functional dispersion correction (DFT-D) for the 94 elements H-Pu. *J. Chem. Phys.* **2010**, *132*, 154104. (b) Johnson, E. R. E.; Becke, A. D. A. A post-Hartree–Fock model of intermolecular interactions. J. Chem. Phys. **2005**, *123*, 024101.
4. Weigend, F.; Ahlrichs, R. Balanced basis sets of split valence, triple zeta valence and quadruple zeta valence quality for H to Rn: Design and assessment of accuracy. *Phys. Chem. Chem. Phys.* **2005**, *7*, 3297−3305.
5. Scalmani, G.; Frisch, M. J. Continuous surface charge polarizable continuum models of solvation. I. General formalism. *J. Chem. Phys.* **2010**, *132*, 114110.
6. Tanaka, R.; Yamashita, M.; Chung, L. W.; Morokuma, K.; Nozaki, K. Mechanistic Studies on the Reversible Hydrogenation of Carbon Dioxide Catalyzed by an Ir-PNP Complex. *Organometallics* **2011**, *30*, 6742−6750.
7. CYLview, 1.0b; Legault, C. Y., Ed.; Université de Sherbrooke, 2009.
8 *SAINT+: Area-Detector Integration Software*, version 6.01; Bruker AXS: Madison, WI, 2001.
9 Sheldrick, G. M. *SADABS program*; University of Göttingen: Göttingen, Germany, 1999.
10 G. M. Sheldrick, *SHELXS 97, Program for the Solution of Crystal Structure*; University of Göttingen: Göttingen, Germany, 1997.
11 Sheldrick, G. M. Crystal structure refinement with SHELXL. Acta Crystallogr., Sect. C: Struct. Chem. **2015**, *71*, 3−8.
12 Farrugia, L. J. WinGX and ORTEP for Windows: an update. *J. Appl. Crystallogr.* **2012**, *45*, 849−854.
